# Effects on gene expression during maize-*Azospirillum* interaction in the presence of a plant-specific inhibitor of indole-3-acetic acid production

**DOI:** 10.1590/1678-4685-GMB-2023-0100

**Published:** 2023-09-18

**Authors:** Eliandro Espindula, Edilena Reis Sperb, Brenda Moz, Vânia Carla Silva Pankievicz, Thalita Regina Tuleski, Michelle Zibetti Tadra-Sfeir, Paloma Bonato, Camila Scheid, Josias Merib, Emanuel Maltempi de Souza, Luciane Maria Pereira Passaglia

**Affiliations:** 1 Universidade Federal do Rio Grande do Sul (UFRGS), Instituto de Biociências, Departamento de Genética, Programa de Pós-Graduação em Genética e Biologia Molecular, Porto Alegre, RS, Brazil.; 2 Universidade Federal do Paraná (UFPR), Centro Politécnico, Departamento de Bioquímica e Biologia Molecular, Curitiba, PR, Brazil.; 3 Universidade Federal de Ciências da Saúde de Porto Alegre (UFCSPA), Programa de Pós-Graduação em Biociências, Porto Alegre, RS, Brazil.; 4 Universidade Federal de Ciências da Saúde de Porto Alegre (UFCSPA), Departamento de Farmacociências, Programa de Pós-Graduação em Biociências, Porto Alegre, Brazil.

**Keywords:** Dual RNA-Seq, Zea mays, Plant Growth-Promoting Bacteria, ABA, yucasin

## Abstract

Amongst the sustainable alternatives to increase maize production is the use of plant growth-promoting bacteria (PGPB). *Azospirillum brasilense* is one of the most well-known PGPB being able to fix nitrogen and produce phytohormones, especially indole-3-acetic acid - IAA. This work investigated if there is any contribution of the bacterium to the plant’s IAA levels, and how it affects the plant. To inhibit plant IAA production, yucasin, an inhibitor of the TAM/YUC pathway, was applied. Plantlets’ IAA concentration was evaluated through HPLC and dual RNA-Seq was used to analyze gene expression. Statistical differences between the group treated with yucasin and the other groups showed that *A. brasilense* inoculation was able to prevent the phenotype caused by yucasin concerning the number of lateral roots. Genes involved in the auxin and ABA response pathways, auxin efflux transport, and the cell cycle were regulated by the presence of the bacterium, yucasin, or both. Genes involved in the response to biotic/abiotic stress, plant disease resistance, and a D-type cellulose synthase changed their expression pattern among two sets of comparisons in which *A. brasilense* acted as treatment. The results suggest that *A. brasilense* interferes with the expression of many maize genes through an IAA-independent pathway.

## Introduction

Plant Growth-Promoting Bacteria (PGPB) are a group of beneficial microorganisms that can colonize the rhizosphere, the phyllosphere, the root’s surface, and the plant’s internal tissues stimulating plant growth (Verna et al., 2010; Khabbaz et al., 2019). It is believed that these bacteria can promote plant growth through the combination of several abilities, like biological nitrogen fixation, production of phytohormones (especially indole-3-acetic acid - IAA), vitamins, and growth factors (Babalola, 2010; Bashan and de-Bashan, 2010; Khabbaz *et al.*, 2019).

*Azospirillum brasilense* is one of the most well-known PGPB being widely used in South America as a cereal crop inoculant. Amongst the plant growth-promoting traits of this bacterium, the most studied are the ability to fix nitrogen and to produce phytohormones (IAA, gibberellins, ethylene, and polyamines) (Cassán et al., 2014; Cassán and Diaz-Zorita, 2016; Fukami et al., 2018; Cassán *et al.*, 2020). It is believed that these two characteristics are responsible for stimulating the increase of the final dry mass of the plants (Bashan and de-Bashan, 2010; Cassán *et al.*, 2020). Among the phytohormones produced by PGPB and plants, auxins (mainly IAA) are the most studied (Spaepen et al., 2007; Baudoin et al., 2010; Spaepen and Vanderleyden, 2010; Yue et al., 2014). The main biosynthetic pathway in both plant and PGPB uses tryptophan (Trp) as a precursor for IAA synthesis (Spaepen *et al*., 2007). In bacteria, this molecule is involved in the quorum-sensing process, which permits them to control their activities based on population density (Spaepen *et al*., 2007; Duca et al., 2014; Yue *et al*., 2014). Although many studies show IAA’s importance in plant-growth promotion, others showed that the *Azospirillum* IAA biosynthesis alone cannot account for the overall plant growth-promoting effect observed (Spaepen *et al*., 2007; Bashan and de-Bashan, 2010). Furthermore, according to Cassán *et al.* (2020), there is evidence that plant growth promoted by *Azospirillum* sp can be both IAA-dependent and IAA-independent. In plants, auxins are responsible for regulating various aspects of their development, such as cell growth and differentiation, the establishment of apical dominance, differentiation of xylem, suppression of abscission, and formation of apical and root meristem (Bishopp et al., 2006; Yue *et al*., 2014). 

Over the years, several IAA biosynthetic pathways using Trp as a precursor have been proposed to explain how plants produce this hormone (Zhao, 2010). Recently studies have indicated that the tryptophan aminotransferase (TAA) and the YUC-flavin monooxygenases families are part of the main pathway of IAA production in plants: TAA converts tryptophan to indole - 3 - pyruvate (IPA), which is converted into IAA by YUC (Mashiguchi et al., 2011; Won et al., 2011; Zhao, 2012; Zhao, 2014; Yue et al., 2014). It was observed by Nishimura et al. (2014) that yucasin [5-(4-chlorophenyl)-4H-1,2,4-triazol-3-thiol] is a competitive inhibitor of YUC, preventing IPA decarboxylation. Since yucasin inhibits the production of IAA by this route, it can be used in studies of modulation of IAA production by the plant over time (Zhao, 2014).

In the present work, the interaction between *Azospirillum brasilense* FP2 and maize was investigated under co-cultivation in the presence of the TAA/YUC pathway inhibitor, yucasin. The use of this inhibitor was made to access if there is any contribution of the bacterium to the IAA levels in the plant, and how it affects the plant. The plantlets’ IAA concentration was accessed by HPLC. Gene expression patterns of the bacterium and plant were analyzed by dual RNA-Seq, and data obtained from the sequencing of plant and bacterium transcriptomes were subjected to a combined analysis approach. The IAA concentration alongside the pattern of genes differentially expressed in maize brings to light evidence of the existence of an IAA-independent pathway for plant-growth promotion by the bacterium.

## Material and Methods

### Bacterial strain and growth curve conditions

*A. brasilense* strain FP2 is a natural mutant originating from strain Sp7 (ATCC29145) that presents resistance to nalidixic acid and streptomycin antibiotics (Pedrosa and Yates, 1984). *A. brasilense* FP2 growth curves were obtained by inoculation of 2 mL of 24 h bacterial pre-culture in 250 mL Erlenmeyer’s flask containing 100 mL of King B medium (Glickmann and Dessaux, 1995) supplemented or not with 50 µM of yucasin [5-(4-chlorophenyl)-4H-1,2,4-triazole-3-thiol; register number CAS: 26028-65-9] to reach an initial OD_600nm_ of 0.02. Cultures were incubated in an orbital shaker at 30^o^C and 120 rpm. Samplings were taken after 2, 4, 6, 26, 30, and 32 h of bacterial growth for CFU calculation. To measure the bacterial indole compounds (ICs) production, as an indirect way of measuring IAA concentration, 2 mL of bacterial culture from each sampling time was centrifuged for 5 min at 12,000 *g*. The supernatant was collected and mixed with the Salkowski reagent at the proportion of 1:2, respectively (Glickmann and Dessaux, 1995). Bacterial ICs production was measured as described by Ambrosini et al. (2012).

### Seed inoculation, experimental conditions, and physiological experiment

The bacterial suspension was prepared by growing *A. brasilense* FP2 in 30 mL of NFb medium supplemented with 5 mg L^-1^ of malic acid (Pedrosa and Yates, 1984) in an orbital shaker (30^o^C, 120 rpm) until an OD_600_ of 0.8 [~10^8^ cells mL^-1^ (Faleiro et al., 2013)]. Aliquots of 3 mL of the culture were centrifuged, and the pellets were suspended in NFb medium without nitrogen. 

*Zea mays* (var. Santa Helena SHS4080) seeds were surface-sterilized by washing them three times with autoclaved ultrapure water, followed by submersion in 70% ethanol for 3 min and in a solution of 2% sodium hypochlorite and 2.5% Tween 20 for 30 min. Seeds were then washed three times with sterile distilled water by gentle shaking (Faleiro et al., 2013). 

The experiment was divided into four groups: Ctr (control plantlets), Yuc {plantlets that received 50 µM of yucasin solution [concentration according to Nishimura et al. (2014)]}, Azo (plantlets inoculated with*A. brasilense* FP2), and AzoYuc (plantlets that received 50 µM of yucasin solution and were inoculated with *A. brasilense* FP2). For inoculation, seeds (0.1 g) were mixed with 0.5 mL of bacterial suspension containing ~3 x 10^8^ bacterial cells mL^-1^ (Hungria et al., 2010; Espindula et al., 2017) and incubated for 5 min in an orbital shaker at 100 rpm (Faleiro et al., 2013). Seeds inoculated or not were placed in a sterilized water-saturated paper and maintained for three days in a 25^o^C growth chamber in the dark for germination. Maize seedlings were then transferred to pots containing sterilized sand wet with plant medium solution (Egener et al., 1999) without nitrogen. Azo and AzoYuc groups were formed with the inoculated seedlings and Ctr and Yuc with the non-inoculated ones. The plantlets were kept in a growing chamber for 10 days (25^o^C, 16 h light/8 h dark, with active photosynthetic radiation of 150 μmol m^2^s^-1^) (Faleiro *et al.*, 2013; Espindula *et al.*, 2017). After this period, each plant from Yuc and AzoYuc groups received 10 mL of an aqueous solution (autoclaved ultrapure water) of yucasin (50 µM final concentration), which was added directly to the soil. Plants from Ctr and Azo groups received only sterilized water. The experiment was carried out with three biological replicates. Each replicate consisted of 20 *Z. mays* plantlets. After five hours under yucasin treatment roots were washed twice with sterilized water, separated from the aerial part, and stored at -80^o^C until RNA extraction and electron microscopy analysis.

To access the physiological effects of the yucasin on the plant’s development, another four groups of maize plantlets were prepared as previously described. Ten days plantlets from Yuc and AzoYuc groups were then daily supplemented with 50 µM of yucasin solution for additional five days, while plantlets from Ctr and Azo groups received only sterilized water. After this period, the lengths of the aerial parts and the main root (the longest one), and the number of lateral roots of each plantlet were evaluated.

### Endogenous auxin quantification by High-Performance Liquid Chromatography (HPLC)

The quantification of endogenous auxin content of plantlets was performed according to Kim et al. (2006) and Vilasboa et al. (2019), with the following modifications. Approximately 200 mg of maize roots were ground with liquid nitrogen and extracted with 100% methanol (2.5 mL g^-1^ FW), followed by centrifugation for 10 min at 16,000 *g* at 4^ ^°C. The supernatant was transferred to a new tube and concentrated in Speed Vac (Christ RVC 2-18 CDPlus) until approximately one-tenth of the initial volume. The remaining solution was resuspended in 200 μL of distilled and deionized water. 

The pH of the solution was adjusted to above 9 with 1 M KOH and then partitioned against 100% ethyl acetate (1:1 v/v). The aqueous and organic phases were separated by centrifugation (16,000 *g* x 5 min). The lower aqueous phase was transferred to a new tube and the pH of the solution was lowered to below 3 with concentrated acetic acid to keep IAA in protonated form. After the solution was partitioned with 100% ethyl acetate (1:1 v/v) and the phases were separated by centrifugation. The upper organic phase was transferred to new tubes, completely dried in Speed Vac, and resuspended in 100 μL 100% HPLC grade methanol.

The samples and the calibration curve were analyzed using an HPLC system (Prominence, Shimadzu) equipped with a Kinetex-Phenomenex C_18_ HPLC reverse-phase column (150 x 4.6 mm x 5 µm). The mobile phases were based on the proposed by Kim et al. (2006) with some modifications, consisting of an aqueous solution of acetic acid at 0.3% v/v (mobile phase A), and a methanolic solution of acetic acid at 0.3% v/v (mobile phase B) at a flow rate of 1 mL min^-1^. The gradient of the mobile phase was adopted as follows: 0 - 5 min using 15% of B; from 5 - 15 min mobile phase B was increased to 100% maintaining up to 17 min; returning to the initial condition at 22 min and maintaining this condition up to 30 min. The column oven temperature was kept at 30⁰C for all analyses. A fluorescence detector Shimadzu RF-20A (emission at 360 nm, excitation at 282 nm) was used to detect indole-3-acetic acid (IAA). A standard curve was generated using purified IAA (Neon) and 20 μL of each sample was analyzed using an autosampler Shimadzu SIL-20A. For this analysis, three biological replicates were analyzed for each experimental group.

### Scanning electron microscopy (SEM)

To visualize *A. brasilense* cells attached to maize roots, two *Zea mays* roots from Azo and AzoYuc groups were fixed with Karnovsky’s (Karnovsky, 1964) fixative and washed in an alcoholic series (20, 40, 60,70, 80, 90, 96, and 100%) during 30 min at each concentration. After complete dehydration, the samples were dried at the critical point of CO_2_ at the equipment Leica EM CDP 300 (Haddad et al., 2007). The dried samples were attached to aluminum supports with the aid of double-sided carbon tape and were carbon-coated at Baltec’s Sputter Coater, model CED 005, for an ultrastructural study. The analysis was conducted in a Scanning Electron Microscope FEI, model Inspect F50, at the Central Laboratory for Microscopy and Microanalysis, Pontifical Catholic University of Rio Grande do Sul (LabCEMM-PUC/RS), Brazil.

### RNA isolation, mRNA enrichment, cDNA synthesis, and sequencing

Total RNA was isolated from 0.1 g of plant tissue from a pool of 20 maize roots for each biological replicate from each treatment. There were three RNA extractions per experimental group, and one per biological replicate, totalizing 12 RNA samples. Total RNA was isolated by RNeasy Plant Mini Kit® (Qiagen, CA, USA). The RNA concentration and purity were determined by spectrophotometry at 260 nm and 280 nm (Jahn et al., 2008) measured in a Nanodrop LITE spectrophotometer (Thermo Fisher Scientific, Wilmington, DE, USA). Then, samples were first treated with DNaseI (Invitrogen), and the rRNA pool was depleted using the RiboMinus™ Plant Kit for RNA-Seq (Invitrogen). The cDNA libraries were constructed using the Ion Total RNA-Seq kit v2 for Whole Transcriptome Library. All RNA quantification and quality evaluation were performed at the Bioanalyzer™ - Agilent 2100 instrument. Each cDNA library obtained was sequenced using the Ion PI Template OT2 200 Kit v3 and the Ion PI Sequencing 200 Kit v3 at the IonTorrent® platform (Thermo FisherScientific, Wilmington, DE, USA). All kits and reagents were used according to the manufacturer’s instructions. The 12 cDNA libraries obtained in this work were deposited in GenBank under the numbers SAMN12391479 to SAMN12391490.

### Data analysis and differential gene expression

The reference genomes and the respective annotations were downloaded from the National Center for Biotechnology Information (NCBI) site. All the cDNA libraries obtained and the reference genomes with annotations were uploaded into the CLC Genomics Workbench (v. 8.0). Reads smaller than 20 nucleotides and with low quality were removed from libraries using the standards setup of CLC Genomics. Ctr and Yuc groups cDNA libraries were mapped against the *Z. mays* cv. B73 (GCF_000005005.2) genome and the Azo and AzoYuc groups cDNA libraries were mapped against a Combined reference file formed by the merging of *Z. mays* cv. B73 and *A. brasilense* Sp7 (GCA_001315015.1) genomes. The mapping parameter used was 0.8 of minimum length fraction and 0.8 of minimum similarity fraction for inclusion as a mapped read. The mapped reads were extracted and counted using the respective annotated genome. The counting parameters used were: 0.8 of minimum length fraction and 0.8 of minimum similarity fraction; with a mismatch, insertion, and deletion costs of 2, 3, and 3, respectively, for inclusion as a mapped read, allowing a maximum of 10 hits, with the exclusion of the reads that mapped to intergenic regions (Espindula et al., 2019, with modifications). 

Count files were analyzed with the DESeq2 v 3.8 (Love et al., 2014) package of R software v 3.5.2 (R Development Core Team). Genes with p-values < 0.05 (Li et al., 2019; Yoo et al., 2019) and log2fold-change [Lg2(FC)] ≥ |1.5| were considered as differentially expressed (Li *et al.*, 2019, with modifications). Metabolic pathways were identified using the Kyoto Encyclopedia of Genes and Genomes (KEGG) database (https://www.genome.jp/kegg/). Annotations for the DEGs were made with the help of the MaizeMine, online version 1.3 (http://maizemine.rnet.missouri.edu:8080/maizemine/begin.do). Heatmaps were generated using the ComplexHeatmap (Gu et al., 2016) package of R software v 3.5.2 (R Development Core Team).

### Statistical analyses

For root and aerial parts length and number of lateral roots analysis, 10 biological repeats were used. Tukey test was used to detect differences among the means of the treatments for each physiological characteristic. For IAA quantification analysis, three biological repeats were used. Dunn’s test was used to detect differences among the means of the treatments. 

For all statistical analyses, the package Agricolae was used on R software v 4.2.0 (R Development Core Team, https://www.r-project.org/).

## Results

The presence of yucasin in the culture medium did not affect bacterial growth and indole compounds (ICs) production

**Figure 1 -:**
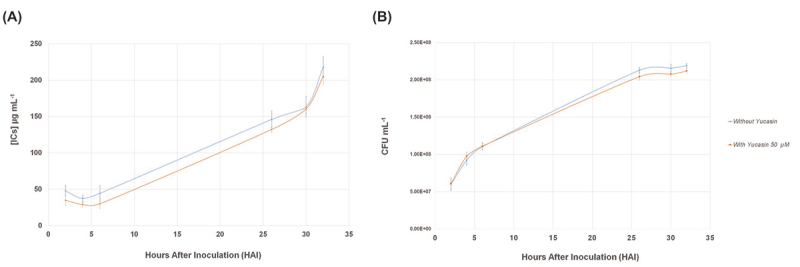
(A) Amount of ICs produced over time by *Azospirillum brasilense* FP2 in King B medium supplemented with tryptophan and with or without yucasin. (B) Growth curves (CFU mL^-1^) of *A. brasilense* FP2 in King B medium supplemented or not with yucasin.

To verify if the IAA inhibitor synthesis, yucasin, could interfere with the growth and ICs production of *A. brasilense* FP2, bacterial growth, and ICs production in the presence of this compound were analyzed. The results showed that the presence of yucasin in the King B medium affected neither ICs production (Figure 1A) nor *A. brasilense* FP2 growth (Figure 1B).

### Quantification of the indole-3-acetic acid present in the plantlet’s roots

**Figure 2 -:**
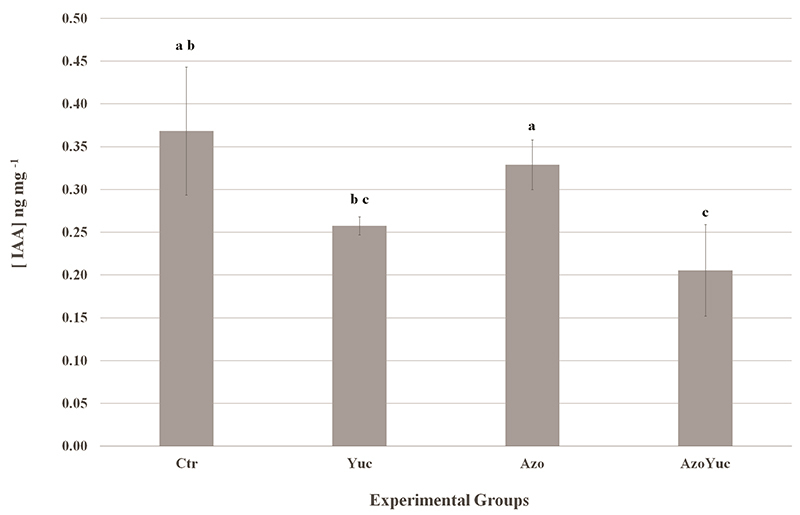
Plantlet roots endogenous IAA concentration in each experimental group. Bars show standard error. Different letters indicate groups statistically different. Means were considered statistically different when the p-value <0.05 according to Dunn’s Test.

To determine the concentration of IAA present in the samples after the treatments, the endogenous auxin concentration in the plantlet’s roots was quantified by HPLC. The results showed that the IAA concentration in plantlets from the AzoYuc group was lower than those in plantlets from the Azo and Ctr groups, while the IAA concentration in the plantlets from the Yuc group was lower than the concentration presented in the plantlets from the Azo group (Figure 2).

###  The plant growth-promoting effect of *Azospirillum* was not affected by the presence of yucasin 

**Figure 3 -:**
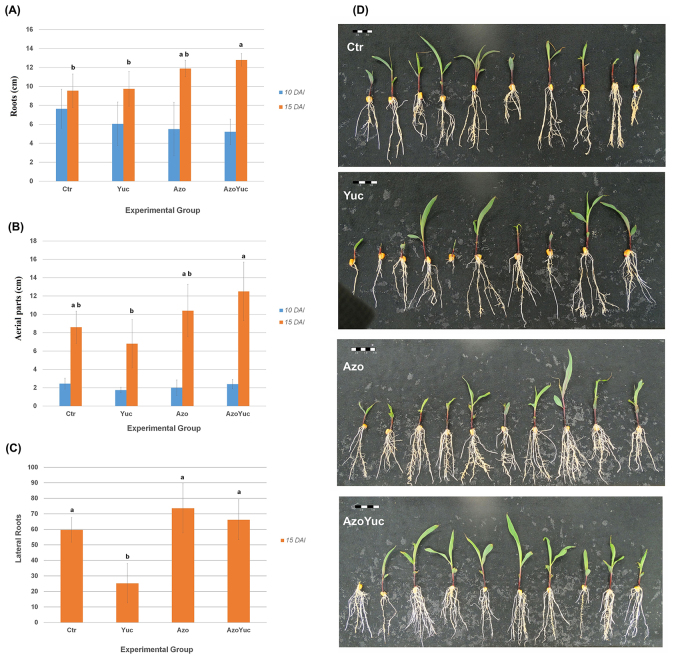
Lengths of the roots (A), aerial parts (B), and the number of lateral roots (C) of maize plantlets according to each experimental group. Bars show twice the standard error. Different letters indicate groups statistically different. Means were considered statistically different when p-adjusted <0.05 according to Tukey’s test. (D) Plantlets from experimental groups: Ctr, Yuc, Azo, and AzoYuc at 15 DAI. Bars represent the scale, where each block has 1.0 cm. DAI = days after inoculation. Ctr = control plantlets; Yuc = plantlets that received 50 µM of yucasin; Azo = plantlets inoculated with *A. brasilense* FP2; AzoYuc = plantlets that received 50 µM of yucasin and were inoculated with *A. brasilense* FP2.

To evaluate if the plant growth-promoting effect of *Azospirillum brasilense* could be affected by the presence of yucasin, maize seedlings were treated with yucasin 10 days after inoculation (DAI) with *A. brasilense*. The first measurement was made 5 hours after yucasin addition, and no significant result was observed for any parameter evaluated (Figure 3A). The next measurement was made 15 DAI, and some differences could be observed among the groups. Concerning the root lengths, although the average length of the roots of the Azo group was longer than those of the Ctr and Yuc groups, this difference was not significant. In its turn, plantlets from the AzoYuc group presented significantly longer roots than those from the Ctr and Yuc groups (Fig 3A). Concerning the length of the aerial part of plantlets (Figure 3B), the mean length from those of the AzoYuc group was statistically different from those of the Yuc group. We also evaluated the effect of the treatments on the number of lateral roots. At 15 DAI plantlets from the Yuc group presented a significantly lower number of lateral roots than plantlets from the other groups (Figure 3C). This result can also be observed in Figure 3D, in which plantlets from the Yuc group visually showed fewer lateral roots than plantlets from the other groups. 

To confirm the presence of *A. brasilense* FP2 on maize’s root surface, scanning electron microscopy analysis was performed using samples from biological replicates of the Azo and AzoYuc groups from the first sampling time point (10 DAI). The bacterium was observed on the root surfaces of plants in both experimental groups (Figure S1). 

### Transcriptome analysis

To investigate the gene expression pattern of maize and *A. brasilense* during their co-cultivation in the presence of the TAA/YUC pathway inhibitor, yucasin, biological samples of all experimental groups were used for RNA extraction. Total RNA was extracted from each biological replicate of each experimental group, generating 12 cDNA libraries. Table S1 shows a summary of library mapping for each experimental group. 

Reads that mapped to the *A. brasilense* and *Z. mays* reference genomes were extracted from libraries of Azo and AzoYuc groups and reads counting was performed separately using the respective annotated genomes. Since Ctr and Yuc groups were not inoculated, reads that mapped to the *Z. mays* reference genome were extracted and counted using its annotated genome. All reads that aligned in the intergenic regions, tRNA, and rRNA sequences were eliminated, and only the reads that aligned to coding sequences (CDS) were further analyzed. As can be observed in Table 1, the majority of reads that mapped to the maize genome corresponded to multireads. According to Mortazavi et al. (2008), multireads are reads that align equally well to several sites in the genome and are attributed to members of multigene families, duplicated genes, or segmental duplications. Since these reads aligned to CDS, and not to rRNA or tRNA, we concluded that the presence of multireads must be because *Z. mays* is an allopolyploid plant (Messing, 2009), presenting several copies of many genes. Thus, these reads were used for further analyses since much of the transcriptional information could be lost if they were discarded (Mortazavi *et al.*, 2008). On the other hand, the majority of reads that mapped to the *A. brasilense* genome corresponded to unique mapped reads. This result was expected since prokaryote genomes are mostly formed by single-copy genes, except for rRNA and tRNA genes.

**Table 1 -:** Number of reads mapped to tRNA, rRNA, and coding sequences (CDS) in each experimental group.

Experimental Group	Library formed by the Reads that Mapped to	Total number of mapped reads	Coverage	Number of Reads Mapped to				Unmapped Reads
				tRNA	rRNA	CDS loci		
						Unique Reads	Total Reads	
Ctr	*Zea mays*	45,111,393	2.6	11,826	1,949,984	810,125	24,786,788	18,362,795
Yuc	*Zea mays*	48,782,996	2.8	7,736	2,022,472	1,043,121	29,210,896	17,541,892
Azo	*Azospirillum brasilense*	84,989	2.8	1,492	77,728	4,561	4,942	827
	*Zea mays*	50,147,339		10,483	2,538,964	1,267,707	25,016,612	22,581,280
AzoYuc	*Azospirillum brasilense*	106,413	2.0	1,753	94,841	7,762	8,420	1,399
	*Zea mays*	40,182,473		11,553	2,317,501	1,300,353	17,509,066	20,344,353

**Figure 4 -:**
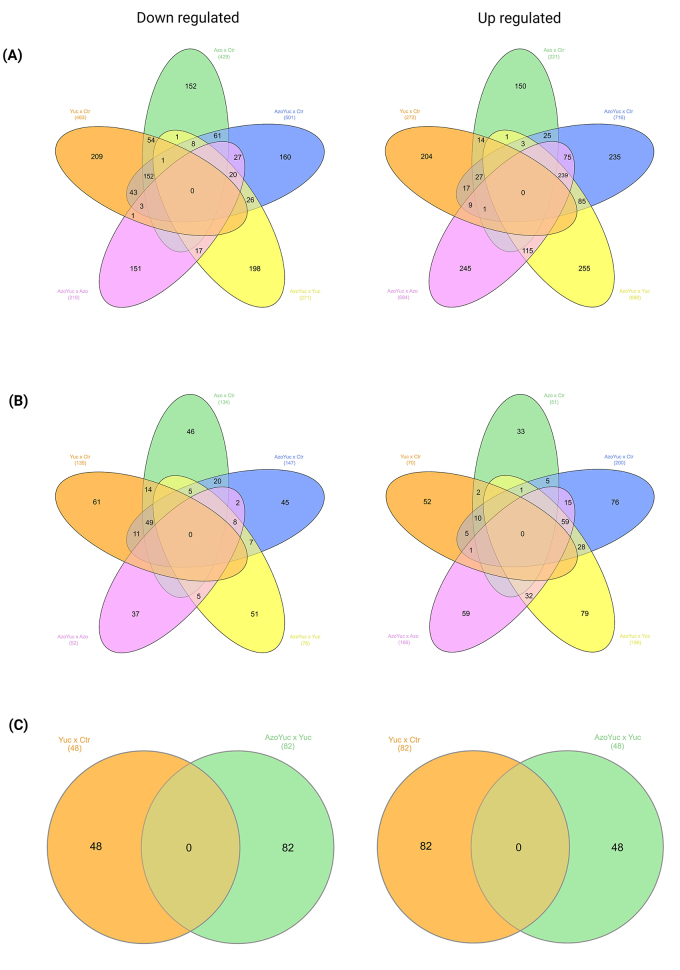
Venn diagrams showing the (A) total of differentially expressed genes (DEG) and (B) total of uncharacterized differentially expressed genes (UDEG) in all experimental situations, and (C) the differentially expressed genes that changed their pattern of expression from being down-regulated to up-regulated, and vice versa, when comparing two experimental conditions (Yuc x Ctr and AzoYuc x Yuc). Ctr = control plantlets; Yuc = plantlets that received 50 µM of yucasin; Azo = plantlets inoculated with *A. brasilense* FP2; AzoYuc = plantlets that received 50 µM of yucasin and were inoculated with *A. brasilense* FP2. Venn’s diagrams were constructed with InteractiVenn [http://www.interactivenn.net/index.html (Heberle et al., 2015)]. Numbers inside parentheses indicate the total amount of DEG or UDEG. Figure created with BioRender.com.

In maize, at least 650 differentially expressed genes (DEGs) were identified in pairwise comparisons between the experimental conditions (Table S2). The comparison between the transcriptional patterns from the Yuc and Azo groups with that of the Ctr group showed that several DEGs were down-regulated. For the other comparisons, the majority of the DEGs were up-regulated (Figure 4A and Table S2 bottom).

Among all the comparisons analyzed, we identified a total of 763 uncharacterized *loci* being differentially expressed in at least one comparison (Figure 4B and Table S3). Depending on the experimental condition, at least 83 loci presented a high difference in relative expression level [Log2(FC)> | 3 |] (Table S3 bottom). Most of them were down-regulated when comparing data from the Yuc and Azo groups with data from the Ctr group. On the other hand, when comparing data from the AzoYuc group with data from the other groups most of the uncharacterized differentially expressed genes were up-regulated (Table S3 bottom).

When analyzing our data, we noticed that some genes related to the maize auxin response pathway and auxin efflux transport were differentially expressed in response to the presence of *A. brasilense* and yucasin (Tables 2 and S2). Some of them are highlighted below.

**Table 2 -:** Maize differentially expressed genes (DEGs) in all experimental conditions highlighted in this study. Numbers represent Log2(Fold Change). Genes descriptions were obtained using the MaizeMine databank at https://maizemine.rnet.missouri.edu/maizemine/begin.do. Ctr = control plantlets; Yuc = plantlets that received 50 µM of yucasin; Azo = plantlets inoculated with *A. brasilense* FP2; AzoYuc = plantlets that received 50 µM of yucasin and were inoculated with *A. brasilense* FP2. Genes are ordered according to the “Gene Description” column.

Gene ID	Gene Symbol	Gene Description	Yuc x Ctr	Azo x Ctr	AzoYuc x Ctr	AzoYuc x Yuc	AzoYuc x Azo
542307	cyc1	*cyclin 1 ( cyc 1b)*	-	2.190359524	-	-	-2.419524577
103637736	LOC103637736	*rth6 (roothairless6)*	-1.736659862	-	-	2.27833168	1.670676432
100282128	sca1	*Zmaba2* (aka sca1 - short-chain alcohol dehydrogenase 1)	1.869562727	-	-	-1.28031897	-
103642400	LOC103642400	*Zmarf1 (Arf-transcription factor 1)*	-	-	1.54978967	-	-
100383226	ARF4	*Zmarf4 (Arf-transcription factor 4)*	2.851759264	-	-	-	-
100857063	LOC100857063	*Zmarf7 (Arf-transcription factor 7)*	-	-	-	-	1.503674008
103654892	ARF16	*Zmarf16 (Arf-transcription factor 16)*	-1.875247704	-	-	-	-
100502480	LOC100502480	*Zmarf18 (Arf-transcription factor 18)*	-	-	-	-	1.509544366
100280136	LOC100280136	*Zmarf19 (Arf-transcription factor 19)*	- 1.576947318	-	-	-	-
103629639	LOC103629639	*Zmarf22 (Arf-transcription factor 22)*	-	-	1.74968193	-	-
103630727	LOC103630727	*Zmarf24 (Arf-transcription factor 24)*	-	-1.778205534	-1.52748748	-	-
100273501	LOC100273501	*Zmarf25 (Arf-transcription factor 25)*	-	-	-	-1.630251914	-
103627702	LOC103627702	*Zmdel1c* (member of E2F transcription factor family - DEL type)	-	-	2.91547323	2.769133717	3.192241895
100193303	cl2682_1	*Zmgh3.10*	-	-	2.274154707	2.055318037	1.594515622
100274580	AUX18	*Zmiaa7* - Aux/IAA-transcription factor 7	-	-	1.93213496	-	-
100284457	LOC100284457	*Zmiaa10/rum1* - Aux/IAA-transcription factor 10/rum1 (rootless with undetectable meristems1)	-	-	-	2.009740326	2.306496583
100194253	AUX22	*Zmiaa27/bif1* - Aux/IAA-transcription factor 27/bif1	-	3.145192611	-	-	-2.228641457
100193847	LOC100193847	*Zmick4* -Inhibitor of cyclin-dependent kinase	-	-	-	3.15901329	2.768899721
109946070	LOC109946070	*Zmmkk5* (mitogen-activated protein kinase kinase 5)	3.511030994	-	-	-2.279009933	-
541618	LOC541618	*Zmmpk5* (mitogen-activated protein kinase 5)	1.954828547	-	-	-2.371341104	-
103654258	LOC103654258	*Zmpin1c* (PIN-formed protein 1c)	-	-	1.801325014	1.562291256	-
100285745	cl464_-1	*Zmpin1d* (PIN-formed protein 1d)	-	-	-	3.159435887	-
100192073	PP2C14	*Zmpp2c14* (protein phosphatase 2C 14)	-	2.110871763	-	-	-2.138141138
100282657	LOC100282657	*Zmpp2c3* (protein phosphatase 2C 3)	-	1.622090607	-	-	-1.734429497
100381549	PP2C4	*Zmpp2c4* (protein phosphatase 2C 4)	-	2.330389372	-	-	-1.711966087
103634514	LOC103634514	*Zmpyl7*	2.190269587	-	2.386251179	-	3.338990236
103653849	LOC103653849	*Zmsaur41*-auxin-responsive SAUR family member	-	4.8360325	-	-	-
100277080	LOC100277080	*Zmtlc17* (TRAM/LAG/CRN8 17)	4.640957394	-	-	-3.136898104	-
100274425	LOC100274425	*Zmtlc9* (TRAM/LAG/CRN8 9)	-17.48003338	-	-	20.0287248	-

When comparing data from the AzoYuc group with the Ctr one, *Zmpin1c* (GeneID: 103654258) was up-regulated [Log2(FC) 1.8]; and *Zmpin1c* and *Zmpin1d* (GeneID: 100285745) were up-regulated when comparing AzoYuc with Yuc [(Log2(FC) 1.56 and 3.16, respectively]. 

When comparing data from the Yuc group with data from the Ctr group, *Zmarf4* (GeneID 100383226) gene was up-regulated [log2(FC) 2.85] and *Zmarf16* (GeneID 103654892), and *Zmarf19* (GeneID 100280136) genes were down-regulated [Log2(FC) -1.88 and -1.58, respectively, Tables 2 and S2]. In its turn, when comparing data from the AzoYuc group with the negative control (Ctr), *Zmarf1* and *Zmarf22* (GeneIDs 103642400 and 103629639, respectively) were up-regulated [Log2(FC) 1.55 and 1.75, respectively, Tables 2 and S2]. Comparing data from the AzoYuc group with the Yuc group, *Zmarf25* (GeneID: 100273501) was down-regulated [Log2(FC) -1.63, Tables 2 and S2]. *Zmarf7* and *Zmarf18* (GeneID: 100857063 and 100502480, respectively) were up-regulated [Log2(FC) 1.51 for both, Tables 2 and S2] when comparing data from the AzoYuc group with the Azo group. Finally, *Zmarf24* (GeneID: 103630727) was differentially expressed in two comparisons (Azo and AzoYuc groups with the Ctr group), being repressed in both [Lg2(FC) - 1.78 and -1.53, respectively, Tables 2 and S2]. 

Three Aux/IAA genes were observed being differently expressed, *Zmiaa7 (*GeneID: 100274580), *Zmiaa10/rum1* (GeneID: 100284457), and *Zmiaa27/bif1* (GeneID: 100194253). When comparing data from the AzoYuc group with Ctr, *Zmiaa7* was up-regulated [Log2(FC) 1.93, Tables 2 and S2]. In two comparisons (AzoYuc group with Yuc and Azo ones), *rum1* was up-regulated [Log2(FC) ≥ 2 in both, Tables 2 and S2]. Finally, *Zmiaa27/bif1* was up-regulated when comparing data from the Azo group with the Ctr [Log2(FC) 3.14, Tables 2 and S2], but it was down-regulated when comparing data from the AzoYuc group with the Azo group [Log2(FC) -2.22, Tables 2 and S2].

Another gene that was strongly up-regulated [Log2(FC): 4.84, Tables 2 and S2] when comparing data from the Azo group with the Ctr group was *Zmsaur41* (GeneID: 103653849); and *Zmgh3.10* (GeneID: 100193303) was up-regulated when comparing data from the AzoYuc group with Ctr, Yuc and Azo ones [Log2(FC) 2.27, 2.05, and 1.59, respectively, Tables 2 and S2].

Genes involved in the cell division control were also observed to be differentially expressed (Tables 2 and S2). When comparing transcription data from the group that was inoculated with *A. brasilense* (Azo group) with data from the control group (Ctr group)*,* we noticed that the gene *cyclin 1* (*cyc1*, GeneID: 542307), was up-regulated [Log2(FC) 2.19, Tables 2 and S2]. However, when comparing data from the AzoYuc group with data from the Azo group this gene was down-regulated [Log2(FC) -2.42, Tables 2 and S2]. When comparing data from the AzoYuc group with the Yuc and Azo, another gene, *Zmick4* (inhibitor of cyclin-dependent kinase 4, GeneID: 100193847) that codes for a protein involved in the cellular cycle regulation was differentially expressed. This gene was strongly up-regulated when comparing data from the AzoYuc group with the Yuc and Azo ones [Log2(FC) 3.16 and 2.77, respectively, Tables 2 and S2]. Another gene observed being differentially expressed was *Zmdel1c* (GeneID: 103627702). It was up-regulated when comparing data from the AzoYuc group with the Ctr, Yuc, and Azo groups [Log2(FC) 2.9, 2.77, and 3.19, respectively, Tables 2 and S2].

Genes involved in the abscisic acid (ABA) response pathway (*Zmpyl7*, *Zmpp2c3, Zmpp2c4,* and *Zmpp2c14*) were differentially regulated (Tables 2 and S2). *Zmpyl7* was up-regulated when comparing data from the Yuc group with Ctr, and from the AzoYuc group with Ctr and Azo groups [log2(FC) 2.19, 2.38, and 3.39, respectively]. In its turn, the genes that code for the PP2Cs presented an interesting expression pattern. When comparing data from the Azo group with Ctr, *Zmpp2c3, -c4,* and *-c14* were all up-regulated [log2(FC) 1.62, 2.33, and 2.11, respectively]. On the other hand, when comparing data from the AzoYuc group with Azo, those three genes became down-regulated [log2(FC) -1.73, -1.71, and -2.13, respectively].

### *Azospirillum brasilense* promoted plant growth in the presence of yucasin by partially altering the maize transcriptional profile 

Looking for an explanation of how *A. brasilense* could prevent the physiological effects of yucasin and promote plant growth in the presence of this inhibitor, we gave a close look into the set of genes that changed their expression pattern in the following comparisons: Yuc group vs Ctr, and AzoYuc group vs Yuc (Figure 4C and S2, and Table S4). The comparison between data from Yuc and Ctr groups formed the control gene set, and the comparison between data from AzoYuc and Yuc groups function as the treatment set. Since the treatment with *A. brasilense* was only present in the AzoYuc group when we compare these two sets of comparisons, the observed change in the gene expression is probably due to the presence of the bacterium. In this scenario, 130 genes were differentially expressed: 48 genes changed their expression from being down- to up-regulated, and the other 82 changed from being up- to down-regulated (Figure 4C and S2, and Table S4). Among these 130 genes, 42 were uncharacterized ones. Of these, 14 changed from being down- to up-regulated, and the remaining 28 changed from being up- to down-regulated (Table S4). Looking into the genes that have annotations, were highlighted genes involved in the ABA biosynthesis pathway, in the response to biotic/abiotic stress, plant disease resistance (R) system, and a D-type cellulose synthase.

One gene involved in the Abscisic acid (ABA) biosynthesis pathway was identified in our samples, a member of the short-chain dehydrogenase/reductase family, *Zmaba2* (aka short-chain alcohol dehydrogenase1 - sca1, GeneID: 100282128). In our data, *Zmaba2* was up-regulated [Log2(FC) 1.87, Tables 2, S2, and S4] in the presence of yucasin (Yuc group vs. Ctr), and down-regulated [Log2(FC) - 1.28, Tables 2 and S2] in the presence of the bacterium (AzoYuc group vs Yuc). Although this gene was below the established threshold for Log2(FC) in the second comparison, it presented a valid p-value (0.022, Table S2). Since it is an important gene in the ABA synthesis pathway, we decided to include it in our analysis.

Concerning the genes involved in the response to biotic/abiotic stress, genes coding for mitogen-activated protein kinase 5 *(Zmmpk5,* GeneID 541618) and mitogen-activated protein kinase kinase 5 (*Zmmkk5,* GeneID 109946070*)* were identified. The expression analysis showed that the presence of the bacterium modified their expression pattern from being up-regulated [Log2(FC) 1.95 and 3.51, respectively] to down-regulated [(Log2(FC) - 2.37 and - 2.37, respectively] (Figure S2 and Table S4). 

Two genes that code for endoplasmic reticulum transmembrane proteins belonging to TLC (TRAM/LAG/CRN8) family were affected by the treatment with yucasin and the inoculation with *A. brasilense* (*Zmtlc9* and -*17*). *Zmtlc9* (GeneID 100274425) presented remarkable expression variation in the compared conditions, being strongly repressed [Log2(FC) - 17] by yucasin (Yuc group vs Ctr) while the inoculation with *A. brasilense* completely rescued its expression [Log2(FC) 20 in AzoYuc vs Yuc comparison]. On the other hand, *Zmtlc17* (GeneID 100277080) was up-regulated in the presence of yucasin [Log2(FC) 4.64] but down-regulated when *A. brasilense* was added [Log2(FC) - 3.14].

Another gene with an opposite expression pattern was the *roothairless 6* (*rth6,* GeneID 103637736). In our experiment, *rth6* was down-regulated [Log2(FC) - 1.74] when the plantlets were treated with yucasin (Yuc group vs Ctr), while its expression was up-regulated [Log2(FC) 2.28] when comparing data from the AzoYuc group with Yuc group. Another interesting finding was revealed by comparing data from the AzoYuc group with Azo. In this comparison, this gene was also up-regulated (Log2(FC) 1.67, Tables 2, S2, and S4, and Figure S2). 

### *A. brasilense* genes expressed in maize roots 

Although rRNA was the most abundant transcript presented in our samples (Table 1), we still were able to detect three genes of *A. brasilense* being differentially expressed when comparing data from the AzoYuc group with Azo (Table S5). They are the *oxIT*, which codes for an oxalate/formate antiporter, 4-(cytidine 5’-diphosphosphate)-2-C-methyl-D-erythritol kinase, and the PrKA family serine protein kinase coding genes. All three were highly up-regulated in our analysis [Log2(FC) > 4, Table S5]. The identified genes are part of general metabolic pathways and their presence indicated that the bacteria were metabolically active.

## Discussion

### *Azospirillum brasilense* plant-growth promotion prevented yucasin physiological effect on the maize plantlets 

It is well known that bacteria belonging to the genus *Azospirillum,* specifically *A. brasilense,* can produce phytohormones and other substances during plant-bacterium interaction (Cassán et al., 2014, 2020). Among them are the auxins, especially the indole-3-acetic acid, IAA (Cassán *et al*., 2014, 2020). However, according to Cassán *et al.* (2020), there is evidence that plant growth promoted by *Azospirillum* sp can be both IAA-dependent and IAA-independent. In plants, IAA is involved in plant development (Casanova-Sáez and Voß, 2019), and as already mentioned, yucasin can inhibit its production in plants (Nishimura et al., 2014). The results observed for root and aerial lengths suggest that yucasin did not interfere in the plant-microbe interaction, because plantlets from the AzoYuc presented longer lengths than those of the Yuc group and no difference was observed when comparing plantlets from the AzoYuc group with those from the Azo one. On the other hand, the presence of yucasin inhibited the formation of lateral roots in maize plantlets (Yuc group), and previous inoculation with A. brasilense was able to prevent the yucasin effect on this plant phenotype. These results, combined with the fact that the plantlets from the AzoYuc and Yuc groups presented lower IAA concentrations than those of the Azo group but no difference among them (Figure 2), suggest that *A. brasilense* was able to prevent the phenotypic effects of yucasin using an IAA-independent plant growth promotion pathway.

### Transcriptome analysis

Eukaryotic mitogen-activated protein kinase (MAPK) cascades transduce of environmental and developmental signals in intracellular responses (Rodriguez et al., 2010). In a general model, MAPK kinase kinase (MAP3Ks; also called MAPKKKs or MEKKs) or MAPK kinase kinase kinase (MAP4Ks) are activated by stimulated plasma membrane receptors, and then, through sequential phosphorylation, activate downstream MAP kinase kinase (MAP2K, also called MKKs or MEKs), which in turn activates MAPKs. Specifically, MAP3Ks (or MAPKKKs) are serine or threonine kinases that phosphorylate MAP2Ks at a conserved S/T-X_3−5-_S/T motif, and MAP2Ks phosphorylate MAPKs on threonine and tyrosine residues at a conserved T-X-Y motif (Rodriguez *et al*., 2010; Kong et al., 2013; Sun et al., 2015). The active MAPKs interact with various effector proteins in the cytoplasm and nucleus, which include other kinases, enzymes, or transcription factors (Rodriguez *et al*., 2010; Kong *et al.*, 2013; Sun *et al.*, 2015). According to KEGG, ZmMPK5 and ZmMKK5 [whose respective genes have changed their pattern of expression due to the presence of the bacterium, from being up-regulated (Yuc vs. Ctr) to down-regulated (AzoYuc vs. Yuc)] participate in the cascade that perceives the apoplastic H_2_O_2_, leading to its higher production and cell death. They also participate in the cascade that triggers the early and late defense response against pathogens. Besides that, ZmMPK5 is also indicated as part of the defense responses mediated by ethylene and in the ethylene synthesis in response to reactive oxygen species (ROS). Ma et al. (2016) identified a member of the short-chain dehydrogenase/reductase family, *Zmaba2* (aka short-chain alcohol dehydrogenase1 - sca1, GeneID: 100282128), as a target of ZmMPK5. According to these authors and KEGG (https://www.genome.jp/kegg-bin/show_pathway?zma00906+100282128), the *Zmaba2* product participates in the ABA synthesis pathway and is responsible for the conversion of xanthoxin into an abscisic aldehyde. In our data, *Zmaba2* was up-regulated in the presence of yucasin (Yuc vs. Ctr), and down-regulated in the presence of the bacterium (AzoYuc vs Yuc). According to Ma *et al.* (2016), ZmABA2 is phosphorylated by ZmMPK5 at its S173 position, which increases ZmABA2 stability and ABA production. Since the overexpression of both genes or the overexpression of *Zmaba2* in the presence of PEG or NaCl leads to increased levels of ABA (Ma *et al.*, 2016), the up-regulation of *Zmmpk5* and *Zmaba2* probably led to increased levels of this hormone in response to the presence of yucasin. On the other hand, *Zmmpk5* and *Zmaba2* down-regulation in the presence of the bacterium (AzoYuc vs. Yuc) probably reduced these levels.

TLC genes are part of the plant disease resistance (R) system and confer resistance to pathogenic toxins. They codify endoplasmic reticula (ER)-resident transmembrane (TM) proteins that act as synthases and activate the synthesis of ceramide-like moieties and/or sphingolipids (Si et al., 2019). According to Si *et al.* (2019), sphingolipid synthesis, including ceramide synthesis, plays an important role in biotic and abiotic stress responses, triggering programmed cell death in plants. According to these authors, *Zmtlc9* and *-17* presented low expression levels in roots and were up-regulated when the plantlets were treated with the mycotoxin Fumonisin B1 or the pathogenic fungus *Curvularia lunata*. In our observations, *Zmtlc9* was strongly down-regulated in the presence of yucasin (Yuc vs. Ctr.) and strongly up-regulated when the bacterium was in the treatment (AzoYuc vs. Yuc). On the other hand, *Zmtlc17* was up-regulated in the first comparison and down-regulated in the second one (Figure S2, Tables 2 and S4). 

According to Kazan and Manners (2009), auxin participates in the plant defense response. It happens in a direct way or by interaction with other hormone signaling pathways (Bari and Jones, 2009; Kazan and Manners, 2009). Auxin production or effects are stimulated or repressed during pathogen infection (Bari and Jones, 2009; Kazan and Manners, 2009), and low levels of this hormone are needed to trigger biotrophic resistance, which may occur due to the production of compounds involved in the plant defense from tryptophan using part of the auxin synthesis pathway (Kazan and Manners, 2009). Taking all these together alongside our observations, we can suggest that the presence of yucasin was responsible for the up-regulation observed for the genes *Zmmkk5, Zmmpk5, Zmaba2,* and *Zmtlc17,* and the down-regulation of *Zmtlc19.* Since IAA concentration in the AzoYuc group was no different from the Yuc group, we can also suggest that *A. brasilense*, through an IAA-independent pathway, was responsible for the change in the expression pattern of these genes (Figure S2, Tables 2 and S4). 

According to Li et al. (2016), the gene *rth6* is involved in root hair formation. This gene encodes the plasma membrane protein CSLD5 (Cellulose Synthase-like D), a D-type cellulose synthase (Li *et al.*, 2016). According to Li *et al.* (2016), this transmembrane protein is responsible for the synthesis of cellulose in the plasma membrane which is extruded in the inner side of the cell wall in the maize root hair tips. These authors observed that maize mutants for this gene showed a reduced number of root hairs and did not express it in their roots. Based on this and this gene expression profile alongside the IAA concentrations observed in our experiment (Figure 2), we can hypothesize that yucasin caused this gene repression (Yuc vs. Ctr), and the bacterium (AzoYuc vs. Yuc), through an IAA-independent pathway, recovered its expression (Figure S2, Table 2, S2 and S4).

In this scenario, a total of 129 genes were differentially expressed: 48 genes changed their expression from being down- to up-regulated, and the other 81 changed from being up- to down-regulated (Figure S2 and Table S4 bottom). When comparing the data from AzoYuc with the data from the Yuc group we observed that the former presented a higher number of lateral roots, and longer lengths (root and aerial parts) than the second one (Figure 3). All these together with the fact that there was no difference in IAA concentration in both comparisons allow us to assume that the bacterium caused these changes in gene expression through an IAA-independent pathway. We also conclude that these gene expressions are somehow involved in the physiological effect observed when comparing the AzoYuc group with Yuc one.

## Conclusion

Gene expression analyses showed genes involved in the IAA response pathway, IAA efflux transport, and cell division control, responding to the bacterium, yucasin, the decrease in IAA concentration, or altogether. Several genes changed their expression pattern in response to bacterial inoculation. Some of them were identified as genes that code for proteins involved in the ABA biosynthesis pathway, response to biotic/abiotic stress, plant disease resistance (R) system, and D-type cellulose synthase. Our results suggest that yucasin itself was enough to trigger the expression of some genes involved in the biotic/abiotic responses, and *A. brasilense* reverted it probably through an IAA-independent pathway. The opposite change was observed for genes involved in plant disease resistance and the D-type cellulose synthase, indicating that *A. brasilense* stimulated their expression.

All these results lead us to suggest that *A. brasilense* interferes with the expression of many maize’s genes through an IAA-independent pathway. The results also showed that the bacterial plant growth effect somehow involves the repression of some genes involved in biotic/abiotic stress and stimulating/repressing genes involved in cell division regulation. Since this is an exploratory study to bring some light on the plant-bacteria relationship, more specific studies are required to better understand how *Azospirillum* interferes with these genes’ expression and which phytohormones and substances other than IAA are involved in plant growth promotion.

## Supplementary material

The following online material is available for this article:

Figure S1 - Scanning electron microscopy of maize roots inoculated with *A. brasilense* strain FP2.

Figure S2 - Genes that switched the expression pattern when analyzing two experimental comparisons (Yuc x Ctr and AzoYuc x Yuc).

Table S1 - Library features and number of total reads attributed to *Zea mays* or the combined reference.

Table S2 - Maize differentially expressed genes (DEGs) in all experimental conditions.

Table S3 - Maize uncharacterized DEGs in all experimental conditions.

Table S4 - Maize DEGs that changed their pattern of expression when comparing two experimental conditions (Yuc x Ctr and AzoYuc x Yuc).

Table S5 -
*Azospirillum brasilense* DEGs in the experimental condition evaluated.

## References

[B1] Ambrosini A, Beneduzi A, Stefanski T, Pinheiro FG, Vargas LK, Passaglia LMP (2012). Screening of plant growth promoting rhizobacteria isolated from
sunflower (Helianthus annuus L.). Plant Soil.

[B2] Babalola OO (2010). Beneficial bacteria of agricultural importance. Biotechnol Lett.

[B3] Bari R, Jones JDG (2009). Role of plant hormones in plant defense responses. Plant Mol Biol.

[B4] Bashan Y, de-Bashan LE (2010). How the plant growth-promoting bacterium Azospirillum promotes
plant growth - A critical assessment. Adv Agron.

[B5] Baudoin E, Lerner A, Mirza MS, Zemrany HE, Prigent-Combaret C, Jurkevich E, Spaepen S, Vanderleyden J, Nazaret S, Okon Y (2010). Effects of Azospirillum brasilense with genetically modified
auxin biosynthesis gene ipdC upon the diversity of the indigenous microbiota
of the wheat rhizosphere. Res Microbiol.

[B6] Bishopp A, Mahonen AP, Helariutta Y (2006). Signs of change: Hormone receptors that regulate plant
development. Development.

[B7] Casanova-Sáez R, Voß U (2019). Auxin metabolism controls developmental decisions in land
plants. Trends Plant Sci.

[B8] Cassán F, Vanderleyden J, Spaepen S (2014). Physiological and agronomical aspects of phytohormone production
by model Plant-Growth-Promoting Rhizobacteria (PGPR) belonging to the genus
Azospirillum. J Plant Growth Regul.

[B9] Cassán F, Diaz-Zorita M (2016). Azospirillum sp. in current agriculture: From the laboratory to
the field. Soil Biol Biochem.

[B10] Cassán F, Coniglio A, López G, Molina R, Nievas S, de Carlan CLN, Donadio F, Torres D, Rosas S, Pedrosa FO (2020). Everything you must know about Azospirillum and its impact on
agriculture and beyond. Biol Fert Soils.

[B11] Duca D, Lorv J, Patten CL, Rose D, Glick BR (2014). Indole-3-acetic acid in plant-microbe
interactions. A Van Leeuw J Microb.

[B12] Egener T, Hurek T, Reinhold-Hurek B (1999). Endophytic expression of nif genes of Azoarcus sp strain BH72 in
rice roots. Mol Plant Microbe In.

[B13] Espindula E, Faleiro AC, Pereira TP, Amaral FP, Arisi ACM (2017). Azospirillum brasilense FP2 modulates respiratory burst oxidase
gene expression in maize seedlings. Indian J Plant Physiol.

[B14] Espindula E, Sperb ER, Bach E, Passaglia LMP (2019). The combined analysis as the best strategy for Dual RNA-Seq
mapping. Genet Mol Biol.

[B15] Faleiro AL, Pereira TP, Espindula E, Brod FCA, Arisi ACM (2013). Real-time PCR detection targeting nifA gene of plant growth
promoting bacteria Azospirillum brasilense strain FP2 in maize
roots. Symbiosis.

[B16] Fukami J, Cerezini P, Hungria M (2018). Azospirillum: Benefits that go far beyond biological nitrogen
fixation. AMB Express.

[B17] Glickmann E, Dessaux Y (1995). A critical examination of the specificity of the Salkowski
reagent for indolic compounds produced by phytopathogenic
bacteria. Appl Environ Microb.

[B18] Gu Z, Eils R, Schlesner M (2016). Complex heatmaps reveal patterns and correlations in
multidimensional genomic data. Bioinformatics.

[B19] Haddad A, Campos APC, Sesso A (2007). Técnicas de microscopia eletrônica aplicadas às ciências
biológicas. Sociedade Brasileira de Microscopia.

[B20] Heberle H, Meirelles GV, da Silva FR, Telles GP, Minghim R (2015). InteractiVenn: A web-based tool for the analysis of sets through
Venn diagrams. BMC Bioinformatics.

[B21] Hungria M, Campo RJ, Souza EM, Pedrosa FO (2010). Inoculation with selected strains of Azospirillum brasilense and
A. lipoferum improves yields of maize and wheat in Brazil. Plant Soil.

[B22] Jahn CE, Charkowski AO, Willis DK (2008). Evaluation of isolation methods and RNA integrity for bacterial
RNA quantitation. J Microbiol Meth.

[B23] Karnovsky M (1964). A Formaldehyde-Glutaraldehyde fixative of high osmolality for use
in electron microscopy. J Cell Biol.

[B24] Kazan KM, Manners JM (2009). Linking development to defense: Auxin in plant-pathogen
interactions. Trends Plant Sci.

[B25] Khabbaz KSE, Ladhalakshmi LD, Babu BM, Kandan A, Ramamoorthy V, Saravanakumar D, Al-Mughrabi T, Kandasamy S (2019). Plant Growth Promoting Bacteria (PGPB) - A versatile tool for
plant health management. Can J Pestic Pest Manag.

[B26] Kim YJ, Oh YJ, Park WJ (2006). HPLC-based quantification of indole-3-acetic acid in the primary
root tip of maize. J Nanobiotechnol.

[B27] Kong X, Pan J, Zhang D, Jiang S, Cai G, Wang L, Li D (2013). Identification of mitogen-activated protein kinase kinase gene
family and MKK-MAPK interaction network in maize. Biochem Bioph Res Co.

[B28] Li L, Hey S, Liu S, Liu Q, McNinch C, Hu HC, Wen TJ, Marcon C, Paschold A, Bruce W (2016). Characterization of maize roothairless6 which encodes a D-type
cellulose synthase and controls the switch from bulge formation to tip
growth. Sci Rep.

[B29] Li Z, Xu M, Wei H, Wang L, Deng M (2019). RNA-seq analyses of antibiotic resistance mechanisms in Serratia
marcescens. Mol Med Rep.

[B30] Love MI, Huber W, Anders S (2014). Moderated estimation of fold change and dispersion for RNA-seq
data with DESeq2. Genome Biol.

[B31] Ma F, Ni L, Liu L, Li X, Zhang H, Zhang A, Tan M, Jiang M (2016). ZmABA2, an interacting protein of ZmMPK5, is involved in abscisic
acid biosynthesis and functions. Plant Biotechnol J.

[B32] Mashiguchi K, Tanaka K, Sakai T, Sugawara S, Kawaide H, Natsume M, Hanada A, Yaeno T, Shirasu K, Yao H (2011). The main auxin biosynthesis pathway in
Arabidopsis. P Natl Acad Sci-Biol.

[B33] Messing J, Bennetzen JL, Hake S (2009). Handbook of maize: Genetics and genomics.

[B34] Mortazavi A, Williams BA, McCue K, Schaeffer L, Wold B (2008). Mapping and quantifying mammalian transcriptomes by
RNA-Seq. Nat Methods.

[B35] Nishimura T, Hayashi K, Suzuki H, Gyohda A, Takaoka C, Sakaguchi Y, Matsumoto S, Kasahara H, Sakai T, Kato J (2014). Yucasin is a potent inhibitor of YUCCA, a key enzyme in auxin
biosynthesis. Plant J.

[B36] Pedrosa FO, Yates MG (1984). Regulation of nitrogen fixation (nif) genes of Azospirillum
brasilense by nifA and ntr (gln) type gene products. FEMS Microbiol Lett.

[B37] Rodriguez MCS, Petersen M, Mundy J (2010). Mitogen-Activated Protein Kinase Signaling in
Plants. Annu Rev Plant Biol.

[B38] Si W, Hang T, Guo M, Chen Z, Liang Q, Gu L, Ding T (2019). Whole-Genome and transposed duplication contribute to the
expansion and diversification of TLC genes in maize. Int J Mol Sci.

[B39] Spaepen S, Vanderleyden J, Remans R (2007). Indole-3-acetic acid in microbial and microorganism-plant
signaling. FEMS Microbiol Rev.

[B40] Spaepen S, Vanderleyden J (2010). Auxin and plant-microbe interactions. Cold Spring Harb Perspect Biol.

[B41] Sun W, Chen H, Wang J, Sun HW, Yang SK, Sang YL, Lu XB, Xu XH (2015). Expression analysis of genes encoding mitogen-activated protein
kinases in maize provides a key link between abiotic stress signaling and
plant reproduction. Funct Integr Genomic.

[B42] Verna JP, Yadav J, Tiwari KN, Singh L, Singh V (2010). Impact of plant growth promoting rhizobacteria on crop
production. Int J Agric Res.

[B43] Vilasboa J, Costa CT, Matsuura HN, Fett-Neto AG (2019). Rooting of cuttings of Passiflora suberosa, a medicinal passion
fruit species: Characterization and modulation by external biochemical
factors. Isr J Plant Sci.

[B44] Won C, Shen X, Mashiguchi K, Zheng Z, Dai X, Cheng Y, Kasahara H, Kamiya Y, Chory J, Zhao Y (2011). Conversion of tryptophan to indole-3-acetic acid by tryptophan
aminotransferases of Arabidopsis and YUCCAs in Arabidopsis. P Natl Acad Sci-Biol.

[B45] Yoo Y-H, Kim M, Chandran AKN, Hong WJ, Ahn HR, Lee GT, Kang S, Suh D, Kim J, Kim YJ (2019). Genome-wide transcriptome analysis of rice seedlings after seed
dressing with Paenibacillus yonginensis DCY84 T and Silicon. Int J Mol Sci.

[B46] Yue J, Hu X, Huang J (2014). Origin of plant auxin biosynthesis. Trends Plant Sci.

[B47] Zhao Y (2010). Auxin biosynthesis and its role in plant
development. Annu Rev Plant Biol.

[B48] Zhao Y (2012). Auxin biosynthesis: A simple two-step pathway converts tryptophan
to indole-3-acetic acid in plants. Mol Plant.

[B49] Zhao Y (2014). Auxin biosynthesis. Arabidopsis Book.

