# Supplementary material for: Effects on gene expression during maize-Azospirillum interaction in the presence of a plant-specific inhibitor of indole-3-acetic acid production
Source: Genet Mol Biol. 2023 Sep 18;46(3 Suppl 1):e20230100. doi: 10.1590/1678-4685-GMB-2023-0100 (PMC10510588; doi:10.1590/1678-4685-GMB-2023-0100)
Supplement: Figure S1 - [file 1415-4757-GMB-46-3-s1-e20230100-s1.pdf]

Genetics and Molecular Biology

https://doi.org/10.1590/1678-4685-GMB-2023-0100

Supplementary Material to "Effects on gene expression during maize-*Azospirillum* interaction in the presence of a plant-specific inhibitor of indole-3-acetic acid production"

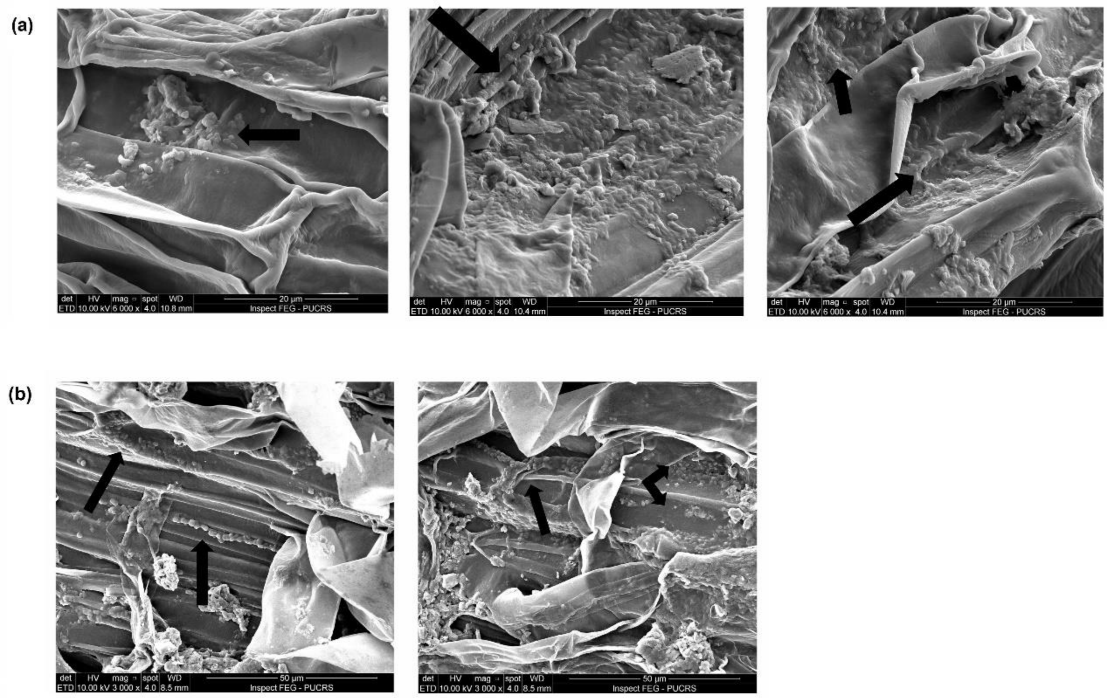

Figure S1 - Scanning electron microscopy of maize roots inoculated with *A. brasilense* strain FP2. (A) Roots from group Azo; (B and C) roots from group AzoYuc. The pictures at (C) represent the same site with different magnifications. Arrows indicates *Azospirillum brasilense* FP2 colonies. Azo = plantlets inoculated with *A. brasilense* FP2; AzoYuc = plantlets that received 50 µM of yucasin and were inoculated with *A. brasilense* FP2.
