# Supplementary material for: Effects on gene expression during maize-Azospirillum interaction in the presence of a plant-specific inhibitor of indole-3-acetic acid production
Source: Genet Mol Biol. 2023 Sep 18;46(3 Suppl 1):e20230100. doi: 10.1590/1678-4685-GMB-2023-0100 (PMC10510588; doi:10.1590/1678-4685-GMB-2023-0100)
Supplement: Figure S2 - [file 1415-4757-GMB-46-3-s1-e20230100-s2.pdf]

*Genetics and Molecular Biology*

https://doi.org/10.1590/1678-4685-GMB-2023-0100

# Supplementary Material to "Effects on gene expression during maize-*Azospirillum* interaction in the presence of a plant-specific inhibitor of indole-3-acetic acid production"

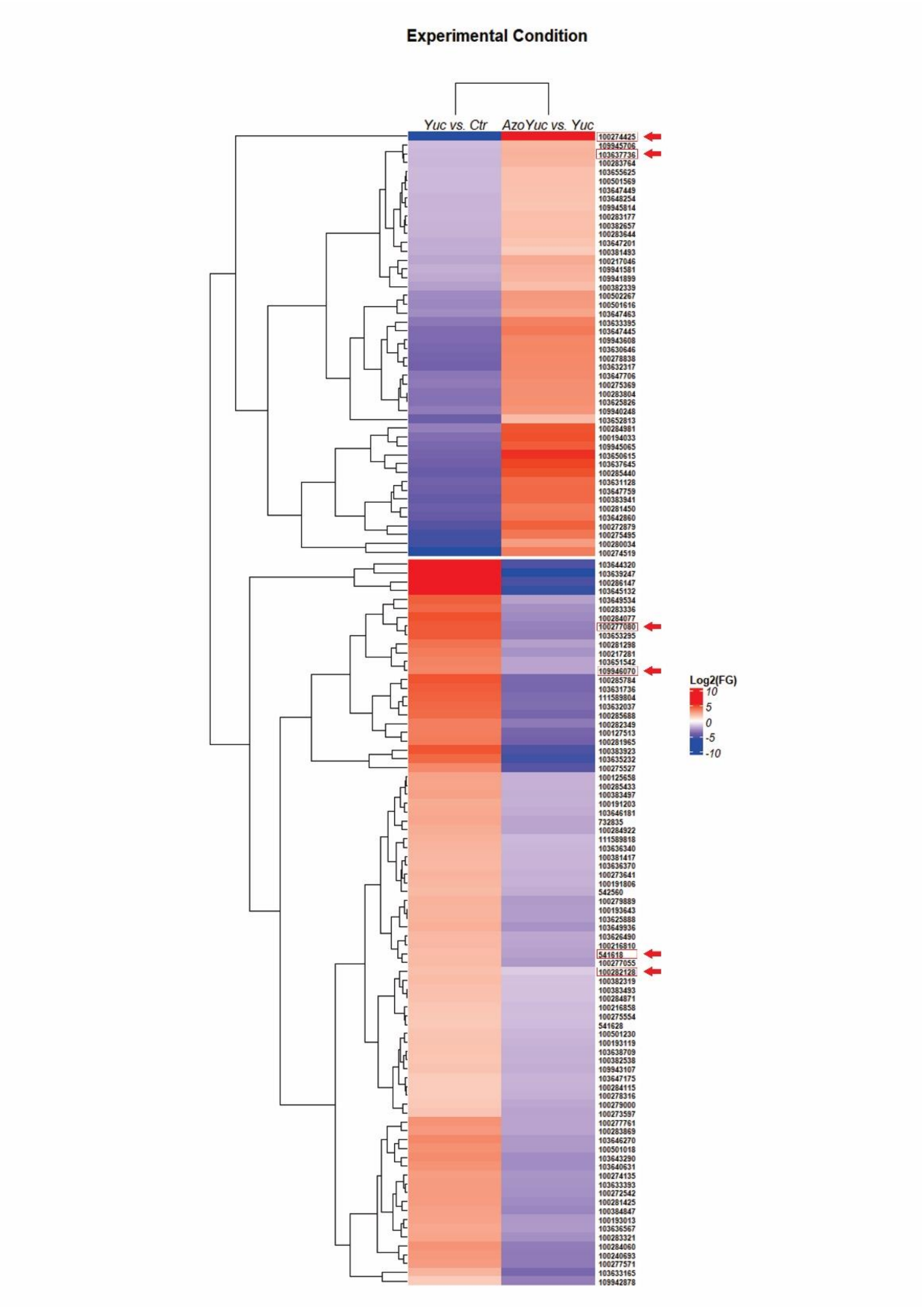

**Figure S2** - Heatmap showing the genes that switched the expression pattern when analyzing two experimental comparisons (Yuc x Ctr and AzoYuc x Yuc). Numbers refer to the GeneID of each gene and can be found in Table S4. GeneIDs inside red squares corresponded to *Zmtlc9, Zmtlc17,* Zmaba2, *Zmmkk5, Zmmpk5,* and *rth6* genes (from the top to the bottom, respectively) discussed in the text. Ctr = control plantlets; Yuc = plantlets that received 50 µM of yucasin; AzoYuc = plantlets that received 50 µM of yucasin and were inoculated with *A. brasilense* FP2.
