# Supplementary material for: Effects on gene expression during maize-Azospirillum interaction in the presence of a plant-specific inhibitor of indole-3-acetic acid production
Source: Genet Mol Biol. 2023 Sep 18;46(3 Suppl 1):e20230100. doi: 10.1590/1678-4685-GMB-2023-0100 (PMC10510588; doi:10.1590/1678-4685-GMB-2023-0100)
Supplement: Table S1 - [file 1415-4757-GMB-46-3-s1-e20230100-s3.pdf]

Genetics and Molecular Biology

https://doi.org/10.1590/1678-4685-GMB-2023-0100

Supplementary Material to "Effects on gene expression during maize-*Azospirillum* interaction in the presence of a plant-specific inhibitor of indole-3-acetic acid production"

Table S1 - Library features and number of total reads attributed to *Zea mays* or the combined reference. Ctr = control plantlets; Yuc = plantlets that received 50 µM of yucasin; Azo = plantlets inoculated with *A. brasilense* FP2; AzoYuc = plantlets that received 50 µM of yucasin and inoculated with *A. brasilense* FP2.

Experimental Group	Treatment	Total Reads per Experimental Group	Trimming (20 nt)	Reference used to Map the Reads	Reads Mapped to the reference	Percentage of Mapped Reads
Ctr	/	51,749,063	49,793,940	*Zea mays*	45,111,393	91%
Yuc	yucasin	55,278,742	53,062,047	*Zea mays*	48,782,996	92%
Azo	*A. brasilense* FP2	57,905,272	55,537,430	Combined	50,232,328	90%
AzoYuc	*A. brasilense* FP2 + yucasin	50,309,689	46,047,663	Combined	40,288,886	87%
