# Supplementary material for: Effects on gene expression during maize-Azospirillum interaction in the presence of a plant-specific inhibitor of indole-3-acetic acid production
Source: Genet Mol Biol. 2023 Sep 18;46(3 Suppl 1):e20230100. doi: 10.1590/1678-4685-GMB-2023-0100 (PMC10510588; doi:10.1590/1678-4685-GMB-2023-0100)
Supplement: Table S2 - [file 1415-4757-GMB-46-3-s1-e20230100-s4.pdf]

Genetics and Molecular Biology
https://doi.org/10.1590/1678-4685-GMB-2023-0100

Supplementary Material to "Effects on gene expression during maize-*Azospirillum* interaction in the presence of a plant-specific inhibitor of indole-3-acetic acid production"

Table S2. Maize differentially expressed genes (DEGs) in all experimental condition. = Genes that presented |Log2(FC) ≥ 1.5| and pvalue ≤ 0.05 were considered as differentially expressed. Gene description were obtained using the MaizeMine databank at http://maizemine.rnet.missouri.edu:8080/maizemine/begin.do . Ctr = control plantlets; Yuc = plantlets that received 50 µM of yucasin; Azo = plantlets inoculated with *A*.

			Yuc x Ctr		Azo x Ctr		AzoYuc x Ctr		AzoYuc x Yuc		AzoYuc x Azo	
Gene ID	Gene Symbol	Gene Description	Log2(FC)	pvalue	Log2(FC)	pvalue	Log2(FC)	pvalue	Log2(FC)	pvalue	Log2(FC)	pvalue
100217183	LOC100217183	( )-neomenthol dehydrogenase	-3,718272	0,02866	0	0	0	0	0	0	0	0
103653744	LOC103653744	( )-neomenthol dehydrogenase	0	0	0	0	1,9072764	0,0448854	2,21984566	0,019063	2,9065342	0,0026926
103638709	LOC103638709	116 kDa U5 small nuclear ribonucleoprotein component-like	1,76947209	0,020537	0	0	0	0	-1,966046	0,009106	0	0
732732	OPR4	12-oxo-phytodienoic acid reductase	0	0	0	0	2,5060286	0,0147347	2,78188836	0,006544	2,1396989	0,0328874
103629503	LOC103629503	14-3-3-like protein GF14-C	0	0	0	0	-3,978871	0,049481	0	0	-4,083055	0,040026
100285585	LOC100285585	1-acyl-sn-glycerol-3-phosphate acyltransferase 4	-3,1351417	0,031647	-3,4410734	0,0190951	0	0	0	0	0	0
100283053	LOC100283053	1-aminocyclopropane-1-carboxylate oxidase 1	0	0	0	0	1,9681887	0,0449962	0	0	0	0
100279981	acs7	1-aminocyclopropane-1-carboxylate synthase 7	0	0	0	0	1,6455121	0,0492687	0	0	2,1963215	0,0083912
103634738	LOC103634738	1-deoxy-D-xylulose 5-phosphate reductoisomerase, chloroplastic	-2,860807	0,012629	-3,0672549	0,0073162	0	0	0	0	0	0
103650571	LOC103650571	23 kDa jasmonate-induced protein	-1,6695563	0,028305	0	0	-1,680838	0,025007	0	0	0	0
103655818	LOC103655818	26S proteasome non-ATPase regulatory subunit 14 homolog	0	0	0	0	0	0	3,22135646	0,006004	2,5388	0,0223513
103640631	LOC103640631	2-aminoethanethiol dioxygenase-like	3,15684224	0,010424	0	0	0	0	-2,8590418	0,010635	0	0
100284320	LOC100284320	2-C-methyl-D-erythritol 2,4-cyclodiphosphate synthase	-2,6965556	0,040282	-2,5899403	0,0389184	0	0	0	0	0	0
103636414	LOC103636414	2-C-methyl-D-erythritol 4-phosphate cytidylyltransferase, chloroplastic	-2,6010452	0,033426	0	0	0	0	0	0	0	0
100191165	LOC100191165	2-dehydro-3-deoxyphosphooctonate aldolase	0	0	0	0	0	0	1,74389148	0,018388	1,6326706	0,0255893
103636258	LOC103636258	2-methoxy-6-polyprenyl-1,4-benzoquinol methylase, mitochondrial	0	0	0	0	0	0	-3,1599066	0,044267	0	0

			Yuc x Ctr		Azo x Ctr		AzoYuc x Ctr		AzoYuc x Yuc		AzoYuc x Azo	
Gene ID	Gene Symbol	Gene Description	Log2(FC)	pvalue	Log2(FC)	pvalue	Log2(FC)	pvalue	Log2(FC)	pvalue	Log2(FC)	pvalue
100191202	LOC100191202	2-oxoglutarate (2OG) and Fe(II)-dependent oxygenase superfamily protein	0	0	0	0	2,2594196	0,0195802	0	0	1,7549646	0,0493011
103639745	LOC103639745	2-oxoglutarate-dependent dioxygenase AOP2	2,47464192	0,004782	1,94035013	0,0281894	3,1417879	0,0002773	0	0	0	0
100273226	LOC100273226	2-oxoglutarate-dependent dioxygenase family protein	0	0	0	0	0	0	0	0	1,8518473	0,0468463
100283777	pco138567b	30S ribosomal protein S1	0	0	0	0	0	0	2,99563938	0,009499	2,2953496	0,0302804
100286104	LOC100286104	39S ribosomal protein L12	0	0	0	0	0	0	-2,7151639	0,044574	0	0
100274377	LOC100274377	3-beta hydroxysteroid dehydrogenase/isomerase family protein	0	0	3,49629325	0,0324398	0	0	0	0	0	0
100284902	LOC100284902	3-isopropylmalate dehydratase large subunit 2	0	0	0	0	-1,702864	0,0184663	0	0	0	0
103655094	LOC103655094	3-ketoacyl-CoA synthase 2	0	0	0	0	1,5470722	0,0211324	1,59830999	0,016512	0	0
103639429	LOC103639429	3-ketoacyl-CoA synthase 6-like	0	0	-3,9656668	0,0208064	0	0	0	0	0	0
100383498	csu43	3-oxoacyl-[acyl-carrier-protein] synthase II chloroplastic	0	0	-1,9014759	0,0201662	-1,963936	0,0164291	0	0	0	0
100272352	LOC100272352	3-oxo-Delta(4,5)-steroid 5-beta-reductase	0	0	0	0	0	0	-1,97091	0,024084	0	0
100281932	pco068483	40S ribosomal protein S12	0	0	0	0	0	0	0	0	1,6395143	0,039647
103629630	LOC103629630	40S ribosomal protein S14-like	0	0	0	0	-1,955965	0,0017402	0	0	0	0
103648250	LOC103648250	40S ribosomal protein S15	0	0	0	0	-1,552621	0,0091359	0	0	0	0
100280584	LOC100280584	40S ribosomal protein S15a	0	0	0	0	-1,511411	0,042074	0	0	0	0
100283242	LOC100283242	40S ribosomal protein S16	0	0	0	0	-1,742934	0,0010567	0	0	0	0
542449	rps21b	40S ribosomal protein S21	0	0	0	0	0	0	0	0	1,7455487	0,0059024
103634185	LOC103634185	40S ribosomal protein S25-3	0	0	0	0	0	0	-3,3081073	0,046642	0	0
103625888	LOC103625888	40S ribosomal protein S27	2,20727724	0,005932	0	0	0	0	-2,4768875	0,001766	0	0
103649288	LOC103649288	40S ribosomal protein S3a	0	0	2,20373629	0,0456437	0	0	0	0	0	0
103653449	LOC103653449	40S ribosomal protein S4-like	3,24540402	0,049133	0	0	3,2427697	0,0482984	0	0	3,1057832	0,0417908
100193155	LOC100193155	40S ribosomal protein S4-like protein	0	0	0	0	0	0	0	0	2,1435129	0,0412222
100191850	LOC100191850	4-coumarate--CoA ligase-like 7	-2,4501553	0,005462	-3,0109891	0,0007717	-2,823447	0,0014392	0	0	0	0
100272879	LOC100272879	4-hydroxy-tetrahydrodipicolinate reductase 2 chloroplastic	-5,0991074	0,001294	0	0	0	0	4,47773609	0,004687	0	0
111589551	LOC111589551	4-O-methyl-glucuronoyl methylesterase 1-like	-4,098523	0,037783	-4,415109	0,0245998	-4,06729	0,0347282	0	0	0	0
109942878	LOC109942878	50S ribosomal protein L2, chloroplastic	1,57254957	0,03359	0	0	0	0	-3,205083	8,71E-05	0	0
101027115	LOC101027115	50S ribosomal protein L27	0	0	0	0	-2,195449	0,0491147	0	0	0	0
100284082	LOC100284082	60S ribosomal protein L12	0	0	0	0	1,6681507	0,0219303	2,19599767	0,002628	1,6267027	0,0237167
100216970	umc2306	60S ribosomal protein L12-3	0	0	0	0	-1,742017	0,0154559	0	0	0	0

			Yuc x Ctr		Azo x Ctr		AzoYuc x Ctr		AzoYuc x Yuc		AzoYuc x Azo	
Gene ID	Gene Symbol	Gene Description	Log2(FC)	pvalue	Log2(FC)	pvalue	Log2(FC)	pvalue	Log2(FC)	pvalue	Log2(FC)	pvalue
100284115	LOC100284115	60S ribosomal protein L19-3	1,57416171	0,009084	0	0	0	0	-1,9122769	0,00133	0	0
100272919	pco134753	60S ribosomal protein L23	0	0	0	0	2,2395046	0,0187478	1,98053387	0,034205	2,2434712	0,0166375
541695	LOC541695	60S ribosomal protein L23A	0	0	0	0	0	0	-1,7469067	0,004762	0	0
100193258	LOC100193258	60S ribosomal protein L27	0	0	0	0	-2,039267	0,0126115	0	0	0	0
100283858	LOC100283858	60S ribosomal protein L27	0	0	0	0	-1,599064	0,0102306	0	0	-1,529812	0,0121736
100281939	LOC100281939	60S ribosomal protein L29	0	0	-2,2096489	0,0003735	-1,614383	0,0079515	0	0	0	0
100281996	LOC100281996	60S ribosomal protein L29	0	0	-1,9001385	0,0085341	-1,818704	0,0112629	0	0	0	0
101027258	LOC101027258	60S ribosomal protein L3	0	0	-1,5224405	0,0019325	-2,478296	8,466E-07	0	0	0	0
100381287	LOC100381287	60S ribosomal protein L32	0	0	0	0	0	0	0	0	1,8269555	0,0087693
100281802	LOC100281802	60S ribosomal protein L35	0	0	0	0	-2,501289	0,028616	-2,3335436	0,039295	-2,414724	0,0313908
100286143	csAtPR5	60S ribosomal protein L37	0	0	0	0	1,6229282	0,0216696	2,14917424	0,002433	1,7363533	0,0130551
100280657	LOC100280657	60S ribosomal protein L4	0	0	0	0	-1,68612	3,531E-05	0	0	0	0
103641720	LOC103641720	60S ribosomal protein L6-3	0	0	0	0	-4,02396	0,0486546	0	0	0	0
103651339	LOC103651339	65-kDa microtubule-associated protein 3	-2,2539446	0,025169	-2,5430376	0,0118912	-3,113794	0,0028094	0	0	0	0
103642932	LOC103642932	65-kDa microtubule-associated protein 8	0	0	0	0	0	0	0	0	1,8720582	0,0486601
103654243	LOC103654243	7-deoxyloganetin glucosyltransferase	0	0	0	0	0	0	3,46155559	0,011903	2,9989495	0,0198103
100192754	LOC100192754	7-hydroxymethyl chlorophyll a reductase chloroplastic	0	0	0	0	3,3411967	0,0346862	0	0	0	0
100191565	cl1004_1	8-amino-7-oxononanoate synthase	0	0	0	0	0	0	-2,8559866	0,037143	0	0
103640324	LOC103640324	9-beta-pimara-7,15-diene oxidase	0	0	-3,928292	0,0301466	-5,690083	0,0028474	0	0	0	0
100191940	LOC100191940	AAA-ATPase ASD mitochondrial	0	0	0	0	0	0	-1,6117924	0,026935	0	0
103648227	LOC103648227	AAA-ATPase ASD mitochondrial	0	0	0	0	0	0	0	0	3,5846713	0,0361098
103626091	LOC103626091	AAA-ATPase At2g46620	0	0	0	0	-1,969674	0,0204041	0	0	0	0
103643314	LOC103643314	ABC transporter A family member 2	0	0	0	0	1,5626573	0,0396726	0	0	0	0
103629799	LOC103629799	ABC transporter A family member 7	2,76488586	0,015475	0	0	0	0	0	0	0	0
103653413	LOC103653413	ABC transporter A family member 7	0	0	0	0	2,8268094	0,0380613	0	0	0	0
103641772	LOC103641772	ABC transporter B family member 15	0	0	-2,1292464	0,0009058	0	0	0	0	1,8729176	0,0032483
100191944	LOC100191944	ABC transporter B family member 2	0	0	0	0	2,2832282	0,0381978	2,23397667	0,034486	2,9931992	0,0069286
103632317	LOC103632317	ABC transporter B family member 28	-4,0568023	0,000486	-1,8885372	0,0479276	0	0	3,43783285	0,003101	0	0
109941983	LOC109941983	ABC transporter C family member 3	0	0	0	0	0	0	1,50829842	0,033419	1,6982542	0,0166282
103640881	LOC103640881	ABC transporter F family member 1	1,89337876	0,022527	0	0	0	0	0	0	0	0
100192636	pco063423	ABC transporter F family member 3	-1,6219808	0,017778	0	0	-1,53092	0,0242503	0	0	0	0
103632890	LOC103632890	ABC transporter G family member 11	0	0	0	0	0	0	-1,7940617	0,045529	-1,78432	0,0449323
100278788	LOC100278788	ABC transporter G family member 16	0	0	0	0	2,3995184	0,0020178	2,14670848	0,004713	2,0383325	0,0068634
100501607	LOC100501607	ABC transporter G family member 16	0	0	0	0	0	0	3,09220582	0,017879	2,7377084	0,0269472
103643524	LOC103643524	ABC transporter G family member 25	-3,5649838	0,000187	-3,2621129	0,0005509	-2,404664	0,009907	0	0	0	0
103636060	LOC103636060	ABC transporter G family member 36	0	0	0	0	3,3230683	0,0368653	0	0	0	0
103632597	LOC103632597	ABC transporter G family member 53	1,76465053	0,027699	0	0	0	0	0	0	0	0
100273146	gpm405	ABC transporter I family member 6 chloroplastic	0	0	0	0	2,209432	0,0384257	0	0	0	0

			Yuc x Ctr		Azo x Ctr		AzoYuc x Ctr		AzoYuc x Yuc		AzoYuc x Azo	
Gene ID	Gene Symbol	Gene Description	Log2(FC)	pvalue	Log2(FC)	pvalue	Log2(FC)	pvalue	Log2(FC)	pvalue	Log2(FC)	pvalue
100285575	LOC100285575	ABC-type transport system involved in resistance to organic solvents, periplasmic component	0	0	0	0	3,9381291	0,0180134	0	0	0	0
103644296	LOC103644296	abscisic acid 8'-hydroxylase 3	0	0	0	0	0	0	3,11266228	0,013185	0	0
100191697	IDP1977	abscisic acid 8'-hydroxylase4	-1,7486014	0,024433	-1,5060788	0,0458279	0	0	0	0	0	0
103627266	LOC103627266	abscisic stress-ripening protein 3	0	0	0	0	0	0	2,69732931	0,018975	3,1353843	0,007345
100281089	IDP708	Acetate/butyrate--CoA ligase AAE7 peroxisomal	0	0	0	0	2,6130415	6,299E-05	0	0	2,1177806	0,0008014
606473	ache1	acetylcholinesterase 1	-3,3604482	0,012075	0	0	0	0	0	0	0	0
100280755	LOC100280755	acid phosphatase	0	0	0	0	2,9013944	0,0367728	0	0	0	0
100280563	LOC100280563	acid phosphatase/vanadium-dependent haloperoxidase related	0	0	0	0	3,068394	0,0010992	3,16529067	0,000645	2,6167593	0,0039122
100285275	LOC100285275	acid phosphatase/vanadium-dependent haloperoxidase related	-4,5985492	0,006759	-3,1551614	0,0296509	0	0	0	0	0	0
100272870	LOC100272870	Acidic endochitinase	0	0	2,36957394	0,0006584	0	0	0	0	-1,790431	0,0064974
103650978	LOC103650978	acidic endochitinase	0	0	0	0	2,4068626	0,0189679	0	0	0	0
542340	arpp2b	acidic ribosomal protein P2b (rpp2b)	0	0	0	0	0	0	2,13882811	0,009104	1,7677636	0,0287065
100278607	LOC100278607	ACT domain-containing protein ACR4	0	0	0	0	-2,731514	0,0342665	0	0	-2,916075	0,0205313
100274521	LOC100274521	ACT domain-containing protein ACR9	0	0	0	0	0	0	-1,8619148	0,042835	0	0
103644169	LOC103644169	actin	0	0	0	0	0	0	-3,5136431	0,044616	-3,540354	0,0420351
100281223	LOC100281223	actin associated protein	0	0	0	0	0	0	2,75965297	0,03464	0	0
100193298	LOC100193298	Actin cross-linking protein	0	0	0	0	0	0	3,90448111	0,044851	0	0
103644347	LOC103644347	actin cytoskeleton-regulatory complex protein PAN1	0	0	0	0	0	0	0	0	2,9997588	0,0413177
109942829	LOC109942829	actin cytoskeleton-regulatory complex protein PAN1-like	0	0	0	0	0	0	2,3940403	0,033523	0	0
103636793	LOC103636793	actin-7	-3,1690079	0,006282	-2,9734052	0,0088688	-3,021513	0,0078141	0	0	0	0
109943939	LOC109943939	actin-85C-like	-5,1746508	0,010576	0	0	-5,430931	0,0072961	0	0	0	0
100285352	cl5157_2	Actin-depolymerizing factor	0	0	0	0	0	0	4,42662883	0,014885	3,6907747	0,0423195
100281262	LOC100281262	actin-like protein 3	0	0	0	0	2,849975	0,0008203	0	0	0	0
103633595	LOC103633595	actin-related protein 2	0	0	0	0	3,683984	0,0033309	3,46046323	0,004493	3,281075	0,006358
100285418	LOC100285418	activator of 90 kDa heat shock protein ATPase	0	0	0	0	4,4993202	0,0056086	3,30599933	0,016766	3,8586833	0,0064119
100283105	LOC100283105	acyl carrier protein	0	0	0	0	2,2074771	0,0073084	1,91035607	0,017881	1,8555684	0,0208068
100282353	pco080082	Acyl carrier protein	0	0	0	0	0	0	-1,8742331	0,014574	0	0
103642829	LOC103642829	acyl-coenzyme A thioesterase 13	0	0	0	0	3,0440841	0,0304855	2,65495586	0,047584	3,0314891	0,0249212
100192559	LOC100192559	acyl-protein thioesterase 2	0	0	0	0	-3,201418	0,0023192	0	0	0	0
103650176	LOC103650176	Acyltransferase-like protein chloroplastic	-2,2802466	0,003621	-1,9021246	0,0112189	-1,503736	0,0399045	0	0	0	0

			Yuc x Ctr		Azo x Ctr		AzoYuc x Ctr		AzoYuc x Yuc		AzoYuc x Azo	
Gene ID	Gene Symbol	Gene Description	Log2(FC)	pvalue	Log2(FC)	pvalue	Log2(FC)	pvalue	Log2(FC)	pvalue	Log2(FC)	pvalue
100383277	LOC100383277	Adagio protein 3	0	0	0	0	1,7992774	0,0093842	0	0	0	0
103647967	LOC103647967	adenine/guanine permease AZG2	0	0	-1,6447042	0,033971	0	0	0	0	0	0
100194033	LOC100194033	adenosine/AMP deaminase family protein	-3,6224522	0,026131	0	0	0	0	4,86819868	0,002065	2,459076	0,0394358
103641022	LOC103641022	adenylylsulfatase HINT3	1,72402849	0,031162	0	0	2,2524101	0,0039583	0	0	0	0
100280524	LOC100280524	adhesive/proline-rich protein	-3,0650409	0,013371	-2,4034245	0,0380632	-3,545419	0,0051551	0	0	0	0
542737	AGP2	ADP-glucose pyrophosphorylase	-2,4431323	0,017931	-2,0107982	0,0381905	0	0	0	0	0	0
100282739	LOC100282739	ADP-ribosylation factor	0	0	0	0	1,527682	0,0310384	1,56263885	0,02521	1,59442	0,0217009
100191770	umc2200	ADP-ribosylation factor C1	2,10983175	0,016504	0	0	2,4501946	0,0052331	0	0	1,998652	0,0194516
103632959	LOC103632959	ADP-ribosylation factor GTPase-activating protein AGD3	-2,601908	0,004318	-2,4849289	0,0059385	-2,746315	0,0025034	0	0	0	0
100285433	LOC100285433	AER	2,69266587	0,000257	0	0	0	0	-1,9464923	0,007156	0	0
100286366	LOC100286366	AGG2	0	0	0	0	-2,102495	0,0389932	0	0	0	0
100281220	LOC100281220	agmatine coumaroyltransferase	0	0	0	0	0	0	3,63460495	0,003238	0	0
100286361	LOC100286361	AIR12	0	0	-5,4850108	0,0139189	0	0	0	0	0	0
103652550	LOC103652550	alanine--glyoxylate aminotransferase 2 homolog 3, mitochondrial	-3,8852669	0,031482	0	0	0	0	0	0	0	0
100273027	umc1204	Alba DNA/RNA-binding protein	0	0	-1,9130494	0,0216284	0	0	0	0	0	0
103638011	LOC103638011	ALBINO3-like protein 2, chloroplastic	0	0	-1,7224546	0,0413623	0	0	0	0	0	0
542567	aldh2	aldehyde dehydrogenase 2	0	0	0	0	0	0	-1,612405	0,012989	0	0
100192890	LOC100192890	Aldolase superfamily protein	-3,108486	0,010384	-2,4515188	0,0416932	0	0	0	0	0	0
100857059	AR5	aldose reductase	-4,3166848	0,015483	0	0	-3,61117	0,0428459	0	0	0	0
606463	AGA1	alkaline alpha galactosidase 1	0	0	2,42308656	2,362E-05	0	0	0	0	-2,05533	0,0003065
103631694	LOC103631694	alkane hydroxylase MAH1	-2,7635995	0,039626	0	0	0	0	0	0	0	0
100274212	LOC100274212	Allantoinase	1,52046242	0,012872	0	0	0	0	0	0	0	0
542151	aoc1	allene oxide cyclase 1	0	0	0	0	0	0	0	0	1,5325078	0,0491905
109945688	LOC109945688	alpha carbonic anhydrase 1, chloroplastic	0	0	0	0	0	0	0	0	1,6129191	0,0145723
109945708	LOC109945708	alpha carbonic anhydrase 8-like	0	0	0	0	2,3166355	0,0300119	3,29093829	0,003887	2,66371	0,0102314
100191604	LOC100191604	alpha/beta-Hydrolases superfamily protein	0	0	1,57440306	0,0260731	0	0	0	0	0	0
100272277	LOC100272277	alpha/beta-Hydrolases superfamily protein	0	0	0	0	0	0	0	0	2,2085588	0,0488026
103629745	LOC103629745	alpha/beta-Hydrolases superfamily protein	0	0	0	0	0	0	1,83091064	0,025444	1,580533	0,0497968
100273029	LOC100273029	Alpha-amylase 1	0	0	0	0	0	0	3,46483134	0,019586	3,1275544	0,0263891
100502506	LOC100502506	Aluminum-activated malate transporter 9	0	0	0	0	0	0	-2,2109317	0,047527	0	0
100271891	am1	ameiotic 1	0	0	0	0	0	0	0	0	3,8254517	0,0241724
100282769	LOC100282769	amelogenin precursor like protein	0	0	3,74602926	0,0140545	3,6778759	0,0159136	0	0	0	0

			Yuc x Ctr		Azo x Ctr		AzoYuc x Ctr		AzoYuc x Yuc		AzoYuc x Azo	
Gene ID	Gene Symbol	Gene Description	Log2(FC)	pvalue	Log2(FC)	pvalue	Log2(FC)	pvalue	Log2(FC)	pvalue	Log2(FC)	pvalue
100282656	LOC100282656	amino acid carrier	0	0	0	0	0	0	0	0	-1,624182	0,0492503
100272489	LOC100272489	Amino acid permease 2	0	0	-4,3242243	0,0089076	0	0	0	0	3,677491	0,0259822
100274246	LOC100274246	Amino acid permease 6	0	0	0	0	0	0	2,97132079	0,038307	0	0
100383679	LOC100383679	aminopeptidase	2,74478719	0,017857	0	0	2,2890816	0,0491932	0	0	0	0
103643317	LOC103643317	aminopeptidase M1-B	0	0	0	0	1,5310642	0,0066279	0	0	0	0
103634048	LOC103634048	aminopeptidase M1-C	-1,6498712	0,00417	0	0	0	0	0	0	0	0
100281193	pco078009(446)	AMP binding protein	0	0	0	0	2,4991168	0,005712	0	0	0	0
100304372	LOC100304372	Anaphase-promoting complex subunit 4	0	0	-2,0980901	0,032428	0	0	0	0	0	0
109940912	LOC109940912	ankyrin repeat domain-containing protein 13C	0	0	0	0	0	0	2,01024486	0,038316	0	0
100272888	LOC100272888	ankyrin repeat domain-containing protein 2	-3,4953102	0,008086	0	0	-2,988594	0,0183771	0	0	0	0
100275271	LOC100275271	Ankyrin repeat protein SKIP35	0	0	2,40574964	0,0096052	0	0	0	0	0	0
100272874	LOC100272874	ankyrin-3	0	0	-1,7115136	0,0279598	0	0	0	0	0	0
542253	an1	anther ear1	0	0	0	0	0	0	0	0	1,5618472	0,0467094
100273028	LOC100273028	anther-specific proline-rich protein APG	0	0	0	0	2,5282137	0,0143515	2,43899185	0,016004	2,5706904	0,0109618
100284218	LOC100284218	anther-specific proline-rich protein APG	0	0	-1,6099842	0,029804	0	0	0	0	0	0
103631128	LOC103631128	anthocyanidin 3-O-glucoside 6"-O-acyltransferase	-4,1119892	0,011419	-2,8368513	0,0413247	0	0	4,20965141	0,008779	2,9345255	0,031945
109939492	LOC109939492	antifreeze protein Maxi	0	0	0	0	4,0906095	0,0410139	0	0	0	0
109944961	LOC109944961	AP2/ERF and B3 domain-containing protein Os01g0141000	0	0	0	0	0	0	0	0	4,7372155	0,0284243
100274398	LOC100274398	AP2-EREBP transcription factor	2,11569915	0,017216	0	0	0	0	0	0	0	0
103643388	LOC103643388	apomucin	-5,1518749	0,009592	0	0	0	0	0	0	-4,104797	0,0276338
100282326	LOC100282326	APx3 - Peroxisomal Ascorbate Peroxidase	3,32110341	0,028877	0	0	0	0	0	0	0	0
100273441	LOC100273441	arginyl-tRNA synthetase	0	0	0	0	1,6666195	0,0152883	0	0	0	0
100384259	LOC100384259	argonaute108	0	0	1,8646824	0,0244423	0	0	0	0	-2,801919	0,0007631
100278719	LOC100278719	ARM repeat superfamily protein	0	0	0	0	0	0	2,24692533	0,038436	0	0
103646122	LOC103646122	ARM repeat superfamily protein	0	0	0	0	0	0	1,81791767	0,045364	0	0
103648171	LOC103648171	armadillo repeat-containing protein 8-like	0	0	0	0	0	0	-1,8620579	0,016775	0	0
103626266	LOC103626266	armadillo/beta-catenin-like repeat family protein	0	0	0	0	3,3218023	0,0080835	0	0	0	0
100280967	LOC100280967	ARR1 protein-like	0	0	0	0	0	0	0	0	2,9544055	0,0413222
100282996	LOC100282996	ASC1-like protein 1	0	0	0	0	6,1992355	0,0051139	0	0	4,6727796	0,0201788
100191676	pco061977	Ascorbate transporter chloroplastic	0	0	3,43784731	0,0115064	0	0	0	0	-2,85809	0,0209834
100192351	asn4	asparagine synthetase 4	-2,6405583	0,024021	0	0	0	0	0	0	0	0

			Yuc x Ctr		Azo x Ctr		AzoYuc x Ctr		AzoYuc x Yuc		AzoYuc x Azo	
Gene ID	Gene Symbol	Gene Description	Log2(FC)	pvalue	Log2(FC)	pvalue	Log2(FC)	pvalue	Log2(FC)	pvalue	Log2(FC)	pvalue
542708	akh2	aspartate kinase homoserine dehydrogenase 2	0	0	0	0	-1,664523	0,0436016	0	0	-1,904263	0,0193661
100274575	LOC100274575	Aspartic proteinase nepenthesin-1	0	0	0	0	0	0	4,33829924	0,008151	2,7345763	0,0499382
100382179	LOC100382179	Aspartyl protease AED1	0	0	-1,8146493	0,041612	0	0	0	0	0	0
109940156	LOC109940156	aspartyl protease family protein 1	0	0	0	0	0	0	0	0	-1,593627	0,0423656
100127507	LOC100127507	Asr protein	0	0	0	0	0	0	0	0	2,4665561	0,0045482
103640067	LOC103640067	atherin-like	-2,9036232	0,030055	0	0	0	0	0	0	0	0
103650767	LOC103650767	atherin-like	0	0	-3,8725262	0,0169469	0	0	0	0	0	0
103652056	LOC103652056	atherin-like	-1,5336945	0,040929	0	0	0	0	0	0	0	0
109941470	LOC109941470	atherin-like	0	0	0	0	2,7992145	0,0152629	0	0	2,3499792	0,0335875
109945065	LOC109945065	atherin-like	-3,8134781	0,000339	-1,9946715	0,0261693	0	0	4,5748587	1,36E-05	2,7560578	0,001782
109945789	LOC109945789	atherin-like	0	0	0	0	5,8661718	0,001431	4,58150561	0,004449	4,6711035	0,0034154
100281356	LOC100281356	ATP binding protein	-2,4541072	3,49E-05	0	0	-1,62349	0,0026537	0	0	0	0
100281776	LOC100281776	ATP binding protein	3,07401165	0,049345	0	0	0	0	0	0	0	0
100285534	LOC100285534	ATP binding protein	1,94018084	0,025129	0	0	0	0	0	0	0	0
100285538	LOC100285538	ATP binding protein	0	0	0	0	4,8762264	0,0021278	3,9906466	0,009209	5,6023373	0,0004438
100285713	LOC100285713	ATP binding protein	1,59653777	0,019645	0	0	0	0	0	0	0	0
100302578	LOC100302578	ATP binding protein	1,52071534	0,001594	0	0	0	0	0	0	0	0
100283477	LOC100283477	ATP/GTP binding protein	0	0	-2,0870019	0,028122	0	0	0	0	0	0
103641793	LOC103641793	ATP/GTP binding protein-like	0	0	0	0	0	0	0	0	1,9481885	0,0395946
103627618	LOC103627618	ATPase family AAA domain-containing protein 3C	0	0	-2,3186456	0,0081602	-1,631869	0,0470476	0	0	0	0
103653966	LOC103653966	ATPase family AAA domain-containing protein 3C	0	0	-2,6697726	0,0412012	0	0	0	0	0	0
100285138	LOC100285138	ATPase, coupled to transmembrane movement of substances	1,6204775	0,02087	0	0	0	0	0	0	0	0
100286184	LOC100286184	ATPase, coupled to transmembrane movement of substances	0	0	0	0	-2,959764	0,0115116	0	0	0	0
100273342	pco133717	ATP-dependent Clp protease proteolytic subunit	0	0	0	0	2,4818205	0,0251672	3,63771493	0,002066	2,602144	0,0154968
100193513	LOC100193513	ATP-dependent protease La (LON) domain protein	0	0	0	0	0	0	0	0	-2,22552	0,0132408
100286176	LOC100286176	ATP-dependent RNA helicase DDX23	0	0	0	0	0	0	1,71019953	0,022457	0	0
100283799	LOC100283799	ATP-dependent RNA helicase dhh1	0	0	0	0	0	0	0	0	1,9431374	0,0201346
100281385	LOC100281385	ATP-dependent RNA helicase DHX8	0	0	0	0	0	0	-3,3414969	0,000364	0	0
100280924	LOC100280924	ATP-dependent rRNA helicase spb4	3,23373253	0,025113	3,54328217	0,0130796	4,033914	0,004389	0	0	0	0
100282925	LOC100282925	ATP-dependent transporter YFL028C	0	0	0	0	2,7626361	0,0030626	2,62181794	0,004109	2,643811	0,0036338
103635431	LOC103635431	ATP-dependent zinc metalloprotease FTSH 5, mitochondrial	0	0	0	0	2,3296175	0,0036509	2,17260998	0,005256	1,7113946	0,0244839
100283810	LOC100283810	ATPP2-B12	0	0	0	0	-1,608592	0,0310828	0	0	0	0

			Yuc x Ctr		Azo x Ctr		AzoYuc x Ctr		AzoYuc x Yuc		AzoYuc x Azo	
Gene ID	Gene Symbol	Gene Description	Log2(FC)	pvalue	Log2(FC)	pvalue	Log2(FC)	pvalue	Log2(FC)	pvalue	Log2(FC)	pvalue
100285260	LOC100285260	atypical receptor-like kinase MARK	0	0	0	0	1,9169709	0,0219197	2,70488284	0,001764	1,9806924	0,0136959
100281926	LOC100281926	augmenter of liver regeneration	-2,2700669	0,027914	-2,5323112	0,0144522	0	0	0	0	0	0
542639	cl30708_1	AUGMIN subunit 7	0	0	0	0	2,999089	0,0056405	0	0	0	0
100240693	Atg12	autophagy-related 12	3,03282468	0,014489	0	0	0	0	-3,2899375	0,005948	0	0
100279947	TIDP2822	Autophagy-related protein 13a	-2,6491647	0,004354	-2,1955684	0,0128396	-2,10964	0,0159521	0	0	0	0
542232	abp1	auxin binding protein 1	0	0	0	0	2,1437086	0,0216815	0	0	0	0
100286190	LOC100286190	auxin-independent growth promoter-like protein	-2,6232236	0,003591	-2,8243981	0,0017115	-2,776401	0,0019416	0	0	0	0
103650873	LOC103650873	B3 domain-containing protein Os01g0905400	-3,6037844	0,033712	0	0	0	0	0	0	0	0
103642409	LOC103642409	B3 domain-containing protein Os06g0107800	0	0	-3,7122355	0,0327843	0	0	0	0	0	0
109944078	LOC109944078	B3 domain-containing protein Os07g0563300	0	0	0	0	0	0	0	0	1,9228673	0,0083855
103643100	LOC103643100	BAG family molecular chaperone regulator 1	0	0	-4,9383011	0,0111496	0	0	0	0	4,4616653	0,0214371
103626967	LOC103626967	BAG family molecular chaperone regulator 6	-2,4517611	0,023502	-2,3637973	0,0271843	0	0	0	0	0	0
103627395	LOC103627395	BAG-associated GRAM protein 1	0	0	0	0	0	0	0	0	2,610269	0,0163429
542062	mlo4	barley mlo defense gene homolog 4	0	0	0	0	0	0	-2,0301126	0,042147	0	0
542673	mlo7	barley mlo defense gene homolog 7	0	0	-2,2747511	0,020332	0	0	0	0	0	0
103652814	LOC103652814	barwin	-2,2176778	0,024823	-2,8058789	0,0050082	-3,069777	0,0023005	0	0	0	0
103632119	LOC103632119	basic helix-loop-helix protein A	0	0	0	0	0	0	4,4236987	0,018213	0	0
100275351	gpm349	Basic leucine zipper 25	0	0	0	0	2,0833062	0,0043133	0	0	0	0
103631589	LOC103631589	basic proline-rich protein-like	0	0	0	0	0	0	0	0	5,6784207	0,0085085
103642388	LOC103642388	basic proline-rich protein-like	0	0	-3,6160203	0,0405306	0	0	0	0	0	0
109944249	LOC109944249	basic proline-rich protein-like	0	0	0	0	2,6384383	0,007315	3,12278322	0,001574	2,6877352	0,0048351
103630179	LOC103630179	B-cell receptor-associated 31-like	0	0	0	0	2,0255514	0,0368463	0	0	0	0
103636340	LOC103636340	beclin-1-like protein	2,21608073	0,015357	0	0	0	0	-1,7985376	0,031259	0	0
100284904	LOC100284904	beta-amylase	0	0	0	0	3,6268173	0,0256328	0	0	0	0
732825	HYD3	beta-carotene hydroxylase	0	0	0	0	2,5908854	0,0165839	0	0	0	0
732807	glu2	beta-D-glucosidase precursor	3,52015895	0,047762	0	0	0	0	0	0	0	0
100279996	LOC100279996	Beta-D-xylosidase 4	0	0	0	0	2,4223433	0,0016849	1,6443923	0,030195	1,6767994	0,0268978
100281892	LOC100281892	beta-expansin 4	0	0	-3,1489757	0,0032634	-1,945707	0,0346625	0	0	0	0
100279283	LOC100279283	beta-fructofuranosidase, insoluble isoenzyme 7	0	0	0	0	2,7321281	0,007542	2,52197982	0,012364	2,5588925	0,0109366
100272792	LOC100272792	beta-glucosidase	-3,8035421	0,026835	0	0	-4,059822	0,0181236	0	0	0	0
103641105	LOC103641105	beta-sesquiphellandrene synthase	0	0	0	0	0	0	3,92834756	0,031761	0	0
100383821	LOC100383821	Bifunctional riboflavin biosynthesis protein RIBA 1 chloroplastic	0	0	0	0	0	0	0	0	-3,678291	0,0186566
100303815	LOC100303815	bile acid sodium symporter	0	0	4,34515758	0,0007124	0	0	0	0	-3,544473	0,0018701

			Yuc x Ctr		Azo x Ctr		AzoYuc x Ctr		AzoYuc x Yuc		AzoYuc x Azo	
Gene ID	Gene Symbol	Gene Description	Log2(FC)	pvalue	Log2(FC)	pvalue	Log2(FC)	pvalue	Log2(FC)	pvalue	Log2(FC)	pvalue
100501347	LOC100501347	binding	0	0	0	0	3,1457149	0,0014368	2,77822076	0,003889	3,5758331	0,0002643
100277719	LOC100277719	Binding protein	0	0	0	0	0	0	-1,817368	0,028571	0	0
541663	phot1	blue-light receptor phototropin 1	0	0	0	0	-2,56609	0,0051241	-2,0671481	0,024617	-2,921425	0,0010362
100381433	LOC100381433	Boron transporter 1	0	0	0	0	0	0	0	0	1,9721426	0,0018603
100282389	LOC100282389	BRCA1-associated protein	0	0	0	0	0	0	1,75564725	0,043971	0	0
100282857	pco095316b	Brix domain-containing protein 1	0	0	0	0	0	0	0	0	-1,891109	0,0439634
100502312	LOC100502312	bromodomain 4	0	0	0	0	3,1216778	0,0446546	0	0	0	0
100382319	LOC100382319	BSD domain-containing protein	2,00798454	0,002921	0	0	0	0	-1,5074022	0,018994	0	0
100383106	LOC100383106	BSD domain-containing protein	0	0	-1,7216872	0,0414575	0	0	0	0	0	0
103655387	LOC103655387	BTB/POZ and TAZ domain-containing protein 3-like	0	0	0	0	0	0	4,60670506	0,030379	0	0
103627405	LOC103627405	BTB/POZ domain-containing protein At2g24240	0	0	0	0	3,4415227	0,0118299	0	0	0	0
103650693	LOC103650693	BTB/POZ domain-containing protein FBL11	0	0	0	0	0	0	0	0	2,0779924	0,0389036
100274431	LOC100274431	BTB/POZ domain-containing protein POB1	-2,107063	0,001727	-1,5726575	0,0152145	-2,75477	6,695E-05	0	0	0	0
100284582	LOC100284582	bZIP transcription factor ABI5	0	0	0	0	0	0	0	0	-1,765376	0,0301635
103643147	LOC103643147	bZIP transcription factor TRAB1	0	0	0	0	-1,780563	0,0130555	0	0	-2,363284	0,0006162
100281450	LOC100281450	Bzip45 (bZIP-transcription factor 45)	-4,2823386	0,009955	0	0	0	0	3,80443796	0,021264	0	0
103626820	LOC103626820	C2 and GRAM domain-containing protein	0	0	0	0	0	0	2,41198435	0,034468	0	0
100216665	LOC100216665	C2 calcium/lipid-binding plant phosphoribosyltransferase family protein	0	0	0	0	-2,177539	0,0317576	0	0	0	0
103631978	LOC103631978	C2 calcium/lipid-binding plant phosphoribosyltransferase family protein	0	0	2,24254026	0,0240053	0	0	0	0	0	0
103647671	LOC103647671	C2 calcium/lipid-binding plant phosphoribosyltransferase family protein	0	0	0	0	0	0	1,57593201	0,010167	0	0
100192718	pco087404(581)	C2 domain-containing protein	0	0	0	0	1,5149851	0,0118956	1,90239888	0,001572	1,6668351	0,0050518
100502466	LOC100502466	C2H2-like zinc finger protein	-4,4858758	0,00169	-2,434046	0,0247384	-3,386786	0,0039601	0	0	0	0
103638397	LOC103638397	cactin	0	0	0	0	0	0	-2,6073043	0,020392	0	0
100285616	LOC100285616	calcineurin B-like protein 4	0	0	0	0	0	0	0	0	1,6777972	0,038117
103641319	LOC103641319	calcium/calmodulin-regulated receptor-like kinase 1	0	0	0	0	1,8102135	0,040024	0	0	1,9480234	0,0248802
100273434	cl34301_1c	Calcium-binding EF hand family protein	0	0	0	0	1,7699759	0,0455533	0	0	0	0
103625979	LOC103625979	calcium-dependent protein kinase 9	0	0	0	0	0	0	1,91664402	0,009622	0	0

			Yuc x Ctr		Azo x Ctr		AzoYuc x Ctr		AzoYuc x Yuc		AzoYuc x Azo	
Gene ID	Gene Symbol	Gene Description	Log2(FC)	pvalue	Log2(FC)	pvalue	Log2(FC)	pvalue	Log2(FC)	pvalue	Log2(FC)	pvalue
100285166	LOC100285166	calcium-dependent protein kinase, isoform AK1	0	0	0	0	-1,814661	0,0290405	-3,2041263	8,77E-05	0	0
103643958	LOC103643958	calcium-transporting ATPase 3, endoplasmic reticulum-type	0	0	0	0	1,6941005	0,0108512	1,8897353	0,004103	0	0
103651639	LOC103651639	callose synthase 7	0	0	0	0	0	0	1,72653311	0,004938	1,5617566	0,0106103
100280813	LOC100280813	calmodulin	0	0	0	0	0	0	-2,1480133	0,018617	0	0
100282669	LOC100282669	calmodulin binding protein	-2,1920302	0,018894	0	0	-2,032625	0,0249068	0	0	0	0
100285104	LOC100285104	calmodulin binding protein	0	0	0	0	0	0	0	0	3,2240449	0,0076364
103649348	LOC103649348	calmodulin-binding protein 60 C	0	0	0	0	-3,206061	0,0375375	0	0	0	0
100283599	LOC100283599	calmodulin-like protein 1	0	0	0	0	2,9525387	0,0048878	3,15991697	0,00242	2,3260226	0,0222114
100280399	TIDP3279	Calmodulin-like protein 11	-2,2042111	0,020768	-2,8359407	0,0033554	-2,930109	0,0024743	0	0	0	0
100283515	LOC100283515	caltractin	0	0	0	0	1,623245	0,0215209	0	0	0	0
100281576	LOC100281576	carbohydrate binding protein	0	0	0	0	2,3955241	0,0032416	0	0	0	0
100281490	LOC100281490	carbohydrate transporter/ sugar porter	0	0	0	0	2,6511156	0,0386562	2,53771246	0,042628	2,7396663	0,0286571
100282913	LOC100282913	carbohydrate transporter/ sugar porter/ transporter	0	0	1,93415469	0,0467986	0	0	0	0	-2,705014	0,0057199
100191347	LOC100191347	carboxy-lyase	0	0	-2,8975959	0,0254786	0	0	0	0	0	0
100192773	LOC100192773	carrier YEL006W	-2,235306	0,015352	-1,871697	0,0340059	0	0	0	0	0	0
103650532	LOC103650532	CASP-like protein 4C1	-5,4154833	0,002605	0	0	0	0	0	0	0	0
100284347	LOC100284347	catalytic/ oxidoreductase, acting on NADH or NADPH	0	0	0	0	0	0	-3,658359	0,01386	0	0
100284946	LOC100284946	catalytic/ protein phosphatase type 2C	0	0	0	0	0	0	0	0	2,405397	0,0064521
100285228	LOC100285228	catalytic/ protein phosphatase type 2C	0	0	0	0	3,0875922	0,0025035	0	0	0	0
100274519	LOC100274519	Cation/calcium exchanger 2	-6,7690189	0,000261	-4,4984568	0,0064298	-3,024845	0,0456208	3,74414503	0,049413	0	0
100191437	LOC100191437	Cation/H( ) antiporter 19	0	0	0	0	0	0	0	0	4,6182442	0,0316823
100285888	LOC100285888	cationic amino acid transporter	0	0	0	0	-2,687611	0,0117396	0	0	0	0
100194142	IDP630	Cationic amino acid transporter 1	0	0	0	0	1,9673271	0,037313	2,46215914	0,009374	2,6518585	0,0051273
103655400	LOC103655400	CBS domain-containing protein CBSCBSPB3	-2,0080554	0,019204	-2,1350628	0,0126218	0	0	0	0	0	0
103632850	LOC103632850	CCAAT/enhancer-binding protein zeta	0	0	0	0	1,5271913	0,031926	0	0	1,5235401	0,0271069
100383246	CA2P12	CCAAT-HAP2 transcription factor	2,86049682	0,024884	0	0	0	0	0	0	0	0
100383682	LOC100383682	CDPK-related kinase 5	0	0	0	0	2,179336	0,0303434	0	0	2,4834975	0,0115064
103642611	LOC103642611	cell division control protein 48 homolog C-like	-3,3372868	0,014014	-3,473758	0,0102662	-2,85254	0,0299744	0	0	0	0
100283092	LOC100283092	cell division control protein 50	0	0	0	0	2,8952294	0,0002613	2,95206567	0,000151	3,1363837	5,605E-05
103653816	LOC103653816	cell division protein FtsZ homolog 1, chloroplastic	0	0	-2,1520739	0,0463742	0	0	0	0	2,163285	0,0399032

			Yuc x Ctr		Azo x Ctr		AzoYuc x Ctr		AzoYuc x Yuc		AzoYuc x Azo	
Gene ID	Gene Symbol	Gene Description	Log2(FC)	pvalue	Log2(FC)	pvalue	Log2(FC)	pvalue	Log2(FC)	pvalue	Log2(FC)	pvalue
100284584	LOC100284584	cell envelope integrity inner membrane protein TolA	0	0	0	0	2,4175842	0,0235368	2,00967903	0,039483	0	0
100284761	CNR10	cell number regulator 10	0	0	0	0	0	0	0	0	1,6118262	0,0325273
103643347	LOC103643347	cellulose synthase-like protein D3	0	0	5,06376474	0,0139903	0	0	0	0	-3,973211	0,0460747
103646301	LOC103646301	centromere protein X	0	0	5,01593634	0,0293924	0	0	0	0	-6,113971	0,009338
100273712	CER1	cerberus 1, cysteine knot superfamily, homolog	0	0	0	0	4,1809013	0,0097768	0	0	0	0
100283336	LOC100283336	CF9	4,28385127	0,000463	0	0	0	0	-2,7748055	0,004336	0	0
103636567	LOC103636567	chaperone protein ClpB2, chloroplastic-like	2,58535093	0,005347	0	0	0	0	-2,5620512	0,004886	0	0
100192597	LOC100192597	chaperone protein dnaJ	0	0	-2,6447164	0,0097183	0	0	0	0	2,9685713	0,0029576
100272398	LOC100272398	chaperone protein dnaJ	0	0	0	0	3,5359235	0,0068292	4,91870289	0,000926	2,9436845	0,0130559
100382440	cl18199_-2	Chaperone protein dnaJ 16	0	0	0	0	3,0211007	0,008058	0	0	0	0
103649927	LOC103649927	chaperone protein dnaJ 20, chloroplastic	0	0	0	0	-2,25595	0,0069016	-1,8484302	0,026835	0	0
103635523	LOC103635523	chaperone protein dnaJ 49	-2,4486104	0,008422	-2,3337022	0,0104281	-1,748064	0,0481054	0	0	0	0
100285638	LOC100285638	chitinase 2	0	0	-2,2059496	0,0023949	-1,571574	0,0218876	0	0	0	0
542525	chn1	chitinase chem 5	0	0	0	0	-1,766677	0,0348702	0	0	0	0
100273606	LOC100273606	CHL-CPN10	0	0	0	0	3,6168735	0,0336668	3,4056262	0,031328	0	0
542114	pco116691	Chloride channel	0	0	0	0	0	0	-1,5977755	0,016056	0	0
100502532	LOC100502532	chloride channel protein	0	0	0	0	2,3614185	0,0032179	2,67891609	0,000786	1,8888527	0,0142802
100191219	LOC100191219	chloride intracellular channel 6	0	0	0	0	0	0	1,99370863	0,003989	0	0
103632406	LOC103632406	chlorophyllase-2, chloroplastic	0	0	0	0	0	0	0	0	3,9508593	0,0152341
100283886	LOC100283886	chloroplast small heat shock protein	0	0	0	0	0	0	-2,9750486	0,034027	0	0
103650893	LOC103650893	chromatin assembly factor 1 subunit FSM	0	0	-2,2648954	0,023873	-2,086005	0,0362894	0	0	0	0
100501760	LOC100501760	chromatin remodeling factor18	0	0	0	0	0	0	2,13087294	0,007618	1,6142432	0,0346953
100192978	IDP190	CHY-type/CTCHY-type/RING-type Zinc finger protein	2,10368353	0,001003	0	0	0	0	0	0	0	0
100382657	LOC100382657	Cinnamoyl-CoA reductase 1	-1,8789232	0,010404	0	0	0	0	1,88367704	0,008844	0	0
103638788	LOC103638788	cinnamoyl-CoA reductase 1	-1,5707014	0,034458	0	0	0	0	0	0	0	0
100285216	LOC100285216	circadian clock coupling factor ZGT	0	0	0	0	0	0	-2,932187	0,015469	0	0
100283149	pco086826a	Circumsporozoite protein	0	0	0	0	-1,832691	0,0394553	0	0	0	0
109944318	LOC109944318	circumsporozoite protein-like	-2,1346362	0,012722	-1,9789115	0,0180135	-2,28652	0,0071895	0	0	0	0
103636476	LOC103636476	cis-zeatin O-glucosyltransferase 2	0	0	0	0	0	0	0	0	3,3114319	0,0091418
542460	cka2	CK2 protein kinase alpha 2	-1,8921495	0,014651	-2,2505903	0,0038906	0	0	0	0	0	0
100191598	LOC100191598	class II heat shock protein	0	0	0	0	0	0	0	0	-4,000043	0,0194123
103650772	LOC103650772	classical arabinogalactan protein 10-like	0	0	0	0	2,296916	0,0467025	0	0	2,3972854	0,0297578
103647801	LOC103647801	classical arabinogalactan protein 4	0	0	0	0	0	0	2,46350054	0,02687	0	0
100281988	ap17	clathrin coat assembly protein AP17	0	0	0	0	0	0	1,95368071	0,009449	1,6774811	0,0214638
109939631	LOC109939631	clathrin heavy chain 1-like	0	0	-4,975457	0,0342762	0	0	0	0	0	0

			Yuc x Ctr		Azo x Ctr		AzoYuc x Ctr		AzoYuc x Yuc		AzoYuc x Azo	
Gene ID	Gene Symbol	Gene Description	Log2(FC)	pvalue	Log2(FC)	pvalue	Log2(FC)	pvalue	Log2(FC)	pvalue	Log2(FC)	pvalue
103632239	LOC103632239	CO(2)-response secreted protease	0	0	2,2087501	0,0157383	0	0	0	0	-2,065221	0,020916
541628	pco084558	Coatomer subunit zeta-2	1,63833293	0,018443	0	0	0	0	-1,6458758	0,012538	0	0
100856929	LOC100856929	co-chaperone protein SBA1	0	0	-1,9575556	0,00669	-1,784121	0,0129199	0	0	0	0
100282247	LOC100282247	cofactor required for Sp1 transcriptional activation subunit 9	0	0	3,48799461	0,0252653	0	0	0	0	-2,975421	0,0374303
100282954	pco097918	Coiled-coil domain-containing protein 55 (DUF2040)	0	0	0	0	2,7521605	0,0184572	2,48120264	0,0277	2,8781821	0,0113591
109944797	LOC109944797	cold and drought-regulated protein CORA	0	0	0	0	1,66273	0,0042862	0	0	0	0
542099	gpm455	Cold-regulated 413 plasma membrane protein 2	0	0	1,55526119	0,0027924	0	0	0	0	-1,656786	0,0009743
100279627	LOC100279627	Collagen, type IV, alpha 5	0	0	0	0	2,1861412	0,0399002	2,46373027	0,019668	0	0
100216826	pco089076	COMPASS-like H3K4 histone methylase component WDR5B	0	0	0	0	-3,108325	0,016823	0	0	0	0
103631772	LOC103631772	conserved oligomeric Golgi complex subunit 4	0	0	0	0	0	0	1,52992505	0,040718	0	0
100281883	LOC100281883	CONSTANS-like protein CO8	0	0	0	0	2,052635	0,0117929	1,80902039	0,022191	2,0571798	0,0094395
100280950	cl60180_1(477)	COP9 signalosome complex subunit 8	0	0	0	0	4,5345404	0,0148065	0	0	0	0
100282864	LOC100282864	copper chaperone	0	0	0	0	-1,622501	0,0073351	0	0	0	0
103639611	LOC103639611	Copper transport protein family	3,75527211	0,003588	0	0	0	0	0	0	0	0
103646092	LOC103646092	copper-transporting ATPase HMA5	0	0	0	0	3,0584435	0,005096	3,40809422	0,001672	0	0
100304350	LOC100304350	Copper-transporting atpase paa1	0	0	0	0	0	0	2,28234466	0,00928	1,8933409	0,0245411
100384228	LOC100384228	Core-2/I-branching beta-16-N-acetylglucosaminyltransferase family protein	0	0	0	0	2,6882403	0,0017644	3,26051912	0,000165	2,5025707	0,0028427
100285831	LOC100285831	cortical cell-delineating protein	0	0	0	0	0	0	-4,5558173	0,016013	0	0
103627516	LOC103627516	cortical cell-delineating protein-like	0	0	0	0	0	0	0	0	2,1200322	0,0143945
103638952	LOC103638952	cortical cell-delineating protein-like	0	0	0	0	-1,732087	0,0085981	0	0	0	0
100281983	LOC100281983	CP5	0	0	0	0	-1,71023	0,033365	-1,7039669	0,030832	0	0
103637336	LOC103637336	CRIB domain-containing protein RIC10	0	0	0	0	2,1599039	0,0467991	2,98641795	0,007452	3,2175684	0,0039831
103645904	LOC103645904	CRIB domain-containing protein RIC10	0	0	0	0	-4,658435	0,026928	0	0	0	0
103648377	LOC103648377	CRIB domain-containing protein RIC10	0	0	-5,4970544	0,0062336	-4,565607	0,0231009	0	0	0	0
542346	cr4	crinkly 4	-2,4421149	0,02586	0	0	0	0	0	0	0	0
100126360	CFM3	CRM family member 3	0	0	0	0	0	0	0	0	2,8013417	0,0098432
103632644	LOC103632644	CRM-domain containing factor CFM3, chloroplastic/mitochondrial	0	0	0	0	1,6186101	0,042224	2,28106723	0,004808	2,0487469	0,009386
100272285	LOC100272285	CRS2-associated factor 1 mitochondrial	0	0	15,5794206	0,0002802	20,107039	2,218E-06	20,7881869	9,94E-07	0	0

			Yuc x Ctr		Azo x Ctr		AzoYuc x Ctr		AzoYuc x Yuc		AzoYuc x Azo	
Gene ID	Gene Symbol	Gene Description	Log2(FC)	pvalue	Log2(FC)	pvalue	Log2(FC)	pvalue	Log2(FC)	pvalue	Log2(FC)	pvalue
100285653	LOC100285653	CTP synthase	0	0	3,09840529	0,0015921	0	0	0	0	-3,307515	0,0005975
100384369	LOC100384369	CwfJ-like family protein	0	0	0	0	0	0	1,71504317	0,022523	1,7959967	0,0164111
100502334	LOC100502334	Cyclase family protein	0	0	0	0	1,5071428	0,0221001	0	0	0	0
100281868	PYL5	cyclase/dehydrase family protein	0	0	0	0	0	0	0	0	1,9168273	0,0207481
100381571	LOC100381571	Cyclic dof factor 2	3,45841159	0,044076	3,80735436	0,0246051	3,5759657	0,0354978	0	0	0	0
100272382	LOC100272382	Cyclic nucleotide-gated ion channel 2	0	0	0	0	-3,055241	0,0322184	0	0	0	0
542307	cyc1	*cyclin 1 (cyc 1b)*	0	0	2,19035952	0,0480894	0	0	0	0	-2,419525	0,0250164
542336	cyc2	cyclin 2	0	0	0	0	-2,436033	0,0294178	0	0	0	0
100193909	gpm643	cyclin12	0	0	0	0	0	0	0	0	1,9697649	0,0463976
103649996	LOC103649996	cyclin-A1-1-like	0	0	-2,0880502	0,0166301	0	0	0	0	1,9275164	0,0236577
103654926	LOC103654926	cycloartenol synthase	0	0	0	0	2,0226132	0,0049835	2,11697782	0,003059	1,8797954	0,0080211
103645750	LOC103645750	cysteine protease ATG4B-like	1,71162116	0,042254	0	0	0	0	0	0	0	0
103633255	LOC103633255	cysteine-rich receptor-like protein kinase 10	0	0	0	0	0	0	-2,1632469	0,046454	0	0
103633255	LOC103633265	cysteine-rich receptor-like protein kinase 10	0	0	0	0	0	0	0	0	2,0607222	0,0017769
103633256	LOC103633256	cysteine-rich receptor-like protein kinase 19	0	0	0	0	1,5020347	0,0300794	0	0	0	0
100037793	cda2	cytidine deaminase 2	-2,5884074	0,002326	-2,0278691	0,0122501	-1,701777	0,0324776	0	0	0	0
100280947	LOC100280947	cytidine/deoxycytidylate deaminase family protein	-1,7006908	0,016778	0	0	0	0	0	0	0	0
103632988	LOC103632988	Cytochrome b561 and DOMON domain-containing protein	0	0	0	0	0	0	-1,5931613	0,00448	0	0
100384526	LOC100384526	cytochrome c oxidase assembly protein ctaG	3,23272112	0,043402	0	0	0	0	0	0	0	0
103649703	MAX1b	cytochrome P450 711A1-like	0	0	0	0	1,8141044	0,0201409	0	0	0	0
103632819	LOC103632819	cytochrome P450 71A1	0	0	0	0	0	0	0	0	1,9402357	0,0048514
103649549	LOC103649549	cytochrome P450 71A1	0	0	0	0	0	0	3,53018431	0,017695	0	0
103652681	LOC103652681	cytochrome P450 71A1	0	0	0	0	4,4879083	0,0046875	4,01728596	0,004945	2,5950592	0,0443925
103651469	LOC103651469	cytochrome P450 72A13	0	0	0	0	0	0	-3,5818444	0,037397	-3,542258	0,0388887
103639316	LOC103639316	cytochrome P450 88A1-like	0	0	0	0	0	0	0	0	1,9533247	0,001428
103647105	LOC103647105	cytochrome P450 90A4	0	0	0	0	0	0	0	0	4,4188211	0,0159243
109944638	LOC109944638	cytochrome P450 93G2	0	0	0	0	-3,956128	0,0494114	0	0	0	0
542585	cko1	cytokinin oxidase 1	-2,6246513	0,017257	-2,1210556	0,0450746	0	0	0	0	0	0
100383171	LOC100383171	Cytokinin riboside 5'-monophosphate phosphoribohydrolase LOG3	0	0	0	0	3,125981	0,044433	0	0	0	0
100281698	LOC100281698	cytokinin-O-glucosyltransferase 3	0	0	0	0	2,5834166	0,0078036	0	0	2,0563178	0,0235088
100192698	TIDP3497	cytoplasmic membrane protein	-2,5263171	0,003785	0	0	-2,663079	0,002126	0	0	0	0
100276448	LOC100276448	Cytoplasmic protein of eukaryotic origin (38.3 kD)	0	0	-2,0110144	0,0460284	0	0	0	0	0	0

			Yuc x Ctr		Azo x Ctr		AzoYuc x Ctr		AzoYuc x Yuc		AzoYuc x Azo	
Gene ID	Gene Symbol	Gene Description	Log2(FC)	pvalue	Log2(FC)	pvalue	Log2(FC)	pvalue	Log2(FC)	pvalue	Log2(FC)	pvalue
103635582	LOC103635582	cytosolic endo-beta-N-acetylglucosaminidase 1	0	0	0	0	0	0	0	0	1,6438596	0,0173164
100284660	LOC100284660	cytosolic purine 5-nucleotidase	-2,1726603	0,008238	-1,7775213	0,0283474	0	0	0	0	0	0
103650751	LOC103650751	cytosolic sulfotransferase 12-like	0	0	0	0	-3,735134	0,0106799	0	0	0	0
100273608	si618066c08	D-2-hydroxyglutarate dehydrogenase mitochondrial	-2,2335715	0,002297	-1,7105113	0,0144334	-1,909998	0,0067402	0	0	0	0
100193788	AY104775	D-aminoacyl-tRNA deacylase	0	0	-2,1145649	0,0207985	0	0	0	0	2,1245012	0,0174785
111591339	LOC111591339	DBF4-type zinc finger-containing protein 2 homolog	0	0	0	0	0	0	2,74551124	0,02738	0	0
100381417	LOC100381417	dcl101 - dicer-like 101	2,13559011	0,004441	0	0	0	0	-1,8397801	0,011654	0	0
100282616	LOC100282616	DCN1-like protein 4	0	0	0	0	2,2395877	0,0151896	2,12778181	0,018596	4,1459477	3,54E-05
103646181	LOC103646181	DDB1- and CUL4-associated factor homolog 1	2,54775045	0,005377	0	0	0	0	-2,0515462	0,021064	0	0
103648226	LOC103648226	DDT domain-containing protein DDR4	1,91294473	0,035008	0	0	0	0	0	0	0	0
103627856	LOC103627856	DEAD-box ATP-dependent RNA helicase 1	0	0	0	0	-2,101732	0,0300369	0	0	0	0
100273968	pco063852	DEAD-box ATP-dependent RNA helicase 16	0	0	0	0	0	0	0	0	1,8403491	0,0250582
100381583	LOC100381583	DEAD-box ATP-dependent RNA helicase 40	0	0	0	0	2,5650971	0,0019307	2,14456221	0,007933	2,4485201	0,0025013
109941472	LOC109941472	DEAD-box ATP-dependent RNA helicase 53	0	0	-3,5003595	0,0475603	0	0	0	0	0	0
100279147	LOC100279147	DEAD-box helicase family protein	3,48477248	0,017208	0	0	0	0	0	0	0	0
100286109	LOC100286109	dehydration-responsive element-binding protein 2A	0	0	0	0	2,9145688	0,02201	0	0	0	0
542373	dhn1	dehydrin 1	0	0	0	0	0	0	0	0	-3,902847	0,0098891
100281087	LOC100281087	dehydrin COR410	0	0	1,60319668	0,0216371	0	0	0	0	0	0
103655617	LOC103655617	delta(3,5)-Delta(2,4)-dienoyl-CoA isomerase, peroxisomal-like	0	0	2,56061443	0,0186702	0	0	0	0	0	0
103642098	LOC103642098	dentin sialophosphoprotein	0	0	0	0	2,4336593	0,0125658	2,20531725	0,01879	1,9950959	0,0305005
100383871	pco124991(180)	DExH-box ATP-dependent RNA helicase DExH11	0	0	0	0	1,9086489	0,0072063	0	0	0	0
103646444	LOC103646444	DExH-box ATP-dependent RNA helicase DExH7, chloroplastic	0	0	0	0	0	0	1,92694341	0,005863	1,9499951	0,0051787
103636484	LOC103636484	diacylglycerol kinase 1	0	0	0	0	0	0	0	0	-1,930711	0,0437639
100193580	LOC100193580	dihydroflavonol-4-reductase	-1,9527932	0,037616	-2,4923822	0,0086523	-2,385847	0,0115105	0	0	0	0
100284190	LOC100284190	dihydroflavonol-4-reductase	-2,2323283	0,027611	0	0	-1,971818	0,0447287	0	0	0	0
100284846	LOC100284846	dihydroflavonol-4-reductase	-2,4416123	0,011148	0	0	0	0	0	0	0	0
100273676	LOC100273676	Dihydroxy-acid dehydratase chloroplastic	0	0	0	0	0	0	2,05072368	0,006117	1,7212603	0,0186063

			Yuc x Ctr		Azo x Ctr		AzoYuc x Ctr		AzoYuc x Yuc		AzoYuc x Azo	
Gene ID	Gene Symbol	Gene Description	Log2(FC)	pvalue	Log2(FC)	pvalue	Log2(FC)	pvalue	Log2(FC)	pvalue	Log2(FC)	pvalue
100384514	LOC100384514	Dihydroxy-acid dehydratase chloroplastic	0	0	0	0	2,5574274	0,0030958	0	0	2,4654361	0,0029783
100284663	LOC100284663	diphthamide biosynthesis protein 1	3,32938881	0,031149	0	0	0	0	0	0	0	0
100280569	LOC100280569	diphthamide biosynthesis protein 3	0	0	0	0	0	0	-2,6048643	0,013651	0	0
100286315	LOC100286315	diphthamide biosynthesis protein 3	0	0	0	0	0	0	-3,504039	0,005313	0	0
100279605	cl27369_1	diphthamide synthesis DPH2 family protein	0	0	0	0	5,2148319	0,0006288	2,29214065	0,031173	2,3381419	0,0265795
100285699	LOC100285699	dirigent protein 1	-2,4871687	0,020845	-2,6572299	0,01346	-2,74913	0,0106719	0	0	0	0
103652771	LOC103652771	dirigent protein 1	0	0	0	0	0	0	2,02868186	0,04108	0	0
100283195	LOC100283195	dirigent protein 22	0	0	0	0	0	0	0	0	1,8489061	0,0202232
103640842	LOC103640842	dirigent protein 25	0	0	0	0	-2,306641	0,0330482	0	0	0	0
100217120	LOC100217120	disease resistance analog PIC11	0	0	0	0	0	0	-2,3229245	0,018255	0	0
103645190	LOC103645190	disease resistance protein RGA2	0	0	0	0	5,215107	0,0103241	0	0	4,9019895	0,015918
103645703	LOC103645703	disease resistance protein RPP13	0	0	0	0	0	0	-2,3743039	0,0073	0	0
100217241	IDP866	D-isomer specific 2-hydroxyacid dehydrogenase family protein	0	0	0	0	0	0	0	0	1,7978788	0,0115772
100280929	LOC100280929	DNA binding protein	0	0	0	0	0	0	3,57809046	0,044473	0	0
100284430	LOC100284430	DNA binding protein	0	0	0	0	-2,519919	0,0398068	0	0	0	0
100285427	LOC100285427	DNA binding protein	-1,7388736	0,047661	0	0	-1,98936	0,023454	0	0	0	0
103640309	LOC103640309	DNA helicase INO80	0	0	0	0	0	0	0	0	-1,5166	0,0179179
100382240	gpm261	DNA mismatch repair protein MutS type 2	0	0	0	0	0	0	0	0	2,6859648	0,0112831
103642101	LOC103642101	DNA polymerase I A, chloroplastic	2,75201878	0,004984	2,60707806	0,0077341	2,3793052	0,0154707	0	0	0	0
103627069	LOC103627069	DNA polymerase I B, mitochondrial	0	0	0	0	5,9338116	0,0001888	3,79593023	0,001736	3,0564885	0,0076185
103650372	LOC103650372	DNA polymerase V family	0	0	0	0	0	0	0	0	3,4843763	0,0486476
100284087	LOC100284087	DNA repair protein RAD51	0	0	0	0	0	0	0	0	2,6044882	0,0458463
100191367	IDP355	DNA repair protein recA homolog 3 mitochondrial	0	0	3,39680087	0,0435764	3,3338307	0,0477119	0	0	0	0
103644907	LOC103644907	DNA-(apurinic or apyrimidinic site) lyase, chloroplastic	0	0	0	0	0	0	3,02683864	0,045662	0	0
100284738	LOC100284738	DNA-binding protein RAV1	2,23897122	0,039055	0	0	0	0	0	0	0	0
103646512	LOC103646512	DNA-binding protein S1FA	1,67931918	0,024673	0	0	0	0	0	0	0	0
100281783	LOC100281783	DNA-directed RNA polymerase II 8.2 kDa polypeptide	0	0	-1,7841963	0,0303196	0	0	0	0	0	0
103654914	LOC103654914	DNA-directed RNA polymerase V subunit 1	0	0	0	0	2,0521124	0,0090059	1,91890318	0,013448	2,0273952	0,0089252
100217277	cl803_1	DNA-directed RNA polymerases I, II, and III 7.3 kDa polypeptide	-2,7016958	0,004086	0	0	0	0	0	0	0	0
100281852	LOC100281852	dnaJ domain containing protein	-2,839622	0,029478	-3,4913944	0,009354	-2,806106	0,0288363	0	0	0	0
100192576	cl2587_-2b	DNAJ heat shock N-terminal domain-containing protein	3,16711456	0,029939	3,03617775	0,0370261	0	0	0	0	0	0

			Yuc x Ctr		Azo x Ctr		AzoYuc x Ctr		AzoYuc x Yuc		AzoYuc x Azo	
Gene ID	Gene Symbol	Gene Description	Log2(FC)	pvalue	Log2(FC)	pvalue	Log2(FC)	pvalue	Log2(FC)	pvalue	Log2(FC)	pvalue
100193395	LOC100193395	DNAJ heat shock N-terminal domain-containing protein	-6,8555886	0,000127	-6,1197269	0,000625	-4,780145	0,0028381	0	0	0	0
103641254	LOC103641254	dnaJ homolog subfamily B member 4	0	0	0	0	0	0	0	0	2,3272211	0,0300752
103630059	LOC103630059	dnaJ protein ERDJ3B	0	0	0	0	2,0691977	0,0038725	0	0	0	0
103636634	LOC103636634	dof zinc finger protein 5	-4,3563577	0,012638	0	0	0	0	0	0	0	0
100277177	LOC100277177	Dof zinc finger protein DOF1.6	0	0	2,45393547	0,0281307	2,3359934	0,0369381	0	0	0	0
100275168	DOF22	Dof zinc finger protein DOF2.5	-2,134909	0,016771	0	0	-1,850607	0,0354186	0	0	0	0
100283759	LOC100283759	dof zinc finger protein MNB1A	0	0	0	0	2,7387573	0,0055909	2,27829893	0,014058	1,8839665	0,0367662
100382512	LOC100382512	Dolichyl-diphosphooligosaccharide--protein glycosyltransferase subunit STT3B	0	0	0	0	2,1080579	0,0081131	2,41319167	0,00237	2,180186	0,0055523
100856943	LOC100856943	dopamine beta-monooxygenase	-1,5042395	0,032951	0	0	0	0	0	0	0	0
100192502	LOC100192502	Dormancy-associated protein homolog 3	2,83637576	0,010517	0	0	4,2949143	9,196E-05	0	0	2,3509325	0,0157302
100280218	LOC100280218	Double-stranded RNA-binding protein 3	-1,9476549	0,049437	0	0	0	0	0	0	0	0
100285381	LOC100285381	dual specificity protein phosphatase 4	0	0	3,45163456	0,0422805	0	0	0	0	0	0
103639513	LOC103639513	DUF1677 family protein	0	0	0	0	0	0	1,83664244	0,041811	1,7886982	0,0445652
103635529	LOC103635529	DUF21 domain-containing protein At4g14240	0	0	0	0	1,61529	0,0401238	1,85658277	0,017966	2,0645355	0,0085331
100277681	LOC100277681	DUF3475 domain protein	0	0	0	0	2,3558303	0,022277	0	0	2,0974807	0,0357825
100276613	LOC100276613	E2F-associated phosphoprotein	0	0	0	0	3,3437143	0,0296762	4,16009386	0,007464	4,5357314	0,0037369
100279342	LOC100279342	E3 ubiquitin-protein ligase COP1	0	0	0	0	-3,608559	0,0143112	0	0	0	0
103645598	LOC103645598	E3 ubiquitin-protein ligase HOS1	0	0	2,2799394	0,0108604	0	0	0	0	0	0
103647118	LOC103647118	E3 ubiquitin-protein ligase listerin	0	0	0	0	-1,806432	0,0007189	0	0	0	0
103649729	LOC103649729	E3 ubiquitin-protein ligase MBR1	0	0	0	0	-1,737079	0,0441658	0	0	0	0
103626988	LOC103626988	E3 ubiquitin-protein ligase Os03g0188200	0	0	0	0	3,7193501	0,0410641	0	0	3,9568878	0,0237098
103629212	LOC103629212	E3 UFM1-protein ligase 1 homolog	0	0	-1,624331	0,0466128	0	0	0	0	0	0
100284128	LOC100284128	early nodulin 93	3,28345255	0,001052	0	0	0	0	0	0	0	0
103638099	LOC103638099	early nodulin 93	0	0	0	0	0	0	-4,2342439	0,032536	-4,807549	0,0139555
109940360	LOC109940360	early nodulin-20-like	0	0	0	0	0	0	4,40130813	0,019864	0	0
103626650	LOC103626650	early nodulin-like protein 2	0	0	-4,0393176	0,0447157	0	0	0	0	0	0
100191322	LOC100191322	Electron transfer flavoprotein subunit alpha mitochondrial	0	0	0	0	-1,911297	0,0269799	0	0	0	0
103628645	LOC103628645	electron transfer flavoprotein subunit alpha, mitochondrial	-3,6618353	0,001509	-3,9659947	0,0006316	-6,402765	2,049E-05	0	0	0	0
103646146	LOC103646146	elongation factor G-2, chloroplastic	-3,3732688	0,019215	-2,9469183	0,03426	0	0	0	0	0	0
100192861	pco086097	elongation factor Ts family protein	0	0	0	0	0	0	0	0	1,5481705	0,0171572
100284040	LOC100284040	elongation factor Tu	-1,896836	0,008961	-1,8973765	0,0081486	0	0	0	0	0	0
109944360	LOC109944360	elongation of fatty acids protein 3-like	0	0	0	0	0	0	0	0	-1,83178	0,003323

			Yuc x Ctr		Azo x Ctr		AzoYuc x Ctr		AzoYuc x Yuc		AzoYuc x Azo	
Gene ID	Gene Symbol	Gene Description	Log2(FC)	pvalue	Log2(FC)	pvalue	Log2(FC)	pvalue	Log2(FC)	pvalue	Log2(FC)	pvalue
100285891	LOC100285891	EMB1688	0	0	1,98647184	0,0361916	0	0	0	0	0	0
100193325	LOC100193325	embryo defective12	0	0	0	0	-2,905616	0,0221135	0	0	0	0
103641656	LOC103641656	endochitinase A	0	0	0	0	-2,016182	0,0201105	-2,104797	0,013678	0	0
100216890	LOC100216890	Endochitinase B	0	0	0	0	-2,832122	0,0377711	0	0	0	0
103654079	LOC103654079	endoglucanase 7-like	0	0	0	0	2,3865099	0,0053131	1,70689212	0,035597	2,4163167	0,0035553
100383198	LOC100383198	Endosomal targeting BRO1-like domain-containing protein	0	0	0	0	-1,540926	0,0031059	0	0	0	0
103628836	LOC103628836	enhancer of mRNA-decapping protein 4-like	3,02448578	0,006969	0	0	2,2411878	0,0465935	0	0	0	0
103653754	LOC103653754	enhancer of rudimentary homolog	0	0	0	0	2,8450811	0,0248339	3,65594694	0,004701	3,3602778	0,0077826
100274024	LOC100274024	ENT domain containing protein	0	0	0	0	0	0	3,80009987	0,015909	0	0
100282274	LOC100282274	enzyme of the cupin superfamily	0	0	0	0	3,16083	0,0348726	0	0	0	0
103629877	LOC103629877	epidermal growth factor receptor substrate 15	0	0	0	0	0	0	1,93270584	0,023697	0	0
103639543	LOC103639543	epidermal growth factor receptor substrate 15-like 1	0	0	0	0	1,8352552	0,0001226	1,58812095	0,00061	0	0
103635715	LOC103635715	ER membrane protein complex subunit 1-like	0	0	0	0	1,6718203	0,0176367	1,88049225	0,007417	1,7113357	0,014388
100383941	LOC100383941	er1 (erecta-like1)	-4,5729042	0,008439	0	0	0	0	4,27785452	0,012992	0	0
100285913	LOC100285913	EREBP-4 like protein	1,87760516	0,040862	0	0	0	0	0	0	0	0
100283804	LOC100283804	erwinia induced protein 1	-3,5151812	0,021482	0	0	0	0	3,28439988	0,029531	0	0
100285793	LOC100285793	esterase	0	0	0	0	2,7309993	0,0020253	2,74520158	0,001681	2,1852143	0,0110287
100285966	LOC100285966	ethylene response factor 1	0	0	0	0	0	0	-3,9817113	0,026815	-3,556116	0,0495951
100216792	LOC100216792	Ethylene-insensitive protein 2	0	0	0	0	5,0636999	0,0111089	0	0	4,0371514	0,0346225
100285594	LOC100285594	ethylene-insensitive3-like protein	0	0	-1,9944471	0,0463303	-2,164211	0,0323866	0	0	0	0
100282305	LOC100282305	ethylene-responsive transcription factor 4	0	0	0	0	-2,35788	0,0384014	0	0	0	0
103655480	LOC103655480	ethylene-responsive transcription factor ERF024	-4,6519151	0,017407	-5,8396422	0,0028344	-5,869989	0,002694	0	0	0	0
103627502	LOC103627502	ethylene-responsive transcription factor ERF043	0	0	0	0	3,4094368	0,0145896	4,28338608	0,002822	4,0582689	0,003701
103627463	LOC103627463	ethylene-responsive transcription factor ERF053	0	0	0	0	6,7506503	0,0115204	8,13515451	0,002329	5,2151654	0,0361804
103655274	LOC103655274	ethylene-responsive transcription factor ERF096	-4,2998189	0,046337	0	0	0	0	0	0	0	0
103638017	LOC103638017	ethylene-responsive transcription factor RAP2-1	0	0	0	0	2,9659788	0,0352906	0	0	0	0
103629531	LOC103629531	ethylene-responsive transcription factor RAP2-9	0	0	0	0	0	0	0	0	-2,733324	0,0272001
111590522	LOC111590522	Eukaryotic 28S ribosomal RNA	0	0	0	0	0	0	2,1924217	0,03313	0	0

			Yuc x Ctr		Azo x Ctr		AzoYuc x Ctr		AzoYuc x Yuc		AzoYuc x Azo	
Gene ID	Gene Symbol	Gene Description	Log2(FC)	pvalue	Log2(FC)	pvalue	Log2(FC)	pvalue	Log2(FC)	pvalue	Log2(FC)	pvalue
100272542	LOC100272542	Eukaryotic aspartyl protease family protein	2,95475097	0,003921	0	0	0	0	-2,7396745	0,004111	0	0
100273869	LOC100273869	Eukaryotic aspartyl protease family protein	0	0	0	0	2,1531062	0,0032866	1,69597907	0,0171	0	0
100383437	LOC100383437	Eukaryotic aspartyl protease family protein	-2,8712072	0,011647	0	0	-2,495772	0,0193003	0	0	0	0
100383436	si687085b03	evolutionarily conserved C-terminal region 7	-1,6849752	0,0046	0	0	0	0	0	0	0	0
103654917	LOC103654917	exocyst complex component EXO70A1	0	0	0	0	3,1370948	0,0458852	0	0	0	0
100304442	LOC100304442	Exocyst complex component SEC5A	-2,0101382	0,000939	0	0	0	0	0	0	0	0
100382718	LOC100382718	Exosome complex component RRP41-like	0	0	2,35692615	0,0475644	2,6302399	0,0262799	0	0	0	0
100193137	LOC100193137	exosome complex exonuclease RRP43	0	0	-4,7100876	0,0040897	0	0	0	0	3,5288496	0,0345156
103631736	LOC103631736	expansin-A15	4,58059281	0,004955	0	0	0	0	-3,8791905	0,00624	0	0
103643533	LOC103643533	expansin-A31	0	0	0	0	2,8957645	0,0026712	0	0	1,9714057	0,0272719
542566	pco112411b	Expansin-B4	0	0	-1,9386698	0,0412156	0	0	0	0	0	0
109942624	LOC109942624	extensin-like	0	0	3,39640809	0,0332102	0	0	0	0	0	0
111590123	LOC111590123	extensin-like	0	0	0	0	2,6202163	0,021333	2,49103432	0,025182	3,2449722	0,0043756
103636591	LOC103636591	external alternative NAD(P)H-ubiquinone oxidoreductase B3, mitochondrial	0	0	-4,3071272	0,0145501	0	0	0	0	0	0
103650928	LOC103650928	extradiol ring-cleavage dioxygenase	0	0	0	0	0	0	1,57524454	0,031606	0	0
103638537	LOC103638537	Extra-large G-protein-like	-1,8617445	0,031924	-2,6529248	0,0034225	0	0	0	0	0	0
103651481	LOC103651481	Extra-large G-protein-like	0	0	0	0	1,6988735	0,0257233	0	0	0	0
100272684	TIDP2929	Farnesol kinase chloroplastic	3,13726931	0,024727	2,99863496	0,0316838	0	0	0	0	0	0
100281644	umc2310	Farnesylated protein 2	0	0	1,75341514	0,0315052	0	0	0	0	-2,246408	0,0056343
109943165	LOC109943165	fatty acid amide hydrolase	0	0	0	0	0	0	-1,8821494	0,049807	0	0
100273168	fab1	fatty acid biosynthesis 1	-1,6090492	0,029873	-1,7571721	0,0172564	-2,987321	0,0002592	0	0	0	0
100285225	LOC100285225	fatty acid elongase	0	0	0	0	0	0	0	0	-1,568178	0,0422079
100281105	LOC100281105	F-box domain containing protein	0	0	0	0	0	0	1,71389064	0,022759	0	0
100285790	LOC100285790	F-box domain containing protein	0	0	0	0	-2,592753	0,0436922	0	0	0	0
100382223	umc1789	F-box protein AFR	0	0	2,23738064	0,0162292	3,0540311	0,0008617	0	0	0	0
100501731	LOC100501731	F-box protein At1g67340	-2,2092433	0,007873	0	0	-1,965973	0,0147281	0	0	0	0
100274363	FBL2	F-box protein FBL2	0	0	1,82948619	0,0373473	1,7341694	0,0486583	0	0	0	0
103641127	LOC103641127	F-box protein SKIP19	0	0	0	0	0	0	0	0	4,5008811	0,0340432
103645791	LOC103645791	F-box/FBD/LRR-repeat protein At1g13570	0	0	0	0	2,9809028	0,0335968	0	0	3,1205971	0,0145475
103647976	LOC103647976	F-box/kelch-repeat protein At2g44130-like	0	0	0	0	2,6512046	0,0051044	0	0	0	0

			Yuc x Ctr		Azo x Ctr		AzoYuc x Ctr		AzoYuc x Yuc		AzoYuc x Azo	
Gene ID	Gene Symbol	Gene Description	Log2(FC)	pvalue	Log2(FC)	pvalue	Log2(FC)	pvalue	Log2(FC)	pvalue	Log2(FC)	pvalue
103626964	LOC103626964	F-box/kelch-repeat protein At3g61590-like	0	0	0	0	2,2312712	0,0260559	0	0	1,8380376	0,0494069
100276488	LOC100276488	Fcf2 pre-rRNA processing protein	0	0	0	0	2,5183117	0,0266947	2,25510298	0,037638	0	0
103625981	LOC103625981	ferredoxin	-2,530381	0,01169	-3,2767636	0,0019716	0	0	0	0	2,3777768	0,0254507
100284586	umc2662	Ferredoxin	0	0	0	0	0	0	1,53851915	0,012807	0	0
542710	fgs1	ferredoxin-dependent glutamate synthase 1	-1,8362217	0,003611	0	0	0	0	0	0	0	0
542392	fer2	ferritin homolog 2	-2,5382716	0,00394	0	0	0	0	0	0	0	0
100285493	LOC100285493	fertility restorer	0	0	0	0	3,7177808	0,0058448	2,24538457	0,047611	2,2477326	0,0451852
100279983	LOC100279983	Fes1A	0	0	0	0	0	0	-1,5609007	0,046473	0	0
100282273	LOC100282273	fiber protein Fb34	0	0	0	0	-2,551704	0,0274285	0	0	0	0
100147735	FtsH2B	filamentation temperature-sensitive H 2B	0	0	0	0	0	0	0	0	-1,560618	0,0450659
103627410	LOC103627410	filament-like plant protein 7	0	0	0	0	0	0	3,3444936	0,027448	0	0
100284924	LOC100284924	FIP1	0	0	0	0	0	0	0	0	-2,442609	0,039268
100285758	LOC100285758	FK506-binding protein 2-1	0	0	0	0	1,5950211	0,0208848	0	0	0	0
103642860	LOC103642860	flavanone 3-dioxygenase 2	-4,5096579	0,004522	0	0	0	0	3,86535508	0,01487	0	0
103653333	LOC103653333	flavonoid 3'-monooxygenase	0	0	0	0	0	0	2,264265	0,012484	0	0
100273153	LOC100273153	Flavonoid 3-monooxygenase	-1,54756	0,010623	0	0	0	0	0	0	0	0
100284951	LOC100284951	flavonoid 3-monooxygenase	0	0	0	0	2,9035075	0,0037872	3,43320705	0,000669	3,6080127	0,0003418
100283644	IDP582	Flavonol synthase-like protein	-1,9104646	0,044517	0	0	0	0	1,95163333	0,036002	0	0
100191666	LOC100191666	Flowering locus K homology domain	0	0	0	0	3,3510651	0,0293196	0	0	0	0
100280079	LOC100280079	Folate synthesis bifunctional protein	0	0	0	0	0	0	0	0	2,2227739	0,0197561
103644171	LOC103644171	folate-biopterin transporter 1, chloroplastic	0	0	0	0	2,5011702	0,0261902	3,19582865	0,004937	4,0994326	0,0004974
103639425	LOC103639425	folate-biopterin transporter-like	0	0	0	0	4,5057588	0,0170482	4,22129328	0,020593	0	0
109941917	LOC109941917	formate dehydrogenase 1, mitochondrial	0	0	0	0	0	0	0	0	2,6775576	0,0311762
103636403	LOC103636403	formin-like protein 1	-1,7550734	0,040767	0	0	-1,793823	0,0350584	0	0	0	0
109939261	LOC109939261	formin-like protein 3	0	0	3,65029284	0,0238396	0	0	0	0	0	0
103626135	LOC103626135	formin-like protein 4	-2,0421587	0,031452	0	0	0	0	0	0	0	0
103642998	LOC103642998	formin-like protein 5	0	0	0	0	-2,270968	0,0432026	0	0	0	0
103647127	LOC103647127	formin-like protein 5	0	0	0	0	0	0	1,5030798	0,015022	0	0
103647807	LOC103647807	formin-like protein 5	-2,214508	0,002696	-2,7725504	0,0002009	-1,746309	0,0163748	0	0	0	0
103637981	LOC103637981	formin-like protein 6	0	0	0	0	2,0868035	0,0259309	0	0	0	0
100284739	LOC100284739	fructose-1,6-bisphosphatase, cytosolic	0	0	0	0	2,3754703	0,0364479	0	0	0	0
100191560	LOC100191560	ftsH6-Zea mays FtsH protease	0	0	0	0	-4,528122	0,0419646	0	0	0	0
103637886	LOC103637886	fucosyltransferase 2	0	0	0	0	3,4371149	0,0078147	4,38898208	0,00177	2,9018276	0,0104793
100283491	LOC100283491	GAGA-binding protein	0	0	0	0	0	0	0	0	1,8154209	0,0262782
606403	LOC606403	galactinol synthase 1	0	0	0	0	-4,747049	0,0050951	-4,239728	0,012589	-5,078076	0,0022587
606405	gols2	galactinol synthase 2	0	0	2,89719956	0,0095114	0	0	0	0	-3,68888	0,0010736

			Yuc x Ctr		Azo x Ctr		AzoYuc x Ctr		AzoYuc x Yuc		AzoYuc x Azo	
Gene ID	Gene Symbol	Gene Description	Log2(FC)	pvalue	Log2(FC)	pvalue	Log2(FC)	pvalue	Log2(FC)	pvalue	Log2(FC)	pvalue
103650935	LOC103650935	Galactose-binding protein	1,69503892	0,033702	0	0	0	0	0	0	0	0
100284640	IDP3798	Gamma-glutamyl peptidase 1	0	0	0	0	2,1014584	0,0202331	0	0	2,2208882	0,0112994
100275899	LOC100275899	gamma-glutamylcyclotransferase 2-2	0	0	0	0	0	0	2,23625466	0,003159	0	0
100280591	LOC100280591	gamma-thionins family protein	0	0	0	0	0	0	3,83360457	0,036369	0	0
100280166	LOC100280166	Gamma-tubulin complex component 3	0	0	0	0	0	0	0	0	2,1397202	0,0496782
100281895	LOC100281895	GATA transcription factor 25	0	0	0	0	0	0	4,38662052	0,03701	0	0
100281278	GATA28	GATA transcription factor 9	0	0	0	0	0	0	0	0	-2,234236	0,0377397
100283313	bZIP116	G-box-binding factor 4	-1,7945772	0,045025	0	0	0	0	0	0	0	0
103638214	LOC103638214	GDSL esterase/lipase At5g37690	0	0	0	0	-1,810331	0,0231059	-1,7681066	0,024031	0	0
103626464	LOC103626464	GDSL esterase/lipase At5g55050	0	0	-4,7268655	0,00716	0	0	0	0	4,530017	0,0092243
103634990	LOC103634990	GDT1-like protein 1, chloroplastic	0	0	-2,6444161	0,0314516	-3,057369	0,0163715	0	0	0	0
103641863	FIP1	GEM-like protein 7	0	0	0	0	0	0	0	0	4,5831074	0,0266322
100284455	LOC100284455	germin-like protein subfamily 1 member 11	0	0	0	0	2,2920601	0,0082214	0	0	0	0
100273345	LOC100273345	germin-like protein subfamily 1 member 17	0	0	0	0	0	0	0	0	-2,101354	0,0122507
100285694	LOC100285694	gibberellin 2-oxidase	0	0	0	0	-3,300184	0,0324637	0	0	0	0
100281689	LOC100281689	gibberellin receptor GID1L2	0	0	-2,2576851	0,0042008	0	0	0	0	0	0
100283652	LOC100283652	gibberellin receptor GID1L2	-4,5300974	0,000124	-3,9391284	0,0005633	-3,66689	0,0011868	0	0	0	0
100283864	LOC100283864	gibberellin receptor GID1L2	-3,1196387	0,009357	-2,2657992	0,0365009	0	0	0	0	0	0
100284722	LOC100284722	gibberellin receptor GID1L2	-2,1936496	0,037237	0	0	-2,164637	0,0330717	0	0	0	0
100501987	LOC100501987	gibberellin-regulated protein 2	0	0	0	0	-2,864937	0,0203149	0	0	0	0
103639107	LOC103639107	glucan endo-1,3-beta-glucosidase 1	0	0	0	0	2,1283519	0,0143238	1,80137911	0,026496	0	0
103633264	LOC103633264	glucan endo-1,3-beta-glucosidase 13	0	0	0	0	2,4127153	0,0175048	2,63410319	0,00898	2,3421186	0,0183801
103635596	LOC103635596	glucan endo-1,3-beta-glucosidase 4	0	0	0	0	3,0498625	0,0026283	2,72352438	0,004506	2,2795106	0,0140516
103652987	LOC103652987	glucan endo-1,3-beta-glucosidase 6	0	0	0	0	0	0	0	0	2,1636654	0,039517
100283045	LOC100283045	glucan endo-1,3-beta-glucosidase 7	3,55261051	0,001931	2,5604855	0,0273295	2,4214832	0,0372195	0	0	0	0
100283442	LOC100283442	glucan endo-1,3-beta-glucosidase A6	-3,3233359	0,00052	-3,1769847	0,000845	-3,26525	0,000605	0	0	0	0
100037765	geb1	glucan endo-1,3-beta-glucosidase homolog 1	0	0	-3,2676669	0,0404628	0	0	0	0	0	0
100383765	LOC100383765	glucose-6-phosphate 1-dehydrogenase	0	0	0	0	0	0	0	0	2,2728065	0,025923
103647445	LOC103647445	glutamate receptor 2.9	-3,7184401	0,041522	0	0	0	0	3,89390199	0,028442	0	0
100193482	LOC100193482	Glutamate-1-semialdehyde 21-aminomutase 2 chloroplastic	0	0	0	0	1,900926	0,0092577	2,40360532	0,001039	1,936355	0,0070255
103644324	LOC103644324	glutamic acid-rich protein	0	0	0	0	0	0	1,85753225	0,014432	0	0
542215	gln3	glutamine synthetase 3	0	0	0	0	0	0	0	0	1,9303639	0,0310734
100280064	GATA	Glutamyl-tRNA(Gln) amidotransferase subunit A, chloroplastic/mitochondrial	-2,0868215	0,048246	0	0	0	0	0	0	0	0
100283257	pco114914	Glutathione peroxidase	0	0	1,85236143	0,0081554	1,6540987	0,018409	0	0	0	0
541833	LOC541833	glutathione S-transferase GST 18	0	0	0	0	-1,536645	0,0089235	0	0	0	0
541847	LOC541847	glutathione S-transferase GST 38	0	0	0	0	0	0	-1,830758	0,011197	0	0

			Yuc x Ctr		Azo x Ctr		AzoYuc x Ctr		AzoYuc x Yuc		AzoYuc x Azo	
Gene ID	Gene Symbol	Gene Description	Log2(FC)	pvalue	Log2(FC)	pvalue	Log2(FC)	pvalue	Log2(FC)	pvalue	Log2(FC)	pvalue
541845	gst23	glutathione transferase23	0	0	0	0	1,9819414	0,0021564	0	0	0	0
541841	gst30	glutathione transferase30	0	0	0	0	0	0	-1,7327437	0,02871	0	0
542583	gpn1	glyceraldehyde-3-phosphate deHaseN1	2,94127347	0,049089	3,34614133	0,0230978	0	0	0	0	0	0
100282981	pco070235b	Glyceraldehyde-3-phosphate dehydrogenase, cytosolic	0	0	0	0	-1,501508	0,0342427	0	0	0	0
100383152	LOC100383152	Glycerol-3-phosphate dehydrogenase [NAD( )] 2 chloroplastic	0	0	0	0	4,4303833	0,0100624	3,44759165	0,023223	0	0
100383215	LOC100383215	Glycerophosphodiester phosphodiesterase GDPD2	0	0	0	0	0	0	3,83123902	0,030111	0	0
103626746	LOC103626746	glycerophosphodiester phosphodiesterase GDPDL7	0	0	0	0	0	0	5,68884825	0,002681	3,9651931	0,0252202
103638939	LOC103638939	glycine cleavage system H protein 2, mitochondrial	0	0	-2,0604809	0,0478087	0	0	0	0	0	0
109942791	LOC109942791	glycine, alanine and asparagine-rich protein-like	-3,2964845	0,003943	-2,9436945	0,0064572	-2,629571	0,0124267	0	0	0	0
103645321	LOC103645321	glycine-rich cell wall structural protein 1	-4,9130889	0,022224	-4,4355266	0,0324095	0	0	0	0	0	0
103625906	LOC103625906	glycine-rich cell wall structural protein 1.8	0	0	0	0	3,353871	0,0005134	2,2341285	0,014876	2,8313988	0,0022285
101027139	LOC101027139	glycine-rich cell wall structural protein 2 precursor	0	0	0	0	3,4290573	0,0353824	0	0	0	0
109939896	LOC109939896	glycine-rich cell wall structural protein-like	-2,3320403	0,01227	0	0	0	0	0	0	0	0
109941160	LOC109941160	glycine-rich protein DOT1-like	0	0	0	0	-2,530571	0,0078084	-2,0221764	0,033997	-1,965288	0,0386094
100381912	pco145034	Glycine-rich RNA-binding protein RZ1C	0	0	0	0	-2,01665	0,0019275	0	0	0	0
100383520	LOC100383520	Glycine--tRNA ligase mitochondrial 1	-1,5259451	0,014431	0	0	0	0	0	0	0	0
103632224	LOC103632224	Glycosyl hydrolase family 10 protein	0	0	-1,9394449	0,0161845	0	0	0	0	0	0
100285084	LOC100285084	glycosyltransferase 5	0	0	0	0	-1,760386	0,0378536	-1,7497539	0,036222	-1,805451	0,0294675
100283509	LOC100283509	glycosyltransferase family 28 C-terminal domain containing protein	0	0	0	0	0	0	2,80535538	0,036714	0	0
100194196	LOC100194196	Glycosyltransferase family 61 protein	0	0	0	0	2,0198713	0,0040633	0	0	0	0
100501509	LOC100501509	Glycosyltransferase family 61 protein	0	0	0	0	3,9304005	1,585E-05	3,14174891	0,000334	2,4880164	0,0039716
100280032	IDP1988	glycosyltransferase family protein 28	0	0	0	0	0	0	-2,6831459	0,03318	0	0
100281531	LOC100281531	glyoxalase/bleomycin resistance protein/dioxygenase	0	0	-3,2263457	0,0193557	0	0	0	0	0	0
100193911	LOC100193911	Golgi apparatus membrane protein-like protein ECHIDNA	-2,4151174	0,042337	0	0	0	0	0	0	0	0
100273877	LOC100273877	Got1/Sft2-like vescicle transport protein family	-2,2715741	0,036762	-2,0546651	0,0473705	-2,176032	0,0374829	0	0	0	0

			Yuc x Ctr		Azo x Ctr		AzoYuc x Ctr		AzoYuc x Yuc		AzoYuc x Azo	
Gene ID	Gene Symbol	Gene Description	Log2(FC)	pvalue	Log2(FC)	pvalue	Log2(FC)	pvalue	Log2(FC)	pvalue	Log2(FC)	pvalue
100192076	LOC100192076	GTP binding protein	-3,0708882	0,011112	-2,5904341	0,0275877	-3,038534	0,0108915	0	0	0	0
103626094	LOC103626094	GTPase-activating protein gyp7	0	0	0	0	2,2481968	0,0078785	2,15501447	0,009327	1,8256388	0,0253334
103633061	LOC103633061	GTPase-activating protein gyp7	0	0	0	0	0	0	2,22830453	0,032274	0	0
103647706	LOC103647706	G-type lectin S-receptor-like serine/threonine-protein kinase	-3,3880452	0,045582	0	0	0	0	3,39652892	0,041607	0	0
103633167	LOC103633167	G-type lectin S-receptor-like serine/threonine-protein kinase At2g19130	0	0	0	0	3,4066623	0,0041003	2,20550149	0,032066	2,8421543	0,0071451
103640816	LOC103640816	G-type lectin S-receptor-like serine/threonine-protein kinase At2g19130	0	0	0	0	1,991878	0,0037208	1,54411295	0,017145	0	0
103641631	LOC103641631	G-type lectin S-receptor-like serine/threonine-protein kinase At2g19130	0	0	0	0	3,8426236	0,0144763	0	0	3,4088512	0,014065
100283207	LOC100283207	guanylate kinase	3,12550743	0,043779	3,33175174	0,0301848	0	0	0	0	0	0
103631635	LOC103631635	guanylate-binding protein 1	0	0	0	0	2,1137943	0,0064529	0	0	0	0
100193647	LOC100193647	Haloacid dehalogenase-like hydrolase (HAD) superfamily protein	4,51326403	0,004036	3,6816679	0,0206556	3,9842701	0,0115701	0	0	0	0
103625877	LOC103625877	Haloacid dehalogenase-like hydrolase (HAD) superfamily protein	0	0	0	0	2,6361626	0,0032017	2,27611314	0,009043	2,7224463	0,0019331
103645758	LOC103645758	HBS1-like protein	0	0	1,65100504	0,0272627	0	0	0	0	0	0
100272911	pco094428	Heat shock 70 kDa protein 5	-2,6876858	0,026917	0	0	0	0	0	0	0	0
103652717	LOC103652717	heat shock cognate 70 kDa protein 2	0	0	0	0	2,6142404	0,0291737	5,10004878	0,000321	3,3002404	0,0060587
100280828	LOC100280828	heat shock factor protein HSF30	0	0	3,49423812	0,0305683	0	0	0	0	-3,347088	0,0275022
100283717	LOC100283717	heat shock factor-binding protein 1	2,9329073	0,036569	0	0	0	0	0	0	0	0
100501536	LOC100501536	heat shock protein 1	0	0	0	0	0	0	0	0	-2,598232	0,0009572
103636099	LOC103636099	heat stress transcription factor C-1a	-4,4864983	0,037903	0	0	-4,742778	0,0282007	0	0	0	0
107403160	LOC107403160	Heavy metal transport/detoxification superfamily protein	2,06391555	0,015228	0	0	0	0	0	0	0	0
100280610	LOC100280610	heavy metal-associated domain containing protein	0	0	0	0	0	0	0	0	1,7070318	0,019337
100284161	LOC100284161	heavy metal-associated domain containing protein	0	0	0	0	0	0	-1,5469845	0,010503	0	0
103646336	LOC103646336	heavy metal-associated isoprenylated plant protein 4	0	0	0	0	-4,726178	0,0191336	0	0	0	0
100191593	pco080661a	Hevein-like preproprotein	-2,2996799	0,046549	0	0	0	0	0	0	0	0
100170246	hex2	hexokinase 2	2,50446947	0,005123	0	0	0	0	0	0	0	0
100283177	LOC100283177	hexose carrier protein HEX6	-1,8233455	0,043189	0	0	0	0	1,91304035	0,028218	0	0
778437	NAR2.1	high affinity nitrate transporter	0	0	0	0	1,5414277	0,0246879	0	0	0	0
778428	NAR2.2	high affinity nitrate transporter	0	0	0	0	-3,917765	0,0430684	-4,5252065	0,016278	0	0

			Yuc x Ctr		Azo x Ctr		AzoYuc x Ctr		AzoYuc x Yuc		AzoYuc x Azo	
Gene ID	Gene Symbol	Gene Description	Log2(FC)	pvalue	Log2(FC)	pvalue	Log2(FC)	pvalue	Log2(FC)	pvalue	Log2(FC)	pvalue
103648259	LOC103648259	high mobility group nucleosome-binding domain-containing protein 5-like	0	0	0	0	0	0	0	0	-1,683196	0,0053087
103636218	LOC103636218	high-affinity nitrate transporter 2.3	-2,1680942	0,023598	0	0	0	0	0	0	0	0
100193955	HAK14	high-affinity potassium transporter	0	0	0	0	2,1536249	0,0225711	1,97451809	0,030788	1,8024481	0,0453514
100383808	HAK17	high-affinity potassium transporter	-1,5216718	0,00933	0	0	0	0	0	0	0	0
732835	HK1b2	histidine kinase	2,60559128	0,014427	0	0	0	0	-2,232286	0,027078	0	0
541641	hk2	histidine kinase 2	0	0	0	0	1,6570616	0,0067022	0	0	0	0
101027153	LOC101027153	histone deacetylase 10	0	0	0	0	0	0	1,56061563	0,01654	0	0
100194333	LOC100194333	histone deacetylase complex subunit SAP18	-1,8126232	0,038752	0	0	-1,652583	0,0494523	0	0	0	0
103629514	LOC103629514	histone H3.2	0	0	0	0	2,5013616	0,028707	0	0	3,0903646	0,0067299
103641629	LOC103641629	histone H3.2	0	0	-1,5555005	0,0070405	0	0	0	0	0	0
103650575	LOC103650575	histone H3.2	-1,984232	0,000723	-1,7128624	0,0026968	-2,301657	9,439E-05	0	0	0	0
103627323	LOC103627323	histone H3.3	0	0	0	0	2,0567914	0,0100914	2,02647234	0,010194	2,1524104	0,006272
103633162	LOC103633162	histone H4	0	0	0	0	0	0	0	0	-1,820672	0,0213854
100193014	his2b2	histone2b 2	-2,2597725	0,001459	0	0	-2,379596	0,0007177	0	0	0	0
103632304	LOC103632304	histone-binding protein N1/N2	0	0	0	0	2,6185353	0,0235439	0	0	0	0
100501309	LOC100501309	Histone-lysine N-methyltransferase ATX2	-2,3970377	0,001651	-2,1330968	0,0048676	0	0	0	0	0	0
100857061	LOC100857061	histone-lysine N-methyltransferase ATXR6	0	0	0	0	0	0	0	0	4,7687494	0,0168828
100284915	LOC100284915	histone-lysine N-methyltransferase SUVR3	-3,9480338	0,000769	-3,6328859	0,0015311	-2,651579	0,0171639	0	0	0	0
100382155	LOC100382155	HNH endonuclease	2,65019221	0,041207	0	0	0	0	0	0	0	0
100286155	LOC100286155	holocarboxylase synthetase	0	0	0	0	2,4166739	0,0037405	0	0	0	0
100284049	LOC100284049	homeobox protein HD1 (knox1 (knotted related homeobox1))	0	0	0	0	2,6304448	0,004309	2,15617105	0,017605	2,4251564	0,0076533
100283277	gnarley1	homeobox protein rough sheath 1	0	0	0	0	3,4008287	0,0260135	2,63285339	0,048056	2,587098	0,0473159
100192557	gpm56a	Homeobox-leucine zipper protein ATHB-14	-1,8075979	0,00563	0	0	0	0	0	0	0	0
100272620	cl52289_1b	Homeobox-leucine zipper protein ATHB-6	0	0	0	0	0	0	1,71503921	0,003844	0	0
100285460	LOC100285460	homeobox-leucine zipper protein ATHB-6	0	0	0	0	0	0	0	0	-3,59513	0,0075742
100283939	LOC100283939	homeobox-leucine zipper protein HAT7	0	0	0	0	3,944755	0,0016034	3,55020721	0,00315	3,7994299	0,001613
100194336	si606017c07	homeodomain leucine zipper protein 10	0	0	0	0	1,9541168	0,0342209	2,02036875	0,025933	2,3647681	0,0096057
541873	hmt1	homocysteine S-methyltransferase 1	2,71240879	0,033215	0	0	2,5116381	0,0483214	0	0	0	0

			Yuc x Ctr		Azo x Ctr		AzoYuc x Ctr		AzoYuc x Yuc		AzoYuc x Azo	
Gene ID	Gene Symbol	Gene Description	Log2(FC)	pvalue	Log2(FC)	pvalue	Log2(FC)	pvalue	Log2(FC)	pvalue	Log2(FC)	pvalue
542244	ZAG1	homologue of Arabidopsis gene AGAMOUS	0	0	0	0	0	0	4,16993133	0,046205	0	0
100283888	LOC100283888	HPP	0	0	0	0	0	0	-4,8450707	0,043308	0	0
100383256	LOC100383256	HSP20-like chaperones superfamily protein	0	0	-2,0855316	0,0410095	-2,422291	0,0185787	0	0	0	0
100383840	LOC100383840	HSP20-like chaperones superfamily protein	-2,9396086	0,003868	-2,674186	0,0066956	-2,653634	0,0069123	0	0	0	0
100280228	si707070g05	HSP20-like chaperones superfamily protein	0	0	0	0	0	0	0	0	-1,586002	0,0149848
100501647	LOC100501647	Hsp70-Hsp90 organizing protein 3	0	0	0	0	2,327051	0,0035569	2,19096834	0,005609	2,7591544	0,0005267
100285798	LOC100285798	hsr203J	0	0	0	0	-3,916263	0,0363718	0	0	0	0
100283764	LOC100283764	HVA22-like protein a	-1,7867436	0,048603	0	0	0	0	2,17982481	0,011731	0	0
100216915	LOC100216915	hydrolase	0	0	0	0	2,2125595	0,0487613	2,59748726	0,02059	2,4372112	0,0282918
100281012	LOC100281012	hydrophobic protein LTI6A	0	0	0	0	-1,794693	0,0048008	0	0	0	0
103635163	LOC103635163	hydroxyethylthiazole kinase-like	0	0	0	0	0	0	3,40529966	0,022385	2,7332087	0,0468614
100383538	LOC100383538	hydroxyproline-rich glycoprotein family protein	0	0	0	0	0	0	0	0	1,9503214	0,0155261
100272345	cl11096_1	hypothetical protein	0	0	1,60943158	0,0324811	0	0	0	0	0	0
100191155	cl19610_1(367)	hypothetical protein	-1,5243728	0,049577	-1,8844666	0,0161085	0	0	0	0	0	0
100283833	cl21519_1	hypothetical protein	0	0	0	0	1,8925647	0,0446933	1,82064057	0,049619	0	0
100278579	cl4845_1	hypothetical protein	0	0	0	0	1,8535489	0,0032796	0	0	0	0
100276328	cl50727_1	hypothetical protein	0	0	0	0	0	0	2,62336652	0,033862	0	0
100279889	gpm923	hypothetical protein	2,28623316	0,044299	0	0	0	0	-2,5042049	0,021037	0	0
100192639	IDP96	hypothetical protein	0	0	0	0	1,8810965	0,0153509	0	0	0	0
100191538	LOC100191538	hypothetical protein	-2,8213206	0,025023	-4,388964	0,0028096	-3,183295	0,012343	0	0	0	0
100216763	LOC100216763	hypothetical protein	0	0	-2,4611833	0,0039581	-1,553172	0,0481409	0	0	0	0
100216892	LOC100216892	hypothetical protein	2,61676687	0,01324	0	0	2,8681653	0,0060266	0	0	0	0
100272391	LOC100272391	hypothetical protein	0	0	0	0	2,1084271	0,0418398	2,71746063	0,009606	2,0832629	0,0375471
100272896	LOC100272896	hypothetical protein	-3,7309142	0,000251	-3,3895928	0,0007265	-3,498242	0,0004944	0	0	0	0
100273562	LOC100273562	hypothetical protein	0	0	0	0	0	0	0	0	-1,524446	0,0234395
100274030	LOC100274030	hypothetical protein	-1,9785339	0,001277	-1,6674921	0,005041	-2,194284	0,0003406	0	0	0	0
100274165	LOC100274165	hypothetical protein	0	0	0	0	0	0	4,11951058	0,044455	0	0
100274884	LOC100274884	hypothetical protein	-3,7922785	0,043199	0	0	0	0	0	0	0	0
100274971	LOC100274971	hypothetical protein	0	0	0	0	0	0	0	0	1,8666668	0,0439233
100275155	LOC100275155	hypothetical protein	0	0	0	0	0	0	4,74381422	0,004328	0	0
100275221	LOC100275221	hypothetical protein	-2,1210267	0,026228	-2,8356391	0,0036127	-3,003791	0,0021751	0	0	0	0
100275283	LOC100275283	hypothetical protein	0	0	0	0	2,5856776	0,0191063	2,81272406	0,010225	2,4896346	0,0208406
100275439	LOC100275439	hypothetical protein	0	0	-2,174068	0,0284166	-2,161561	0,0287345	0	0	0	0
100275520	LOC100275520	hypothetical protein	-1,7683254	0,004825	-1,6088012	0,0090283	-1,605291	0,0090084	0	0	0	0
100275726	LOC100275726	hypothetical protein	0	0	0	0	-2,81054	0,0491323	0	0	0	0
100275729	LOC100275729	hypothetical protein	0	0	0	0	2,4168	0,0083241	2,38134808	0,00853	2,4608585	0,006442
100276178	LOC100276178	hypothetical protein	0	0	0	0	2,0996028	0,0286758	2,43093181	0,010767	1,9676029	0,0313841

			Yuc x Ctr		Azo x Ctr		AzoYuc x Ctr		AzoYuc x Yuc		AzoYuc x Azo	
Gene ID	Gene Symbol	Gene Description	Log2(FC)	pvalue	Log2(FC)	pvalue	Log2(FC)	pvalue	Log2(FC)	pvalue	Log2(FC)	pvalue
100276367	LOC100276367	hypothetical protein	0	0	0	0	0	0	-1,6101476	0,032283	0	0
100276607	LOC100276607	hypothetical protein	-4,2288852	0,04456	0	0	0	0	0	0	0	0
100276733	LOC100276733	hypothetical protein	0	0	0	0	0	0	0	0	-1,875528	0,0208117
100276745	LOC100276745	hypothetical protein	0	0	0	0	0	0	2,6746722	0,040844	0	0
100276777	LOC100276777	hypothetical protein	0	0	0	0	-2,235236	0,0047103	0	0	0	0
100276816	LOC100276816	hypothetical protein	0	0	-3,155633	0,0029204	-2,131036	0,0272342	0	0	0	0
100277074	LOC100277074	hypothetical protein	0	0	0	0	0	0	2,66998399	0,01861	0	0
100277571	LOC100277571	hypothetical protein	2,89799328	0,009532	0	0	0	0	-3,288019	0,002185	0	0
100278953	LOC100278953	hypothetical protein	0	0	0	0	0	0	0	0	1,6479673	0,0339778
100279333	LOC100279333	hypothetical protein	0	0	0	0	0	0	0	0	-3,508051	0,0454517
100279742	LOC100279742	hypothetical protein	0	0	-3,7467301	0,0036887	0	0	0	0	0	0
100303943	LOC100303943	hypothetical protein	0	0	0	0	0	0	-1,5811667	0,045809	0	0
100304006	LOC100304006	hypothetical protein	0	0	0	0	3,3013845	0,0014005	2,68142608	0,005855	3,9923557	0,0001199
100304071	LOC100304071	hypothetical protein	1,53333149	0,036121	0	0	0	0	0	0	0	0
100381536	LOC100381536	hypothetical protein	1,85228973	0,01891	2,20465927	0,0047931	0	0	0	0	0	0
100381692	LOC100381692	hypothetical protein	-2,451068	0,018373	0	0	0	0	0	0	0	0
100382145	LOC100382145	hypothetical protein	0	0	0	0	3,8591018	0,0021849	4,24793339	0,000732	4,6973668	0,0002297
100382479	LOC100382479	hypothetical protein	3,23748967	0,036531	0	0	0	0	0	0	0	0
100382728	LOC100382728	hypothetical protein	0	0	0	0	0	0	0	0	2,2842934	0,0001136
100383627	LOC100383627	hypothetical protein	-5,2884542	0,000876	-5,4695809	0,0004425	-3,509587	0,0069114	0	0	0	0
100383644	LOC100383644	hypothetical protein	0	0	0	0	1,8627799	0,0030282	0	0	0	0
100383830	LOC100383830	hypothetical protein	0	0	0	0	1,8860281	0,0073413	2,08014593	0,002966	1,6698588	0,0159637
100384001	LOC100384001	hypothetical protein	0	0	0	0	4,4335369	0,0090826	0	0	0	0
100384566	LOC100384566	hypothetical protein	-4,8708207	0,013503	0	0	0	0	0	0	0	0
100384754	LOC100384754	hypothetical protein	0	0	0	0	-2,534662	0,0317586	0	0	0	0
100501575	LOC100501575	hypothetical protein	0	0	0	0	0	0	2,45100613	0,007022	0	0
100501637	LOC100501637	hypothetical protein	-1,9513714	0,022744	-1,6976203	0,0447892	-1,93873	0,0225424	0	0	0	0
100502151	LOC100502151	hypothetical protein	2,79477837	0,041865	0	0	0	0	0	0	0	0
101202700	LOC101202700	hypothetical protein	0	0	2,30557298	0,0046858	0	0	0	0	-2,282615	0,0040751
103625826	LOC103625826	hypothetical protein	-3,5265423	0,030598	0	0	0	0	3,26986531	0,042736	0	0
103626119	LOC103626119	hypothetical protein	0	0	0	0	0	0	1,81058631	0,009514	2,0894975	0,0028947
103627269	LOC103627269	hypothetical protein	-2,5616849	0,004758	-2,9974346	0,0010348	-2,188423	0,0145391	0	0	0	0
103629308	LOC103629308	hypothetical protein	-1,8758738	0,002536	0	0	0	0	0	0	0	0
103629460	LOC103629460	hypothetical protein	3,3500795	0,048194	3,8145351	0,021993	0	0	0	0	0	0
103630192	LOC103630192	hypothetical protein	-1,7022558	0,016494	0	0	0	0	0	0	0	0
103631691	LOC103631691	hypothetical protein	0	0	-4,5611809	0,0291576	0	0	0	0	0	0
103632763	LOC103632763	hypothetical protein	0	0	0	0	0	0	2,22487292	0,022312	0	0
103633597	LOC103633597	hypothetical protein	0	0	-3,8110553	0,0377122	0	0	0	0	0	0
103638374	LOC103638374	hypothetical protein	0	0	0	0	2,8156823	0,0416493	0	0	0	0
103638588	LOC103638588	hypothetical protein	0	0	1,69674965	0,0171774	0	0	0	0	0	0
103639540	LOC103639540	hypothetical protein	-3,6461325	0,022764	0	0	0	0	0	0	0	0
103640530	LOC103640530	hypothetical protein	0	0	0	0	0	0	-2,4483935	0,049373	0	0

			Yuc x Ctr		Azo x Ctr		AzoYuc x Ctr		AzoYuc x Yuc		AzoYuc x Azo	
Gene ID	Gene Symbol	Gene Description	Log2(FC)	pvalue	Log2(FC)	pvalue	Log2(FC)	pvalue	Log2(FC)	pvalue	Log2(FC)	pvalue
103641799	LOC103641799	hypothetical protein	1,92041093	0,033815	0	0	0	0	0	0	0	0
103647530	LOC103647530	hypothetical protein	0	0	0	0	2,8387137	0,0485486	3,82022475	0,013731	4,0308211	0,0082111
103649946	LOC103649946	hypothetical protein	0	0	0	0	2,8549572	0,0465757	0	0	0	0
103650853	LOC103650853	hypothetical protein	0	0	0	0	2,7488444	0,016451	0	0	2,1542955	0,0458828
103652923	LOC103652923	hypothetical protein	0	0	-3,1836621	0,0405078	0	0	0	0	4,731431	0,0012643
103653202	LOC103653202	hypothetical protein	0	0	2,91058138	0,0362601	0	0	0	0	0	0
103653702	LOC103653702	hypothetical protein	-4,3516393	0,000136	-2,8290251	0,0047964	-3,979243	0,0002096	0	0	0	0
103654538	LOC103654538	hypothetical protein	0	0	-3,3845869	0,0397152	0	0	0	0	0	0
107648858	LOC107648858	hypothetical protein	0	0	0	0	0	0	0	0	-1,553388	0,0444843
4055991	orf105-a	hypothetical protein	0	0	0	0	0	0	0	0	2,4124073	0,0433723
4055918	orf106-a2	hypothetical protein	-4,5218218	0,014635	0	0	0	0	0	0	0	0
4055982	orf129-a	hypothetical protein	0	0	0	0	0	0	1,96463022	0,007049	1,5801907	0,0237169
4055983	orf129-b	hypothetical protein	0	0	0	0	-3,391174	0,026156	0	0	0	0
100382959	pco077498	hypothetical protein	0	0	-3,4841816	0,0333574	0	0	0	0	0	0
100283056	pco116292	hypothetical protein	0	0	0	0	-2,127898	0,0178503	0	0	0	0
100383777	pco117880	hypothetical protein	0	0	0	0	0	0	2,1395797	0,001417	0	0
100194113	pco131351	hypothetical protein	-2,0436649	0,025185	-2,1699282	0,0165391	0	0	0	0	0	0
100274652	pco144937	hypothetical protein	0	0	0	0	0	0	0	0	2,4362774	0,0402598
100276744	si496037e09	hypothetical protein	0	0	0	0	2,8468232	0,0296001	3,8278071	0,005148	0	0
100276145	si605011d10	hypothetical protein	0	0	0	0	0	0	0	0	1,5643159	0,038647
100193834	si618043h03	hypothetical protein	0	0	0	0	-2,056796	0,0194897	0	0	0	0
100276200	si946037c04	hypothetical protein	-1,9541694	0,022057	0	0	0	0	0	0	0	0
100278774	TIDP2682	hypothetical protein	0	0	0	0	1,6428403	0,0046036	0	0	0	0
100275369	TIDP3280	hypothetical protein	-3,2997038	0,030009	0	0	0	0	3,28893456	0,027141	0	0
100274544	TIDP9177	hypothetical protein	2,35910947	0,022563	0	0	0	0	0	0	0	0
1466366	ZemaCp066	hypothetical protein	0	0	-2,3198725	0,0410189	0	0	0	0	0	0
100191375	LOC100191375	IM30 protein-like protein	0	0	0	0	3,2152785	0,0160452	3,07616177	0,017824	2,5368772	0,0451055
100192475	LOC100192475	import inner membrane translocase subunit TIM50	0	0	0	0	3,8703462	0,0473213	0	0	0	0
100279735	umc1153	Inactive leucine-rich repeat receptor-like serine/threonine-protein kinase	0	0	0	0	0	0	0	0	2,9307949	0,0114917
103654264	LOC103654264	inactive poly [ADP-ribose] polymerase RCD1	-1,7127785	0,01358	0	0	-2,045914	0,0034168	0	0	0	0
103642225	LOC103642225	Increased DNA methylation 1	0	0	0	0	0	0	-1,5675344	0,007409	0	0
103629010	LOC103629010	indole-2-monooxygenase-like	-3,525069	0,037974	0	0	0	0	0	0	0	0
103646260	LOC103646260	indole-2-monooxygenase-like	0	0	0	0	0	0	2,08189497	0,006011	0	0
100285511	LOC100285511	indole-3-acetate beta-glucosyltransferase	0	0	0	0	6,5929345	0,0181476	0	0	5,374005	0,0415569
100274566	LOC100274566	Indole-3-glycerol phosphate synthase chloroplastic	0	0	-3,9505356	0,0202867	0	0	0	0	0	0

			Yuc x Ctr		Azo x Ctr		AzoYuc x Ctr		AzoYuc x Yuc		AzoYuc x Azo	
Gene ID	Gene Symbol	Gene Description	Log2(FC)	pvalue	Log2(FC)	pvalue	Log2(FC)	pvalue	Log2(FC)	pvalue	Log2(FC)	pvalue
100282484	pco105901	Inner membrane protease subunit 1, nuclear encoding mitochondrial protein, mRNA	0	0	0	0	0	0	-2,9203824	0,005088	0	0
100282593	LOC100282593	inorganic phosphate cotransporter	-2,3220134	0,039995	0	0	0	0	0	0	0	0
606432	pht1-6	inorganic phosphate transporter 6	-4,1295085	0,030845	0	0	-4,385788	0,0218446	0	0	0	0
100501392	LOC100501392	inosine-5'-monophosphate dehydrogenase	0	0	0	0	2,0174125	0,0124447	1,95285458	0,013317	2,2404864	0,0046675
100280570	LOC100280570	inositol hexaphosphate kinase	0	0	0	0	-2,075534	0,0024102	-2,1053723	0,001882	-2,425748	0,0003053
103652433	LOC103652433	inositol transporter 4-like	0	0	0	0	0	0	-2,5476576	0,022651	-2,601347	0,0190613
100279200	LOC100279200	IRK-interacting protein	0	0	-2,9421808	0,0255526	0	0	0	0	0	0
100282850	LOC100282850	iron-sulfur assembly protein IscA	0	0	0	0	1,8145902	0,0308613	2,56356249	0,003121	2,4394538	0,0036566
103632694	LOC103632694	isoflavone 3'-hydroxylase	-3,9161964	0,041623	0	0	0	0	0	0	0	0
100282269	LOC100282269	kelch motif family protein	0	0	0	0	2,7137467	0,0020176	2,15590583	0,010264	2,5826831	0,0022965
103652836	LOC103652836	keratin, type II cytoskeletal 5	0	0	0	0	0	0	2,65579129	0,031032	2,4248052	0,0459173
103625706	LOC103625706	kinesin-like protein KIN-12G	0	0	0	0	0	0	2,18567111	0,04584	2,1485332	0,0443854
103646141	LOC103646141	kinesin-like protein KIN-7F	0	0	0	0	3,1626976	0,001488	2,74175749	0,004645	4,7591093	5,996E-06
100283504	LOC100283504	KNOX1 domain containing protein	0	0	0	0	2,910741	0,0191182	0	0	2,1904996	0,0404277
103654985	LOC103654985	L10-interacting MYB domain-containing protein-like	-4,8553486	0,005982	-5,0169333	0,0044442	-4,983875	0,0045618	0	0	0	0
103631856	LOC103631856	laccase-14	0	0	0	0	0	0	1,77861794	0,034014	1,7931083	0,0313125
103648173	LOC103648173	laccase-15	0	0	0	0	0	0	-3,2738115	0,006698	0	0
732787	lac4	laccase-like	0	0	0	0	-2,049989	0,022254	0	0	-1,766883	0,0456534
100282507	LOC100282507	lactoylglutathione lyase	0	0	0	0	0	0	-1,9725301	0,044656	0	0
103641118	LOC103641118	lactoylglutathione lyase	0	0	0	0	0	0	2,33305865	0,0279	0	0
103632652	LOC103632652	L-ascorbate oxidase	0	0	0	0	0	0	0	0	2,2572859	0,0328678
103643803	LOC103643803	L-ascorbate oxidase homolog	-1,5828465	0,04267	-1,7621234	0,0240053	-2,412612	0,0024131	0	0	0	0
103644300	LOC103644300	L-aspartate oxidase, chloroplastic	0	0	-3,5414017	0,0103477	0	0	0	0	0	0
100191528	LOC100191528	Late embryogenesis abundant protein group 2	0	0	0	0	2,7686343	0,0008094	2,02781826	0,011412	2,0640544	0,0098378
103650694	LOC103650694	late embryogenesis abundant protein-related / LEA protein-related	0	0	0	0	1,9836545	0,0084208	1,70904215	0,019371	0	0
103652891	LOC103652891	late embryogenesis abundant protein-related / LEA protein-related	-2,0645467	0,027751	0	0	0	0	0	0	0	0
100281565	LOC100281565	LEC14B	0	0	1,64812338	0,0461582	0	0	0	0	0	0
100281445	LOC100281445	lectin-like receptor kinase 7	0	0	-1,6220011	0,0177717	0	0	0	0	0	0
541929	cl2-1	legumin-like protein	1,60264713	0,032699	0	0	0	0	0	0	0	0
100283693	LOC100283693	legumin-like protein	0	0	3,13381419	0,0395594	0	0	0	0	-3,882058	0,0115841
103627183	LOC103627183	leucine aminopeptidase 2, chloroplastic	0	0	0	0	0	0	0	0	1,8744796	0,0076597
103653136	LOC103653136	L-gulonolactone oxidase 2	0	0	0	0	2,3049773	0,0041891	0	0	1,8262644	0,0194228
100284943	LOC100284943	limonoid UDP-glucosyltransferase	-2,7691869	0,000531	-2,7665524	0,0005034	-3,299269	4,274E-05	0	0	0	0

			Yuc x Ctr		Azo x Ctr		AzoYuc x Ctr		AzoYuc x Yuc		AzoYuc x Azo	
Gene ID	Gene Symbol	Gene Description	Log2(FC)	pvalue	Log2(FC)	pvalue	Log2(FC)	pvalue	Log2(FC)	pvalue	Log2(FC)	pvalue
100192094	LOC100192094	lipase	-3,0497354	0,007286	-2,3684028	0,0228934	-2,520598	0,0164262	0	0	0	0
100283980	LOC100283980	lipid binding protein	0	0	0	0	3,2862287	0,001218	2,45691539	0,009776	3,35419	0,0006261
103648254	LOC103648254	lipid transfer protein	-1,8440997	0,030343	0	0	0	0	1,82615729	0,031396	0	0
103626441	LOC103626441	lipid transfer-like protein VAS	0	0	0	0	2,2549416	0,0402267	0	0	0	0
100037829	LOX12	lipoxygenase	0	0	0	0	0	0	1,65746368	0,019419	2,1721667	0,0025565
100279043	LOC100279043	Little protein 1	0	0	0	0	0	0	0	0	-4,580332	0,0150609
103634553	LOC103634553	lon protease homolog, mitochondrial-like	0	0	0	0	2,2350226	0,0162368	0	0	0	0
100274574	si606042h11b	Long-chain-alcohol oxidase FAO4B	-1,5604541	0,02255	0	0	0	0	0	0	0	0
778429	NRT1.2	low affinity nitrate transporter	-2,6714204	0,000892	-2,9170683	0,0002925	-3,436994	2,779E-05	0	0	0	0
100282852	pco090777	Low-molecular-weight cysteine-rich protein LCR70	0	0	0	0	0	0	-1,5739173	0,042822	-1,550934	0,0451967
103630103	LOC103630103	LRR receptor-like serine/threonine-protein kinase	0	0	0	0	-1,645167	0,0083909	0	0	0	0
103654469	LOC103654469	LRR receptor-like serine/threonine-protein kinase	0	0	0	0	-1,741123	0,0001118	0	0	0	0
100193598	pco069596	LRR receptor-like serine/threonine-protein kinase ERECTA	0	0	0	0	-1,666669	0,0042429	-1,5549645	0,006854	0	0
103630101	LOC103630101	LRR receptor-like serine/threonine-protein kinase GSO1	-2,7773543	0,000629	-3,3340888	4,863E-05	-2,972367	0,0002481	0	0	0	0
100282349	LOC100282349	LSM7-like	3,68112702	0,003424	0	0	0	0	-3,3119275	0,003877	0	0
100285982	LOC100285982	LSM7-like	0	0	0	0	0	0	1,64098319	0,037501	1,9991181	0,0116114
103630002	LOC103630002	L-type lectin-domain containing receptor kinase IV.1	0	0	4,4370419	0,0242593	0	0	0	0	-5,116048	0,0093832
103642561	LOC103642561	L-type lectin-domain containing receptor kinase IV.1	0	0	0	0	0	0	0	0	2,4049721	0,0420394
103647759	LOC103647759	L-type lectin-domain containing receptor kinase IX.1	-4,2502839	0,015702	0	0	0	0	4,31020223	0,012682	0	0
100192853	LOC100192853	lustrin A	0	0	0	0	0	0	0	0	4,8574103	0,0140531
100276651	LOC100276651	LYR motif-containing protein At3g19508	0	0	0	0	0	0	2,74048877	0,034128	0	0
541648	lkrsdh1	lysine-ketoglutarate reductase/saccharopine dehydrogenase 1	0	0	0	0	0	0	0	0	-1,603924	0,0167369
103626493	LOC103626493	lysine-specific demethylase 5B	0	0	0	0	1,7592754	0,0095322	1,9285668	0,00424	1,9509716	0,0036872
109939882	LOC109939882	lysosomal Pro-X carboxypeptidase	0	0	3,93390185	0,0257809	0	0	0	0	0	0
542044	m26	m26 protein	0	0	0	0	3,6241801	0,0424426	0	0	0	0
542041	m4	m4 protein	0	0	0	0	0	0	3,3021242	0,007121	0	0
100192049	LOC100192049	MACPF domain-containing protein	0	0	0	0	2,5614407	0,0453064	0	0	0	0
100383384	LOC100383384	MACPF domain-containing protein	0	0	0	0	3,3214404	0,0001584	2,63707345	0,001838	2,7146374	0,0013044
542042	mads1	MADS1	0	0	0	0	0	0	1,91628996	0,019445	0	0

			Yuc x Ctr		Azo x Ctr		AzoYuc x Ctr		AzoYuc x Yuc		AzoYuc x Azo	
Gene ID	Gene Symbol	Gene Description	Log2(FC)	pvalue	Log2(FC)	pvalue	Log2(FC)	pvalue	Log2(FC)	pvalue	Log2(FC)	pvalue
542019	zmm16	MADS16	-2,7541998	0,001191	-2,3707923	0,0039752	-2,814191	0,0008059	0	0	0	0
100415939	MADS9	MADS-domain transcription factor	4,04335306	0,035395	0	0	0	0	0	0	0	0
100501814	LOC100501814	Major facilitator superfamily protein	0	0	0	0	1,8334831	0,0062688	1,69659659	0,008363	0	0
100283525	cl9362_1	MAK16-like protein RBM13	-4,4828164	8,68E-05	-4,2551907	0,0001766	-4,057987	0,000332	0	0	0	0
542252	mas1	malate synthase 1	1,73789507	0,003187	0	0	0	0	0	0	0	0
100272437	LOC100272437	MAP3K-like protein kinase	0	0	0	0	-1,843742	0,0357083	0	0	-2,644449	0,0020907
103651325	LOC103651325	MAP7 domain-containing protein 1	0	0	0	0	0	0	-4,0581898	0,034143	0	0
100276832	LOC100276832	maternal effect embryo arrest 60	0	0	0	0	-2,983956	0,0280141	0	0	0	0
103646094	LOC103646094	MDIS1-interacting receptor like kinase 2	0	0	0	0	0	0	0	0	2,5530146	0,0154134
100274258	MED7.2	MED7 transcription factor	0	0	3,9929861	0,0179216	4,3771097	0,0089506	0	0	0	0
100279223	LOC100279223	Mediator of RNA polymerase II transcription subunit 11	3,70421462	0,038219	0	0	0	0	0	0	0	0
103643172	LOC103643172	mediator of RNA polymerase II transcription subunit 15a	0	0	0	0	0	0	1,82026462	0,010451	1,8956355	0,007413
100274560	LOC100274560	Mediator of RNA polymerase II transcription subunit 27	0	0	0	0	0	0	0	0	2,1871331	0,0397932
100277277	LOC100277277	Mediator-associated protein 2	0	0	-1,8566314	0,0266302	0	0	0	0	0	0
109940583	LOC109940583	meiotic recombination protein DMC1 homolog	0	0	2,35798357	0,0450174	0	0	0	0	-2,996821	0,0107385
100275316	IDP2596	Membrane magnesium transporter	0	0	0	0	0	0	-1,9539852	0,044266	0	0
100274505	LOC100274505	membrane protein	0	0	0	0	0	0	1,845534	0,005606	0	0
100282365	LOC100282365	membrane protein	0	0	0	0	1,5259602	0,0081363	0	0	0	0
100283507	LOC100283507	membrane protein	0	0	0	0	-1,65053	0,012601	0	0	0	0
103648033	LOC103648033	metal transporter Nramp4	0	0	0	0	0	0	2,06747193	0,018368	0	0
100280443	LOC100280443	metal transporter Nramp5	0	0	0	0	1,5433013	0,0101198	0	0	0	0
100383923	LOC100383923	methyl binding domain123	4,76691224	0,003165	0	0	0	0	-5,3382878	0,000928	0	0
109940248	LOC109940248	methyl-CpG-binding domain protein 2-like	-3,2647098	0,008269	-2,2183715	0,0455821	0	0	3,11057369	0,011273	0	0
100274929	LOC100274929	Methyltransferase-related protein	0	0	0	0	0	0	0	0	1,9651652	0,0377755
100283333	LOC100283333	MFP1 attachment factor 1	0	0	-2,4424283	0,0185721	-2,417747	0,0192545	0	0	0	0
103628686	LOC103628686	MFP1 attachment factor 1-like	-1,906747	0,047775	-2,8545513	0,0059916	0	0	0	0	2,1402546	0,0396077
103318213	MIR166h	microRNA MIR166h	0	0	0	0	-2,441425	0,035183	0	0	0	0
100191316	LOC100191316	Mitochondrial arginine transporter BAC1	-1,6368941	0,04291	0	0	-1,588674	0,0469428	0	0	0	0
100284060	LOC100284060	mitochondrial carrier protein CGI-69	3,18421609	0,011801	0	0	0	0	-3,2545099	0,008242	0	0
100285900	pco107818	Mitochondrial import inner membrane translocase subunit Tim10	0	0	0	0	-3,20704	0,0192254	-3,5781107	0,007631	-3,228471	0,0164094
100282065	LOC100282065	mitochondrial import inner membrane translocase subunit TIM14	-3,2389618	0,03975	-4,0427509	0,0137679	-3,542022	0,0249565	0	0	0	0

			Yuc x Ctr		Azo x Ctr		AzoYuc x Ctr		AzoYuc x Yuc		AzoYuc x Azo	
Gene ID	Gene Symbol	Gene Description	Log2(FC)	pvalue	Log2(FC)	pvalue	Log2(FC)	pvalue	Log2(FC)	pvalue	Log2(FC)	pvalue
103629105	LOC103629105	mitochondrial import inner membrane translocase subunit TIM22-2	0	0	0	0	3,9572133	0,0408568	0	0	0	0
100193036	cl31945_1	Mitochondrial import inner membrane translocase subunit TIM44-2	-1,7375592	0,040296	0	0	0	0	0	0	0	0
100283857	LOC100283857	mitochondrial NADH ubiquinone oxidoreductase 29 kDa subunit	0	0	0	0	0	0	1,50061591	0,007758	0	0
100282481	LOC100282481	Mitochondrial substrate carrier family protein	0	0	0	0	3,5660953	0,0154971	3,20910627	0,019651	2,6528393	0,0438354
100284813	pco141217	Mitochondrial substrate carrier family protein	3,07489759	0,043972	3,60087553	0,0166667	0	0	0	0	0	0
100193567	si606070a01a	Mitochondrial substrate carrier family protein	0	0	0	0	2,7686498	0,0413818	0	0	0	0
103650800	LOC103650800	mitochondrial thiamine diphosphate carrier 2-like	0	0	0	0	0	0	2,31550187	0,007762	0	0
109940255	LOC109940255	mitogen-activated protein kinase 8-like	0	0	0	0	0	0	1,87983327	0,032792	0	0
103627282	LOC103627282	mitogen-activated protein kinase kinase kinase 3	0	0	0	0	2,9910697	0,0038413	3,00523282	0,002765	2,0012258	0,0348488
100281486	LOC100281486	MKK4 - putative MAPKK	-3,0992411	0,005524	-4,1022465	0,0004348	-4,443488	0,0001927	0	0	0	0
103643796	LOC103643796	MLO-like protein 4	0	0	0	0	-1,885307	0,0449303	0	0	0	0
100502388	LOC100502388	MOB kinase activator-like 1B	0	0	-1,6095614	0,0306471	-1,50881	0,0418323	0	0	0	0
103629655	LOC103629655	molybdenum cofactor sulfurase	0	0	0	0	0	0	1,70245912	0,016071	0	0
100284923	umc2551	Molybdopterin synthase catalytic subunit	-2,0145171	0,019751	0	0	0	0	0	0	0	0
103633384	LOC103633384	momilactone A synthase	0	0	0	0	2,4751021	0,0063924	2,01052462	0,021314	0	0
100279805	uaz204	Monodehydroascorbate reductase 4 peroxisomal	0	0	0	0	2,833097	0,0010177	1,85218088	0,018858	0	0
103649242	LOC103649242	monothiol glutaredoxin-S11	0	0	0	0	-2,330285	0,048684	0	0	0	0
109942117	LOC109942117	mRNA decay activator protein ZFP36L3-like	0	0	-3,1686616	0,0433563	0	0	0	0	0	0
100286299	LOC100286299	MTA/SAH nucleosidase	0	0	0	0	1,5090174	0,0494017	2,04178943	0,008039	1,6120813	0,0338597
100285204	LOC100285204	mTERF family protein	0	0	0	0	5,1495947	0,0048757	4,66216031	0,006998	4,0846509	0,0106269
100285943	LOC100285943	mTERF family protein	0	0	0	0	4,3296123	0,0023376	3,28525072	0,011365	4,5810603	0,0008898
100192602	LOC100192602	mtN3-like protein	0	0	2,11106916	0,0335576	0	0	0	0	0	0
100285320	LOC100285320	multicystatin	1,57785298	0,036733	0	0	0	0	0	0	0	0
100217212	LOC100217212	Multifunctional methyltransferase subunit TRM112-like protein	0	0	0	0	0	0	2,41102589	0,013561	2,185154	0,023315
100273260	LOC100273260	MYB transcription factor	0	0	0	0	-1,790235	0,0110213	-1,8291897	0,009329	-2,286047	0,001146
100191731	LOC100191731	MYBGA transcription factor	0	0	0	0	0	0	0	0	-2,09982	0,042064
103635903	LOC103635903	myb-like protein J	-5,2671969	0,020162	-5,4931296	0,0153938	0	0	0	0	0	0
109944229	LOC109944229	myc-associated zinc finger protein-like	0	0	0	0	0	0	1,90200071	0,041475	1,9441103	0,0336958
100273576	LOC100273576	Myosin heavy chain-related protein	0	0	0	0	2,1355736	0,0332232	1,9651697	0,041207	2,2624353	0,0193988

			Yuc x Ctr		Azo x Ctr		AzoYuc x Ctr		AzoYuc x Yuc		AzoYuc x Azo	
Gene ID	Gene Symbol	Gene Description	Log2(FC)	pvalue	Log2(FC)	pvalue	Log2(FC)	pvalue	Log2(FC)	pvalue	Log2(FC)	pvalue
103651836	LOC103651836	myosin-1-like	0	0	1,97030003	0,0424275	1,9302928	0,0467271	0	0	0	0
103655625	LOC103655625	myosin-1-like	-1,6885818	0,008604	0	0	0	0	1,87397198	0,002545	0	0
100191480	cl5992_1b	Myosin-binding protein 7	0	0	0	0	0	0	2,41684133	0,038023	0	0
100281380	NAC40	NAC domain protein NAC5	1,70587649	0,00755	1,60438089	0,0118297	1,7607977	0,0055353	0	0	0	0
107275227	LOC107275227	NAC domain-containing protein 18	0	0	0	0	0	0	3,1840955	0,027983	0	0
103650490	LOC103650490	NAC domain-containing protein 21/22	0	0	0	0	0	0	2,11959622	0,028805	0	0
100285036	cl9439_1	NAD(P)-binding Rossmann-fold superfamily protein	0	0	0	0	5,0709816	0,0002187	4,43302424	0,00044	4,400595	0,0004077
100193896	LOC100193896	NAD(P)-binding Rossmann-fold superfamily protein	0	0	0	0	1,9156864	0,0317411	0	0	0	0
100191925	umc2667	NAD(P)-binding Rossmann-fold superfamily protein	0	0	0	0	0	0	2,40981389	0,033392	2,2146207	0,0462718
109940833	LOC109940833	NAD(P)H-quinone oxidoreductase subunit 5, chloroplastic-like	-1,6256037	0,007386	-3,2382373	9,9E-08	-3,308836	5,186E-08	-1,6832305	0,005632	0	0
7804324	nad1_1	NADH dehydrogenase subunit 1.1	0	0	1,54095674	0,0039204	1,6397241	0,0020941	0	0	0	0
845186	ndhG	NADH dehydrogenase subunit 6	0	0	0	0	0	0	3,92272007	0,022293	0	0
100283792	LOC100283792	NADH-ubiquinone oxidoreductase 10.5 kDa subunit	2,54298013	0,002268	2,65507018	0,0013166	2,2959648	0,005839	0	0	0	0
542233	me3	NADP malic enzyme 3	0	0	0	0	2,7374173	0,035138	2,49902309	0,040616	2,9111732	0,0189177
109946101	LOC109946101	NADPH:adrenodoxin oxidoreductase, mitochondrial-like	0	0	0	0	0	0	-3,3524965	0,021955	0	0
103641626	LOC103641626	NDR1/HIN1-like 1	0	0	0	0	0	0	4,01379402	0,041517	0	0
103642212	LOC103642212	NDR1/HIN1-like protein 2	-2,3906986	0,01251	0	0	0	0	0	0	0	0
100500945	LOC100500945	necrotic4	0	0	0	0	2,8391174	0,0392377	0	0	0	0
103644329	LOC103644329	neurofilament heavy polypeptide	0	0	0	0	0	0	0	0	1,5468795	0,0350583
103634581	LOC103634581	neutral/alkaline invertase 1, mitochondrial	0	0	-1,8380835	0,0288676	0	0	0	0	0	0
103650144	LOC103650144	neutral/alkaline invertase 1, mitochondrial	0	0	0	0	2,7031215	0,0071812	2,08510946	0,031507	2,2301176	0,0213019
100283700	LOC100283700	NF-180	0	0	-1,7156966	0,0197942	-1,616702	0,0269133	0	0	0	0
542480	nas1	nicotianamine synthase 1	0	0	0	0	0	0	0	0	-1,935062	0,0240159
109942391	LOC109942391	nicotianamine synthase 2	0	0	0	0	0	0	0	0	-2,155247	0,0222275
103653736	LOC103653736	nicotinamidase 1	3,8339894	0,013575	3,48648464	0,0253729	3,7590467	0,0150292	0	0	0	0
100274271	pco103153	Nicotinate phosphoribosyltransferase 2	-2,2069678	0,003176	-1,8170363	0,0145093	-2,508553	0,000805	0	0	0	0
103636411	LOC103636411	ninja-family protein 6-like	0	0	1,71697898	0,0343428	0	0	0	0	0	0
542092	nrt2	nitrate transport 2	0	0	0	0	0	0	2,26660473	0,01045	2,4858761	0,0050162
100280214	cl36905_1b	Nitrate-induced NOI protein	0	0	0	0	2,4218664	0,0234058	0	0	0	0
100193184	LOC100193184	nitrate-induced NOI protein	0	0	1,88133616	0,0108306	1,9490537	0,0081581	0	0	0	0
103636180	LOC103636180	nodulation-signaling pathway 1 protein	0	0	0	0	2,6453396	0,024755	0	0	0	0

			Yuc x Ctr		Azo x Ctr		AzoYuc x Ctr		AzoYuc x Yuc		AzoYuc x Azo	
Gene ID	Gene Symbol	Gene Description	Log2(FC)	pvalue	Log2(FC)	pvalue	Log2(FC)	pvalue	Log2(FC)	pvalue	Log2(FC)	pvalue
100384237	LOC100384237	Nodulin homeobox	0	0	0	0	0	0	2,49358869	0,01825	0	0
103651248	LOC103651248	non-classical arabinogalactan protein 31	0	0	0	0	2,1068778	0,0387163	2,18151023	0,029935	2,4447208	0,0152387
100276627	LOC100276627	non-specific lipid transfer protein-like 1	0	0	0	0	0	0	0	0	-1,646719	0,0283617
103647871	LOC103647871	non-specific lipid transfer protein-like 1	0	0	0	0	-3,123244	0,0441963	0	0	0	0
100284199	LOC100284199	nonspecific lipid-transfer protein	0	0	0	0	1,9937302	0,0072897	2,23233041	0,002626	0	0
100274469	umc1594	Non-specific lipid-transfer protein	5,48868432	0,004879	0	0	0	0	0	0	0	0
100285645	LOC100285645	nonspecific lipid-transfer protein AKCS9	-2,1056026	0,048498	0	0	0	0	0	0	0	0
103640483	LOC103640483	non-specific lipid-transfer protein-like protein At5g64080	0	0	0	0	0	0	1,85645423	0,018765	1,9730139	0,0120892
100381918	LOC100381918	Non-structural maintenance of chromosomes element 4 homolog A	-2,8079196	0,020442	0	0	0	0	0	0	0	0
103641036	LOC103641036	noroxomaritidine synthase	0	0	0	0	0	0	3,3531633	0,029717	3,8316437	0,0154298
100193537	LOC100193537	nramp aluminum transporter1	0	0	0	0	0	0	0	0	1,9807791	0,0049871
100216910	LOC100216910	Nuclear pore complex protein NUP35	-1,6102588	0,039376	0	0	-2,068821	0,009617	0	0	-1,650589	0,0372158
103634826	LOC103634826	nuclear pore complex protein NUP93A	2,08002603	0,005519	0	0	0	0	0	0	0	0
100274945	pco103628	Nuclear speckle RNA-binding protein A	0	0	0	0	0	0	2,20749391	0,020123	2,1370722	0,0234333
100284408	LOC100284408	nuclear transcription factor Y subunit A-10	0	0	-6,1491601	3,697E-05	-3,727828	0,0024809	-2,6811397	0,032039	0	0
100282695	LOC100282695	nucleic acid binding protein	0	0	0	0	-1,940961	0,0045249	0	0	-1,869006	0,005505
103644287	LOC103644287	Nucleic acid-binding proteins superfamily	0	0	0	0	0	0	-3,2307851	0,012042	0	0
100272944	TIDP3720	Nucleobase-ascorbate transporter 6	0	0	0	0	0	0	0	0	-1,552151	0,0116221
100285187	LOC100285187	nucleolar protein,Nop52 containing protein	0	0	0	0	2,1660526	0,0471275	0	0	0	0
100278385	BE518809	nucleoporin-related	0	0	-1,7597604	0,0409527	0	0	0	0	0	0
100282231	pco154542(637)	Nucleoside N-ribohydrolase 3	0	0	0	0	-1,773353	0,0014179	0	0	0	0
100285127	LOC100285127	nucleoside transporter	0	0	0	0	2,0144567	0,0166075	2,16936844	0,008421	0	0
100280725	LOC100280725	nucleotide binding protein	-2,3734328	0,010296	-2,9831554	0,0014422	0	0	0	0	0	0
100170249	BT2A	nucleotide sugar translocator BT2A	0	0	0	0	5,0321672	0,0027134	3,33063353	0,015018	0	0
100194181	LOC100194181	Nucleotide-diphospho-sugar transferase family protein	0	0	0	0	-4,53636	0,0034681	-4,5747193	0,002776	-4,653121	0,0021763
100272585	LOC100272585	Nudix hydrolase 23 chloroplastic	0	0	0	0	0	0	0	0	-2,380178	0,0285693
100277138	LOC100277138	OBP3-responsive gene 4	0	0	0	0	0	0	0	0	-2,994949	0,0197254

			Yuc x Ctr		Azo x Ctr		AzoYuc x Ctr		AzoYuc x Yuc		AzoYuc x Azo	
Gene ID	Gene Symbol	Gene Description	Log2(FC)	pvalue	Log2(FC)	pvalue	Log2(FC)	pvalue	Log2(FC)	pvalue	Log2(FC)	pvalue
103632340	LOC103632340	Octicosapeptide/Phox/Bem1p (PB1) domain-containing protein / tetratricopeptide repeat (TPR)-containing protein	0	0	0	0	3,6874374	0,0204079	0	0	3,0332629	0,0284143
542407	obf3.2	octopine synthase binding factor 3	-1,8650721	0,035245	0	0	0	0	0	0	0	0
100192765	gpm566c	O-fucosyltransferase family protein	0	0	0	0	-2,075413	0,0217942	0	0	0	0
100382271	LOC100382271	O-fucosyltransferase family protein	0	0	0	0	0	0	0	0	-2,289068	0,0261218
103651025	LOC103651025	O-fucosyltransferase family protein	0	0	0	0	2,1125102	0,0225724	0	0	0	0
100193995	LOC100193995	oligomeric Golgi complex component-related protein	0	0	0	0	2,7117915	1,56E-05	1,57532807	0,006098	0	0
103642159	LOC103642159	oligouridylate-binding protein 1	0	0	0	0	2,5980013	0,0041913	2,41672311	0,007004	1,9345084	0,0293759
100282744	LOC100282744	omega-6 fatty acid desaturase, endoplasmic reticulum isozyme 2	0	0	1,91470596	0,0061622	0	0	0	0	0	0
103630606	LOC103630606	O-methyltransferase ZRP4-like	0	0	0	0	-2,528508	0,0409583	-3,399147	0,004561	0	0
100284956	CNR01	ORFX protein	0	0	0	0	0	0	-1,8402166	0,010496	0	0
103641920	LOC103641920	organelle RRM domain-containing protein 6, chloroplastic	0	0	0	0	0	0	4,41576951	0,008113	0	0
541938	orc2	origin recognition complex subunit 2	0	0	0	0	3,0494229	0,0366517	3,39889193	0,018177	0	0
103631786	LOC103631786	Os03g0176600-like protein	0	0	-4,4210504	0,0219819	0	0	0	0	0	0
107305673	LOC107305673	Os12g0146300-like protein	0	0	0	0	2,202021	0,0354246	2,07411679	0,041275	2,2090888	0,029262
103642055	LOC103642055	OSJNBa0008M17.14-like protein	0	0	0	0	0	0	0	0	-3,249441	0,0120598
103634932	LOC103634932	OSJNBa0089E12.13-like protein	0	0	-6,0214096	0,0083012	-6,0124	0,0082004	0	0	0	0
100277241	LOC100277241	OSJNBb0048E02.16-like protein	0	0	0	0	3,7994823	0,0385466	0	0	0	0
100280707	LOC100280707	osmotin-like protein	0	0	0	0	0	0	0	0	-2,829758	0,0234489
100276695	LOC100276695	Osmotin-like protein OSM34	0	0	0	0	0	0	3,59637375	0,015458	2,9065456	0,0409659
100285862	LOC100285862	O-succinylhomoserine sulfhydrylase	0	0	0	0	0	0	0	0	3,2993288	0,0113448
103645785	LOC103645785	OTU domain-containing protein 3	3,37989259	0,035096	3,40327401	0,0324586	3,4288474	0,0308279	0	0	0	0
100382679	LOC100382679	Outer envelope pore protein 24A chloroplastic	-4,1553414	0,005118	0	0	-3,002885	0,0150343	0	0	0	0
100191859	LOC100191859	Outer envelope protein 80 chloroplastic	0	0	0	0	0	0	-2,6255667	0,042972	0	0
100216624	LOC100216624	Outer membrane OMP85 family protein	0	0	0	0	0	0	0	0	2,5702648	0,020429
100280941	LOC100280941	outer membrane protein, OMP85 family protein	0	0	0	0	1,991886	0,0382859	0	0	0	0
606477	LOC606477	outward rectifying potassium channel 1	0	0	0	0	0	0	-1,6933461	0,008089	0	0
103636394	LOC103636394	oxalate--CoA ligase	-3,6923188	9,86E-05	-3,2700713	0,0004354	-3,703225	8,152E-05	0	0	0	0
100281254	LOC100281254	oxidoreductase	-2,1060236	0,039363	0	0	0	0	0	0	0	0
100286195	LOC100286195	oxidoreductase	0	0	0	0	1,8111685	0,0263375	0	0	0	0
542560	pco103506(726)	oxo-glutarate/malate transporter1	2,04386473	0,019806	0	0	0	0	-2,0364076	0,016138	0	0

			Yuc x Ctr		Azo x Ctr		AzoYuc x Ctr		AzoYuc x Yuc		AzoYuc x Azo	
Gene ID	Gene Symbol	Gene Description	Log2(FC)	pvalue	Log2(FC)	pvalue	Log2(FC)	pvalue	Log2(FC)	pvalue	Log2(FC)	pvalue
100217187	umc1964	oxysterol-binding protein OBPa	0	0	2,74771083	0,0016426	0	0	0	0	-3,029291	0,0004113
100216902	LOC100216902	palmitoyl-protein thioesterase 1	0	0	0	0	1,8265556	0,0338551	0	0	0	0
100282464	LOC100282464	palmitoyltransferase ZDHHC20	0	0	0	0	0	0	1,69644757	0,007987	0	0
100192016	LOC100192016	PAN domain-containing protein	-3,6489945	0,038914	0	0	0	0	0	0	0	0
103627233	LOC103627233	PAN domain-containing protein	0	0	0	0	0	0	2,76977814	0,016566	2,6260014	0,0195651
103630956	LOC103630956	PAN domain-containing protein At5g03700	0	0	0	0	0	0	1,83108497	0,023917	0	0
103645278	LOC103645278	PAN domain-containing protein At5g03700	0	0	0	0	0	0	0	0	4,4271372	0,0393024
100304391	pco079591	PAP/OAS1 substrate-binding domain superfamily	0	0	3,09378147	0,0479633	0	0	0	0	0	0
103639615	LOC103639615	paramyosin	0	0	0	0	0	0	1,5816094	0,033131	1,5615168	0,0343713
109942796	LOC109942796	paraneoplastic antigen Ma6E-like	0	0	0	0	0	0	2,62443349	0,013015	0	0
100283335	LOC100283335	partner of Nob1	4,16038892	0,011716	0	0	5,2036399	0,0013336	0	0	3,4538573	0,0134428
100273358	LOC100273358	partner of Y14-MAGO	0	0	0	0	2,5848685	0,0235128	0	0	0	0
100272901	LOC100272901	Patatin-like protein 2	0	0	0	0	0	0	-2,5834293	0,044694	0	0
100284065	LOC100284065	patatin-like protein 3	0	0	0	0	1,7013112	0,0249316	1,59096745	0,032606	0	0
103649554	LOC103649554	pathogenesis-related genes transcriptional activator PTI6	-1,5448923	0,045044	0	0	0	0	0	0	0	0
103646628	LOC103646628	pathogenesis-related protein PR-1 type	-3,5114019	0,007048	-4,0183202	0,0023295	-4,303808	0,0012618	0	0	0	0
100284353	TIDP3734	Pathogenicity protein PATH531-like protein	1,89635011	0,029862	0	0	0	0	0	0	0	0
100285321	LOC100285321	pathogen-related protein	1,81115453	0,002958	0	0	0	0	0	0	0	0
107548106	LOC107548106	pco080605(66)	0	0	0	0	0	0	1,88529924	0,044514	0	0
100277572	pco108839	pco108839(627)	0	0	0	0	-3,103632	0,0030675	0	0	-2,144973	0,0427614
100279044	LOC100279044	PEBP (phosphatidylethanolamine-binding protein) family protein	0	0	0	0	0	0	0	0	-4,362375	0,0343863
100274042	pco091441	Pectin acetylesterase 12	0	0	0	0	3,2289864	0,0011089	2,60791739	0,007168	3,4623406	0,0004185
100274158	LOC100274158	Pectin acetylesterase 5	0	0	-3,8052113	0,0053096	-3,634332	0,0070698	0	0	0	0
103637955	LOC103637955	pectinesterase	0	0	3,81765522	0,041972	0	0	0	0	0	0
100283750	LOC100283750	pectinesterase inhibitor domain containing protein	0	0	0	0	0	0	4,51382229	0,01817	0	0
100281178	LOC100281178	pectinesterase PPE8B	0	0	3,00209644	0,0483716	0	0	0	0	0	0
103632574	LOC103632574	pentatricopeptide repeat-containing protein	0	0	3,0385927	0,0145252	0	0	3,54700899	0,01392	0	0
103648257	LOC103648257	pentatricopeptide repeat-containing protein	-3,7756121	0,037209	0	0	0	0	0	0	0	0
103626085	LOC103626085	pentatricopeptide repeat-containing protein At1g09900	0	0	0	0	-4,090668	0,0159985	0	0	0	0

			Yuc x Ctr		Azo x Ctr		AzoYuc x Ctr		AzoYuc x Yuc		AzoYuc x Azo	
Gene ID	Gene Symbol	Gene Description	Log2(FC)	pvalue	Log2(FC)	pvalue	Log2(FC)	pvalue	Log2(FC)	pvalue	Log2(FC)	pvalue
103644172	LOC103644172	pentatricopeptide repeat-containing protein At1g09900	3,74287676	0,023694	0	0	0	0	0	0	0	0
103647847	LOC103647847	pentatricopeptide repeat-containing protein At1g63070, mitochondrial	0	0	0	0	3,0241353	0,0070473	0	0	2,5507524	0,0151327
103633614	LOC103633614	pentatricopeptide repeat-containing protein At1g74850, chloroplastic-like	0	0	0	0	1,6775251	0,0237189	0	0	0	0
111590272	LOC111590272	pentatricopeptide repeat-containing protein At2g17670-like	-2,1123752	0,020032	0	0	0	0	0	0	0	0
103633771	LOC103633771	pentatricopeptide repeat-containing protein At2g36240	-3,0103644	0,012459	0	0	0	0	0	0	0	0
103639844	LOC103639844	pentatricopeptide repeat-containing protein At3g16010	-3,7857423	0,010538	-4,4791156	0,0028542	-3,467886	0,0177745	0	0	0	0
109942253	LOC109942253	pentatricopeptide repeat-containing protein At3g16010	-5,8946793	0,01283	-5,2701181	0,0227246	-6,247193	0,0086077	0	0	0	0
109944852	LOC109944852	pentatricopeptide repeat-containing protein At3g48250, chloroplastic	3,51271942	0,006853	2,71198013	0,0387884	2,9343976	0,0246132	0	0	0	0
100274763	LOC100274763	pentatricopeptide repeat-containing protein At4g13650	0	0	0	0	2,645667	0,0137548	2,27294572	0,019159	0	0
103641737	LOC103641737	pentatricopeptide repeat-containing protein At4g39952, mitochondrial	0	0	-3,1093559	0,0302926	0	0	0	0	0	0
103638071	LOC103638071	pentatricopeptide repeat-containing protein At5g39710	0	0	-2,7046898	0,0456655	0	0	0	0	3,0619038	0,018796
103630024	LOC103630024	pentatricopeptide repeat-containing protein At5g48730, chloroplastic	-5,5754285	3,79E-05	-6,3406567	3,587E-06	-6,402718	2,871E-06	0	0	0	0
103651237	LOC103651237	pentatricopeptide repeat-containing protein At5g50280, chloroplastic	0	0	0	0	-3,383138	0,0130577	-3,1155981	0,021675	-2,731732	0,0451793
103637407	LOC103637407	pentatricopeptide repeat-containing protein At5g64320, mitochondrial	0	0	0	0	4,6608983	0,046495	6,19085633	0,011246	5,1087429	0,0276443
100383543	LOC100383543	Pentatricopeptide repeat-containing protein chloroplastic	0	0	3,93674863	0,0186527	0	0	0	0	0	0
103639281	LOC103639281	pentatricopeptide repeat-containing protein, chloroplastic-like	0	0	0	0	0	0	2,15254516	0,021088	0	0
103633393	LOC103633393	pentatricopeptide repeat-containing protein, mitochondrial-like	2,94710347	0,007627	0	0	0	0	-2,6871991	0,01123	0	0
103642182	LOC103642182	pentatricopeptide repeat-containing protein, mitochondrial-like	-3,3733992	0,047538	0	0	0	0	0	0	0	0
103634510	LOC103634510	PE-PGRS family protein PE_PGRS16	3,39518129	0,03738	0	0	0	0	0	0	0	0
100193542	cl5362_2b	Peptidase S24/S26A/S26B/S26C family protein	0	0	0	0	0	0	0	0	1,9233397	0,0294821
100273900	LOC100273900	Peptidase S24/S26A/S26B/S26C family protein	0	0	0	0	0	0	0	0	2,6291996	0,0388675

			Yuc x Ctr		Azo x Ctr		AzoYuc x Ctr		AzoYuc x Yuc		AzoYuc x Azo	
Gene ID	Gene Symbol	Gene Description	Log2(FC)	pvalue	Log2(FC)	pvalue	Log2(FC)	pvalue	Log2(FC)	pvalue	Log2(FC)	pvalue
103635514	LOC103635514	peptide chain release factor 1	0	0	0	0	0	0	-2,969812	0,01071	0	0
100274396	pco064740	Peptide chain release factor 1	0	0	1,55815984	0,0479453	0	0	0	0	0	0
100281737	LOC100281737	peptide chain release factor 2	0	0	0	0	4,6406328	0,0091734	5,06336863	0,004471	0	0
100283982	LOC100283982	peptide methionine sulfoxide reductase	0	0	0	0	1,5554625	0,0446189	1,70925567	0,026631	0	0
100281619	LOC100281619	peptide transporter PTR2	0	0	1,98590223	0,0327091	0	0	0	0	-2,224483	0,015063
100281736	LOC100281736	peptide transporter PTR2	4,16750999	0,002588	3,20177486	0,0224715	3,6224014	0,009147	0	0	0	0
100282887	LOC100282887	peptidyl-prolyl cis-trans isomerase	0	0	0	0	0	0	0	0	-2,408654	0,01935
100384625	TIDP2776	Peptidyl-prolyl cis-trans isomerase CYP63	0	0	0	0	0	0	2,48400189	0,02026	2,0614917	0,0386184
100193972	LOC100193972	peptidyl-prolyl cis-trans isomerase FKBP43-like	0	0	0	0	0	0	2,65963416	0,032824	2,703106	0,0279441
103641695	LOC103641695	peptidyl-prolyl cis-trans isomerase FKBP53	0	0	0	0	0	0	-1,8644245	0,016302	0	0
103638313	LOC103638313	peroxidase 1	0	0	0	0	5,1951828	0,0009476	5,01066293	0,001184	5,5801768	0,0003499
100194341	LOC100194341	Peroxidase 12	0	0	0	0	-4,721753	0,0185719	0	0	0	0
100274427	pco083783	Peroxidase 16	0	0	0	0	-2,066298	0,0179408	-2,1150832	0,013373	0	0
100285056	LOC100285056	peroxidase 2	0	0	0	0	0	0	2,26362271	0,006896	0	0
103635633	LOC103635633	peroxidase 2	0	0	-4,6486588	0,0099625	0	0	0	0	4,1214194	0,0218765
103641350	LOC103641350	peroxidase 2	0	0	-3,1831396	0,0245593	-3,079464	0,0280206	0	0	0	0
103635393	LOC103635393	peroxidase 2-like	0	0	0	0	2,2445665	0,0025602	0	0	2,4188753	0,0010145
103647881	LOC103647881	peroxidase 2-like	0	0	0	0	0	0	0	0	-1,916818	0,0379088
103653548	LOC103653548	peroxidase 5	0	0	0	0	0	0	-1,6157083	0,039909	0	0
103647239	LOC103647239	peroxidase 50	0	0	0	0	3,536358	0,0029811	0	0	2,2962919	0,028856
100191769	gpm853	Peroxidase 52	0	0	-5,0707588	0,0022065	0	0	0	0	3,3593655	0,049683
103642594	LOC103642594	peroxidase 52	0	0	0	0	0	0	5,00863933	0,006812	3,54719	0,0436753
100192603	LOC100192603	Peroxidase 53	0	0	0	0	-3,629565	0,0351419	0	0	0	0
100216995	LOC100216995	peroxidase 54	1,52549406	0,045171	0	0	0	0	0	0	0	0
103638909	LOC103638909	peroxidase 57	0	0	0	0	0	0	-1,6442786	0,030017	-1,811717	0,0154219
100282124	LOC100282124	peroxidase 72	0	0	-1,5144378	0,0047738	0	0	0	0	0	0
103626198	LOC103626198	peroxidase N	0	0	0	0	2,1519367	0,023087	0	0	0	0
100282867	LOC100282867	peroxisomal biogenesis factor 19	0	0	0	0	0	0	1,64954037	0,025085	2,1778512	0,0037617
100284127	LOC100284127	peroxisomal multifunctional enzyme type 2	2,4768065	0,006893	0	0	0	0	0	0	0	0
103643490	LOC103643490	peroxisome biogenesis protein 12	0	0	0	0	0	0	2,69217855	0,015149	0	0
103633997	LOC103633997	peroxisome biogenesis protein 3-1	0	0	0	0	0	0	1,54382461	0,021471	0	0
100283630	LOC100283630	PHD finger protein	0	0	0	0	2,0881815	0,0212097	2,14886002	0,015827	2,0845997	0,0179919
103655990	LOC103655990	phenylalanine ammonia-lyase	0	0	0	0	0	0	0	0	2,739857	0,034802
100285794	LOC100285794	pherophorin like protein	-2,6135492	0,011963	-3,7545875	0,0007324	-2,832665	0,0063496	0	0	0	0
100193506	LOC100193506	phosducin-like protein 3	0	0	0	0	0	0	-1,5121019	0,019273	0	0
100284789	pco107612	Phosphatase phospho1	-2,9088161	0,034116	0	0	0	0	0	0	0	0

			Yuc x Ctr		Azo x Ctr		AzoYuc x Ctr		AzoYuc x Yuc		AzoYuc x Azo	
Gene ID	Gene Symbol	Gene Description	Log2(FC)	pvalue	Log2(FC)	pvalue	Log2(FC)	pvalue	Log2(FC)	pvalue	Log2(FC)	pvalue
100286314	LOC100286314	phosphate import ATP-binding protein pstB 1	0	0	0	0	0	0	0	0	-1,557328	0,0373545
100194409	LOC100194409	phosphatidate cytidylyltransferase	-3,8025331	2,52E-05	-3,5509932	6,819E-05	-3,106288	0,0004306	0	0	0	0
100383415	LOC100383415	Phosphatidylinositol 4-phosphate 5-kinase 2	0	0	-3,1601319	0,0239037	0	0	0	0	3,5084897	0,0100689
103647104	LOC103647104	phosphatidylinositol 4-phosphate 5-kinase 6	0	0	0	0	0	0	4,28558158	0,030804	0	0
103654120	LOC103654120	Phosphatidylinositol N-acetyglucosaminlytransferase subunit P-related	0	0	0	0	0	0	0	0	1,6061504	0,0195732
103633205	LOC103633205	phosphatidylinositol N-acetylglucosaminyltransferase subunit A	0	0	3,40097273	0,0297441	0	0	0	0	0	0
100136826	LOC100136826	phosphatidylinositol synthase 2	0	0	0	0	-2,135877	0,0278366	-2,1493391	0,02433	-1,974629	0,0383619
100274086	cl5549_1a	Phosphatidylinositol/phosphatidylcholine transfer protein SFH10	0	0	0	0	-2,384742	0,0060454	0	0	-2,021809	0,0188509
100285123	IDP504	Phosphatidylinositol/phosphatidylcholine transfer protein SFH13	0	0	0	0	0	0	0	0	-2,039359	0,0155956
100192955	LOC100192955	Phosphatidylinositol:ceramide inositolphosphotransferase 1	0	0	-2,5143906	0,0195278	0	0	0	0	0	0
100273314	LOC100273314	Phosphatidylinositol:ceramide inositolphosphotransferase 1	0	0	0	0	2,7362016	0,0134093	4,12973332	0,000549	2,6500956	0,0131876
103643201	LOC103643201	phosphatidylinositol-3-phosphatase myotubularin-1	-2,4155043	0,001357	-2,658557	0,0004298	-1,566128	0,0340486	0	0	0	0
100275554	cl40750_1	Phosphatidylserine decarboxylase proenzyme 3	1,67609769	0,045123	0	0	0	0	-1,6054517	0,045222	0	0
103649694	LOC103649694	phosphoenolpyruvate/phosphate translocator 3, chloroplastic	1,89698661	0,015885	0	0	0	0	0	0	0	0
100193796	LOC100193796	Phosphoglycolate phosphatase 1A chloroplastic	0	0	0	0	0	0	-5,1457293	0,023531	0	0
103648908	LOC103648908	phosphoinositide phosphatase SAC7	0	0	0	0	0	0	0	0	1,5185359	0,0141477
103626494	LOC103626494	phosphoinositide phospholipase C 2	0	0	1,78797625	0,0055216	0	0	0	0	-1,675611	0,0078769
103651424	LOC103651424	phospholipase A1-II 2	0	0	2,64217324	0,0257512	0	0	0	0	0	0
103649838	LOC103649838	phospholipid-transporting ATPase 2-like	0	0	0	0	0	0	4,44985847	0,007263	0	0
103632291	LOC103632291	phosphomannomutase/phosphoglucomutase	-2,8405776	2,31E-06	-2,5990835	1,356E-05	-2,282326	0,000124	0	0	0	0
103626591	LOC103626591	phosphopantetheine adenylyltransferase-like	0	0	0	0	0	0	0	0	2,0025834	0,0320442
100283358	LOC100283358	phosphosulfolactate synthase-related protein	0	0	0	0	0	0	2,02105552	0,006444	0	0

			Yuc x Ctr		Azo x Ctr		AzoYuc x Ctr		AzoYuc x Yuc		AzoYuc x Azo	
Gene ID	Gene Symbol	Gene Description	Log2(FC)	pvalue	Log2(FC)	pvalue	Log2(FC)	pvalue	Log2(FC)	pvalue	Log2(FC)	pvalue
845197	psaI	photosystem I subunit VIII	0	0	0	0	0	0	4,57837697	0,041226	4,8043064	0,0321951
100502224	LOC100502224	Phox-associated domain	0	0	-2,131076	1,36E-05	0	0	0	0	0	0
542349	phyS11	phytase	-1,8153693	0,007621	0	0	0	0	0	0	0	0
109940986	LOC109940986	phytoene synthase 2, chloroplastic	0	0	1,62727968	0,0262771	0	0	0	0	0	0
103627437	LOC103627437	phytosulfokine receptor 1	0	0	0	0	0	0	2,04231107	0,033905	0	0
100272500	pco118508(516)	PIN domain-like family protein	0	0	0	0	0	0	0	0	-2,024158	0,0296761
100284519	LOC100284519	pistil-specific extensin-like protein	0	0	0	0	0	0	0	0	-3,48939	0,0490407
100283719	LOC100283719	PIT1	0	0	0	0	-1,566094	0,0364662	0	0	0	0
100274421	LOC100274421	PLAC8 family protein	0	0	0	0	0	0	1,64217551	0,042383	0	0
100194210	LOC100194210	Plant basic secretory protein (BSP) family protein	0	0	0	0	-3,581709	0,0133949	0	0	0	0
103651667	LOC103651667	Plant mobile domain protein family	1,76955529	0,0202	1,75073958	0,0207761	0	0	0	0	0	0
100216837	LOC100216837	Plant Tudor-like RNA-binding protein	0	0	-1,7449713	0,0227486	-1,576177	0,0360025	0	0	0	0
103651281	LOC103651281	plant/F18B13-26 protein	0	0	0	0	5,7251024	0,0021748	0	0	5,4119843	0,0037601
103629390	LOC103629390	plant/F18O14-17 protein	0	0	0	0	3,0294505	0,0016896	2,13283898	0,017834	2,1916391	0,0144754
100273416	LOC100273416	plant/F20M13-60 protein	0	0	-1,6786782	0,0252766	0	0	0	0	0	0
103625874	LOC103625874	PLASMODESMATA CALLOSE-BINDING PROTEIN 3	0	0	-2,7663089	0,0452632	-3,421299	0,0201031	0	0	0	0
100279341	cl37643_1	P-loop containing nucleoside triphosphate hydrolase superfamily protein	-4,5707234	0,001748	-5,4405808	0,0002216	-4,7983	0,0010136	0	0	0	0
100273044	gpm464	P-loop containing nucleoside triphosphate hydrolase superfamily protein	0	0	0	0	0	0	0	0	1,7630632	0,0097029
100216810	LOC100216810	P-loop containing nucleoside triphosphate hydrolase superfamily protein	2,08341579	0,021769	0	0	0	0	-2,2566331	0,011782	0	0
100276773	LOC100276773	P-loop containing nucleoside triphosphate hydrolase superfamily protein	0	0	0	0	-2,153201	0,0348031	0	0	0	0
100278656	LOC100278656	P-loop containing nucleoside triphosphate hydrolase superfamily protein	0	0	0	0	0	0	-5,1626938	0,001764	0	0
100501892	LOC100501892	P-loop containing nucleoside triphosphate hydrolase superfamily protein	0	0	0	0	0	0	-1,5259548	0,046562	0	0
100383156	umc1982	P-loop containing nucleoside triphosphate hydrolase superfamily protein	0	0	0	0	-1,666349	0,0252445	0	0	0	0
100193323	LOC100193323	P-loop NTPase domain-containing protein LPA1 homolog 1	-1,5902996	0,026013	-1,9654952	0,0064408	-2,231314	0,0023045	0	0	0	0

			Yuc x Ctr		Azo x Ctr		AzoYuc x Ctr		AzoYuc x Yuc		AzoYuc x Azo	
Gene ID	Gene Symbol	Gene Description	Log2(FC)	pvalue	Log2(FC)	pvalue	Log2(FC)	pvalue	Log2(FC)	pvalue	Log2(FC)	pvalue
100284540	LOC100284540	polcalcin Jun o 2	0	0	5,4829555	0,0020199	0	0	0	0	0	0
100283108	LOC100283108	pollen-specific arabinogalacta protein BAN102	0	0	0	0	0	0	1,54238103	0,043072	0	0
109939828	LOC109939828	pollen-specific leucine-rich repeat extensin-like protein 1	-1,7571275	0,045232	0	0	0	0	0	0	0	0
100281636	LIM12	pollen-specific protein SF3	0	0	-1,7264222	0,0411698	0	0	0	0	0	0
100281953	IDP496	Pollen-specific protein-like	0	0	3,10505241	0,0215444	0	0	0	0	-2,648248	0,026696
103647201	PARP1	poly (ADP-ribose) polymerase 1	-2,0480566	0,006735	0	0	0	0	1,77127838	0,01811	0	0
103627237	LOC103627237	poly [ADP-ribose] polymerase 3	0	0	2,40011501	0,0259513	0	0	0	0	0	0
100280315	LOC100280315	Poly(ADP-ribose) glycohydrolase 1	0	0	0	0	2,3906316	0,0336602	0	0	0	0
103654662	LOC103654662	polyadenylate-binding protein-interacting protein 4	0	0	0	0	1,8073312	0,0026241	0	0	0	0
100283875	LOC100283875	polygalacturonase	0	0	0	0	0	0	0	0	4,2780649	0,018633
103629296	LOC103629296	polygalacturonase inhibitor	0	0	0	0	2,7074232	0,0002491	0	0	1,5487948	0,0263242
100192958	LOC100192958	Polygalacturonate 4-alpha-galacturonosyltransferase	0	0	0	0	0	0	1,76891872	0,021171	0	0
103633224	LOC103633224	Polynucleotidyl transferase ribonuclease H fold protein with HRDC domain	0	0	0	0	1,8532697	0,0175292	2,12058436	0,006096	0	0
103641967	LOC103641967	polyphenol oxidase, chloroplastic	0	0	0	0	0	0	0	0	4,3635889	0,0344586
100273885	PHS1	poor synapsis 1 protein	-2,0139594	0,048044	0	0	0	0	0	0	0	0
100283927	LOC100283927	postsynaptic protein CRIPT	0	0	-1,6961278	0,0121905	0	0	0	0	0	0
100279541	si660001f06	Potassium transporter 2	0	0	-1,5035852	0,0156075	0	0	0	0	0	0
103645925	LOC103645925	potassium transporter 26	0	0	0	0	0	0	4,3081613	0,04747	0	0
103641785	LOC103641785	POU domain, class 3, transcription factor 3	0	0	0	0	0	0	0	0	1,5503895	0,0434273
103645874	LOC103645874	precursor elicitor peptide3	-8,3336497	0,049806	-8,5895326	0,0431912	0	0	0	0	0	0
109945297	LOC109945297	premnaspirodiene oxygenase	-3,7850841	0,021072	0	0	0	0	0	0	0	0
100284798	LOC100284798	pre-mRNA cleavage complex II protein Clp1	0	0	0	0	0	0	0	0	2,696639	0,0063745
100273812	pco079129b	Pre-mRNA cleavage factor Im 25 kDa subunit 2	-1,5619865	0,017127	0	0	0	0	0	0	0	0
100191855	pco093291	Pre-mRNA-processing factor 19 homolog 2	-1,6449576	0,046107	0	0	0	0	0	0	0	0
100279564	LOC100279564	Pre-mRNA-splicing factor 3	2,10459002	0,026801	0	0	2,2364911	0,0174844	0	0	0	0
100284891	si606059b05	Pre-mRNA-splicing factor 38	0	0	0	0	1,5099352	0,031958	1,69089247	0,015769	1,9179965	0,0062177
103629005	LOC103629005	pre-mRNA-splicing factor SLU7	0	0	0	0	1,5960626	0,0175358	1,53469901	0,019975	1,6094012	0,0143095
100283986	LOC100283986	PRKR interacting protein 1	0	0	0	0	0	0	2,59889618	0,023605	3,6382254	0,0024214
103639274	LOC103639274	probable 2-oxoglutarate-dependent dioxygenase At5g05600	3,54988591	0,030042	0	0	0	0	0	0	0	0
109942380	LOC109942380	probable acyl-activating enzyme 6	-1,8222875	0,012146	0	0	0	0	0	0	0	0

Gene ID	Gene Symbol	Gene Description	Yuc x Ctr		Azo x Ctr		AzoYuc x Ctr		AzoYuc x Yuc		AzoYuc x Azo	
			Log2(FC)	pvalue	Log2(FC)	pvalue	Log2(FC)	pvalue	Log2(FC)	pvalue	Log2(FC)	pvalue
103650626	LOC103650626	probable adenylate kinase 1, chloroplastic	0	0	-2,4447201	0,0070959	-1,967221	0,0270851	0	0	0	0
103654185	LOC103654185	probable alpha,alpha-trehalose-phosphate synthase [UDP-forming] 9	1,74189367	0,013556	0	0	0	0	0	0	0	0
103634608	LOC103634608	probable apyrase 1	0	0	0	0	2,16039	0,0082304	2,15389499	0,007398	2,1400715	0,0073864
103638806	LOC103638806	probable beta-D-xylosidase 2	0	0	0	0	0	0	0	0	2,5649318	0,0234495
103631970	LOC103631970	probable carboxylesterase 15	0	0	0	0	1,7241728	0,0410246	0	0	2,4356455	0,0041024
103634834	LOC103634834	probable carboxylesterase 15	0	0	0	0	3,5413845	0,0225125	0	0	3,7971023	0,009746
109943959	LOC109943959	probable cation transporter HKT7	0	0	0	0	3,1551297	0,0023909	0	0	0	0
109939159	LOC109939159	probable CCR4-associated factor 1 homolog 11	0	0	0	0	0	0	0	0	1,6080581	0,0067047
103638148	LOC103638148	probable E3 ubiquitin-protein ligase XBOS36	0	0	0	0	0	0	1,5257774	0,033105	0	0
103631913	LOC103631913	probable fucosyltransferase 8	0	0	0	0	0	0	0	0	1,9024718	0,0179961
103651482	LOC103651482	probable GABA transporter 2	0	0	0	0	2,2622269	0,0245282	0	0	0	0
103641497	LOC103641497	probable galactinol--sucrose galactosyltransferase 1	0	0	0	0	3,5343321	0,0228442	0	0	0	0
103654257	LOC103654257	probable galacturonosyltransferase 9	0	0	0	0	-3,678881	0,0292271	0	0	0	0
103637314	LOC103637314	probable glutathione S-transferase GSTU6	3,48465339	0,039348	3,71903165	0,0260994	0	0	0	0	0	0
103640748	LOC103640748	probable glycosyltransferase 3	0	0	2,28926372	0,0218444	0	0	0	0	-2,248366	0,0211229
103634552	LOC103634552	probable glycosyltransferase 4	0	0	0	0	-2,024222	0,0236375	0	0	0	0
109944053	LOC109944053	probable GTP diphosphokinase RSH3, chloroplastic	0	0	0	0	0	0	-1,5190773	0,040865	0	0
103638868	LOC103638868	probable histidine kinase 1	0	0	2,53422274	0,0177934	0	0	0	0	-2,724867	0,0093477
103654967	LOC103654967	probable histone acetyltransferase HAC-like 1	0	0	2,3614268	0,0034829	0	0	0	0	0	0
103643167	LOC103643167	probable inactive histone-lysine N-methyltransferase SUVR2	2,49993918	0,031826	2,51756501	0,0298031	2,3372777	0,0442911	0	0	0	0
103632681	LOC103632681	probable inactive receptor kinase At2g26730	-3,6705719	0,023517	0	0	0	0	0	0	0	0
103645841	LOC103645841	probable isoaspartyl peptidase/L-asparaginase 2	0	0	-1,5027667	0,0142022	0	0	0	0	0	0
103626627	LOC103626627	probable LRR receptor-like serine/threonine-protein kinase	0	0	0	0	0	0	0	0	1,8407077	0,0419404
103652174	LOC103652174	probable LRR receptor-like serine/threonine-protein kinase At1g05700	0	0	1,55139636	0,0472811	0	0	0	0	0	0
103634832	LOC103634832	probable LRR receptor-like serine/threonine-protein kinase At3g47570	0	0	0	0	3,7094929	0,0331489	0	0	0	0

			Yuc x Ctr		Azo x Ctr		AzoYuc x Ctr		AzoYuc x Yuc		AzoYuc x Azo	
Gene ID	Gene Symbol	Gene Description	Log2(FC)	pvalue	Log2(FC)	pvalue	Log2(FC)	pvalue	Log2(FC)	pvalue	Log2(FC)	pvalue
103645020	LOC103645020	probable LRR receptor-like serine/threonine-protein kinase At3g47570	0	0	0	0	0	0	0	0	3,4676688	0,0479658
103652739	LOC103652739	probable LRR receptor-like serine/threonine-protein kinase At3g47570	0	0	0	0	0	0	3,87740912	0,001777	3,0687439	0,0078024
103632364	LOC103632364	probable LRR receptor-like serine/threonine-protein kinase At4g37250	0	0	0	0	3,2869755	0,0013214	2,395643	0,010608	2,1666381	0,0187498
103654507	LOC103654507	probable L-type lectin-domain containing receptor kinase S.5	0	0	1,68477047	0,0269669	0	0	0	0	0	0
103642357	LOC103642357	probable L-type lectin-domain containing receptor kinase S.7	0	0	0	0	0	0	0	0	1,6534263	0,0093922
103633579	LOC103633579	probable lysophospholipase BODYGUARD 4	0	0	0	0	0	0	0	0	4,6792744	0,021973
103637686	LOC103637686	probable methyltransferase PMT2	-1,5729106	0,038755	-2,3720418	0,0028896	0	0	0	0	0	0
103629621	LOC103629621	probable methyltransferase PMT9	0	0	0	0	0	0	-4,6421569	0,012564	0	0
103635088	LOC103635088	probable pectinesterase 55	0	0	0	0	0	0	3,04342795	0,042711	3,0680988	0,0371394
103641147	LOC103641147	probable pectinesterase 8	0	0	0	0	0	0	0	0	-3,603827	0,0315
103633115	LOC103633115	probable plastid-lipid-associated protein 12, chloroplastic	0	0	0	0	0	0	0	0	4,620566	0,0170822
103631435	LOC103631435	probable polyamine oxidase 2	0	0	0	0	0	0	0	0	4,2405857	0,013439
103645756	LOC103645756	probable polyol transporter 4	2,92805575	0,040794	0	0	0	0	0	0	0	0
103634426	LOC103634426	probable protein phosphatase 2C 31	2,95233953	0,032885	0	0	0	0	0	0	0	0
103641516	LOC103641516	probable protein phosphatase 2C 37	0	0	0	0	0	0	-2,8024926	0,033129	-2,87807	0,0276579
103645961	LOC103645961	probable protein phosphatase 2C 44	-1,7268034	0,037557	0	0	0	0	0	0	0	0
103649384	LOC103649384	probable protein S-acyltransferase 6	0	0	0	0	0	0	0	0	3,6875192	0,0483919
103650825	LOC103650825	probable protein S-acyltransferase 7	0	0	0	0	0	0	-3,3995366	0,004611	0	0
103645929	LOC103645929	probable receptor-like protein kinase At1g30570	0	0	0	0	0	0	0	0	2,170747	0,0128015
103642297	LOC103642297	probable receptor-like serine/threonine-protein kinase At5g57670	0	0	0	0	4,1956726	0,0051449	0	0	0	0
103641335	LOC103641335	probable sugar phosphate/phosphate translocator At3g14410	0	0	-1,9159794	0,0424966	0	0	0	0	0	0
103627752	LOC103627752	probable trehalose-phosphate phosphatase 4	0	0	0	0	-2,049361	0,0321687	0	0	-2,097021	0,024633
103632655	LOC103632655	probable trehalose-phosphate phosphatase 7	2,63185767	0,04701	0	0	3,6095606	0,005358	0	0	2,4056496	0,011133
103636597	LOC103636597	probable ubiquitin conjugation factor E4	0	0	0	0	1,7965505	0,0320721	1,71513212	0,038615	0	0
103650432	LOC103650432	probable Ufm1-specific protease	0	0	0	0	0	0	1,86103123	0,002502	0	0

			Yuc x Ctr		Azo x Ctr		AzoYuc x Ctr		AzoYuc x Yuc		AzoYuc x Azo	
Gene ID	Gene Symbol	Gene Description	Log2(FC)	pvalue	Log2(FC)	pvalue	Log2(FC)	pvalue	Log2(FC)	pvalue	Log2(FC)	pvalue
103654285	LOC103654285	probable WRKY transcription factor 23	0	0	-3,0007241	0,0007071	-1,776523	0,024339	-2,171461	0,004997	0	0
103639063	LOC103639063	probable WRKY transcription factor 4	0	0	1,95465762	0,0464648	2,9744563	0,0019797	1,83225332	0,021493	0	0
103638415	LOC103638415	probable xyloglucan endotransglucosylase/hydrolase protein 26	0	0	0	0	0	0	0	0	-2,014452	0,0470177
100283110	LOC100283110	profilin A	0	0	-1,6147006	0,0202337	0	0	0	0	0	0
100193074	LOC100193074	Profilin homolog5	0	0	0	0	1,5359934	0,044381	1,6826037	0,025723	3,1002313	0,000137
100192510	LOC100192510	proline-rich family protein	0	0	0	0	1,9962318	0,0268319	2,44348556	0,006798	1,9888137	0,0247225
100275488	LOC100275488	proline-rich family protein	0	0	0	0	4,4709688	0,0244616	3,93192087	0,047867	0	0
103631403	LOC103631403	proline-rich receptor-like protein kinase PERK8	0	0	0	0	0	0	0	0	-2,898842	0,0449516
100381646	pco064771(293)	prolyl oligopeptidase family protein	-1,846853	0,016455	0	0	0	0	0	0	0	0
100285233	LOC100285233	protease 2	0	0	0	0	0	0	1,95873041	0,027678	0	0
100037815	magi50422	Proteasome subunit alpha type	0	0	0	0	0	0	-1,7492019	0,033852	0	0
100191806	pco069906	Proteasome subunit beta type-6	2,142135	0,001896	0	0	0	0	-1,9113291	0,004353	0	0
103655792	LOC103655792	proteasome-associated protein ECM29 homolog	0	0	-1,6009524	0,0210917	0	0	0	0	0	0
100272296	pco062587(708)	Protein AE7	0	0	0	0	3,389927	0,0028463	2,81349373	0,008377	2,9126057	0,0061105
100282149	LOC100282149	protein AIG1	0	0	2,39169639	0,0415799	2,3042334	0,0497822	0	0	0	0
103647832	LOC103647832	protein ALP1-like	0	0	-4,9706593	0,0400505	0	0	0	0	0	0
100383756	LOC100383756	Protein arginine N-methyltransferase 1.5	0	0	0	0	2,3562513	0,0086965	0	0	1,9460307	0,0221525
109943529	LOC109943529	protein argonaute 12	0	0	-7,6790528	0,0102955	0	0	0	0	0	0
103641968	LOC103641968	protein argonaute 1B	0	0	0	0	0	0	-1,5504814	0,017688	0	0
100191172	cl11072_-2	Protein BASIC PENTACYSTEINE1	0	0	0	0	1,8182441	0,0304146	0	0	1,9720761	0,0157398
100280668	LOC100280668	protein binding protein	0	0	0	0	3,223089	0,0059394	0	0	0	0
100282596	LOC100282596	protein binding protein	-2,1200225	0,003677	-2,1511337	0,0030124	-2,442291	0,0008487	0	0	0	0
100283142	LOC100283142	protein binding protein	0	0	0	0	1,705862	0,0321121	1,6257887	0,036371	0	0
100283571	LOC100283571	protein binding protein	0	0	0	0	4,6082655	0,0064181	4,84858508	0,003876	3,3127558	0,039868
100273088	LOC100273088	Protein BTR1	-1,9428177	0,019763	0	0	-1,834228	0,021958	0	0	0	0
103633350	LOC103633350	protein BZR1 homolog 1-like	0	0	0	0	0	0	0	0	-2,3217	0,0346893
100384583	LOC100384583	Protein CHROMATIN REMODELING 24	-3,1372973	0,000126	-3,0734226	0,0001397	-3,01844	0,0001704	0	0	0	0
103641317	LOC103641317	protein CHROMATIN REMODELING 35	0	0	0	0	4,6082421	0,0098569	0	0	0	0
103636274	LOC103636274	protein CLT1, chloroplastic	-1,757254	0,046571	0	0	-1,982612	0,0243911	0	0	0	0
103631990	LOC103631990	protein containing PDZ domain a K-box domain and a TPR region	3,36146407	0,043524	3,56186937	0,030366	0	0	0	0	0	0
606437	LOC606437	protein CUP-SHAPED COTYLEDON 1	-2,642092	0,040817	-3,4656272	0,013	0	0	0	0	0	0

			Yuc x Ctr		Azo x Ctr		AzoYuc x Ctr		AzoYuc x Yuc		AzoYuc x Azo	
Gene ID	Gene Symbol	Gene Description	Log2(FC)	pvalue	Log2(FC)	pvalue	Log2(FC)	pvalue	Log2(FC)	pvalue	Log2(FC)	pvalue
103636627	LOC103636627	protein CURVATURE THYLAKOID 1C, chloroplastic	0	0	0	0	0	0	-3,9252001	0,01934	0	0
103633980	LOC103633980	protein DENND6B	-1,930601	0,019668	0	0	0	0	0	0	0	0
103626124	LOC103626124	protein DETOXIFICATION 19	0	0	2,6739929	0,0085807	0	0	0	0	0	0
103633151	LOC103633151	protein DETOXIFICATION 45, chloroplastic	0	0	0	0	0	0	2,00058495	0,028611	0	0
103627543	LOC103627543	protein DETOXIFICATION 49	-5,2573222	0,002807	-3,674233	0,0146493	-4,655226	0,0051782	0	0	0	0
103643187	LOC103643187	protein DMR6-LIKE OXYGENASE 2	2,06660792	0,031777	0	0	2,2843558	0,0163193	0	0	1,9059576	0,0212348
100274041	pco107514	Protein Dr1-like protein	-3,4726531	0,001619	-4,0089468	0,0003254	-4,719772	4,205E-05	0	0	0	0
103654479	LOC103654479	Protein ENHANCED DOWNY MILDEW 2	0	0	2,52976543	0,0275121	0	0	0	0	-2,503032	0,0242813
100501091	LOC100501091	Protein EXORDIUM	0	0	0	0	0	0	-1,8978483	0,033982	0	0
103637609	LOC103637609	Protein EXORDIUM-like 5	0	0	0	0	0	0	4,1212299	0,009381	0	0
103646318	LOC103646318	protein FAR1-RELATED SEQUENCE 6	0	0	0	0	2,4451829	0,0104218	3,70057128	0,000331	2,3288984	0,0107549
100279414	LOC100279414	Protein FLX-like 1	-3,1389712	0,001555	-2,6861927	0,0058697	-3,103621	0,0016008	0	0	0	0
103651051	LOC103651051	protein G1-like7	0	0	0	0	0	0	3,09590587	0,01814	0	0
103638867	LOC103638867	protein GLUTAMINE DUMPER 5	0	0	0	0	3,2851677	0,0272395	0	0	0	0
100274016	LOC100274016	Protein HAPLESS 2	0	0	0	0	0	0	2,07173119	0,035967	2,7525142	0,0066498
103638323	LOC103638323	protein HOMOLOG OF MAMMALIAN LYST-INTERACTING PROTEIN 5	0	0	0	0	0	0	1,95856196	0,043858	0	0
109943608	LOC109943608	protein ILITYHIA	-3,7418316	0,020852	0	0	0	0	3,48423349	0,02989	0	0
103651273	LOC103651273	protein IQ-DOMAIN 1	-4,1847817	0,01793	0	0	0	0	0	0	0	0
100281561	LOC100281561	protein kinase	1,70548764	0,024781	0	0	0	0	0	0	0	0
100281444	LOC100281444	protein kinase domain containing protein	0	0	0	0	0	0	0	0	1,6001039	0,0268846
100192473	cl28036_1b	Protein kinase superfamily protein	-1,5354242	0,043573	0	0	0	0	0	0	0	0
100502407	LOC100502407	Protein kinase superfamily protein	0	0	0	0	0	0	0	0	-1,537407	0,0215508
100382518	LOC100382518	Protein LAZ1	0	0	0	0	1,9045057	0,043304	2,37561498	0,012028	1,9430389	0,0330182
100502274	LOC100502274	Protein LAZ1	0	0	0	0	-2,239735	0,0146727	0	0	0	0
103637435	LOC103637435	protein LAZ1 homolog 2	0	0	-5,4035457	0,0012832	-3,198639	0,0184776	0	0	0	0
100384603	LOC100384603	Protein LURP-one-related 8	0	0	0	0	-1,692674	0,0308085	0	0	-2,080732	0,0058014
103655717	LOC103655717	protein MAIN-LIKE 1	-3,5098554	0,044887	0	0	0	0	0	0	0	0
103636370	LOC103636370	protein MEN-8	2,07918099	0,008547	0	0	0	0	-1,8376935	0,012187	0	0
103651104	LOC103651104	protein MICRORCHIDIA 6	0	0	0	0	0	0	0	0	-1,965539	0,0267263
100277314	LOC100277314	Protein NEF1	0	0	-1,9876086	0,016591	-2,054221	0,0134756	0	0	0	0
103649534	LOC103649534	protein NLP3	4,50352264	0,001093	0	0	0	0	-2,3706061	0,040967	0	0
103647992	LOC103647992	protein NRT1/ PTR FAMILY 1.1	0	0	0	0	0	0	2,59390102	0,036056	0	0
100191990	LOC100191990	Protein NRT1/ PTR FAMILY 3.1	0	0	0	0	-1,591817	0,0130953	-1,6930534	0,007522	0	0

			Yuc x Ctr		Azo x Ctr		AzoYuc x Ctr		AzoYuc x Yuc		AzoYuc x Azo	
Gene ID	Gene Symbol	Gene Description	Log2(FC)	pvalue	Log2(FC)	pvalue	Log2(FC)	pvalue	Log2(FC)	pvalue	Log2(FC)	pvalue
103647969	LOC103647969	protein NRT1/ PTR FAMILY 4.4	0	0	1,78141555	0,0164539	0	0	0	0	0	0
100382172	LOC100382172	Protein NRT1/ PTR FAMILY 5.10	0	0	-1,6978952	0,0073549	0	0	0	0	0	0
103643475	LOC103643475	protein NRT1/ PTR FAMILY 5.2	0	0	0	0	2,7703294	0,0340717	0	0	2,8009406	0,0231614
103653994	LOC103653994	protein NRT1/ PTR FAMILY 7.3	-4,195658	0,01375	0	0	0	0	0	0	0	0
100383742	LOC100383742	Protein NRT1/ PTR FAMILY 8.3	0	0	0	0	0	0	2,93079598	0,012507	2,7458017	0,0171367
100281135	pco103560(319)	Protein P21	-3,6150288	0,018656	0	0	0	0	0	0	0	0
100273854	cl37436_2(612)	Protein PAF1-like protein	0	0	0	0	1,99224	0,0077143	0	0	0	0
103630570	LOC103630570	protein PAIR1	0	0	0	0	-2,284959	0,0085494	0	0	-1,845721	0,0329556
103639620	LOC103639620	protein PAIR1	0	0	0	0	0	0	0	0	4,4043999	0,0246018
541808	pp2c-1	protein phosphatase type-2C	2,59272795	0,007593	0	0	2,6863804	0,0053251	0	0	0	0
100273725	LOC100273725	Protein POLLENLESS 3-LIKE 2	0	0	0	0	0	0	0	0	2,2116938	0,0139709
103641534	LOC103641534	Protein RAE1	-6,1512337	5,91E-07	-6,9609278	1,717E-08	-6,170564	5,355E-07	0	0	0	0
100384114	cl33992_1	Protein REDUCED WALL ACETYLATION 2	0	0	0	0	4,0940432	2,518E-05	3,66665625	0,000111	4,1036013	1,702E-05
109941464	LOC109941464	protein REDUCED WALL ACETYLATION 3	0	0	-1,6635899	0,0126888	-1,709797	0,0103914	0	0	0	0
109942397	LOC109942397	protein REVEILLE 6	0	0	2,14717853	0,0330871	0	0	0	0	-1,926191	0,0378862
103635420	LOC103635420	protein Rf1, mitochondrial	0	0	0	0	0	0	3,49818686	0,035333	0	0
103646232	LOC103646232	protein ROOT HAIR DEFECTIVE 3 homolog 1-like	0	0	-4,6780035	0,0076356	0	0	0	0	0	0
100280249	LOC100280249	Protein ROOT INITIATION DEFECTIVE 3	0	0	0	0	2,4028084	0,0118096	0	0	0	0
103643378	LOC103643378	protein ROOT PRIMORDIUM DEFECTIVE 1	3,82045969	0,014819	3,60217062	0,0216208	3,6589959	0,0193013	0	0	0	0
100384536	LOC100384536	Protein RRP6-like 2	0	0	0	0	1,5597651	0,021801	0	0	0	0
100191148	pco080890	Protein SCO1 homolog 1 mitochondrial	0	0	2,77007219	0,0176759	0	0	0	0	0	0
100283355	pco129491	Protein SENSITIVE TO PROTON RHIZOTOXICITY 1	1,75984642	0,018505	0	0	0	0	0	0	0	0
103644425	LOC103644425	protein SHOOT GRAVITROPISM 6	-2,0359163	6,29E-05	-1,5561284	0,0019622	0	0	0	0	0	0
103627590	LOC103627590	protein starmaker	0	0	1,76856159	0,0252781	0	0	0	0	0	0
103626758	LOC103626758	protein STRUBBELIG-RECEPTOR FAMILY 8	0	0	0	0	-2,317228	0,0014589	0	0	0	0
103650615	LOC103650615	protein SUPPRESSOR OF GENE SILENCING 3 homolog	-4,007263	0,025544	0	0	0	0	5,49462333	0,001446	2,8875629	0,0474537
100217281	LOC100217281	Protein tesmin/TSO1-like CXC 4	3,77882034	0,001202	0	0	0	0	-2,6835129	0,01287	0	0
100384736	pco123637(113)	Protein TIFY 3B	-1,6113147	0,001887	-2,370663	7,082E-06	-1,602937	0,0018653	0	0	0	0
100501833	LOC100501833	Protein TOC75-3 chloroplastic	1,66077045	0,047966	0	0	0	0	0	0	0	0
100382028	pco070745(700)	Protein TOC75-3 chloroplastic	0	0	0	0	0	0	-1,752282	0,028229	0	0
100277440	LOC100277440	Protein translocase/ protein transporter	0	0	-2,1635042	0,030236	-2,385005	0,0183472	0	0	0	0

			Yuc x Ctr		Azo x Ctr		AzoYuc x Ctr		AzoYuc x Yuc		AzoYuc x Azo	
Gene ID	Gene Symbol	Gene Description	Log2(FC)	pvalue	Log2(FC)	pvalue	Log2(FC)	pvalue	Log2(FC)	pvalue	Log2(FC)	pvalue
103626511	LOC103626511	Protein transport protein SEC16B-like protein	0	0	0	0	2,4132453	0,0012428	1,56437706	0,028373	1,5846617	0,0258192
103638902	LOC103638902	protein transport protein sec31	0	0	0	0	4,1442618	0,0024707	3,15088719	0,009069	2,8341739	0,0159245
109941581	LOC109941581	protein trichome berefringence-like 7	-1,9916263	0,047681	0	0	0	0	2,29158759	0,018085	0	0
103654216	LOC103654216	protein trichome birefringence-like 1	2,57575282	0,038741	0	0	0	0	0	0	0	0
103650115	LOC103650115	protein trichome birefringence-like 38	0	0	0	0	0	0	1,93130422	0,020334	0	0
103642120	LOC103642120	protein TSS	0	0	0	0	0	0	0	0	1,668597	0,0160674
100279531	LOC100279531	Protein UPSTREAM OF FLC	0	0	0	0	-1,886453	0,016519	-1,6570223	0,03354	0	0
103643835	LOC103643835	protein UPSTREAM OF FLC	0	0	0	0	0	0	1,74776036	0,031104	0	0
100381815	LOC100381815	Protein VAC14-like protein	-2,6202873	0,004977	-2,5361369	0,0062423	-3,448433	0,0002791	0	0	0	0
100194065	LOC100194065	Protein WALLS ARE THIN 1	0	0	0	0	0	0	2,08951172	0,018674	0	0
100279417	LOC100279417	Protein WALLS ARE THIN 1	0	0	0	0	-1,595539	0,0270352	0	0	0	0
100192097	magi67004	Protein WVD2-like 5	0	0	-1,7977164	0,0414184	0	0	0	0	0	0
100280603	LOC100280603	PTD008 protein	0	0	0	0	2,1054974	0,0096328	2,33270838	0,003839	1,8668787	0,0180365
100282599	LOC100282599	pterin-4-alpha-carbinolamine dehydratase	0	0	0	0	3,9586404	0,0177262	0	0	0	0
100283124	LOC100283124	purine permease	0	0	0	0	-2,975672	0,0015462	0	0	-1,870636	0,049505
103632874	LOC103632874	purine permease 3	0	0	0	0	-1,788062	0,0355353	0	0	-1,954445	0,0180246
100274120	LOC100274120	Purple acid phosphatase 10	0	0	0	0	2,10982	0,0138846	0	0	2,3604788	0,0049696
100272946	LOC100272946	Purple acid phosphatase 15	0	0	2,8452633	0,0342821	0	0	0	0	-2,103712	0,0489094
103643294	LOC103643294	putative 1-phosphatidylinositol-3-phosphate 5-kinase FAB1C	2,17379819	0,002809	0	0	0	0	0	0	0	0
103629934	LOC103629934	putative 1-phosphatidylinositol-3-phosphate 5-kinase FAB1D	4,78885631	0,023266	0	0	0	0	0	0	0	0
103646184	LOC103646184	putative 1-phosphatidylinositol-3-phosphate 5-kinase FAB1D	0	0	1,56395474	0,0441363	0	0	0	0	-3,043954	0,0001416
100272887	AY110514	putative 2-oxoacid dependent dioxygenase	0	0	0	0	0	0	2,20059871	0,000288	1,802008	0,0023121
100272598	LOC100272598	Putative ABC1 protein	0	0	0	0	0	0	3,28054986	0,00246	0	0
100382954	GNAT21	putative acyl-CoA N-acyltransferases (NAT) family protein	0	0	0	0	0	0	0	0	2,4947748	0,0449072
100279721	csu191	putative ADP-ribosylation factor GTPase-activating protein AGD14	1,98891056	0,01626	0	0	0	0	0	0	0	0
100272357	LOC100272357	putative alcohol dehydrogenase superfamily protein	0	0	0	0	1,9502702	0,0292753	1,61414206	0,046561	0	0
107522037	LOC107522037	Putative alcohol dehydrogenase superfamily protein	0	0	0	0	1,7667384	0,0142015	0	0	1,512709	0,0277042
103634718	LOC103634718	putative aldehyde dehydrogenase family protein	0	0	0	0	0	0	-2,6271235	0,038623	0	0
100217129	LOC100217129	putative AP2/EREBP transcription factor superfamily protein	0	0	0	0	3,9075581	0,0153258	0	0	0	0

			Yuc x Ctr		Azo x Ctr		AzoYuc x Ctr		AzoYuc x Yuc		AzoYuc x Azo	
Gene ID	Gene Symbol	Gene Description	Log2(FC)	pvalue	Log2(FC)	pvalue	Log2(FC)	pvalue	Log2(FC)	pvalue	Log2(FC)	pvalue
100272244	LOC100272244	putative apyrase family protein	1,72198509	0,025359	0	0	0	0	0	0	0	0
100384615	LOC100384615	putative arabinose 5-phosphate isomerase	0	0	-2,9566572	0,0185406	0	0	0	0	0	0
100279909	LOC100279909	Putative beta-glucosidase 41	0	0	0	0	3,490406	0,0006933	3,24629794	0,001271	3,8175572	0,0001786
100191685	LOC100191685	putative bZIP transcription factor superfamily protein	0	0	0	0	1,7650308	0,045833	0	0	0	0
100217162	LOC100217162	putative calcium-dependent protein kinase family protein	0	0	0	0	0	0	1,90322806	0,049259	0	0
100277352	LOC100277352	putative calcium-dependent protein kinase family protein	0	0	0	0	0	0	1,59633596	0,036064	0	0
100383398	LOC100383398	putative calmodulin-binding family protein	0	0	0	0	3,4140189	0,000838	2,05172579	0,036885	3,0175097	0,0024216
100193569	LOC100193569	putative CBL-interacting protein kinase family protein	0	0	0	0	0	0	0	0	2,4526274	0,0497737
100383954	LOC100383954	putative CBL-interacting protein kinase family protein	0	0	1,85343749	0,0243243	0	0	0	0	0	0
103631292	LOC103631292	putative cellulose synthase A catalytic subunit 11 [UDP-forming]	4,76265871	0,002803	0	0	3,9525978	0,0140272	0	0	0	0
100193235	pco093519b	Putative chaperone clbp family protein	0	0	0	0	4,1489111	0,0070069	5,50870321	0,000503	4,5514954	0,0029784
100216819	LOC100216819	putative cinnamyl-alcohol dehydrogenase family protein	0	0	0	0	2,0005851	0,0243116	2,4998926	0,005015	2,5772339	0,0036858
100283913	LOC100283913	putative conserved membrane protein	0	0	0	0	-3,639721	0,0340641	0	0	0	0
100192876	LOC100192876	Putative cytochrome P450 superfamily protein	0	0	2,50223716	0,0278708	2,5964719	0,0220842	0	0	0	0
100273344	LOC100273344	putative cytochrome P450 superfamily protein	-4,9623306	0,006024	0	0	0	0	0	0	0	0
100273597	LOC100273597	putative cytochrome P450 superfamily protein	1,73967637	0,046058	0	0	0	0	-2,3017798	0,007722	0	0
100274015	LOC100274015	putative cytochrome P450 superfamily protein	-2,4183436	0,005478	-1,7323798	0,0408918	-2,005848	0,0185171	0	0	0	0
100274430	LOC100274430	putative cytochrome P450 superfamily protein	0	0	0	0	0	0	0	0	2,2919639	0,0288703
100280034	LOC100280034	putative cytochrome P450 superfamily protein	-5,5706763	9,27E-06	-2,070782	0,0076073	-2,68167	0,0008121	2,88899586	0,025445	0	0
100383503	LOC100383503	putative cytochrome P450 superfamily protein	0	0	0	0	0	0	2,48943247	0,009935	2,1863342	0,0204971
103641973	LOC103641973	putative deoxyribonuclease	0	0	0	0	0	0	0	0	2,7024497	0,0474478
100274480	pco134925	putative desiccation-related protein LEA14	0	0	0	0	-1,904732	0,0032397	0	0	0	0

			Yuc x Ctr		Azo x Ctr		AzoYuc x Ctr		AzoYuc x Yuc		AzoYuc x Azo	
Gene ID	Gene Symbol	Gene Description	Log2(FC)	pvalue	Log2(FC)	pvalue	Log2(FC)	pvalue	Log2(FC)	pvalue	Log2(FC)	pvalue
103632580	LOC103632580	putative disease resistance protein RGA1	-2,4917135	0,000711	-1,587383	0,0267368	0	0	0	0	0	0
103630851	LOC103630851	putative disease resistance protein RGA3	0	0	0	0	1,613807	0,0249869	0	0	0	0
103638075	LOC103638075	putative disease resistance protein RGA3	0	0	0	0	1,8894091	0,0184505	1,56285294	0,039562	0	0
103640708	LOC103640708	putative disease resistance protein RGA3	0	0	0	0	0	0	1,61951261	0,015795	0	0
100279616	LOC100279616	Putative disease resistance RPP13-like protein 1	0	0	0	0	1,7841898	0,0329553	1,75514995	0,029328	0	0
103648157	LOC103648157	putative disease resistance RPP13-like protein 2	0	0	0	0	0	0	0	0	2,5549269	0,0244936
103627692	LOC103627692	putative disease resistance RPP13-like protein 3	2,82140847	0,046244	0	0	0	0	0	0	0	0
100384761	LOC100384761	putative DNA-binding bromodomain-containing family protein	0	0	2,7250703	0,0437748	0	0	0	0	0	0
100282286	pco132322	putative dolichyl-diphosphooligosaccharide--protein glycosyltransferase subunit 3B	0	0	0	0	2,3024713	0,0290735	0	0	0	0
103630129	LOC103630129	putative domain of unknown function (DUF641) containing family protein	0	0	0	0	0	0	1,72915235	0,026276	0	0
100383586	LOC100383586	putative DUF231 domain containing family protein	0	0	0	0	0	0	0	0	3,8749565	0,0045121
100216999	LOC100216999	Putative E3 ubiquitin-protein ligase XBAT31	-1,7212456	0,01878	0	0	-1,510871	0,032827	0	0	0	0
103651400	LOC103651400	putative exosome complex component rrp40	-2,6457673	0,01388	0	0	0	0	0	0	0	0
100272968	LOC100272968	putative galacturonosyltransferase 10	2,42907878	0,014689	0	0	0	0	0	0	0	0
100191914	LOC100191914	putative galacturonosyltransferase-like 7	0	0	0	0	2,2334404	0,0334856	2,05154332	0,043617	0	0
100191794	umc2341	putative galacturonosyltransferase-like 7	-1,5637734	0,018942	0	0	0	0	0	0	0	0
100502524	LOC100502524	putative geranylgeranyl pyrophosphate synthase 3	0	0	-1,7313256	0,0474512	0	0	0	0	0	0
100276438	LOC100276438	Putative glucuronosyltransferase PGSIP7	0	0	0	0	2,0025088	0,0232202	0	0	1,9832346	0,0211851
103646979	LOC103646979	putative glycine-rich cell wall structural protein 1	0	0	0	0	0	0	0	0	1,6262296	0,03232
100216625	bHLH168	putative HLH DNA-binding domain superfamily protein	0	0	0	0	2,6488624	0,0493707	0	0	0	0

			Yuc x Ctr		Azo x Ctr		AzoYuc x Ctr		AzoYuc x Yuc		AzoYuc x Azo	
Gene ID	Gene Symbol	Gene Description	Log2(FC)	pvalue	Log2(FC)	pvalue	Log2(FC)	pvalue	Log2(FC)	pvalue	Log2(FC)	pvalue
100272246	bHLH39	putative HLH DNA-binding domain superfamily protein	0	0	0	0	1,627237	0,0478167	0	0	0	0
100279537	bHLH94	putative HLH DNA-binding domain superfamily protein	0	0	0	0	0	0	0	0	1,5807821	0,0141164
100275319	LOC100275319	putative HLH DNA-binding domain superfamily protein	0	0	3,68120608	0,019723	0	0	0	0	0	0
100279552	HB131	putative homeodomain-like transcription factor superfamily protein	0	0	0	0	0	0	3,09532373	0,008921	0	0
100502236	LOC100502236	putative homeodomain-like transcription factor superfamily protein	0	0	0	0	0	0	3,93695338	0,022908	0	0
103641593	LOC103641593	Putative homeodomain-like transcription factor superfamily protein	0	0	0	0	1,9863829	0,0021601	1,63925055	0,00739	0	0
100280076	THX25	putative homeodomain-like transcription factor superfamily protein	3,04028915	0,047547	0	0	0	0	0	0	0	0
109945111	LOC109945111	putative hydro-lyase KRH_21160	0	0	-7,3031696	0,0279197	-7,333516	0,0272758	0	0	0	0
100272729	si606013c03	putative inactive heme oxygenase 2 chloroplastic	-2,636418	0,017721	-3,4059788	0,0030444	-2,235846	0,0387124	0	0	0	0
100272330	LOC100272330	Putative leucine-rich repeat protein kinase family protein	-2,3483188	0,036467	0	0	0	0	0	0	0	0
100501230	LOC100501230	putative leucine-rich repeat protein kinase family protein	1,75090091	0,013228	0	0	0	0	-1,8074669	0,008699	0	0
100192366	LOC100192366	putative leucine-rich repeat receptor-like protein kinase family protein	0	0	0	0	1,9886688	0,0001585	0	0	0	0
100382395	LOC100382395	putative leucine-rich repeat receptor-like protein kinase family protein	0	0	0	0	-1,994172	0,0002827	0	0	0	0
100381506	LOC100381506	putative LOB domain-containing family protein	0	0	-1,9093755	0,0494792	-2,036743	0,037365	0	0	0	0
100383891	LOC100383891	Putative lysine-specific demethylase JMJ16	-1,9404042	0,02544	-1,6959757	0,0483217	-1,998444	0,0206833	0	0	0	0
100279630	LOC100279630	putative MADS-box transcription factor family protein	-1,7506662	0,005759	0	0	0	0	0	0	0	0
103637617	LOC103637617	putative magnesium transporter MRS2-G	0	0	0	0	2,2743098	0,032584	0	0	0	0
100382015	LOC100382015	putative magnesium transporter NIPA4	0	0	0	0	0	0	1,74850504	0,02423	2,3001152	0,0035678
100282528	LOC100282528	putative magnesium transporter NIPA6	0	0	0	0	-1,621224	0,0054103	0	0	0	0

			Yuc x Ctr		Azo x Ctr		AzoYuc x Ctr		AzoYuc x Yuc		AzoYuc x Azo	
Gene ID	Gene Symbol	Gene Description	Log2(FC)	pvalue	Log2(FC)	pvalue	Log2(FC)	pvalue	Log2(FC)	pvalue	Log2(FC)	pvalue
103628997	LOC103628997	putative mannan endo-1,4-beta-mannosidase 5	0	0	0	0	-1,606794	0,023214	0	0	-1,545252	0,0246072
100193757	LOC100193757	putative mannan synthase 7	0	0	0	0	0	0	0	0	2,0562268	0,0349038
100191147	LOC100191147	Putative MAPKKK family protein kinase	0	0	1,57854013	0,0188437	0	0	0	0	0	0
100381765	LOC100381765	putative MAPKKK family protein kinase isoform 2	0	0	0	0	0	0	-2,5728541	0,020599	0	0
100501847	LOC100501847	putative MATE efflux family protein	0	0	0	0	0	0	2,71909706	0,048362	0	0
100279387	LOC100279387	putative metal-nicotianamine transporter YSL6	0	0	0	0	2,4897349	0,0049892	0	0	0	0
103633404	LOC103633404	putative methylesterase 11, chloroplastic	0	0	0	0	0	0	0	0	2,8737216	0,0382283
103651542	LOC103651542	putative methyltransferase DDB_G0268948	3,59599778	0,000803	0	0	0	0	-2,2696165	0,01269	0	0
101027245	LOC101027245	putative methyltransferase PMT15	-2,2785686	0,013705	0	0	0	0	0	0	0	0
100285805	EMB2219	putative mitochondrial transcription termination factor family protein	0	0	0	0	3,8220593	0,0146485	3,02093137	0,038734	3,9867481	0,0085801
100193811	mTRF8	putative mitochondrial transcription termination factor family protein	0	0	0	0	2,7710351	0,0416461	0	0	2,7198303	0,0346028
100382399	LOC100382399	Putative MO25-like protein	0	0	0	0	2,2112887	0,019556	1,91232525	0,037731	2,2918632	0,0133405
100272614	LOC100272614	putative MYB DNA-binding domain superfamily protein	0	0	0	0	0	0	0	0	1,7631608	0,0407255
100273616	LOC100273616	putative MYB DNA-binding domain superfamily protein	0	0	3,61641846	0,036114	0	0	0	0	0	0
100384757	LOC100384757	putative MYB DNA-binding domain superfamily protein	0	0	2,86993569	0,037034	0	0	0	0	0	0
100193481	MYB83	putative MYB DNA-binding domain superfamily protein	0	0	0	0	4,1207101	0,0404562	0	0	0	0
100279912	NAC57	putative NAC domain transcription factor superfamily protein	0	0	0	0	0	0	0	0	2,5349669	0,0360359
100281057	TIP	putative NAC domain transcription factor superfamily protein	0	0	-1,8080449	0,0436197	-2,014314	0,0252141	0	0	0	0
103654396	LOC103654396	putative nuclease HARBI1	0	0	0	0	-3,578354	0,0441003	0	0	0	0
100272570	LOC100272570	putative oxysterol binding domain family protein	1,50565726	0,038116	0	0	0	0	0	0	0	0
103627042	LOC103627042	putative pectinesterase 63	0	0	3,65396064	0,0260279	4,111146	0,0117759	0	0	0	0
100384243	LOC100384243	putative pectinesterase/pectinesterase inhibitor 13	0	0	0	0	0	0	2,2046785	0,026159	0	0
100194323	LOC100194323	Putative pentatricopeptide repeat-containing protein	0	0	-4,6476731	0,0151476	0	0	0	0	0	0

			Yuc x Ctr		Azo x Ctr		AzoYuc x Ctr		AzoYuc x Yuc		AzoYuc x Azo	
Gene ID	Gene Symbol	Gene Description	Log2(FC)	pvalue	Log2(FC)	pvalue	Log2(FC)	pvalue	Log2(FC)	pvalue	Log2(FC)	pvalue
103629748	LOC103629748	putative pentatricopeptide repeat-containing protein At3g08820	0	0	-4,4872024	0,0102472	0	0	0	0	3,4934959	0,0479831
103638375	LOC103638375	putative phospholipid-transporting ATPase 9	0	0	0	0	0	0	0	0	1,7379948	0,0038008
100279869	LOC100279869	putative phototropic-resoponsive NPH3 family protein	0	0	0	0	-1,515484	0,0221265	0	0	0	0
103637063	LOC103637063	putative polyol transporter 1	0	0	3,08413677	0,009537	0	0	0	0	-2,128829	0,0461398
100272381	LOC100272381	putative prolyl 4-hydroxylase 12	0	0	3,72773832	0,0434597	0	0	0	0	0	0
100272528	LOC100272528	putative protein kinase superfamily protein	0	0	0	0	0	0	1,81125232	0,005518	1,9291084	0,0028931
100272561	LOC100272561	putative protein kinase superfamily protein	0	0	0	0	5,643894	0,0024633	4,10713544	0,018832	3,8361116	0,0214247
100274135	LOC100274135	putative protein kinase superfamily protein	2,89023022	0,000363	0	0	0	0	-2,6389689	0,00085	0	0
100274231	LOC100274231	putative protein kinase superfamily protein	2,95927026	0,006313	2,36737791	0,0302776	2,9109933	0,0070083	0	0	0	0
100281653	LOC100281653	putative protein kinase superfamily protein	0	0	0	0	0	0	0	0	2,6489584	0,0192813
100381954	LOC100381954	putative protein kinase superfamily protein	-2,8054018	0,00309	-1,9958967	0,0181723	0	0	0	0	0	0
100283350	pco087470	putative protein phosphatase 2C 42	0	0	0	0	0	0	2,15682223	0,016321	0	0
100382074	LOC100382074	putative protein phosphatase 2C 68	0	0	0	0	2,4573098	0,0122548	0	0	0	0
100193736	LOC100193736	putative protein phosphatase 2C family protein	0	0	-3,0273837	0,0142945	-3,014319	0,014331	0	0	0	0
100272395	LOC100272395	putative protein phosphatase 2C family protein	0	0	0	0	2,1108916	0,0273671	1,86502104	0,044132	2,1329516	0,0217056
100273154	LOC100273154	putative protein S-acyltransferase 16	0	0	1,65374931	0,0161566	0	0	0	0	0	0
100383535	LOC100383535	putative receptor-like protein kinase family protein	0	0	0	0	0	0	1,63810946	0,015602	0	0
100304276	LOC100304276	putative regulator of chromosome condensation (RCC1) family protein	-1,828221	0,045387	0	0	0	0	0	0	0	0
103635232	LOC103635232	rboh5 - respiratory burst oxidase 5 (GRMZM2G323731)	4,28718614	0,002947	0	0	0	0	-5,5894091	0,000354	0	0
100280177	LOC100280177	putative ribosomal protein S8 family protein	0	0	0	0	-1,728001	0,0092981	0	0	0	0
100192925	LOC100192925	putative RING zinc finger domain superfamily protein	0	0	0	0	0	0	3,57555255	0,028248	0	0
100272564	LOC100272564	putative RING zinc finger domain superfamily protein	0	0	-1,5228802	0,0103037	0	0	0	0	0	0
100274803	LOC100274803	putative RING zinc finger domain superfamily protein	-3,9436608	0,001797	-4,3041217	0,0007433	-3,326916	0,0058804	0	0	0	0

			Yuc x Ctr		Azo x Ctr		AzoYuc x Ctr		AzoYuc x Yuc		AzoYuc x Azo	
Gene ID	Gene Symbol	Gene Description	Log2(FC)	pvalue	Log2(FC)	pvalue	Log2(FC)	pvalue	Log2(FC)	pvalue	Log2(FC)	pvalue
100279533	LOC100279533	putative RING zinc finger domain superfamily protein	1,6902445	0,043482	0	0	0	0	0	0	0	0
100383448	LOC100383448	putative RING zinc finger domain superfamily protein	0	0	2,17495863	0,0005732	0	0	0	0	0	0
100384847	LOC100384847	Putative RING zinc finger domain superfamily protein	2,79083831	0,002393	0	0	0	0	-3,0168145	0,000768	0	0
100501846	LOC100501846	putative RING zinc finger domain superfamily protein	0	0	0	0	2,8091001	0,0020734	2,34371201	0,008813	2,7923646	0,0018967
100217208	cl9164_1a	Putative RNA polymerase II subunit B1 CTD phosphatase RPAP2-like protein	0	0	2,70428898	0,0035495	0	0	0	0	-1,793073	0,0270189
100191254	LOC100191254	putative serine/threonine protein phosphatase superfamily protein	0	0	0	0	0	0	1,97016033	0,036337	0	0
100170244	SINA6	putative seven in absentia domain family protein	0	0	0	0	3,6742676	0,0220967	3,33337038	0,024273	0	0
100279804	LOC100279804	putative signal peptide peptidase family protein	0	0	0	0	0	0	2,43476801	0,017283	0	0
103642087	LOC103642087	Putative SNF2-domain/RING finger domain/helicase domain protein	0	0	0	0	4,2664006	0,0079434	0	0	0	0
100501569	LOC100501569	putative SPOC domain / Transcription elongation factor S-II protein	-1,7092191	0,018096	0	0	0	0	1,86897739	0,008322	0	0
100273623	LOC100273623	putative SPRY-domain family protein	0	0	0	0	1,7825922	0,0440494	2,30102673	0,009565	1,8573048	0,0336409
100501616	LOC100501616	putative subtilase family protein	-2,9912516	0,015684	0	0	0	0	2,93019229	0,016311	0	0
100383801	LOC100383801	Putative SWI/SNF-related matrix-associated actin-dependent regulator of chromatin subfamily A member 3-like 3	0	0	0	0	0	0	1,76017037	0,018862	0	0
100381530	LOC100381530	putative TCP-1/cpn60 chaperonin family protein	-2,6860724	0,001911	-1,9262025	0,0229474	-2,252425	0,0081167	0	0	0	0
100283194	pco100031b	putative translation elongation factor family protein isoform 1	-4,3090653	0,000294	-3,3650547	0,0024501	-4,098008	0,0003705	0	0	0	0
100274276	LOC100274276	putative tRNA (guanine(26)-N(2))-dimethyltransferase 2	0	0	0	0	0	0	3,87322699	0,035498	4,570333	0,014664
100382564	si605022b11(700)	putative tRNA (guanine(26)-N(2))-dimethyltransferase 2	0	0	-1,5110363	0,0390029	0	0	0	0	0	0
100194284	pco104496	putative ubiquitin-conjugating enzyme E2 25	3,00067169	0,001051	2,14497582	0,0205815	0	0	0	0	0	0
103625696	LOC103625696	putative ubiquitin-like-specific protease 1B	0	0	1,50814215	0,0464764	0	0	0	0	0	0
100275553	LOC100275553	Putative UPF0481 protein	0	0	0	0	-1,656308	0,0042191	-2,1063628	0,000176	0	0
103648661	LOC103648661	putative UPF0496 protein 2	0	0	0	0	-1,518632	0,0412646	0	0	-1,593097	0,0298009

			Yuc x Ctr		Azo x Ctr		AzoYuc x Ctr		AzoYuc x Yuc		AzoYuc x Azo	
Gene ID	Gene Symbol	Gene Description	Log2(FC)	pvalue	Log2(FC)	pvalue	Log2(FC)	pvalue	Log2(FC)	pvalue	Log2(FC)	pvalue
100216858	LOC100216858	putative vesicle-associated membrane protein family protein	1,73510139	0,020695	0	0	0	0	-1,6400464	0,023783	0	0
103643337	LOC103643337	putative wall-associated receptor kinase-like 16	0	0	0	0	0	0	4,7246017	0,018659	0	0
103641407	LOC103641407	Putative WD40-like beta propeller repeat family protein	0	0	0	0	0	0	0	0	-2,343801	0,0031724
100501518	LOC100501518	putative WRKY DNA-binding domain superfamily protein	0	0	0	0	1,6022584	0,0276548	1,57493714	0,026905	0	0
100501702	LOC100501702	Putative WRKY DNA-binding domain superfamily protein	0	0	0	0	1,8499298	0,0440641	2,16524629	0,017804	0	0
103630738	LOC103630738	Putative WRKY DNA-binding domain superfamily protein	0	0	0	0	3,7923373	0,0305531	0	0	0	0
107457600	LOC107457600	Putative WRKY DNA-binding domain superfamily protein	0	0	0	0	-4,706616	0,0252847	0	0	0	0
100191206	WRKY120	putative WRKY DNA-binding domain superfamily protein	0	0	0	0	0	0	0	0	5,9335413	0,0388736
100194255	WRKY29	putative WRKY DNA-binding domain superfamily protein	0	0	1,7340447	0,0313198	0	0	0	0	0	0
100279449	WRKY42	putative WRKY DNA-binding domain superfamily protein	0	0	2,67901201	0,0320157	0	0	0	0	0	0
100384128	LOC100384128	putative WRKY transcription factor 34	0	0	0	0	2,4830663	0,0005199	2,95798763	3,79E-05	3,3191732	4,663E-06
100273667	LOC100273667	putative xyloglucan endotransglucosylase/hydrolase protein 32	0	0	0	0	3,2888773	0,0350059	0	0	2,6267439	0,0498146
100194385	LOC100194385	putative xylose isomerase family protein	0	0	0	0	0	0	2,0263316	0,009389	1,8166126	0,0189531
100193942	LOC100193942	putidaredoxin reductase homolog1	-3,6112482	0,034755	0	0	0	0	0	0	0	0
103629709	LOC103629709	PX domain-containing protein EREL1	-2,3016074	0,032654	0	0	0	0	0	0	0	0
100280069	LOC100280069	Pyridoxal phosphate (PLP)-dependent transferase superfamily protein	0	0	0	0	1,9100721	0,0083575	0	0	1,8198869	0,0083414
100277985	LOC100277985	Pyridoxine/pyridoxamine 5'-phosphate oxidase 2	0	0	0	0	0	0	3,17496945	0,025196	2,7616921	0,0403552
100286099	LOC100286099	pyrophosphate-energized vacuolar membrane proton pump	-5,4911599	0,001169	-5,949336	0,0004916	-6,015354	0,0004268	0	0	0	0
101027252	LOC101027252	pyrophosphate--fructose 6-phosphate 1-phosphotransferase subunit alpha	-1,5088369	0,044439	0	0	0	0	0	0	0	0
542651	pdc2	pyruvate decarboxylase	0	0	0	0	0	0	0	0	-2,714229	0,0351255
100192883	LOC100192883	RAD1 DNA repair protein homolog	-1,6396026	0,023714	0	0	0	0	0	0	0	0
100193308	ra2	ramosa 2	0	0	0	0	0	0	-2,1846352	0,025939	0	0
103649620	LOC103649620	RAP	2,8140927	0,040756	0	0	0	0	0	0	0	0

			Yuc x Ctr		Azo x Ctr		AzoYuc x Ctr		AzoYuc x Yuc		AzoYuc x Azo	
Gene ID	Gene Symbol	Gene Description	Log2(FC)	pvalue	Log2(FC)	pvalue	Log2(FC)	pvalue	Log2(FC)	pvalue	Log2(FC)	pvalue
100127017	ras1	ras related protein 1	0	0	0	0	0	0	2,2487516	0,040531	0	0
100272423	LOC100272423	ras-related protein ARA-3	0	0	0	0	0	0	0	0	-2,050528	0,0094332
100284328	LOC100284328	ras-related protein Rab-18	0	0	0	0	0	0	0	0	-3,292633	0,0461395
100383532	LOC100383532	Receptor-like kinase-like	-3,7169783	0,040088	0	0	0	0	0	0	0	0
100283678	LOC100283678	receptor-like protein kinase	0	0	0	0	-2,531842	0,0289393	0	0	0	0
100285492	LOC100285492	receptor-like protein kinase 5	-2,398585	0,031297	-2,2031972	0,0411845	-2,3677	0,0296558	0	0	0	0
103639167	LOC103639167	receptor-like protein kinase FERONIA	0	0	0	0	-1,921263	0,0038373	0	0	0	0
100286383	LOC100286383	receptor-like protein kinase RK20-1	0	0	0	0	0	0	0	0	1,6360574	0,001829
109942244	LOC109942244	receptor-like serine/threonine-protein kinase At3g01300	0	0	0	0	2,5581936	0,0155039	3,21853823	0,002524	2,8589185	0,0063845
103636588	LOC103636588	receptor-like serine/threonine-protein kinase SD1-8	-2,1536584	0,010051	0	0	0	0	0	0	0	0
103645885	LOC103645885	receptor-like serine/threonine-protein kinase SD1-8	0	0	0	0	3,0670384	0,0123414	3,34888748	0,005673	3,6780737	0,0026195
541709	rad51a	recombination protein51 gene a	0	0	0	0	-1,552904	0,0239603	0	0	0	0
100304236	pco061645(76)	Regulator of nonsense transcripts 1-like protein	0	0	0	0	-1,710557	0,0085009	0	0	0	0
100272449	LOC100272449	Regulator of Vps4 activity in the MVB pathway protein	-2,9389064	0,044702	0	0	0	0	0	0	0	0
103640651	LOC103640651	regulatory-associated protein of TOR 2	-2,6661442	0,006009	-2,072501	0,0283039	-1,924546	0,0406421	0	0	0	0
100192691	LOC100192691	Remorin family protein	1,95227354	0,026405	0	0	1,9863896	0,0231356	0	0	0	0
100277761	LOC100277761	Remorin family protein	3,19296149	0,004163	0	0	0	0	-2,2743962	0,027995	0	0
103653295	LOC103653295	Remorin family protein	4,63335456	0,011463	0	0	0	0	-3,2098472	0,03935	0	0
100216703	LOC100216703	replication factor C subunit 3	0	0	-2,5879358	0,021276	0	0	0	0	0	0
100037782	rmr7	required to maintain repression 7	-1,8019683	0,006706	-1,5865057	0,0146143	0	0	0	0	0	0
100501929	LOC100501929	resistance to phytophthora 1	-3,7452772	0,010728	-3,298813	0,0186788	-5,089461	0,0020632	0	0	0	0
103648040	LOC103648040	respiratory burst oxidase homolog protein B	0	0	3,36589262	0,0399308	0	0	0	0	0	0
103648044	LOC103648044	respiratory burst oxidase homolog protein B	0	0	0	0	-1,916151	0,0458088	0	0	-2,593927	0,0048605
100191362	pco082817	Reticulon-like protein B4	0	0	0	0	0	0	2,01297979	0,021043	1,9332831	0,0244858
100285880	LOC100285880	retinol dehydrogenase 12	0	0	0	0	-3,23573	0,0332969	-5,2580912	0,000335	0	0
100192500	LOC100192500	retrotransposon protein	0	0	0	0	0	0	0	0	-4,845144	0,0051556
100286141	LOC100286141	retrotransposon protein SINE subclass	0	0	0	0	1,9337636	0,0033635	1,61856293	0,012554	0	0
100285962	LOC100285962	rho GDP-dissociation inhibitor 1	0	0	0	0	0	0	-1,6242696	0,028713	-1,856451	0,0113584
100501537	LOC100501537	Rho GTPase activation protein (RhoGAP) with PH domain	0	0	2,36135316	0,0091776	0	0	0	0	-3,256962	0,0004041
103633525	LOC103633525	rho GTPase-activating protein 7	0	0	0	0	1,6620609	0,0429494	1,6048758	0,045542	0	0

			Yuc x Ctr		Azo x Ctr		AzoYuc x Ctr		AzoYuc x Yuc		AzoYuc x Azo	
Gene ID	Gene Symbol	Gene Description	Log2(FC)	pvalue	Log2(FC)	pvalue	Log2(FC)	pvalue	Log2(FC)	pvalue	Log2(FC)	pvalue
103633789	LOC103633789	rho GTPase-activating protein 7	0	0	1,71461278	0,0264285	0	0	0	0	0	0
109940910	LOC109940910	rho GTPase-activating protein 7-like	0	0	0	0	3,5964335	0,0226429	5,48818178	0,002075	0	0
103638184	LOC103638184	rho GTPase-activating protein REN1	3,27448011	0,038483	0	0	0	0	0	0	0	0
103644349	LOC103644349	rhodanese-like domain-containing protein 8, chloroplastic	0	0	3,24606206	0,0335958	0	0	0	0	0	0
100191668	LOC100191668	RHOMBOID-like protein 3	0	0	-2,5287055	0,0449161	0	0	0	0	0	0
100275753	LOC100275753	RHOMBOID-like protein 9 chloroplastic	3,16509685	0,033253	0	0	0	0	0	0	0	0
542503	rop7	Rho-related protein from plants 7	-5,7242609	0,003152	-9,3056443	2,046E-05	-7,266205	0,000452	0	0	0	0
103640768	LOC103640768	ribonuclease H2 subunit A	0	0	0	0	0	0	-3,1477258	0,004902	0	0
103630287	LOC103630287	ribonuclease P protein subunit p29-like	3,55735324	0,027471	0	0	0	0	0	0	0	0
100285676	LOC100285676	ribose-5-phosphate isomerase	0	0	1,90520878	0,0193937	2,5262908	0,0017576	0	0	0	0
100274933	LOC100274933	Ribosomal protein L18ae family	-3,0726155	0,006147	-3,6958166	0,0013824	-2,343074	0,0280982	0	0	0	0
542547	LOC542547	ribosomal protein L2	0	0	0	0	-1,606653	0,0080759	0	0	0	0
541790	rpl29	ribosomal protein L29	0	0	0	0	0	0	-3,9231438	0,027699	0	0
542444	rpl39	ribosomal protein L39	0	0	-1,5157357	0,005938	0	0	0	0	0	0
100272255	IDP476	ribosomal protein13	0	0	0	0	0	0	-2,0786143	0,006192	0	0
100285083	LOC100285083	ribosome biogenesis protein RLP24	0	0	-1,5791961	0,0100854	0	0	0	0	0	0
100282035	LOC100282035	ring finger protein	3,55354491	0,029738	3,25380315	0,0471912	3,4131383	0,0361908	0	0	0	0
100194198	LOC100194198	RING/U-box superfamily protein	0	0	0	0	1,593788	0,0187695	0	0	0	0
100502508	LOC100502508	RING/U-box superfamily protein	4,0274705	0,00359	0	0	0	0	0	0	0	0
103630632	LOC103630632	RING/U-box superfamily protein	0	0	0	0	6,2500213	0,0032264	4,06205308	0,028465	4,7509747	0,0118856
100282229	pco067763	RING/U-box superfamily protein	0	0	0	0	0	0	-1,6838302	0,007988	0	0
100217225	pco137118d	RING/U-box superfamily protein	0	0	-1,5258397	0,0188635	0	0	0	0	0	0
100383259	umc1452	RING/U-box superfamily protein	0	0	0	0	1,8722055	0,0090161	0	0	0	0
103654184	LOC103654184	RING-H2 finger protein ATL13	0	0	0	0	0	0	0	0	2,3218951	0,041237
100284922	LOC100284922	RING-H2 finger protein ATL2B	2,4202801	0,002946	0	0	0	0	-2,2517644	0,003513	0	0
100285097	LOC100285097	RING-H2 finger protein ATL2B	0	0	0	0	0	0	0	0	1,8601642	0,025661
100281635	LOC100281635	RING-H2 finger protein ATL2K	0	0	0	0	2,9350455	0,0128865	3,64124878	0,002303	3,1533983	0,0067963
103637698	LOC103637698	RING-H2 finger protein ATL8	-5,3569011	0,000865	-5,1488692	0,0007141	-6,495733	0,0001467	0	0	0	0
100191739	IDP774	RmlC-like cupins superfamily protein	1,54575048	0,029509	0	0	0	0	0	0	0	0
542653	pco117817(260)	RmlC-like cupins superfamily protein	0	0	0	0	3,3826472	0,0208495	3,10264622	0,025809	0	0
103625741	LOC103625741	RMUA	0	0	0	0	2,3183555	0,0115118	0	0	2,0016091	0,0194137
100274116	pco109284	RNA binding (RRM/RBD/RNP motifs) family protein	0	0	0	0	-2,141752	0,0048836	-1,5587673	0,042003	0	0
845225	rpoB	RNA polymerase beta subunit	0	0	0	0	-2,717321	0,0116308	0	0	0	0
845226	rpoC1	RNA polymerase beta' subunit	-2,026898	0,049792	0	0	0	0	0	0	0	0
100272267	LOC100272267	RNA polymerase II transcription elongation factor	0	0	0	0	2,1702771	0,0473842	2,243794	0,034949	0	0
103647913	LOC103647913	RNA pseudouridine synthase 6, chloroplastic	0	0	-2,7036569	0,0421998	0	0	0	0	0	0

			Yuc x Ctr		Azo x Ctr		AzoYuc x Ctr		AzoYuc x Yuc		AzoYuc x Azo	
Gene ID	Gene Symbol	Gene Description	Log2(FC)	pvalue	Log2(FC)	pvalue	Log2(FC)	pvalue	Log2(FC)	pvalue	Log2(FC)	pvalue
100217137	LOC100217137	RNA pseudouridine synthase 7	-4,146505	7,73E-05	-4,3067833	3,859E-05	-2,965885	0,0028235	0	0	0	0
100272487	cl32255_1	RNA-binding (RRM/RBD/RNP motifs) family protein	0	0	0	0	-1,783221	0,0409838	0	0	-1,962351	0,021102
100272319	cl41174_1d	RNA-binding (RRM/RBD/RNP motifs) family protein	-2,0245699	0,044869	0	0	-2,347463	0,0204599	0	0	0	0
100501435	LOC100501435	RNA-binding (RRM/RBD/RNP motifs) family protein	0	0	0	0	1,8285251	0,0394588	3,5345457	0,000263	2,6756678	0,0028848
100502234	LOC100502234	RNA-binding KH domain-containing protein	0	0	0	0	1,6924165	0,0365621	1,85616617	0,021331	1,8792244	0,0194983
542422	LOC542422	RNA-binding protein Nova-2	0	0	0	0	0	0	-1,5597144	0,006004	0	0
100216804	LOC100216804	RNA-binding protein-like protein	0	0	0	0	1,9544036	0,0139295	1,76631911	0,022298	0	0
100281821	LOC100281821	RNA-binding region RNP-1	1,91060969	0,024564	0	0	0	0	0	0	0	0
100037826	rld2	rolled leaf 2	0	0	0	0	0	0	0	0	1,5066857	0,0210046
100284560	LOC100284560	root cap protein 2	3,26679478	0,020867	0	0	0	0	0	0	0	0
103647148	LOC103647148	root phototropism protein 2	0	0	0	0	0	0	4,13308866	0,029722	0	0
100193109	si687016e08	Rop guanine nucleotide exchange factor 7	0	0	0	0	-2,731788	0,0319481	0	0	0	0
100217060	LOC100217060	ROTUNDIFOLIA like 8	3,45453928	0,049278	0	0	0	0	0	0	0	0
109945002	LOC109945002	rRNA 2'-O-methyltransferase fibrillarin-like	0	0	0	0	0	0	2,12387679	0,001101	1,6766359	0,0082878
100304408	LOC100304408	rRNA N-glycosidase	0	0	0	0	1,5406655	0,0276439	0	0	0	0
103637736	LOC103637736	*rth6 (roothairless6)*	-1,7366599	0,04232	0	0	0	0	2,27833168	0,006891	1,6706764	0,0416171
103641459	LOC103641459	rust resistance kinase Lr10	0	0	0	0	-3,970996	0,0169307	-3,3384327	0,045912	-3,301146	0,0473994
103632084	LOC103632084	SAC3 family protein B	0	0	0	0	-3,525465	0,0399008	0	0	0	0
100382943	LOC100382943	S-adenosyl-L-methionine-dependent methyltransferase superfamily protein	0	0	-3,4962379	0,0372389	0	0	-3,08068	0,017217	0	0
100191816	LOC100191816	S-alkyl-thiohydroximate lyase SUR1	0	0	-4,450569	0,0298999	0	0	0	0	0	0
100285359	LOC100285359	salt tolerance-like protein	0	0	1,93356035	0,041734	0	0	0	0	0	0
100284329	LOC100284329	SAM domain family protein	0	0	0	0	0	0	2,31480745	0,049926	0	0
109943421	LOC109943421	sanguinarine reductase	0	0	0	0	-3,799829	0,0434076	0	0	0	0
100281900	MYBR115	SANT/MYB protein	-4,2741744	0,026961	0	0	0	0	0	0	0	0
103652785	LOC103652785	saposin B domain-containing protein	0	0	2,52875244	0,0281702	0	0	0	0	0	0
103652878	LOC103652878	saposin B domain-containing protein	0	0	3,81310482	0,0274069	0	0	-3,552184	0,049962	-5,453914	0,0016062
100283638	LOC100283638	saposin-like type B, region 1 family protein	0	0	0	0	0	0	0	0	-3,313247	0,0008533
100285854	LOC100285854	SC3 protein	0	0	0	0	0	0	0	0	2,2911917	0,0374701
103655011	LOC103655011	scarecrow-like protein 9	3,7133716	0,040696	0	0	0	0	0	0	0	0
100284137	LOC100284137	sec20 family protein	3,06720205	0,019775	0	0	0	0	0	0	0	0
100192643	NAC103	secondary wall NAC transcription factor 4	0	0	0	0	0	0	0	0	-3,830422	0,0287056
100283018	LOC100283018	seed maturation protein	0	0	0	0	-2,045339	0,0061508	0	0	-2,136231	0,0038738

			Yuc x Ctr		Azo x Ctr		AzoYuc x Ctr		AzoYuc x Yuc		AzoYuc x Azo	
Gene ID	Gene Symbol	Gene Description	Log2(FC)	pvalue	Log2(FC)	pvalue	Log2(FC)	pvalue	Log2(FC)	pvalue	Log2(FC)	pvalue
100217128	LOC100217128	seed maturation protein PM41	0	0	0	0	2,2736181	0,0307261	3,45307272	0,002317	0	0
100285188	LOC100285188	seed maturation protein PM41	0	0	0	0	0	0	0	0	-3,325252	0,0464883
100282086	LOC100282086	seed specific protein Bn15D17A	1,82237624	0,007686	0	0	1,6734877	0,0141565	0	0	0	0
100285126	LOC100285126	selT-like protein	-2,1937122	0,040575	0	0	0	0	0	0	0	0
100276582	LOC100276582	senescence regulator	0	0	0	0	-2,778821	0,0321445	0	0	0	0
100277341	LOC100277341	senescence regulator	0	0	0	0	0	0	0	0	-2,621188	0,0249233
100285592	LOC100285592	senescence-associated protein 15	0	0	0	0	0	0	1,77100653	0,028328	0	0
100192995	LOC100192995	Senescence-associated protein 5	-2,8280179	0,0304	0	0	0	0	0	0	0	0
103638253	LOC103638253	senescence-specific cysteine protease SAG39	-4,3815485	0,015426	0	0	-4,637828	0,0103503	0	0	0	0
541958	sat1	serine acetyltransferase 1	0	0	0	0	-4,260098	0,0237958	0	0	-3,804506	0,042556
103630156	LOC103630156	serine carboxypeptidase 2	0	0	0	0	2,2936941	0,0490019	0	0	0	0
103639025	LOC103639025	serine carboxypeptidase-like 51	0	0	2,28345707	0,0268353	0	0	0	0	0	0
100281777	LOC100281777	serine esterase family protein	0	0	0	0	2,3444935	0,0203707	2,23582592	0,023153	2,2382945	0,021949
100285688	LOC100285688	serine esterase family protein	4,19741488	0,000435	0	0	0	0	-3,8925492	0,00052	0	0
100191532	LOC100191532	serine hydroxymethyltransferase	2,29614169	0,0126	0	0	0	0	0	0	0	0
109944989	LOC109944989	serine hydroxymethyltransferase 4	0	0	0	0	0	0	0	0	-3,038019	0,004045
100194272	LOC100194272	Serine/arginine-rich splicing factor SR45a	0	0	0	0	0	0	2,37014854	0,031874	0	0
100384234	LOC100384234	Serine/threonine protein phosphatase 2A regulatory subunit B"alpha	0	0	-2,1586896	0,0410656	0	0	0	0	2,4795922	0,0179713
100382597	LOC100382597	Serine/threonine-protein kinase ATG1c	0	0	1,99698515	0,0159705	0	0	0	0	0	0
103645957	LOC103645957	serine/threonine-protein kinase CTR1	0	0	0	0	0	0	0	0	1,6727155	0,0379349
100281277	LOC100281277	serine/threonine-protein kinase NAK	0	0	0	0	0	0	2,27133519	0,019232	0	0
100281460	LOC100281460	serine/threonine-protein kinase NAK	0	0	0	0	2,360255	0,005758	2,49070048	0,00303	2,2534194	0,0061394
103626047	LOC103626047	serine/threonine-protein kinase Nek2	0	0	0	0	0	0	0	0	4,1641619	0,0124193
100274512	pco126351	Serine/threonine-protein kinase SRK2A	-2,5307549	0,006088	-2,3413817	0,0101294	-2,564649	0,0050797	0	0	0	0
103630308	LOC103630308	serine/threonine-protein kinase-like protein At3g51990	0	0	0	0	2,1746327	0,0260323	0	0	2,9778533	0,0025186
103652899	LOC103652899	serine/threonine-protein kinase-like protein CCR4	0	0	0	0	1,7724799	0,0158542	0	0	0	0
103652598	LOC103652598	serine/threonine-protein phosphatase 6 regulatory subunit 2-like	0	0	0	0	0	0	1,54287533	0,03188	1,8399198	0,0108028
100283219	LOC100283219	serine-type endopeptidase inhibitor	0	0	-4,0455679	0,0195983	0	0	0	0	0	0
100273136	si660046c04	Serpin-Z1	0	0	0	0	2,3870943	0,0117893	0	0	0	0
542678	SET102	SET domain-containing protein SET102	0	0	0	0	0	0	1,90204099	0,032888	0	0
100192507	LOC100192507	Short-chain dehydrogenase TIC 32 chloroplastic	0	0	0	0	-3,364754	0,0339694	0	0	0	0

			Yuc x Ctr		Azo x Ctr		AzoYuc x Ctr		AzoYuc x Yuc		AzoYuc x Azo	
Gene ID	Gene Symbol	Gene Description	Log2(FC)	pvalue	Log2(FC)	pvalue	Log2(FC)	pvalue	Log2(FC)	pvalue	Log2(FC)	pvalue
100280491	LOC100280491	short-chain dehydrogenase/reductase SDR	0	0	0	0	3,1592769	0,0119418	0	0	3,3665941	0,0063848
732745	TIDP3620	sialyltransferase like protein	0	0	0	0	0	0	1,98589833	0,02657	0	0
103646246	LOC103646246	sialyltransferase-like protein 3	0	0	2,27833833	0,0310159	0	0	0	0	0	0
100280752	LOC100280752	signal recognition particle 14 kDa protein	0	0	0	0	0	0	2,42812333	0,007729	2,6718114	0,003431
100193747	LOC100193747	signal transducer	-1,6117108	0,037415	0	0	0	0	0	0	0	0
103637625	LOC103637625	silicon efflux transporter LSI3	0	0	2,35821295	0,0044617	0	0	0	0	0	0
100502546	Lsi2	silicon transporter	2,31954158	0,00944	0	0	2,3120356	0,0095613	0	0	2,4016558	0,0060336
103652570	LOC103652570	skin secretory protein xP2-like	0	0	-2,7097488	0,0093186	0	0	0	0	2,2879149	0,0271488
109945805	LOC109945805	skin secretory protein xP2-like	0	0	0	0	1,6811392	0,0206231	0	0	0	0
103649936	LOC103649936	small G protein signaling modulator 1	2,36854214	0,012228	0	0	0	0	-2,6823524	0,003969	0	0
100284147	LOC100284147	small nuclear ribonucleoprotein F	0	0	0	0	2,812253	0,0219926	0	0	3,8374179	0,0027021
LOC111590745	LOC111590745	small nucleolar RNA F1/F2/snoR5a	0	0	0	0	-3,548369	0,0315343	0	0	-3,502207	0,0310206
LOC111590746	LOC111590746	small nucleolar RNA F1/F2/snoR5a	0	0	0	0	-1,829305	0,0381194	0	0	0	0
111590093	LOC111590093	small nucleolar RNA R24	-1,5200027	0,029001	0	0	0	0	0	0	0	0
LOC111591432	LOC111591432	small nucleolar RNA snoR60	0	0	-2,7922205	0,0446299	0	0	0	0	0	0
111589459	LOC111589459	small nucleolar RNA snoR77	0	0	0	0	0	0	-1,8362991	0,02783	-2,024614	0,0145649
111589585	LOC111589585	small nucleolar RNA SNORD14	-4,024711	0,018651	-3,4864436	0,0270412	-5,00338	0,005023	0	0	0	0
111589806	LOC111589806	small nucleolar RNA SNORD34	0	0	0	0	0	0	0	0	-3,329172	0,045391
111589909	LOC111589909	small nucleolar RNA SNORD96 family	0	0	0	0	0	0	1,76023364	0,000837	0	0
111589818	LOC111589818	small nucleolar RNA U54	2,30977578	0,008698	0	0	0	0	-1,7380483	0,036521	0	0
111589381	LOC111589381	small nucleolar RNA Z103	-3,1994286	0,005573	0	0	0	0	0	0	0	0
111589383	LOC111589383	small nucleolar RNA Z103	0	0	0	0	-1,636554	0,0062089	0	0	0	0
111589804	LOC111589804	small nucleolar RNA Z105	4,43566348	0,005572	0	0	0	0	-3,7322949	0,006383	0	0
111590335	LOC111590335	small nucleolar RNA Z118/Z121/Z120	0	0	0	0	1,8223896	0,0088094	0	0	0	0
111590340	LOC111590340	small nucleolar RNA Z118/Z121/Z120	2,92602088	0,018923	0	0	0	0	0	0	0	0
LOC111591026	LOC111591026	small nucleolar RNA Z221/R21b	0	0	0	0	0	0	-2,0786099	0,021843	0	0
LOC111591051	LOC111591051	small nucleolar RNA Z266	0	0	0	0	0	0	-1,5743484	0,042465	0	0
100193976	cl42326_1	SNARE associated Golgi protein family	0	0	0	0	0	0	-2,4187307	0,005087	0	0
100273321	LOC100273321	SNARE associated Golgi protein family	0	0	0	0	2,8254673	0,0134842	2,92785282	0,009323	2,7161807	0,0142363
100272928	pco123983	SNARE associated Golgi protein family	0	0	0	0	-1,841575	0,0352907	0	0	0	0
542687	SnrK1	SNF1-related protein kinase	0	0	0	0	0	0	0	0	1,8931951	0,0173563
103647175	LOC103647175	snrk1a2 (SNF1-related kinase alpha1-like2) - GRMZM2G180704	1,57178881	0,049207	0	0	0	0	-1,8709178	0,017693	0	0

			Yuc x Ctr		Azo x Ctr		AzoYuc x Ctr		AzoYuc x Yuc		AzoYuc x Azo	
Gene ID	Gene Symbol	Gene Description	Log2(FC)	pvalue	Log2(FC)	pvalue	Log2(FC)	pvalue	Log2(FC)	pvalue	Log2(FC)	pvalue
100285804	LOC100285804	SNF1-related protein kinase regulatory subunit beta-1	0	0	0	0	0	0	0	0	2,5147345	0,0425254
103634586	LOC103634586	SNF1-related protein kinase regulatory subunit beta-1	0	0	0	0	-2,044417	0,0310297	0	0	0	0
100274253	si707014d06	SNF7 family protein	0	0	0	0	2,3330791	0,0229726	2,95686912	0,004327	3,3390768	0,001413
103625710	LOC103625710	S-norcoclaurine synthase 1	1,59815086	0,010419	0	0	0	0	0	0	0	0
542481	LOC542481	soluble starch synthase 2-3, chloroplastic/amyloplastic	0	0	0	0	4,1230226	0,0186176	0	0	4,7716893	0,0064631
542670	LOC542670	somatic embryogenesis receptor-like kinase 3	0	0	0	0	1,8203774	0,003185	0	0	0	0
100277081	si614027e10	Sorting nexin 2B	3,51445284	0,022799	0	0	0	0	0	0	0	0
103648962	LOC103648962	spidroin-1-like	0	0	0	0	4,0746117	0,0472353	0	0	0	0
109942577	LOC109942577	spidroin-1-like	0	0	-2,5045351	0,015729	0	0	0	0	2,9933157	0,0032844
109942764	LOC109942764	spidroin-1-like	0	0	-3,8667867	0,026515	0	0	0	0	0	0
109945825	LOC109945825	spidroin-1-like	0	0	0	0	4,6303461	0,0432009	0	0	0	0
103632101	LOC103632101	splicing factor 3B subunit 3	0	0	0	0	0	0	0	0	1,9426755	0,0099387
100216718	LOC100216718	SPX domain-containing protein 4	0	0	0	0	2,170317	0,0195947	2,10854104	0,020714	2,0074099	0,0261782
100279234	LOC100279234	squamosa promoter-binding protein-like (SBP domain) transcription factor family protein	0	0	0	0	2,2973939	0,017472	0	0	2,6319985	0,0059905
103637478	LOC103637478	squamosa promoter-binding-like protein 2	-1,6566031	0,04928	0	0	0	0	0	0	0	0
100304375	srk1	S-receptor kinase 1	0	0	0	0	0	0	-1,7661981	0,020294	0	0
100281190	LOC100281190	stachyose synthase	0	0	0	0	0	0	0	0	-2,879822	0,0084942
542342	LOC542342	starch branching enzyme 3	-1,9981665	0,009423	0	0	-2,796318	0,000556	0	0	0	0
100170250	LOC100170250	starch synthase V	0	0	0	0	0	0	0	0	1,9700326	0,0395978
103629895	LOC103629895	STOREKEEPER protein	0	0	0	0	0	0	2,15855048	0,021095	0	0
100284893	LOC100284893	stress regulated protein	0	0	-1,5572168	0,0083469	0	0	0	0	0	0
542299	sip1	stress-induced protein 1	0	0	0	0	0	0	0	0	-4,295044	0,0356751
100286246	gpm462	Stress-inducible membrane pore protein	0	0	0	0	0	0	0	0	-4,813616	0,0461159
103650436	LOC103650436	stromal 70 kDa heat shock-related protein, chloroplastic-like	0	0	0	0	-1,520566	0,0136559	-1,8909368	0,001899	0	0
100280933	LOC100280933	subtilisin-chymotrypsin inhibitor CI-1B	-2,8574596	0,03034	0	0	0	0	0	0	0	0
100282452	LOC100282452	subtilisin-chymotrypsin inhibitor CI-1B	-2,6683377	0,015399	0	0	0	0	0	0	0	0
103629592	LOC103629592	subtilisin-like protease	-2,2879965	0,033606	-2,0858089	0,0486636	-2,818581	0,0099165	0	0	0	0
107197951	LOC107197951	Subtilisin-like protease	0	0	1,59781803	0,0271137	0	0	0	0	-1,626445	0,0170078
103646447	LOC103646447	subtilisin-like protease SBT1.7	0	0	0	0	2,1219101	0,0059788	1,51805247	0,04461	1,6299495	0,0308926

			Yuc x Ctr		Azo x Ctr		AzoYuc x Ctr		AzoYuc x Yuc		AzoYuc x Azo	
Gene ID	Gene Symbol	Gene Description	Log2(FC)	pvalue	Log2(FC)	pvalue	Log2(FC)	pvalue	Log2(FC)	pvalue	Log2(FC)	pvalue
103638067	LOC103638067	succinate dehydrogenase assembly factor 1, mitochondrial	-3,9420648	0,028852	0	0	0	0	0	0	0	0
542091	sus2	sucrose synthase 2	0	0	0	0	0	0	0	0	-2,988562	0,0112523
103634623	LOC103634623	sucrose synthase 4	0	0	0	0	0	0	3,58965376	0,00447	0	0
542661	LOC542661	sucrose-phosphatase	0	0	0	0	2,5243742	0,0024293	0	0	2,3411883	0,0037154
100282631	IDP152	sugars will eventually be exported transporter6a	-3,6875383	0,044225	0	0	0	0	0	0	0	0
100284487	LOC100284487	sulfate transporter	0	0	0	0	2,4600092	0,0336916	4,25542632	0,001424	3,1834118	0,0063324
100281787	LOC100281787	sulfate transporter 3.4	0	0	0	0	3,120377	0,0014019	2,95735596	0,00178	2,6143434	0,0047896
542722	sod4	superoxide dismutase 4	0	0	0	0	1,5493601	0,0206813	1,57589214	0,017263	1,6207064	0,0139818
100278739	cl5119_-3c	survival motor neuron protein	0	0	-4,2614893	0,0124141	0	0	0	0	4,6527672	0,0049809
100285646	LOC100285646	syntaxin 132	2,10279274	0,035394	0	0	0	0	0	0	0	0
100192511	LOC100192511	Syntaxin 32	0	0	-1,5918378	0,0431098	-1,558126	0,0467754	0	0	0	0
100283387	LOC100283387	syntaxin 32	-4,0303449	0,000703	-4,1102714	0,0005065	-4,348272	0,0002692	0	0	0	0
100283626	LOC100283626	syntaxin-related protein KNOLLE	0	0	0	0	-3,476618	0,0152316	0	0	-2,819305	0,0492793
100384575	TCP12	TCP transcription factor	0	0	0	0	2,2194774	0,0268196	2,60635741	0,009217	2,0159731	0,0374853
111589230	LOC111589230	telomere-binding protein 1-like	0	0	0	0	0	0	2,74256184	0,017082	2,1071765	0,047242
100286188	pco085164	Temperature-induced lipocalin-1	-4,8276399	0,005998	0	0	-4,122125	0,0189602	0	0	-3,966447	0,0228907
103634194	LOC103634194	testis-specific gene A8 protein-like	0	0	0	0	0	0	3,15535653	0,049694	0	0
100383407	LOC100383407	Tetraspanin-2	0	0	3,24867931	0,0353829	3,5267447	0,0216059	0	0	0	0
100193007	LOC100193007	Tetratricopeptide repeat (TPR)-like superfamily protein	-2,0038378	0,036439	0	0	0	0	0	0	0	0
100383987	pco063627	Tetratricopeptide repeat (TPR)-like superfamily protein	0	0	0	0	0	0	3,3349271	0,012544	0	0
100384484	LOC100384484	Tetratricopeptide-like helical	0	0	0	0	4,2636858	0,0018715	2,52822478	0,031137	4,1073419	0,0011889
100281263	pco079405	Thaumatin-like protein	0	0	0	0	3,7942941	0,0026734	3,6852487	0,002546	3,5440559	0,0031515
103633630	LOC103633630	thaumatin-like protein 1	0	0	0	0	0	0	0	0	4,8690077	0,0044449
100275585	LOC100275585	thiaminase2	0	0	0	0	0	0	1,50059433	0,044706	1,8140722	0,0158403
542715	thi2	thiamine biosynthesis 2	0	0	0	0	0	0	1,61005241	0,000497	0	0
100285440	cl574_1	thic1 (hydroxymethylpyrimidine phosphate synthase1)	-4,4648392	0,006192	0	0	0	0	4,83475549	0,00243	0	0
100217079	LOC100217079	Thioesterase family protein	0	0	0	0	0	0	1,50490068	0,008874	0	0
542748	umc2618	Thioesterase family protein, mRNA	-2,3910094	0,001142	-2,7761194	0,0001744	-3,456872	5,287E-06	0	0	0	0
100381995	LOC100381995	Thioesterase superfamily protein	0	0	0	0	0	0	0	0	4,419739	0,030525
100281886	LOC100281886	thiol protease SEN102	0	0	0	0	1,5990107	0,0288368	0	0	0	0
100273825	IDP532	Thioredoxin	0	0	0	0	-4,77986	0,0036105	0	0	-4,292762	0,0087136
100303914	LOC100303914	thioredoxin H-type 5	0	0	0	0	2,5062917	0,0324755	0	0	0	0
100193002	LOC100193002	Thioredoxin-like 3-2 chloroplastic	2,06566285	0,035384	0	0	0	0	0	0	0	0
100280892	LOC100280892	thioredoxin-like protein 4B	0	0	0	0	-2,232058	0,0427458	0	0	0	0
100280051	LOC100280051	thioredoxin-like protein 5	0	0	0	0	2,9937047	0,0070195	2,76821306	0,010817	3,5357664	0,0014084
103646316	LOC103646316	thyroid receptor-interacting protein 6	0	0	1,82911822	0,0483618	0	0	0	0	0	0

			Yuc x Ctr		Azo x Ctr		AzoYuc x Ctr		AzoYuc x Yuc		AzoYuc x Azo	
Gene ID	Gene Symbol	Gene Description	Log2(FC)	pvalue	Log2(FC)	pvalue	Log2(FC)	pvalue	Log2(FC)	pvalue	Log2(FC)	pvalue
100192992	LOC100192992	TLD-domain containing nucleolar protein	-2,5234826	0,001439	0	0	-2,135307	0,0045805	0	0	-1,868425	0,0124689
100285926	LOC100285926	TMV response-related protein	0	0	0	0	-2,007852	0,0421676	0	0	0	0
100383391	pco128969	Tonoplast dicarboxylate transporter	0	0	0	0	0	0	2,88917192	0,047087	0	0
541897	tip4a	tonoplast intrinsic protein 4	0	0	0	0	3,5269442	0,0312918	0	0	0	0
100191474	Top1	topoisomerase I	0	0	0	0	1,737763	0,0076897	0	0	1,7648819	0,0060479
100502223	LOC100502223	TP53-regulating kinase	0	0	0	0	3,2755714	0,0268363	3,05610812	0,028323	2,6910697	0,0447249
100273959	LOC100273959	trafficking protein particle complex subunit 2-like protein	0	0	0	0	0	0	-1,5035346	0,048393	0	0
100285232	LOC100285232	trafficking protein particle complex subunit 3	0	0	0	0	-2,162545	0,003969	-1,5809761	0,036623	0	0
100383633	LOC100383633	transcription associated factor1	0	0	0	0	0	0	0	0	3,3596282	0,0013435
103641361	LOC103641361	transcription factor APG	0	0	-3,4336381	0,0226822	0	0	0	0	4,191813	0,0044777
103627392	LOC103627392	transcription factor bHLH128	0	0	0	0	0	0	0	0	1,7039366	0,0145425
103634500	LOC103634500	transcription factor bHLH140	0	0	0	0	2,7456313	0,0215093	2,05345025	0,044662	0	0
103632755	LOC103632755	transcription factor bHLH69	0	0	0	0	0	0	0	0	2,5955333	0,0401604
103637126	LOC103637126	transcription factor DIVARICATA	0	0	0	0	0	0	1,6508252	0,037333	0	0
103641578	LOC103641578	transcription factor FER-LIKE IRON DEFICIENCY-INDUCED TRANSCRIPTION FACTOR	0	0	0	0	1,6695638	0,0390478	0	0	1,6668814	0,0373194
103647221	LOC103647221	transcription factor FER-LIKE IRON DEFICIENCY-INDUCED TRANSCRIPTION FACTOR	0	0	0	0	0	0	-1,6781367	0,018009	0	0
103641126	LOC103641126	transcription factor GTE7	0	0	0	0	0	0	1,9545004	0,039573	0	0
103642827	LOC103642827	transcription factor MYB2	0	0	0	0	-2,66428	0,0238773	0	0	-2,418242	0,0393946
103632720	LOC103632720	transcription factor MYB20	0	0	0	0	0	0	0	0	-3,743231	0,0367798
103638687	LOC103638687	transcription factor MYB29	14,863536	3,87E-05	12,4784653	0,0007962	20,987683	2,755E-09	0	0	8,5091881	0,0159278
103628080	LOC103628080	transcription factor MYB30	0	0	0	0	0	0	0	0	4,6159834	0,0224811
541743	LOC541743	transcription factor MYB30	1,50428392	0,044017	0	0	0	0	0	0	0	0
100101513	myb42	transcription factor MYB42	2,83099416	0,039417	0	0	0	0	0	0	0	0
103638892	LOC103638892	transcription factor MYC2	2,08420953	0,038283	0	0	0	0	0	0	0	0
109941404	LOC109941404	transcription factor NIGTH1-like	0	0	0	0	1,8969384	0,0461087	3,21156967	0,001278	2,6887787	0,0050205
109940870	LOC109940870	transcription factor PCF6	0	0	0	0	0	0	0	0	1,8339693	0,0118795
103651274	LOC103651274	transcription factor SPATULA	0	0	0	0	0	0	0	0	4,8087303	0,0022749
100276975	LOC100276975	transcription factor UPBEAT1	0	0	0	0	0	0	0	0	3,6657614	0,0316208
100282260	LOC100282260	transcription factor/ transcription initiation factor	0	0	0	0	0	0	0	0	-2,008278	0,0322885
100383020	LOC100383020	Transcription factor-like protein DPB	-2,8558534	0,030711	0	0	-2,861953	0,0273147	0	0	0	0
103633132	LOC103633132	transcription initiation factor TFIID subunit 15	0	0	0	0	0	0	2,70861449	0,011436	0	0
103642210	LOC103642210	Transcription repressor OFP13	0	0	0	0	0	0	3,85020667	0,040349	0	0

			Yuc x Ctr		Azo x Ctr		AzoYuc x Ctr		AzoYuc x Yuc		AzoYuc x Azo	
Gene ID	Gene Symbol	Gene Description	Log2(FC)	pvalue	Log2(FC)	pvalue	Log2(FC)	pvalue	Log2(FC)	pvalue	Log2(FC)	pvalue
103647057	LOC103647057	Transcription repressor OFP6	0	0	2,0218866	0,0252325	1,9767189	0,0286462	0	0	0	0
100273788	LOC100273788	transcriptional regulator of RNA polII SAGA subunit	0	0	0	0	0	0	-4,0525965	0,000803	0	0
111589747	LOC111589747	transcriptional regulatory protein AlgP-like	0	0	0	0	2,1416149	0,01668	2,86796536	0,00182	1,6365072	0,0497978
103642006	LOC103642006	transducin beta-like protein 3	0	0	0	0	2,6868011	0,0125475	2,10781016	0,026214	0	0
100193557	LOC100193557	Transducin family protein / WD-40 repeat family protein	0	0	0	0	2,194927	0,0311004	1,9595796	0,043521	2,4501513	0,0131891
103634524	LOC103634524	transducin family protein / WD-40 repeat family protein	2,59022099	0,005248	0	0	0	0	0	0	0	0
100272560	LOC100272560	transferase	-2,2631223	0,000873	0	0	-1,851239	0,0049478	0	0	0	0
100284954	LOC100284954	transferase	0	0	0	0	2,1920238	0,0136727	1,75141915	0,039525	1,7667786	0,0365584
100281722	LOC100281722	transferase, transferring glycosyl groups	2,38734965	0,028623	0	0	0	0	0	0	0	0
103633702	LOC103633702	translation initiation factor IF-2	0	0	0	0	4,1091099	0,0403576	0	0	0	0
103636907	LOC103636907	translation initiation factor IF-2-like	0	0	0	0	0	0	0	0	-1,890645	0,0151342
109940571	LOC109940571	translation initiation factor IF-2-like	0	0	0	0	0	0	0	0	2,2633056	0,0327472
109941040	LOC109941040	translation initiation factor IF-2-like	0	0	0	0	0	0	2,53050795	0,031555	0	0
109941104	LOC109941104	translation initiation factor IF-2-like	-2,7737652	0,035973	-2,9546627	0,0249508	0	0	0	0	0	0
109942356	LOC109942356	translation initiation factor IF-2-like	0	0	0	0	0	0	0	0	2,1026903	0,0331272
109942462	LOC109942462	translation initiation factor IF-2-like	0	0	0	0	0	0	0	0	4,5057055	0,0440586
109942799	LOC109942799	translation initiation factor IF-2-like	0	0	1,63039451	0,0399518	1,7647064	0,0254711	0	0	0	0
109945058	LOC109945058	translation initiation factor IF-2-like	-3,4817726	0,032296	-3,1365876	0,0429154	-4,11127	0,0152209	0	0	0	0
109945814	LOC109945814	translation initiation factor IF-2-like	-1,8394248	0,044577	0	0	0	0	1,75740858	0,049628	0	0
100272490	LOC100272490	translin	-4,4374493	0,001142	-4,082755	0,0018127	-3,746171	0,0034521	0	0	0	0
103643094	LOC103643094	translocase of chloroplast 159, chloroplastic	1,54163902	0,004937	0	0	0	0	0	0	0	0
100273641	LOC100273641	translocon-associated protein alpha subunit	2,21772691	0,003712	0	0	0	0	-1,9502752	0,008414	0	0
100283273	LOC100283273	translocon-associated protein beta containing protein	0	0	0	0	-1,681541	0,009778	0	0	0	0
100193254	tap1	translocon-associated protein homolog 1	0	0	0	0	0	0	-1,7709108	0,028594	0	0
103631168	LOC103631168	transmembrane 9 superfamily member 11	0	0	0	0	0	0	1,58585317	0,037086	0	0
100273033	LOC100273033	Transmembrane amino acid transporter family protein	0	0	0	0	1,9777626	0,0421742	3,72909974	0,000462	3,0395385	0,0022682
100282793	LOC100282793	transmembrane BAX inhibitor motif-containing protein 4	0	0	-1,7947714	0,0278607	0	0	0	0	0	0
100194123	LOC100194123	Transmembrane emp24 domain-containing protein p24beta2	0	0	0	0	0	0	0	0	1,523383	0,0117533

			Yuc x Ctr		Azo x Ctr		AzoYuc x Ctr		AzoYuc x Yuc		AzoYuc x Azo	
Gene ID	Gene Symbol	Gene Description	Log2(FC)	pvalue	Log2(FC)	pvalue	Log2(FC)	pvalue	Log2(FC)	pvalue	Log2(FC)	pvalue
100281371	pco090197	Transmembrane emp24 domain-containing protein p24beta2	0	0	0	0	-1,781924	0,0076396	0	0	0	0
100283503	LOC100283503	Transposon protein	0	0	0	0	1,736189	0,0316927	0	0	0	0
107546758	LOC107546758	trehalose-6-phosphate phosphatase2	0	0	0	0	0	0	0	0	2,031168	0,0249295
100282902	LOC100282902	triacylglycerol lipase	0	0	0	0	0	0	0	0	2,4670733	0,0339358
100286304	LOC100286304	triacylglycerol lipase	0	0	0	0	0	0	2,59716577	0,02978	2,4595903	0,0376066
103651369	LOC103651369	trihelix transcription factor	0	0	0	0	0	0	2,13538974	0,016151	2,5385423	0,0044929
109943509	LOC109943509	trihelix transcription factor GTL1	0	0	1,54810282	0,0013753	0	0	0	0	0	0
100381749	LOC100381749	Triosephosphate isomerase	0	0	0	0	3,9391779	0,0333137	0	0	0	0
100192674	LOC100192674	tRNA/rRNA methyltransferase (SpoU) family protein	0	0	-1,8241066	0,0371177	0	0	0	0	0	0
103635013	LOC103635013	tRNase Z TRZ3, mitochondrial	0	0	0	0	0	0	1,53546318	0,039533	1,9535906	0,0102273
103652733	LOC103652733	tropinone reductase homolog At1g07440	0	0	0	0	1,9516723	0,0116715	1,63330196	0,028358	2,6060349	0,0007751
100383587	LOC100383587	Tryptophan aminotransferase-related protein 4	0	0	1,64585079	0,0161633	0	0	0	0	0	0
103653540	LOC103653540	tubulin beta-5 chain-like	0	0	0	0	-1,953197	0,0459336	0	0	0	0
100283359	LOC100283359	two-component response regulator ARR3	0	0	0	0	0	0	4,75889018	0,014428	0	0
541630	gpm84	Two-component response regulator ARR9	-2,1179145	0,039416	0	0	0	0	0	0	0	0
100285170	LOC100285170	two-component response regulator-like PRR95	-4,378324	0,000381	-2,8173203	0,0136248	-2,56094	0,0239178	0	0	0	0
103642551	LOC103642551	type II inositol polyphosphate 5-phosphatase 15	0	0	0	0	0	0	-3,7772986	0,028322	0	0
103626086	LOC103626086	tyrosine N-monooxygenase	0	0	0	0	2,70911	0,0095813	0	0	3,2580232	0,0018064
100283963	LOC100283963	tyrosine specific protein phosphatase family protein	0	0	0	0	3,0547506	0,0205244	0	0	3,084916	0,0154721
103625831	LOC103625831	Tyrosine specific protein phosphatase-like	0	0	0	0	0	0	3,4366152	0,036615	0	0
111589360	LOC111589360	U1 spliceosomal RNA	0	0	-2,1623504	0,0062947	0	0	0	0	0	0
111589443	LOC111589443	U1 spliceosomal RNA	0	0	0	0	3,2627431	0,0122765	0	0	0	0
111589688	LOC111589688	U1 spliceosomal RNA	0	0	0	0	2,1769959	0,008052	1,69741913	0,034336	0	0
111590063	LOC111590063	U1 spliceosomal RNA	0	0	2,41069377	0,0068605	2,3069736	0,0097043	0	0	0	0
111590227	LOC111590227	U1 spliceosomal RNA	0	0	0	0	1,6479626	0,003615	0	0	0	0
111590367	LOC111590367	U1 spliceosomal RNA	0	0	0	0	2,0210063	0,0402346	0	0	0	0
111590422	LOC111590422	U1 spliceosomal RNA	0	0	0	0	2,8150739	0,0024025	1,9687595	0,022545	0	0
LOC111590787	LOC111590787	U1 spliceosomal RNA	0	0	0	0	1,9427441	0,0003975	0	0	0	0
LOC111590945	LOC111590945	U1 spliceosomal RNA	0	0	0	0	1,6177392	0,0011724	0	0	0	0
LOC111590946	LOC111590946	U1 spliceosomal RNA	0	0	0	0	1,7338548	0,0005218	0	0	0	0
LOC111590947	LOC111590947	U1 spliceosomal RNA	0	0	0	0	1,8765045	0,0003759	0	0	0	0

			Yuc x Ctr		Azo x Ctr		AzoYuc x Ctr		AzoYuc x Yuc		AzoYuc x Azo	
Gene ID	Gene Symbol	Gene Description	Log2(FC)	pvalue	Log2(FC)	pvalue	Log2(FC)	pvalue	Log2(FC)	pvalue	Log2(FC)	pvalue
LOC111591372	LOC111591372	U1 spliceosomal RNA	0	0	1,5914256	0,047805	0	0	0	0	0	0
100279189	LOC100279189	U11/U12 small nuclear ribonucleoprotein 48 kDa protein	2,60788241	0,046831	0	0	0	0	0	0	0	0
111590057	LOC111590057	U2 spliceosomal RNA	0	0	0	0	0	0	1,72117581	0,02241	0	0
111590216	LOC111590216	U2 spliceosomal RNA	0	0	0	0	3,4234511	0,0027641	2,43014431	0,013956	3,0786816	0,0026084
111590221	LOC111590221	U2 spliceosomal RNA	0	0	0	0	2,0654194	0,0204279	0	0	0	0
111590249	LOC111590249	U2 spliceosomal RNA	0	0	0	0	1,904585	0,002102	0	0	0	0
111590260	LOC111590260	U2 spliceosomal RNA	0	0	0	0	1,6917089	0,0200985	0	0	0	0
LOC111590661	LOC111590661	U2 spliceosomal RNA	0	0	0	0	1,7842519	0,022847	0	0	1,8196938	0,0162796
LOC111590782	LOC111590782	U2 spliceosomal RNA	0	0	0	0	0	0	0	0	4,1432768	0,0114965
LOC111591262	LOC111591262	U2 spliceosomal RNA	0	0	0	0	1,5238125	0,0178391	0	0	0	0
LOC111591461	LOC111591461	U2 spliceosomal RNA	0	0	0	0	1,7054001	0,0007118	0	0	0	0
111590330	LOC111590330	U5 spliceosomal RNA	0	0	2,08651955	0,0084214	0	0	0	0	-1,798189	0,0196097
111590038	LOC111590038	U6atac minor spliceosomal RNA	0	0	0	0	1,5560058	0,0022886	0	0	0	0
100283539	LOC100283539	ubiquinone biosynthesis protein COQ9	-1,9454599	0,044064	0	0	0	0	0	0	0	0
103645851	LOC103645851	ubiquitin carboxyl-terminal hydrolase 22	0	0	0	0	0	0	0	0	4,3427365	0,0140155
100282112	cl26792_1(160)	Ubiquitin system component Cue protein	0	0	0	0	0	0	0	0	1,5364653	0,0495002
103630718	LOC103630718	ubiquitin-conjugating enzyme E2	0	0	1,74066876	0,0386649	0	0	0	0	0	0
100284000	LOC100284000	ubiquitin-conjugating enzyme E2 16	0	0	0	0	0	0	1,88484904	0,049943	0	0
100192052	LOC100192052	Ubiquitin-conjugating enzyme E2 2	0	0	0	0	0	0	-1,8471054	0,02721	0	0
100284762	pco096181	Ubiquitin-conjugating enzyme E2 2	-1,5755762	0,016651	0	0	-1,951581	0,0031237	0	0	0	0
100282170	umc107b(croc)	Ubiquitin-conjugating enzyme E2 variant 1A	-3,8540804	0,000531	-4,1868814	0,0001933	-3,37717	0,0014029	0	0	0	0
542600	ubc7	ubiquitin-conjugating enzyme protein E2	0	0	0	0	0	0	0	0	1,6218444	0,0160467
103647934	LOC103647934	ubiquitin-like-specific protease ESD4	0	0	0	0	2,8699907	0,0267226	0	0	0	0
100281562	LOC100281562	ubiquitin-protein ligase	0	0	2,96175207	0,020821	0	0	0	0	-2,184539	0,0476522
100285280	LOC100285280	ubiquitin-protein ligase CIP8	0	0	0	0	0	0	3,57211783	0,009995	3,7383883	0,006542
103637786	LOC103637786	U-box domain-containing protein 21	0	0	0	0	0	0	0	0	3,4314258	0,0337544
100193510	LOC100193510	U-box domain-containing protein 34	-6,7347313	0,000423	-7,8001324	7,675E-05	-6,788669	0,0003536	0	0	0	0
103632728	LOC103632728	U-box domain-containing protein 35	0	0	-1,5802454	0,0229933	-1,516694	0,027978	0	0	0	0
103641784	LOC103641784	U-box domain-containing protein 7	0	0	0	0	2,2617585	0,0394205	0	0	2,7561278	0,010524
103641314	LOC103641314	U-box domain-containing protein 8	-2,4489941	0,024917	-2,1719404	0,0405751	-2,163391	0,0407722	0	0	0	0
542127	LOC542127	UDP-glucose-4-epimerase	0	0	2,32024992	0,0207006	0	0	0	0	-2,420152	0,0139369
103629864	LOC103629864	UDP-glucuronate 4-epimerase 1	0	0	0	0	0	0	0	0	4,1487213	0,0338336
100286027	cl108_1	UDP-glucuronate:xylan alpha-glucuronosyltransferase 1	0	0	0	0	2,8572017	0,0004771	3,00897462	0,000216	2,7505394	0,000654

			Yuc x Ctr		Azo x Ctr		AzoYuc x Ctr		AzoYuc x Yuc		AzoYuc x Azo	
Gene ID	Gene Symbol	Gene Description	Log2(FC)	pvalue	Log2(FC)	pvalue	Log2(FC)	pvalue	Log2(FC)	pvalue	Log2(FC)	pvalue
103627281	LOC103627281	UDP-glucuronate:xylan alpha-glucuronosyltransferase 1	0	0	0	0	1,7063237	0,0135738	0	0	0	0
103645214	LOC103645214	UDP-glucuronic acid decarboxylase 6	0	0	0	0	4,0649442	0,0342262	4,48767943	0,019408	0	0
103649679	LOC103649679	UDP-glycosyltransferase 73E1	0	0	0	0	0	0	-2,4553935	0,004263	0	0
100274286	umc2700	UDP-glycosyltransferase 75B1	0	0	2,64421792	0,0158492	0	0	0	0	0	0
103649471	LOC103649471	UDP-glycosyltransferase 76B1	0	0	0	0	0	0	0	0	3,2716109	0,0420564
100382222	LOC100382222	UDP-glycosyltransferase 85A7	0	0	0	0	0	0	0	0	-2,634167	0,0214446
100501354	LOC100501354	UDP-glycosyltransferase 85A7	0	0	0	0	-4,365592	0,0437159	0	0	0	0
103654463	LOC103654463	UDP-glycosyltransferase 89B2	0	0	-3,6131424	9,347E-06	-3,462087	1,491E-05	-3,8135702	1,47E-06	0	0
100272814	gpm263	UDP-glycosyltransferase 91A1	2,60843234	0,002654	0	0	1,8731499	0,0317968	0	0	0	0
100217302	LOC100217302	UDP-Glycosyltransferase superfamily protein	0	0	0	0	-1,920432	0,041906	0	0	0	0
100282472	LOC100282472	ufm1-conjugating enzyme 1	0	0	-1,9230574	0,0327315	0	0	0	0	0	0
100216658	LOC100216658	Uncharacterized conserved protein UCP009193	0	0	0	0	0	0	1,954297	0,022667	0	0
103633290	LOC103633290	uncharacterized GPI-anchored protein At4g28100	-2,3673827	0,017256	0	0	0	0	0	0	0	0
100037785	gpm255	uncharacterized LOC100037785	0	0	0	0	0	0	0	0	-4,052054	0,0271821
100101546	TIDP3215	uncharacterized LOC100101546	0	0	0	0	0	0	-1,8840589	0,040348	0	0
100125658	umc1760	uncharacterized LOC100125658	2,7192843	0,006738	0	0	0	0	-1,9285993	0,043236	0	0
100170237	pco064225	uncharacterized LOC100170237	2,50341273	0,041543	3,02332503	0,0126624	0	0	0	0	0	0
100191163	LOC100191163	uncharacterized LOC100191163	0	0	0	0	3,468039	0,0004172	3,26882755	0,000641	3,4270449	0,0003392
100191176	LOC100191176	uncharacterized LOC100191176	3,68398079	0,02731	3,36730939	0,0443208	3,5673472	0,031966	0	0	0	0
100191184	AI665898	uncharacterized LOC100191184	0	0	0	0	0	0	-1,8231522	0,046692	-2,715447	0,0023795
100191196	umc1979	uncharacterized LOC100191196	0	0	0	0	4,3420505	0,039992	0	0	0	0
100191198	si606063g10	uncharacterized LOC100191198	0	0	0	0	0	0	1,50434819	0,028457	1,6168028	0,0179616
100191203	pco137008b	uncharacterized LOC100191203	2,49947534	0,00709	0	0	0	0	-1,9615023	0,026608	0	0
100191221	LOC100191221	uncharacterized LOC100191221	0	0	0	0	1,8069459	0,0494153	3,10797197	0,002094	0	0
100191249	LOC100191249	uncharacterized LOC100191249	0	0	0	0	0	0	0	0	-4,211053	0,0110796
100191422	LOC100191422	uncharacterized LOC100191422	0	0	3,68464325	0,0048572	0	0	0	0	-2,693634	0,0216105
100191433	LOC100191433	uncharacterized LOC100191433	0	0	0	0	0	0	0	0	3,300904	0,0460443
100191451	pco120288	uncharacterized LOC100191451	0	0	0	0	0	0	3,92178316	0,041231	0	0
100191575	LOC100191575	uncharacterized LOC100191575	0	0	0	0	-1,771871	0,0459384	0	0	0	0
100191599	LOC100191599	uncharacterized LOC100191599	0	0	0	0	1,6322447	0,0271662	0	0	0	0
100191709	LOC100191709	uncharacterized LOC100191709	0	0	3,51067592	0,0402197	0	0	0	0	0	0
100191723	LOC100191723	uncharacterized LOC100191723	0	0	0	0	0	0	0	0	-1,912097	0,0462947
100191836	pco081433(10)	uncharacterized LOC100191836	0	0	0	0	-3,004534	0,0132622	0	0	-3,052576	0,0110337
100191866	LOC100191866	uncharacterized LOC100191866	0	0	-1,6931266	0,0456712	0	0	0	0	1,8503812	0,0243123
100191932	cl14781_1	uncharacterized LOC100191932	-1,5122202	0,009985	0	0	-1,597252	0,005831	0	0	0	0
100192117	IDP2565	uncharacterized LOC100192117	0	0	-1,65903	0,0204954	0	0	0	0	0	0
100192499	LOC100192499	uncharacterized LOC100192499	-3,2362563	0,002311	-3,9822247	0,0002267	-4,451093	5,049E-05	0	0	0	0
100192503	IDP753	uncharacterized LOC100192503	0	0	0	0	3,8204315	0,0018636	2,92756222	0,010775	3,6192405	0,0019909

			Yuc x Ctr		Azo x Ctr		AzoYuc x Ctr		AzoYuc x Yuc		AzoYuc x Azo	
Gene ID	Gene Symbol	Gene Description	Log2(FC)	pvalue	Log2(FC)	pvalue	Log2(FC)	pvalue	Log2(FC)	pvalue	Log2(FC)	pvalue
100192543	LOC100192543	uncharacterized LOC100192543	0	0	0	0	2,5635094	0,0045874	1,88010676	0,025416	2,1055639	0,0124122
100192551	LOC100192551	uncharacterized LOC100192551	-1,5807326	0,005116	0	0	0	0	0	0	0	0
100192676	LOC100192676	uncharacterized LOC100192676	0	0	-1,5594338	0,0468246	-1,748014	0,0268862	0	0	0	0
100192679	TIDP3001	uncharacterized LOC100192679	0	0	0	0	-1,89418	0,0009024	0	0	0	0
100192738	LOC100192738	uncharacterized LOC100192738	0	0	-1,794605	0,0463355	0	0	0	0	0	0
100192786	pco111209	uncharacterized LOC100192786	0	0	0	0	1,9175734	0,0420901	0	0	0	0
100192807	LOC100192807	uncharacterized LOC100192807	0	0	3,92974764	0,0086842	4,0269142	0,0070531	5,41143895	0,000294	0	0
100192834	LOC100192834	uncharacterized LOC100192834	0	0	0	0	2,3423419	0,0357265	0	0	0	0
100192870	LOC100192870	uncharacterized LOC100192870	0	0	0	0	0	0	0	0	-4,21134	0,02246
100193013	LOC100193013	uncharacterized LOC100193013	2,71787179	0,003629	0	0	0	0	-2,5897326	0,004043	0	0
100193067	LOC100193067	uncharacterized LOC100193067	0	0	0	0	3,9666277	0,0278839	0	0	0	0
100193119	pco130460	uncharacterized LOC100193119	1,81096506	0,013745	0	0	0	0	-1,8832719	0,009504	0	0
100193206	LOC100193206	uncharacterized LOC100193206	0	0	0	0	3,3856872	0,0492668	0	0	0	0
100193252	LOC100193252	uncharacterized LOC100193252	0	0	0	0	0	0	0	0	5,0217186	0,0094176
100193350	LOC100193350	uncharacterized LOC100193350	3,36090621	0,034486	0	0	0	0	0	0	0	0
100193369	LOC100193369	uncharacterized LOC100193369	-3,2626575	0,003778	-2,4467998	0,0205942	0	0	0	0	0	0
100193385	LOC100193385	uncharacterized LOC100193385	0	0	0	0	0	0	0	0	1,9384452	0,0405726
100193490	LOC100193490	uncharacterized LOC100193490	0	0	0	0	0	0	-3,4664518	0,001763	-3,479098	0,0016188
100193544	LOC100193544	uncharacterized LOC100193544	-3,2841419	0,000386	-2,6441292	0,0030073	-3,523499	0,0001383	0	0	0	0
100193572	LOC100193572	uncharacterized LOC100193572	0	0	0	0	0	0	-2,8996781	0,031779	0	0
100193630	LOC100193630	uncharacterized LOC100193630	0	0	0	0	4,5471526	0,0299826	0	0	0	0
100193631	pco148690(146)	uncharacterized LOC100193631	0	0	0	0	0	0	0	0	1,6607767	0,0229533
100193643	LOC100193643	uncharacterized LOC100193643	2,21026145	0,019306	0	0	0	0	-2,478156	0,008009	0	0
100193686	LOC100193686	uncharacterized LOC100193686	0	0	0	0	1,8819576	0,0152072	0	0	1,6336442	0,0251567
100193708	LOC100193708	uncharacterized LOC100193708	0	0	-2,7079814	0,0437984	0	0	0	0	0	0
100193792	LOC100193792	uncharacterized LOC100193792	0	0	0	0	-2,01352	0,0480728	0	0	0	0
100193832	LOC100193832	uncharacterized LOC100193832	0	0	0	0	3,1259235	0,011079	4,40486842	0,000675	4,4084008	0,0005553
100193845	LOC100193845	uncharacterized LOC100193845	-1,5442768	0,036804	0	0	0	0	0	0	0	0
100193903	LOC100193903	uncharacterized LOC100193903	0	0	0	0	-2,309042	0,0255338	0	0	0	0
100193962	LOC100193962	uncharacterized LOC100193962	2,3355842	0,01388	0	0	0	0	0	0	0	0
100193967	LOC100193967	uncharacterized LOC100193967	1,89503893	0,027866	0	0	0	0	0	0	0	0
100194035	LOC100194035	uncharacterized LOC100194035	0	0	0	0	0	0	0	0	-1,775621	0,0276304
100194075	IDP2402	uncharacterized LOC100194075	0	0	0	0	3,0544123	0,0082654	3,00867894	0,007927	3,4774241	0,0024095
100194144	LOC100194144	uncharacterized LOC100194144	0	0	0	0	0	0	-4,1048258	0,030382	0	0
100194194	LOC100194194	uncharacterized LOC100194194	0	0	0	0	1,8715262	0,0119801	1,52968385	0,035143	1,6092074	0,0262601
100194233	LOC100194233	uncharacterized LOC100194233	0	0	0	0	0	0	0	0	-3,260815	0,0427846
100194263	LOC100194263	uncharacterized LOC100194263	0	0	0	0	0	0	0	0	3,9474362	0,0403254
100194267	LOC100194267	uncharacterized LOC100194267	-2,1561859	0,049756	-2,4052136	0,0289671	-2,643295	0,0173515	0	0	0	0
100194317	LOC100194317	uncharacterized LOC100194317	-4,4161215	0,007306	-5,6267927	0,0014472	-3,337188	0,0220749	0	0	0	0
100194322	LOC100194322	uncharacterized LOC100194322	0	0	0	0	1,6938499	0,0488435	0	0	0	0
100194342	pco083336	uncharacterized LOC100194342	0	0	0	0	0	0	-2,2100177	0,048279	0	0
100216602	LOC100216602	uncharacterized LOC100216602	0	0	0	0	1,567423	0,0267884	0	0	0	0

			Yuc x Ctr		Azo x Ctr		AzoYuc x Ctr		AzoYuc x Yuc		AzoYuc x Azo	
Gene ID	Gene Symbol	Gene Description	Log2(FC)	pvalue	Log2(FC)	pvalue	Log2(FC)	pvalue	Log2(FC)	pvalue	Log2(FC)	pvalue
100216613	LOC100216613	uncharacterized LOC100216613	3,45350896	0,023267	0	0	0	0	0	0	0	0
100216691	cl5021_1	uncharacterized LOC100216691	0	0	0	0	1,7776274	0,0017211	0	0	0	0
100216693	pco137385	uncharacterized LOC100216693	0	0	0	0	0	0	-1,5129455	0,03638	0	0
100216724	cl426_1a	uncharacterized LOC100216724	0	0	0	0	-1,999361	0,0370913	-2,0179593	0,032567	0	0
100216752	cl56262_1	uncharacterized LOC100216752	2,69903241	0,014882	0	0	0	0	0	0	0	0
100216762	LOC100216762	uncharacterized LOC100216762	0	0	0	0	-1,770314	0,0460811	0	0	0	0
100216854	LOC100216854	uncharacterized LOC100216854	-4,1593705	0,01751	0	0	0	0	0	0	0	0
100216879	LOC100216879	uncharacterized LOC100216879	0	0	0	0	0	0	3,16158746	0,018839	0	0
100216964	LOC100216964	uncharacterized LOC100216964	0	0	0	0	3,3140136	0,0084049	2,43028188	0,031364	2,1685821	0,0493638
100216966	LOC100216966	uncharacterized LOC100216966	0	0	0	0	-1,885132	0,0318737	0	0	0	0
100217030	pco131458	uncharacterized LOC100217030	-2,1296474	0,048565	-2,5333119	0,0206665	0	0	0	0	0	0
100217046	pco130711	uncharacterized LOC100217046	-2,2121452	0,022274	0	0	0	0	2,51423106	0,008741	1,8239377	0,049706
100217059	LOC100217059	uncharacterized LOC100217059	0	0	0	0	-1,732893	0,026267	0	0	0	0
100217087	LOC100217087	uncharacterized LOC100217087	0	0	0	0	4,4342441	0,0033556	2,95399834	0,018956	2,7403196	0,0262685
100217101	LOC100217101	uncharacterized LOC100217101	0	0	-2,1666674	0,0286744	0	0	0	0	0	0
100217106	LOC100217106	uncharacterized LOC100217106	0	0	0	0	2,3739915	0,037315	2,77725773	0,014755	0	0
100217114	TIDP2761	uncharacterized LOC100217114	0	0	0	0	-1,517622	0,0373785	0	0	0	0
100217130	LOC100217130	uncharacterized LOC100217130	0	0	0	0	0	0	-2,2963953	0,009479	-2,823149	0,0011651
100217178	LOC100217178	uncharacterized LOC100217178	-2,7567802	0,021217	-2,4345622	0,0364103	-3,680138	0,003426	0	0	0	0
100217254	LOC100217254	uncharacterized LOC100217254	0	0	0	0	1,6100673	0,0463117	0	0	0	0
100217290	LOC100217290	uncharacterized LOC100217290	0	0	0	0	0	0	0	0	4,3539808	0,024081
100217299	LOC100217299	uncharacterized LOC100217299	0	0	2,88487702	0,0191759	0	0	0	0	-2,960909	0,0138803
100217309	LOC100217309	uncharacterized LOC100217309	4,06483679	0,007512	3,56460338	0,0200294	3,8358883	0,0115946	0	0	0	0
100272290	LOC100272290	uncharacterized LOC100272290	0	0	2,97689155	0,0202821	2,7895219	0,0300427	0	0	0	0
100272298	LOC100272298	uncharacterized LOC100272298	0	0	0	0	3,4856066	0,0143116	6,10342495	0,000414	3,0540284	0,0231182
100272343	uaz277(201)	uncharacterized LOC100272343	0	0	0	0	2,7392193	0,0456073	0	0	0	0
100272353	gpm443	uncharacterized LOC100272353	0	0	0	0	4,6070165	0,0006171	4,14533116	0,000698	3,1577008	0,0053284
100272427	LOC100272427	uncharacterized LOC100272427	0	0	0	0	-1,575789	0,0199453	0	0	0	0
100272433	LOC100272433	uncharacterized LOC100272433	0	0	0	0	0	0	0	0	4,1988521	0,024413
100272453	pco133450	uncharacterized LOC100272453	4,13294714	0,028448	0	0	0	0	0	0	0	0
100272543	LOC100272543	uncharacterized LOC100272543	0	0	-3,7168256	0,0435575	-3,747173	0,0418707	0	0	0	0
100272571	LOC100272571	uncharacterized LOC100272571	-3,5027714	0,005922	-3,1858395	0,0094885	0	0	0	0	0	0
100272634	LOC100272634	uncharacterized LOC100272634	0	0	0	0	0	0	0	0	3,2981719	0,0344641
100272671	LOC100272671	uncharacterized LOC100272671	0	0	0	0	1,5718433	0,0358124	0	0	0	0
100272733	LOC100272733	uncharacterized LOC100272733	0	0	0	0	0	0	0	0	3,8877232	0,0317936
100272755	CL11475_1	uncharacterized LOC100272755	0	0	0	0	2,0733205	0,018778	1,80189542	0,034041	0	0
100273040	LOC100273040	uncharacterized LOC100273040	0	0	0	0	3,3854612	0,0486888	0	0	0	0
100273080	LOC100273080	uncharacterized LOC100273080	-2,5280016	0,013665	-2,8134065	0,0062331	-2,169488	0,0311228	0	0	0	0
100273158	LOC100273158	uncharacterized LOC100273158	-1,809192	0,021069	-1,585049	0,0417591	-1,975159	0,0116439	0	0	0	0
100273339	LOC100273339	uncharacterized LOC100273339	0	0	1,59908603	0,0343986	0	0	0	0	-1,552559	0,0357516
100273371	LOC100273371	uncharacterized LOC100273371	0	0	2,92561421	0,021609	0	0	0	0	-3,833346	0,0034186
100273476	pco087970b	uncharacterized LOC100273476	1,60205166	0,022392	0	0	0	0	0	0	0	0

			Yuc x Ctr		Azo x Ctr		AzoYuc x Ctr		AzoYuc x Yuc		AzoYuc x Azo	
Gene ID	Gene Symbol	Gene Description	Log2(FC)	pvalue	Log2(FC)	pvalue	Log2(FC)	pvalue	Log2(FC)	pvalue	Log2(FC)	pvalue
100273577	TIDP3460	uncharacterized LOC100273577	0	0	3,07808403	0,0489464	0	0	0	0	0	0
100273700	pco073762	uncharacterized LOC100273700	0	0	0	0	0	0	0	0	3,4442462	0,0224666
100273721	LOC100273721	uncharacterized LOC100273721	-2,0566228	0,034778	0	0	0	0	0	0	0	0
100273729	cl7341_1a	uncharacterized LOC100273729	0	0	0	0	-2,084194	0,0227979	-2,2729962	0,011425	0	0
100273730	LOC100273730	uncharacterized LOC100273730	-2,2098681	0,007489	-2,4396122	0,0031813	-2,825396	0,0006938	0	0	0	0
100273734	LOC100273734	uncharacterized LOC100273734	0	0	0	0	0	0	1,90811325	0,040658	0	0
100273811	LOC100273811	uncharacterized LOC100273811	0	0	0	0	0	0	2,49036384	0,046899	2,4909702	0,0453918
100273889	LOC100273889	uncharacterized LOC100273889	0	0	0	0	2,1754539	0,0053255	1,50618098	0,044469	1,8989959	0,0117559
100273916	LOC100273916	uncharacterized LOC100273916	0	0	0	0	3,2065506	0,025842	0	0	0	0
100274052	pco079415(520)	uncharacterized LOC100274052	0	0	0	0	0	0	0	0	3,8752543	0,0308186
100274066	LOC100274066	uncharacterized LOC100274066	-2,5286973	0,024807	-3,2991934	0,0052146	-4,100375	0,0015312	0	0	0	0
100274126	LOC100274126	uncharacterized LOC100274126	0	0	0	0	0	0	1,5970536	0,028506	0	0
100274143	LOC100274143	uncharacterized LOC100274143	0	0	0	0	-2,840881	0,0338757	-3,1171027	0,017025	-2,906416	0,0260864
100274175	LOC100274175	uncharacterized LOC100274175	0	0	0	0	3,3120495	0,0350494	3,19421271	0,030231	0	0
100274247	LOC100274247	uncharacterized LOC100274247	0	0	0	0	0	0	0	0	4,5165776	0,0113412
100274327	pco077290a	uncharacterized LOC100274327	0	0	0	0	0	0	2,60015225	0,036388	0	0
100274346	LOC100274346	uncharacterized LOC100274346	0	0	0	0	-1,583654	0,0323943	0	0	0	0
100274388	pco128421	uncharacterized LOC100274388	1,51307248	0,010004	0	0	0	0	0	0	0	0
100274417	LOC100274417	uncharacterized LOC100274417	0	0	0	0	3,7606412	0,020953	0	0	0	0
100274428	LOC100274428	uncharacterized LOC100274428	0	0	0	0	1,5971232	0,0310012	0	0	0	0
100274481	LOC100274481	uncharacterized LOC100274481	0	0	0	0	1,8548853	0,0387466	2,70415144	0,003299	0	0
100274493	cl4945_1	uncharacterized LOC100274493	0	0	1,97992757	0,0288025	0	0	0	0	0	0
100274547	LOC100274547	uncharacterized LOC100274547	0	0	0	0	0	0	0	0	3,213486	0,0327359
100274555	LOC100274555	uncharacterized LOC100274555	0	0	-3,869411	0,0334095	0	0	0	0	3,7798918	0,0339059
100274655	LOC100274655	uncharacterized LOC100274655	0	0	0	0	0	0	0	0	4,7697462	0,0350329
100274722	LOC100274722	uncharacterized LOC100274722	0	0	-1,7530351	0,045662	0	0	0	0	0	0
100274819	LOC100274819	uncharacterized LOC100274819	0	0	0	0	2,248102	0,039223	4,55055685	0,000542	2,7752012	0,0104919
100274850	IDP2453	uncharacterized LOC100274850	0	0	0	0	-3,003941	0,0003119	-3,2636375	8,66E-05	-2,664582	0,0013707
100274866	LOC100274866	uncharacterized LOC100274866	1,65231536	0,011085	0	0	1,6629872	0,0099862	0	0	0	0
100274896	LOC100274896	uncharacterized LOC100274896	0	0	0	0	0	0	1,95921258	0,012896	1,7405333	0,0254824
100274912	LOC100274912	uncharacterized LOC100274912	0	0	0	0	0	0	5,06457318	0,017726	4,3287219	0,0426871
100274944	LOC100274944	uncharacterized LOC100274944	0	0	0	0	4,2950513	0,0253753	4,71778647	0,014062	0	0
100274990	LOC100274990	uncharacterized LOC100274990	0	0	0	0	-1,518065	0,0096253	0	0	0	0
100274993	LOC100274993	uncharacterized LOC100274993	-4,047178	0,028792	0	0	0	0	0	0	0	0
100275001	LOC100275001	uncharacterized LOC100275001	0	0	0	0	0	0	5,52643413	0,045663	0	0
100275057	LOC100275057	uncharacterized LOC100275057	-2,9114086	0,002326	-2,5124731	0,0054034	-2,083748	0,017169	0	0	0	0
100275125	LOC100275125	uncharacterized LOC100275125	0	0	0	0	1,8136456	0,0489728	0	0	0	0
100275129	LOC100275129	uncharacterized LOC100275129	0	0	-3,5111091	0,0082355	-2,854175	0,0253724	0	0	0	0
100275204	TIDP3241	uncharacterized LOC100275204	-1,6114537	0,037318	-1,7971081	0,0196442	0	0	0	0	0	0
100275297	LOC100275297	uncharacterized LOC100275297	-1,6683333	0,029106	0	0	0	0	0	0	0	0
100275309	pco110563	uncharacterized LOC100275309	0	0	0	0	0	0	0	0	-5,058115	0,0033463
100275318	LOC100275318	uncharacterized LOC100275318	0	0	2,30459137	0,0270943	0	0	0	0	0	0

			Yuc x Ctr		Azo x Ctr		AzoYuc x Ctr		AzoYuc x Yuc		AzoYuc x Azo	
Gene ID	Gene Symbol	Gene Description	Log2(FC)	pvalue	Log2(FC)	pvalue	Log2(FC)	pvalue	Log2(FC)	pvalue	Log2(FC)	pvalue
100275374	LOC100275374	uncharacterized LOC100275374	0	0	0	0	4,2752647	0,0352322	4,69799942	0,020674	0	0
100275449	LOC100275449	uncharacterized LOC100275449	-3,5412097	4,33E-05	-2,9665255	0,0005234	-3,169571	0,000216	0	0	0	0
100275463	cl5155_1	uncharacterized LOC100275463	0	0	0	0	2,6059817	0,0013538	2,02104363	0,007557	1,9427769	0,0092246
100275495	LOC100275495	uncharacterized LOC100275495	-5,5765119	0,0013	-4,8406502	0,005244	0	0	3,94033886	0,025429	0	0
100275503	LOC100275503	uncharacterized LOC100275503	0	0	0	0	2,6447994	0,0275183	0	0	0	0
100275527	LOC100275527	uncharacterized LOC100275527	3,45375998	0,004183	0	0	0	0	-4,992062	0,000112	0	0
100275576	LOC100275576	uncharacterized LOC100275576	0	0	0	0	4,0209283	0,0266385	0	0	0	0
100275588	LOC100275588	uncharacterized LOC100275588	0	0	2,95619214	0,0026336	0	0	0	0	-3,579267	0,0002652
100275671	LOC100275671	uncharacterized LOC100275671	0	0	-3,5848232	0,0121773	0	0	0	0	0	0
100275705	LOC100275705	uncharacterized LOC100275705	0	0	0	0	0	0	3,83943523	0,003122	3,2522487	0,0077949
100275748	LOC100275748	uncharacterized LOC100275748	0	0	0	0	2,8273106	0,0479568	0	0	0	0
100275772	LOC100275772	uncharacterized LOC100275772	0	0	0	0	0	0	-4,0541033	0,049517	0	0
100275797	LOC100275797	uncharacterized LOC100275797	-5,1922343	0,021392	0	0	0	0	0	0	0	0
100275804	LOC100275804	uncharacterized LOC100275804	-2,3340838	0,013385	0	0	0	0	0	0	0	0
100275868	LOC100275868	uncharacterized LOC100275868	0	0	2,3579079	0,0038723	0	0	0	0	-2,368108	0,0017209
100275894	LOC100275894	uncharacterized LOC100275894	-3,6607237	0,006376	0	0	-3,817477	0,0042647	0	0	0	0
100275960	IDP2353	uncharacterized LOC100275960	0	0	-2,6944638	0,0275697	0	0	0	0	0	0
100276011	cl16961_1(9)	uncharacterized LOC100276011	0	0	-1,965994	0,0192402	0	0	0	0	0	0
100276016	LOC100276016	uncharacterized LOC100276016	0	0	0	0	4,029539	0,0212692	0	0	0	0
100276169	LOC100276169	uncharacterized LOC100276169	0	0	0	0	0	0	1,95299526	0,031435	2,005661	0,0265227
100276236	umc2366	uncharacterized LOC100276236	0	0	0	0	0	0	-2,3505884	0,037268	0	0
100276246	pco101498	uncharacterized LOC100276246	0	0	0	0	-1,654851	0,0361565	0	0	0	0
100276267	IDP1437	uncharacterized LOC100276267	0	0	0	0	0	0	0	0	-2,710649	0,0319898
100276273	IDP1474	uncharacterized LOC100276273	0	0	0	0	2,7446123	0,0105006	0	0	0	0
100276291	cl5598_1(581)	uncharacterized LOC100276291	0	0	0	0	3,3679023	0,012615	0	0	3,8126702	0,004189
100276318	LOC100276318	uncharacterized LOC100276318	0	0	0	0	3,9622078	0,0071527	4,32169463	0,003235	5,0302543	0,0009432
100276376	TIDP3268	uncharacterized LOC100276376	3,21493747	0,035089	0	0	0	0	0	0	0	0
100276388	LOC100276388	uncharacterized LOC100276388	3,4795556	0,032155	3,35310308	0,0384342	5,1902758	0,0009117	0	0	0	0
100276395	LOC100276395	uncharacterized LOC100276395	0	0	0	0	1,785715	0,0465374	0	0	0	0
100276401	LOC100276401	uncharacterized LOC100276401	0	0	0	0	4,7082834	0,0054947	2,93977912	0,038952	3,0396709	0,0321073
100276431	LOC100276431	uncharacterized LOC100276431	0	0	-1,5242871	0,0162332	0	0	0	0	0	0
100276434	LOC100276434	uncharacterized LOC100276434	0	0	0	0	0	0	1,59954711	0,026477	0	0
100276521	pco076094	uncharacterized LOC100276521	0	0	3,97449095	0,0119883	4,340231	0,0056832	0	0	0	0
100276526	LOC100276526	uncharacterized LOC100276526	0	0	-1,7676775	0,0145255	0	0	0	0	0	0
100276553	LOC100276553	uncharacterized LOC100276553	-1,6978776	0,013079	0	0	-1,517045	0,0249514	0	0	0	0
100276570	LOC100276570	uncharacterized LOC100276570	0	0	0	0	0	0	0	0	3,7585163	0,0076218
100276581	LOC100276581	uncharacterized LOC100276581	-1,756316	0,032483	0	0	-1,800292	0,0263795	0	0	0	0
100276587	LOC100276587	uncharacterized LOC100276587	0	0	0	0	2,7317398	0,0016421	2,39358838	0,00446	2,9311499	0,0005806
100276802	pco097773	uncharacterized LOC100276802	0	0	0	0	0	0	0	0	-2,3338	0,0287507
100276845	LOC100276845	uncharacterized LOC100276845	0	0	0	0	-1,883314	0,0382867	0	0	0	0
100276855	umc1238	uncharacterized LOC100276855	0	0	0	0	0	0	0	0	-2,165457	0,0368992
100276899	LOC100276899	uncharacterized LOC100276899	0	0	0	0	0	0	0	0	3,0860253	0,0471465

			Yuc x Ctr		Azo x Ctr		AzoYuc x Ctr		AzoYuc x Yuc		AzoYuc x Azo	
Gene ID	Gene Symbol	Gene Description	Log2(FC)	pvalue	Log2(FC)	pvalue	Log2(FC)	pvalue	Log2(FC)	pvalue	Log2(FC)	pvalue
100276922	LOC100276922	uncharacterized LOC100276922	-3,8721789	0,029871	0	0	0	0	0	0	0	0
100276955	LOC100276955	uncharacterized LOC100276955	0	0	1,80041134	0,0263155	0	0	0	0	0	0
100276958	LOC100276958	uncharacterized LOC100276958	0	0	0	0	0	0	2,25453762	0,043002	0	0
100276977	LOC100276977	uncharacterized LOC100276977	0	0	0	0	2,1846288	0,0466335	2,64391568	0,016389	2,4069027	0,0240792
100276989	LOC100276989	uncharacterized LOC100276989	2,64933311	0,049485	0	0	0	0	0	0	0	0
100277035	pco133003	uncharacterized LOC100277035	0	0	0	0	1,951998	0,0188863	2,33424675	0,004941	1,9602258	0,0162268
100277055	LOC100277055	uncharacterized LOC100277055	2,04194019	0,01706	0	0	0	0	-2,531351	0,003008	0	0
100277126	LOC100277126	uncharacterized LOC100277126	-2,3388491	0,035949	0	0	0	0	0	0	0	0
100277172	LOC100277172	uncharacterized LOC100277172	-2,6594035	0,00905	-2,6865663	0,007447	-2,018414	0,0356956	0	0	0	0
100277363	LOC100277363	uncharacterized LOC100277363	0	0	-3,8058449	0,0202488	0	0	0	0	0	0
100277380	LOC100277380	uncharacterized LOC100277380	0	0	0	0	3,7200508	0,0269666	0	0	0	0
100277402	cl7681_1a	uncharacterized LOC100277402	0	0	0	0	0	0	0	0	-2,913372	0,0477735
100277500	umc2034	uncharacterized LOC100277500	0	0	0	0	0	0	2,73611096	0,038908	0	0
100277507	LOC100277507	uncharacterized LOC100277507	0	0	0	0	2,89239	0,0033526	2,84226412	0,002816	1,8863896	0,0359787
100277551	LOC100277551	uncharacterized LOC100277551	4,40179006	0,003526	0	0	3,3647044	0,0273614	0	0	0	0
100277562	pco136884	uncharacterized LOC100277562	0	0	2,09916304	0,0008141	0	0	0	0	-1,504086	0,0085126
100277564	LOC100277564	uncharacterized LOC100277564	0	0	0	0	-2,231031	0,0371957	0	0	0	0
100277588	umc2512	uncharacterized LOC100277588	-2,2595235	0,012454	0	0	0	0	0	0	0	0
100277664	LOC100277664	uncharacterized LOC100277664	-3,3863018	0,001027	-3,9950607	0,0001373	-3,224031	0,001554	0	0	0	0
100277683	LOC100277683	uncharacterized LOC100277683	0	0	0	0	2,7710684	0,0110014	2,75784885	0,009837	2,2673668	0,029915
100277689	LOC100277689	uncharacterized LOC100277689	0	0	0	0	0	0	-2,068221	0,044883	-2,239543	0,0276695
100277718	LOC100277718	uncharacterized LOC100277718	0	0	0	0	0	0	4,09771219	0,005962	0	0
100277730	LOC100277730	uncharacterized LOC100277730	0	0	0	0	0	0	1,82955617	0,029518	0	0
100277755	LOC100277755	uncharacterized LOC100277755	0	0	0	0	3,0292386	0,0450989	0	0	0	0
100277777	LOC100277777	uncharacterized LOC100277777	0	0	0	0	3,2521263	0,0457245	0	0	0	0
100277794	LOC100277794	uncharacterized LOC100277794	-4,0836844	0,049002	0	0	0	0	0	0	0	0
100277797	LOC100277797	uncharacterized LOC100277797	-5,1312441	0,009501	-4,1473122	0,0290734	-5,357105	0,0067038	0	0	0	0
100277822	LOC100277822	uncharacterized LOC100277822	0	0	0	0	-2,181975	0,0339964	0	0	0	0
100277858	LOC100277858	uncharacterized LOC100277858	0	0	0	0	3,0562797	0,0112937	0	0	2,9370717	0,0081667
100277880	LOC100277880	uncharacterized LOC100277880	0	0	0	0	0	0	2,72727136	0,010076	0	0
100277944	LOC100277944	uncharacterized LOC100277944	0	0	0	0	-1,532328	0,0439446	0	0	0	0
100277950	cl6482_1b	uncharacterized LOC100277950	2,24877614	0,02959	0	0	0	0	0	0	0	0
100278035	LOC100278035	uncharacterized LOC100278035	0	0	3,86342783	0,0362807	0	0	0	0	0	0
100278056	LOC100278056	uncharacterized LOC100278056	0	0	0	0	2,3439208	0,0153206	2,62206941	0,006174	2,5590745	0,0065891
100278071	LOC100278071	uncharacterized LOC100278071	0	0	-3,114495	0,0376065	0	0	0	0	0	0
100278125	LOC100278125	uncharacterized LOC100278125	0	0	-1,6934266	0,020332	0	0	0	0	0	0
100278194	LOC100278194	uncharacterized LOC100278194	0	0	0	0	0	0	0	0	3,2560193	0,0190461
100278201	LOC100278201	uncharacterized LOC100278201	0	0	2,81145941	0,0234273	0	0	0	0	0	0
100278255	IDP353	uncharacterized LOC100278255	0	0	0	0	0	0	0	0	1,833375	0,0171706
100278279	LOC100278279	uncharacterized LOC100278279	0	0	0	0	0	0	0	0	2,0782328	0,0385154
100278302	LOC100278302	uncharacterized LOC100278302	0	0	0	0	2,7247532	0,0277783	0	0	0	0
100278316	si946092e03	uncharacterized LOC100278316	1,55774951	0,020889	0	0	0	0	-1,9475724	0,003243	0	0

			Yuc x Ctr		Azo x Ctr		AzoYuc x Ctr		AzoYuc x Yuc		AzoYuc x Azo	
Gene ID	Gene Symbol	Gene Description	Log2(FC)	pvalue	Log2(FC)	pvalue	Log2(FC)	pvalue	Log2(FC)	pvalue	Log2(FC)	pvalue
100278318	IDP667	uncharacterized LOC100278318	0	0	0	0	-2,661527	0,0198842	0	0	0	0
100278326	LOC100278326	uncharacterized LOC100278326	3,49423461	0,021679	3,1015046	0,0423532	0	0	0	0	0	0
100278328	LOC100278328	uncharacterized LOC100278328	0	0	0	0	0	0	2,44631187	0,019185	2,4704948	0,0165811
100278395	LOC100278395	uncharacterized LOC100278395	0	0	0	0	-1,811163	0,0207085	0	0	0	0
100278460	LOC100278460	uncharacterized LOC100278460	0	0	0	0	0	0	2,60284897	0,008781	2,7204177	0,0060962
100278461	LOC100278461	uncharacterized LOC100278461	0	0	0	0	0	0	1,52192503	0,000536	0	0
100278465	LOC100278465	uncharacterized LOC100278465	-2,7699553	0,011682	0	0	0	0	0	0	0	0
100278522	LOC100278522	uncharacterized LOC100278522	0	0	-2,6730468	0,0100893	0	0	0	0	0	0
100278542	LOC100278542	uncharacterized LOC100278542	0	0	0	0	0	0	-2,4752054	0,033705	0	0
100278555	LOC100278555	uncharacterized LOC100278555	0	0	2,53946361	0,0363899	0	0	0	0	0	0
100278616	LOC100278616	uncharacterized LOC100278616	0	0	0	0	2,084328	0,0490491	0	0	0	0
100278628	LOC100278628	uncharacterized LOC100278628	0	0	0	0	0	0	2,05278175	0,033946	0	0
100278657	LOC100278657	uncharacterized LOC100278657	0	0	-3,2278785	0,0294388	0	0	0	0	0	0
100278713	LOC100278713	uncharacterized LOC100278713	0	0	0	0	3,8258994	0,0029808	4,57895741	0,000505	4,0359623	0,0013768
100278787	LOC100278787	uncharacterized LOC100278787	0	0	0	0	0	0	-1,9120366	0,022688	0	0
100278838	LOC100278838	uncharacterized LOC100278838	-3,9358592	0,012361	0	0	0	0	3,47150991	0,026655	0	0
100278859	LOC100278859	uncharacterized LOC100278859	0	0	0	0	2,2068463	0,0335784	1,96209674	0,041334	0	0
100278862	LOC100278862	uncharacterized LOC100278862	0	0	2,14499912	0,0361776	0	0	0	0	0	0
100278869	LOC100278869	uncharacterized LOC100278869	0	0	0	0	-2,234053	0,0289626	0	0	0	0
100278901	LOC100278901	uncharacterized LOC100278901	0	0	0	0	0	0	2,20742205	0,033461	2,3732328	0,0219217
100279000	LOC100279000	uncharacterized LOC100279000	1,62986932	0,046292	0	0	0	0	-2,1836241	0,007371	0	0
100279003	LOC100279003	uncharacterized LOC100279003	0	0	0	0	1,8004412	0,0435111	2,23850115	0,012208	0	0
100279006	LOC100279006	uncharacterized LOC100279006	0	0	0	0	2,1506081	0,0360393	0	0	0	0
100279155	LOC100279155	uncharacterized LOC100279155	0	0	0	0	0	0	1,51558711	0,026406	0	0
100279197	LOC100279197	uncharacterized LOC100279197	0	0	-3,0520881	0,0261009	0	0	0	0	0	0
100279214	LOC100279214	uncharacterized LOC100279214	-4,8239552	0,021584	0	0	0	0	0	0	0	0
100279239	cl21263_1	uncharacterized LOC100279239	0	0	0	0	2,1831084	0,0023974	2,38930797	0,00078	2,0747863	0,0028629
100279249	LOC100279249	uncharacterized LOC100279249	0	0	0	0	2,1356459	0,0007263	0	0	0	0
100279259	LOC100279259	uncharacterized LOC100279259	0	0	0	0	2,4698588	0,0475123	0	0	0	0
100279299	LOC100279299	uncharacterized LOC100279299	0	0	0	0	1,888341	0,0347796	0	0	0	0
100279324	LOC100279324	uncharacterized LOC100279324	0	0	0	0	4,4675361	0,0039557	0	0	0	0
100279364	LOC100279364	uncharacterized LOC100279364	-3,9815156	0,00471	-4,8686166	0,0006457	-3,91367	0,0052759	0	0	0	0
100279366	pco123556	uncharacterized LOC100279366	0	0	-2,299451	0,0007165	-2,376021	0,0004752	0	0	0	0
100279369	LOC100279369	uncharacterized LOC100279369	0	0	0	0	2,3365608	0,0178745	0	0	0	0
100279390	LOC100279390	uncharacterized LOC100279390	3,10345212	0,013793	3,28620102	0,0086584	3,1164763	0,0129548	0	0	0	0
100279423	umc1322	uncharacterized LOC100279423	0	0	0	0	4,1300887	0,0334648	0	0	0	0
100279455	pco076369b	uncharacterized LOC100279455	0	0	0	0	1,7554151	0,0295212	0	0	0	0
100279471	LOC100279471	uncharacterized LOC100279471	0	0	0	0	-3,209198	0,0285307	0	0	0	0
100279525	LOC100279525	uncharacterized LOC100279525	0	0	0	0	2,1766223	0,021597	3,19587874	0,00105	2,5031051	0,0076052
100279655	IDP1984	uncharacterized LOC100279655	0	0	0	0	1,9633076	0,0007095	0	0	0	0
100279714	umc2615	uncharacterized LOC100279714	1,8850073	0,021457	0	0	2,4225988	0,0025757	0	0	0	0
100279717	cl715_-2a	uncharacterized LOC100279717	0	0	0	0	2,0666716	0,0122124	1,76924365	0,028044	2,0585931	0,010804

			Yuc x Ctr		Azo x Ctr		AzoYuc x Ctr		AzoYuc x Yuc		AzoYuc x Azo	
Gene ID	Gene Symbol	Gene Description	Log2(FC)	pvalue	Log2(FC)	pvalue	Log2(FC)	pvalue	Log2(FC)	pvalue	Log2(FC)	pvalue
100279753	LOC100279753	uncharacterized LOC100279753	0	0	0	0	0	0	3,18200114	0,00221	3,7430098	0,0004139
100279761	LOC100279761	uncharacterized LOC100279761	-3,135828	0,009944	-4,031075	0,0021866	-3,156628	0,0079477	0	0	0	0
100279862	LOC100279862	uncharacterized LOC100279862	0	0	0	0	2,4891405	0,0144107	0	0	0	0
100279863	LOC100279863	uncharacterized LOC100279863	0	0	3,27438291	0,0114141	0	0	0	0	-3,265468	0,0084898
100279897	LOC100279897	uncharacterized LOC100279897	0	0	0	0	0	0	2,22646938	0,004341	0	0
100279908	pco101952	uncharacterized LOC100279908	0	0	0	0	2,247108	0,0003052	2,25427708	0,000245	2,4546598	6,546E-05
100279915	LOC100279915	uncharacterized LOC100279915	-1,8521075	0,000872	0	0	0	0	0	0	0	0
100279917	LOC100279917	uncharacterized LOC100279917	0	0	0	0	0	0	-1,6531328	0,013398	0	0
100279942	cl40794_1	uncharacterized LOC100279942	0	0	0	0	0	0	2,33311455	0,022231	2,6236561	0,0104873
100279985	LOC100279985	uncharacterized LOC100279985	-1,569972	0,022403	0	0	0	0	0	0	0	0
100279991	LOC100279991	uncharacterized LOC100279991	0	0	0	0	0	0	0	0	-4,220807	0,0490968
100280005	LOC100280005	uncharacterized LOC100280005	0	0	0	0	2,4721817	0,0425533	0	0	0	0
100280049	LOC100280049	uncharacterized LOC100280049	0	0	0	0	4,0843365	0,0172402	0	0	0	0
100280100	LOC100280100	uncharacterized LOC100280100	0	0	0	0	2,5402771	0,0022282	2,65907807	0,001109	1,6911426	0,0295066
100280106	LOC100280106	uncharacterized LOC100280106	0	0	0	0	3,0968129	0,0143845	2,87156804	0,016799	2,8842012	0,015017
100280146	LOC100280146	uncharacterized LOC100280146	0	0	0	0	-3,774919	0,0278142	-4,5454115	0,00725	0	0
100280165	LOC100280165	uncharacterized LOC100280165	0	0	0	0	3,7629828	0,0118597	0	0	0	0
100280286	LOC100280286	uncharacterized LOC100280286	-4,4489256	9,46E-06	-4,1192366	3,014E-05	-3,471765	0,000352	0	0	0	0
100280323	LOC100280323	uncharacterized LOC100280323	0	0	0	0	1,843845	0,0071376	2,04726756	0,002728	1,9118791	0,004949
100280354	LOC100280354	uncharacterized LOC100280354	-1,72767	0,028315	-2,0109169	0,0110783	0	0	0	0	0	0
100280368	LOC100280368	uncharacterized LOC100280368	0	0	0	0	0	0	3,42051479	0,007446	3,4995748	0,0056476
100280415	LOC100280415	uncharacterized LOC100280415	0	0	0	0	2,2527055	0,0219906	2,42623253	0,012271	0	0
100280438	LOC100280438	uncharacterized LOC100280438	1,52311554	0,032855	0	0	0	0	0	0	0	0
100280497	gpm667	uncharacterized LOC100280497	0	0	0	0	0	0	2,45813277	0,015894	2,4820175	0,0139604
100280586	si605047e03	uncharacterized LOC100280586	-2,1835159	0,003717	-2,4904653	0,0009953	-2,062606	0,0053328	0	0	0	0
100280589	pco099218(710)	uncharacterized LOC100280589	-1,636848	0,035694	0	0	-1,51795	0,0496977	0	0	0	0
100280710	TIDP3391	uncharacterized LOC100280710	0	0	0	0	-2,338954	0,0021186	0	0	0	0
100280770	pco066751	uncharacterized LOC100280770	1,78568213	0,033248	0	0	0	0	0	0	0	0
100280824	uaz235(px)	uncharacterized LOC100280824	0	0	3,22696401	0,0065556	0	0	0	0	-4,861949	0,0001558
100280958	IDP641	uncharacterized LOC100280958	0	0	0	0	-1,601312	0,0354112	0	0	0	0
100281002	pco108588	uncharacterized LOC100281002	0	0	-1,8792379	0,0233375	0	0	0	0	0	0
100281016	si603006c09	uncharacterized LOC100281016	0	0	2,62244524	0,0135472	0	0	0	0	-2,123374	0,0201072
100281034	pco102615	uncharacterized LOC100281034	0	0	-1,5501037	0,024042	0	0	0	0	0	0
100281131	gpm680	uncharacterized LOC100281131	-2,195424	0,045234	0	0	0	0	0	0	0	0
100281166	si687013d08	uncharacterized LOC100281166	2,41548105	0,000651	0	0	0	0	0	0	0	0
100281219	pco141803	uncharacterized LOC100281219	0	0	0	0	0	0	1,7496417	0,0117	0	0
100281298	si687036a06	uncharacterized LOC100281298	4,05614602	0,003189	0	0	0	0	-2,4332428	0,044378	0	0
100281394	si707002b03b	uncharacterized LOC100281394	0	0	0	0	3,3198498	0,0084589	2,92690125	0,013656	0	0
100281422	cl29544_1a	uncharacterized LOC100281422	0	0	0	0	2,07988	0,0240412	0	0	2,0291687	0,0260773
100281425	LOC100281425	uncharacterized LOC100281425	2,91218518	0,011941	0	0	0	0	-2,8843189	0,010051	0	0
100281458	pco141348	uncharacterized LOC100281458	0	0	0	0	0	0	0	0	-2,222642	0,0263994
100281468	magi100654	uncharacterized LOC100281468	0	0	1,53146574	0,0400462	0	0	0	0	0	0

			Yuc x Ctr		Azo x Ctr		AzoYuc x Ctr		AzoYuc x Yuc		AzoYuc x Azo	
Gene ID	Gene Symbol	Gene Description	Log2(FC)	pvalue	Log2(FC)	pvalue	Log2(FC)	pvalue	Log2(FC)	pvalue	Log2(FC)	pvalue
100281503	umc1155	uncharacterized LOC100281503	0	0	0	0	1,8999004	0,0038694	0	0	0	0
100281507	pco138672	uncharacterized LOC100281507	2,64999218	0,013922	0	0	0	0	0	0	0	0
100281537	si605069e01	uncharacterized LOC100281537	0	0	0	0	2,4243538	0,0247051	2,35806024	0,024493	2,2535698	0,0291634
100281767	pco104637	uncharacterized LOC100281767	0	0	0	0	0	0	0	0	-1,968679	0,0200568
100281771	cl18044_1	uncharacterized LOC100281771	-2,6021897	0,024377	0	0	0	0	0	0	0	0
100281965	LOC100281965	uncharacterized LOC100281965	3,81232465	0,000904	0	0	0	0	-4,0551332	0,000277	0	0
100282018	umc2196	uncharacterized LOC100282018	0	0	0	0	0	0	-2,1986759	0,01547	0	0
100282310	umc1314	uncharacterized LOC100282310	0	0	-1,5096672	0,020051	0	0	0	0	0	0
100282455	pco106809	uncharacterized LOC100282455	0	0	-2,1980955	0,0026075	-1,86621	0,0091112	0	0	0	0
100282684	pco120183	uncharacterized LOC100282684	0	0	0	0	1,822801	0,0096881	0	0	0	0
100282946	gpm447	uncharacterized LOC100282946	0	0	0	0	-1,706458	0,0035557	0	0	0	0
100283086	pco084622	uncharacterized LOC100283086	0	0	0	0	2,1339196	0,0267124	0	0	0	0
100283169	umc2388	uncharacterized LOC100283169	0	0	0	0	1,9058368	0,0002586	0	0	0	0
100283198	pco133953b	uncharacterized LOC100283198	0	0	-1,5975185	0,0152263	0	0	0	0	0	0
100283272	pco138129	uncharacterized LOC100283272	0	0	0	0	0	0	1,74845151	0,008961	0	0
100283315	pco087009	uncharacterized LOC100283315	0	0	-1,9495901	0,0342363	-2,697026	0,005543	0	0	0	0
100283321	cl421_1	uncharacterized LOC100283321	2,68434888	0,005654	0	0	0	0	-2,7550133	0,003182	0	0
100283417	pco127462b	uncharacterized LOC100283417	1,90634645	0,002087	0	0	0	0	0	0	0	0
100283481	cl15601_2	uncharacterized LOC100283481	0	0	0	0	3,474748	0,0241904	3,8655453	0,011497	0	0
100283761	pco066552	uncharacterized LOC100283761	-1,6609796	0,04283	0	0	-1,62503	0,0444016	0	0	0	0
100283869	LOC100283869	uncharacterized LOC100283869	2,9919231	0,006764	0	0	0	0	-2,2657599	0,030041	0	0
100284077	pco109288	uncharacterized LOC100284077	4,82599207	0,006114	0	0	0	0	-2,8949197	0,042292	0	0
100284102	gpm917	uncharacterized LOC100284102	0	0	0	0	0	0	2,81615685	0,017124	0	0
100284215	pco101548(16)	uncharacterized LOC100284215	0	0	0	0	1,9192384	0,0237636	1,80886422	0,027961	1,8592094	0,0228819
100284334	IDP1611	uncharacterized LOC100284334	0	0	0	0	-1,839063	0,0024386	0	0	0	0
100284351	umc2542	uncharacterized LOC100284351	0	0	0	0	2,079388	0,0003624	0	0	1,8541455	0,0008831
100284363	si707024f02	uncharacterized LOC100284363	0	0	0	0	1,5763669	0,0468219	0	0	0	0
100284589	cl362_2b	uncharacterized LOC100284589	1,6742817	0,035565	0	0	0	0	0	0	0	0
100284700	pco142662	uncharacterized LOC100284700	0	0	-1,8363347	0,010565	0	0	1,618614	0,020617	2,3361661	0,001
100284708	umc2624	uncharacterized LOC100284708	-1,8764007	0,007895	0	0	0	0	0	0	0	0
100284832	si614088f03a	uncharacterized LOC100284832	-1,7325686	0,029551	-1,7349497	0,0282671	-2,37518	0,0033323	0	0	0	0
100284871	pco093706	uncharacterized LOC100284871	1,85979548	0,024184	0	0	0	0	-1,5465132	0,036786	0	0
100284970	cl1774_-2(594)	uncharacterized LOC100284970	-5,0121175	0,005699	-3,3303157	0,0323942	0	0	0	0	0	0
100284981	LOC100284981	uncharacterized LOC100284981	-3,1609134	0,026018	0	0	0	0	4,76853598	0,000625	3,6094963	0,004009
100285006	umc2775	uncharacterized LOC100285006	2,03743855	0,025547	0	0	3,0936893	0,0005384	0	0	1,5524175	0,0386727
100285339	cl4013_1	uncharacterized LOC100285339	0	0	-1,7804886	0,0027791	0	0	0	0	0	0
100285342	umc2759	uncharacterized LOC100285342	0	0	0	0	-2,440153	0,0118335	0	0	0	0
100285558	pco148653	uncharacterized LOC100285558	-1,9485546	0,009374	-1,9339559	0,0089413	-1,881124	0,010558	0	0	0	0
100285784	TIDP2770	uncharacterized LOC100285784	4,79387768	0,001179	0	0	0	0	-3,831118	0,001497	0	0
100285816	LOC100285816	uncharacterized LOC100285816	0	0	0	0	3,0672134	0,0290513	0	0	0	0
100285908	cl7206_1	uncharacterized LOC100285908	-1,8585573	0,021238	0	0	0	0	0	0	0	0
100285911	pco148010	uncharacterized LOC100285911	0	0	0	0	0	0	2,9104412	0,03795	2,7348375	0,0468194

			Yuc x Ctr		Azo x Ctr		AzoYuc x Ctr		AzoYuc x Yuc		AzoYuc x Azo	
Gene ID	Gene Symbol	Gene Description	Log2(FC)	pvalue	Log2(FC)	pvalue	Log2(FC)	pvalue	Log2(FC)	pvalue	Log2(FC)	pvalue
100285948	cl31549_1	uncharacterized LOC100285948	0	0	0	0	0	0	0	0	2,7203512	0,0409745
100286147	si605092h09	uncharacterized LOC100286147	6,04545034	0,004364	0	0	0	0	-5,4110684	0,006048	0	0
100286373	cl27617_1b	uncharacterized LOC100286373	0	0	-2,1367592	0,0047603	-1,628222	0,0236818	0	0	0	0
100303788	LOC100303788	uncharacterized LOC100303788	0	0	-2,2278223	0,0335774	0	0	0	0	0	0
100303858	LOC100303858	uncharacterized LOC100303858	3,26191991	0,046851	0	0	0	0	0	0	0	0
100304181	LOC100304181	uncharacterized LOC100304181	0	0	0	0	2,0411403	0,0390939	0	0	0	0
100304309	LOC100304309	uncharacterized LOC100304309	0	0	0	0	1,7863466	0,039427	0	0	0	0
100304328	LOC100304328	uncharacterized LOC100304328	2,03174037	0,03517	0	0	0	0	0	0	0	0
100304388	LOC100304388	uncharacterized LOC100304388	0	0	0	0	3,9213976	0,0026301	4,19550833	0,001094	3,6610011	0,0025166
100381420	LOC100381420	uncharacterized LOC100381420	0	0	0	0	1,8592934	0,0454288	1,90279821	0,034698	0	0
100381430	LOC100381430	uncharacterized LOC100381430	0	0	0	0	0	0	4,30935385	0,02461	0	0
100381474	LOC100381474	uncharacterized LOC100381474	0	0	0	0	3,0989998	0,0036333	2,86896705	0,005187	2,9610085	0,0037406
100381493	LOC100381493	uncharacterized LOC100381493	-2,0304927	0,010633	0	0	0	0	1,5594228	0,048572	0	0
100381535	LOC100381535	uncharacterized LOC100381535	0	0	1,5548614	0,00186	0	0	0	0	0	0
100381550	LOC100381550	uncharacterized LOC100381550	-2,2776491	4,13E-05	-2,3692866	1,963E-05	-2,311362	3,037E-05	0	0	0	0
100381562	LOC100381562	uncharacterized LOC100381562	-1,6797112	0,020507	0	0	0	0	0	0	0	0
100381570	LOC100381570	uncharacterized LOC100381570	-1,641366	0,001577	0	0	0	0	0	0	0	0
100381574	LOC100381574	uncharacterized LOC100381574	0	0	0	0	2,6103827	0,0229259	0	0	0	0
100381723	LOC100381723	uncharacterized LOC100381723	0	0	0	0	0	0	2,34938589	0,013943	0	0
100381760	pco065117	uncharacterized LOC100381760	-1,7334226	0,015735	-1,7031826	0,0174492	-1,72786	0,015842	0	0	0	0
100381855	LOC100381855	uncharacterized LOC100381855	-3,4701058	0,032257	0	0	0	0	0	0	0	0
100381900	LOC100381900	uncharacterized LOC100381900	0	0	0	0	6,3215116	0,0040394	5,84620768	0,005811	4,0489193	0,0356518
100381927	LOC100381927	uncharacterized LOC100381927	-3,2678582	0,029546	0	0	0	0	0	0	0	0
100381936	LOC100381936	uncharacterized LOC100381936	0	0	2,15504174	0,0009996	0	0	0	0	-1,780835	0,0052728
100382105	LOC100382105	uncharacterized LOC100382105	0	0	0	0	3,1680696	0,0020031	3,35199275	0,000934	3,4623089	0,0005956
100382148	LOC100382148	uncharacterized LOC100382148	0	0	0	0	2,4040697	0,009472	0	0	2,2022966	0,0120173
100382289	LOC100382289	uncharacterized LOC100382289	0	0	0	0	0	0	1,69298463	0,000883	0	0
100382339	LOC100382339	uncharacterized LOC100382339	-2,4037984	0,012211	0	0	0	0	1,9329933	0,04228	0	0
100382354	LOC100382354	uncharacterized LOC100382354	0	0	0	0	0	0	3,77364533	0,012311	2,9728643	0,0345644
100382367	TIDP2768	uncharacterized LOC100382367	0	0	3,22683128	0,0082175	0	0	0	0	-3,018104	0,0090494
100382396	LOC100382396	uncharacterized LOC100382396	0	0	-3,9564797	0,0436808	0	0	0	0	0	0
100382441	LOC100382441	uncharacterized LOC100382441	0	0	0	0	0	0	-2,8402216	0,031518	0	0
100382444	LOC100382444	uncharacterized LOC100382444	0	0	0	0	0	0	0	0	2,0265528	0,0164816
100382497	LOC100382497	uncharacterized LOC100382497	-2,0494018	0,030554	-2,855621	0,003631	-2,331819	0,0139996	0	0	0	0
100382515	LOC100382515	uncharacterized LOC100382515	0	0	0	0	4,9829374	0,009279	3,3614184	0,042861	4,1328256	0,0165663
100382538	IDP1623	uncharacterized LOC100382538	1,73127411	0,016015	0	0	0	0	-1,9410168	0,004934	0	0
100382629	LOC100382629	uncharacterized LOC100382629	0	0	-1,8996756	0,0451594	-2,413845	0,0113922	0	0	0	0
100382694	LOC100382694	uncharacterized LOC100382694	0	0	3,56556333	0,0235143	0	0	0	0	-2,95644	0,0303857
100382748	LOC100382748	uncharacterized LOC100382748	0	0	2,0125119	0,0249562	2,0379526	0,0229185	0	0	0	0
100382837	LOC100382837	uncharacterized LOC100382837	0	0	0	0	2,5536243	0,0334117	2,55357726	0,027933	2,355677	0,0375352
100382848	LOC100382848	uncharacterized LOC100382848	0	0	3,96914505	0,0001805	0	0	0	0	-3,49262	0,0005966
100382849	LOC100382849	uncharacterized LOC100382849	0	0	4,47168942	0,0193968	0	0	0	0	0	0

			Yuc x Ctr		Azo x Ctr		AzoYuc x Ctr		AzoYuc x Yuc		AzoYuc x Azo	
Gene ID	Gene Symbol	Gene Description	Log2(FC)	pvalue	Log2(FC)	pvalue	Log2(FC)	pvalue	Log2(FC)	pvalue	Log2(FC)	pvalue
100382925	si946015a02(578)	uncharacterized LOC100382925	0	0	0	0	2,054934	0,0101408	2,59996171	0,001178	2,976281	0,0002169
100382927	LOC100382927	uncharacterized LOC100382927	0	0	0	0	0	0	1,60503615	0,027716	0	0
100382929	LOC100382929	uncharacterized LOC100382929	0	0	-2,8167795	0,0486851	0	0	0	0	0	0
100382956	LOC100382956	uncharacterized LOC100382956	0	0	0	0	0	0	3,99763118	0,00407	0	0
100383117	LOC100383117	uncharacterized LOC100383117	0	0	2,41463592	0,0177566	2,102489	0,0402402	0	0	0	0
100383128	LOC100383128	uncharacterized LOC100383128	0	0	0	0	1,9428979	0,0083849	1,64149021	0,017469	1,5732294	0,0202335
100383285	LOC100383285	uncharacterized LOC100383285	0	0	0	0	0	0	2,10368189	0,013187	0	0
100383323	LOC100383323	uncharacterized LOC100383323	0	0	-2,7510637	0,0084711	0	0	0	0	2,7671069	0,0070834
100383331	LOC100383331	uncharacterized LOC100383331	0	0	0	0	2,8640129	0,0463563	2,72542431	0,049917	0	0
100383493	LOC100383493	uncharacterized LOC100383493	1,87621016	0,002434	0	0	0	0	-1,5429123	0,010905	0	0
100383495	LOC100383495	uncharacterized LOC100383495	-1,9966857	0,021652	-2,4521667	0,0050428	-3,37498	0,0001698	0	0	0	0
100383497	LOC100383497	uncharacterized LOC100383497	2,72476415	0,0066	0	0	0	0	-1,9928791	0,027168	0	0
100383501	TIDP3746	uncharacterized LOC100383501	0	0	0	0	1,9905047	0,0440354	0	0	1,9473149	0,033465
100383508	LOC100383508	uncharacterized LOC100383508	0	0	0	0	5,5116168	0,0009476	3,48216997	0,008465	3,5733036	0,0064556
100383513	LOC100383513	uncharacterized LOC100383513	0	0	0	0	0	0	-1,7237022	0,004179	0	0
100383606	LOC100383606	uncharacterized LOC100383606	0	0	0	0	2,6308219	0,0128562	0	0	0	0
100383614	LOC100383614	uncharacterized LOC100383614	0	0	0	0	0	0	1,54789657	0,040742	0	0
100383655	LOC100383655	uncharacterized LOC100383655	0	0	0	0	-2,284618	0,0458934	0	0	0	0
100383661	LOC100383661	uncharacterized LOC100383661	0	0	0	0	0	0	2,13209881	0,036345	2,0010212	0,0452877
100383715	LOC100383715	uncharacterized LOC100383715	0	0	0	0	-3,35387	0,0489453	0	0	0	0
100383726	LOC100383726	uncharacterized LOC100383726	0	0	0	0	0	0	4,44120866	0,022306	4,6671389	0,0163339
100383771	LOC100383771	uncharacterized LOC100383771	0	0	-2,1493417	0,0250814	-2,818597	0,0050164	0	0	0	0
100383810	LOC100383810	uncharacterized LOC100383810	0	0	0	0	3,9958118	0,0417213	0	0	0	0
100383860	LOC100383860	uncharacterized LOC100383860	-1,5753899	0,043578	0	0	-1,527836	0,0469191	0	0	0	0
100383887	LOC100383887	uncharacterized LOC100383887	0	0	0	0	-1,759915	0,018578	0	0	0	0
100384029	LOC100384029	uncharacterized LOC100384029	0	0	0	0	0	0	3,93541576	0,049068	0	0
100384037	LOC100384037	uncharacterized LOC100384037	0	0	0	0	0	0	2,17677952	0,00713	1,7971558	0,0247234
100384052	LOC100384052	uncharacterized LOC100384052	0	0	0	0	0	0	-1,8552409	0,008571	0	0
100384061	LOC100384061	uncharacterized LOC100384061	-4,7790753	0,011675	-5,7985179	0,0027272	-6,67361	0,000855	0	0	0	0
100384180	LOC100384180	uncharacterized LOC100384180	0	0	0	0	0	0	4,41842422	0,030871	0	0
100384269	LOC100384269	uncharacterized LOC100384269	0	0	0	0	0	0	1,66791169	0,011891	1,724205	0,0091079
100384296	umc2190	uncharacterized LOC100384296	-2,7213779	0,043675	0	0	0	0	0	0	0	0
100384354	LOC100384354	uncharacterized LOC100384354	0	0	0	0	0	0	0	0	3,9654267	0,0145911
100384436	LOC100384436	uncharacterized LOC100384436	-2,3394191	0,000572	-1,9400561	0,0036117	-1,541375	0,0194644	0	0	0	0
100384533	LOC100384533	uncharacterized LOC100384533	0	0	0	0	3,7017382	0,0022714	3,52768387	0,002397	3,9339292	0,0008279
100384790	LOC100384790	uncharacterized LOC100384790	-2,6551571	0,004104	0	0	0	0	0	0	0	0
100384837	LOC100384837	uncharacterized LOC100384837	0	0	0	0	0	0	2,09214973	0,001682	0	0
100384854	LOC100384854	uncharacterized LOC100384854	0	0	0	0	0	0	3,07894011	0,049492	0	0
100500963	LOC100500963	uncharacterized LOC100500963	0	0	0	0	-2,004149	0,0454029	0	0	0	0
100501018	LOC100501018	uncharacterized LOC100501018	3,20558051	0,023657	0	0	0	0	-2,5443399	0,029216	0	0
100501100	LOC100501100	uncharacterized LOC100501100	0	0	0	0	0	0	0	0	2,8421251	0,0093633
100501222	LOC100501222	uncharacterized LOC100501222	0	0	0	0	0	0	2,23119321	0,033538	0	0

			Yuc x Ctr		Azo x Ctr		AzoYuc x Ctr		AzoYuc x Yuc		AzoYuc x Azo	
Gene ID	Gene Symbol	Gene Description	Log2(FC)	pvalue	Log2(FC)	pvalue	Log2(FC)	pvalue	Log2(FC)	pvalue	Log2(FC)	pvalue
100501255	LOC100501255	uncharacterized LOC100501255	0	0	0	0	0	0	0	0	2,7011054	0,029455
100501315	LOC100501315	uncharacterized LOC100501315	-2,2024784	0,003465	-2,5769773	0,0006512	-2,466306	0,001065	0	0	0	0
100501385	LOC100501385	uncharacterized LOC100501385	0	0	1,87142213	0,0347413	0	0	0	0	0	0
100501426	LOC100501426	uncharacterized LOC100501426	-2,2124459	0,008543	-2,5370923	0,0026725	-2,409354	0,0041214	0	0	0	0
100501437	LOC100501437	uncharacterized LOC100501437	4,84974693	0,001365	3,34441433	0,0321001	3,7595088	0,0146255	0	0	0	0
100501464	LOC100501464	uncharacterized LOC100501464	0	0	0	0	0	0	0	0	1,83494	0,0371452
100501650	LOC100501650	uncharacterized LOC100501650	0	0	0	0	-3,553887	0,0166253	0	0	0	0
100501673	LOC100501673	uncharacterized LOC100501673	0	0	0	0	0	0	0	0	3,4311799	0,0128013
100501712	LOC100501712	uncharacterized LOC100501712	0	0	0	0	2,3370358	0,0076394	1,93319515	0,022192	0	0
100501753	LOC100501753	uncharacterized LOC100501753	0	0	0	0	1,9740268	0,0096972	0	0	0	0
100501789	LOC100501789	uncharacterized LOC100501789	-1,8537725	0,006651	0	0	0	0	0	0	0	0
100501821	LOC100501821	uncharacterized LOC100501821	0	0	0	0	0	0	-1,5548743	0,019277	0	0
100501921	LOC100501921	uncharacterized LOC100501921	0	0	0	0	3,8776976	0,0117935	0	0	0	0
100501994	LOC100501994	uncharacterized LOC100501994	-3,2247412	0,00068	0	0	-1,783318	0,0219269	0	0	0	0
100502152	LOC100502152	uncharacterized LOC100502152	1,51115305	0,01947	0	0	0	0	0	0	0	0
100502153	LOC100502153	uncharacterized LOC100502153	3,54767514	0,044717	0	0	0	0	0	0	0	0
100502159	LOC100502159	uncharacterized LOC100502159	0	0	0	0	1,5596231	0,041018	0	0	1,9689936	0,0092499
100502162	LOC100502162	uncharacterized LOC100502162	0	0	0	0	0	0	3,71026567	0,028321	0	0
100502267	LOC100502267	uncharacterized LOC100502267	-2,9090924	0,031365	0	0	0	0	3,01737658	0,022479	0	0
100502313	LOC100502313	uncharacterized LOC100502313	0	0	0	0	2,1894569	0,0029093	0	0	2,2112055	0,0018373
100502421	LOC100502421	uncharacterized LOC100502421	0	0	0	0	0	0	0	0	-2,240741	0,0302194
100502453	LOC100502453	uncharacterized LOC100502453	3,63802724	0,030234	0	0	0	0	0	0	0	0
100502455	LOC100502455	uncharacterized LOC100502455	0	0	0	0	0	0	-3,5254725	0,02686	0	0
100502500	LOC100502500	uncharacterized LOC100502500	0	0	0	0	-3,502738	0,0429457	0	0	0	0
100856921	LOC100856921	uncharacterized LOC100856921	0	0	0	0	3,1596638	0,0362987	0	0	0	0
103625797	LOC103625797	uncharacterized LOC103625797	0	0	0	0	0	0	0	0	3,9319702	0,0421939
103625887	LOC103625887	uncharacterized LOC103625887	0	0	0	0	0	0	1,59923907	0,029138	0	0
103626186	LOC103626186	uncharacterized LOC103626186	0	0	2,63018993	0,0359382	0	0	0	0	-4,020825	0,0026049
103626188	LOC103626188	uncharacterized LOC103626188	0	0	0	0	0	0	-3,5858958	0,043201	0	0
103626226	LOC103626226	uncharacterized LOC103626226	0	0	0	0	1,8645859	0,0381392	0	0	0	0
103626267	LOC103626267	uncharacterized LOC103626267	0	0	0	0	3,6718989	0,0208766	0	0	0	0
103626314	LOC103626314	uncharacterized LOC103626314	0	0	0	0	4,1342948	0,0064015	2,6166931	0,03145	0	0
103626342	LOC103626342	uncharacterized LOC103626342	0	0	-2,5203521	0,0349805	0	0	0	0	0	0
103626490	LOC103626490	uncharacterized LOC103626490	2,07451983	0,007357	0	0	0	0	-2,1975874	0,00378	0	0
103626595	LOC103626595	uncharacterized LOC103626595	0	0	0	0	2,7272749	0,0160042	2,35334822	0,028938	0	0
103626607	LOC103626607	uncharacterized LOC103626607	-4,6644863	8,46E-05	-4,9494464	3,379E-05	-3,436276	0,0013114	0	0	0	0
103626673	LOC103626673	uncharacterized LOC103626673	-4,9612486	0,010739	0	0	0	0	0	0	0	0
103626751	LOC103626751	uncharacterized LOC103626751	0	0	0	0	3,9280012	0,040315	5,31252	0,00555	0	0
103626979	LOC103626979	uncharacterized LOC103626979	-3,5764854	0,018558	-3,1775686	0,0225318	0	0	0	0	0	0
103627150	LOC103627150	uncharacterized LOC103627150	0	0	0	0	0	0	5,15563946	0,019765	0	0
103627207	LOC103627207	uncharacterized LOC103627207	4,59150342	0,02028	0	0	0	0	0	0	0	0
103627564	LOC103627564	uncharacterized LOC103627564	-1,8676748	0,033834	0	0	0	0	0	0	0	0

			Yuc x Ctr		Azo x Ctr		AzoYuc x Ctr		AzoYuc x Yuc		AzoYuc x Azo	
Gene ID	Gene Symbol	Gene Description	Log2(FC)	pvalue	Log2(FC)	pvalue	Log2(FC)	pvalue	Log2(FC)	pvalue	Log2(FC)	pvalue
103627782	LOC103627782	uncharacterized LOC103627782	0	0	0	0	3,2832027	0,0237089	0	0	0	0
103628086	LOC103628086	uncharacterized LOC103628086	0	0	-2,5245819	0,0277917	0	0	0	0	0	0
103628099	LOC103628099	uncharacterized LOC103628099	0	0	0	0	-3,519991	0,0039293	-2,4837318	0,045902	-2,615639	0,0334777
103628528	LOC103628528	uncharacterized LOC103628528	0	0	0	0	2,0395687	0,0223836	2,56801855	0,004462	0	0
103628985	LOC103628985	uncharacterized LOC103628985	0	0	0	0	2,3979842	0,0297683	0	0	0	0
103629117	LOC103629117	uncharacterized LOC103629117	0	0	0	0	1,643385	0,0416101	1,79768176	0,023804	0	0
103629455	LOC103629455	uncharacterized LOC103629455	0	0	0	0	0	0	4,4275048	0,008502	0	0
103629667	LOC103629667	uncharacterized LOC103629667	0	0	0	0	2,9627893	0,0007488	2,70042707	0,001635	2,6483745	0,0018757
103629968	LOC103629968	uncharacterized LOC103629968	-4,4906845	0,012889	0	0	-3,78517	0,03607	0	0	0	0
103630054	LOC103630054	uncharacterized LOC103630054	-4,1156736	0,003471	-3,5312421	0,0065973	-3,761572	0,004423	0	0	0	0
103630114	LOC103630114	uncharacterized LOC103630114	0	0	0	0	2,1732191	0,0067392	2,21904726	0,005365	2,4839473	0,0018549
103630157	LOC103630157	uncharacterized LOC103630157	0	0	-3,9072894	0,0044644	-2,53106	0,0264017	-2,4335405	0,031256	0	0
103630187	LOC103630187	uncharacterized LOC103630187	0	0	-2,4962489	0,0477694	0	0	0	0	0	0
103630270	LOC103630270	uncharacterized LOC103630270	0	0	0	0	1,6438503	0,0366712	0	0	0	0
103630401	LOC103630401	uncharacterized LOC103630401	0	0	0	0	0	0	-2,7046367	0,030616	0	0
103630456	LOC103630456	uncharacterized LOC103630456	0	0	0	0	-1,672361	0,0096266	-1,6819194	0,008018	-1,697296	0,0070238
103630492	LOC103630492	uncharacterized LOC103630492	0	0	0	0	0	0	0	0	3,0167637	0,0451908
103630642	LOC103630642	uncharacterized LOC103630642	-3,6693108	0,01093	-3,3044487	0,0204187	0	0	0	0	0	0
103630646	LOC103630646	uncharacterized LOC103630646	-3,8348523	0,028183	0	0	0	0	3,53394197	0,040415	0	0
103630787	LOC103630787	uncharacterized LOC103630787	0	0	0	0	0	0	0	0	2,4912257	0,0432315
103631148	LOC103631148	uncharacterized LOC103631148	0	0	-1,5482701	0,0393487	-1,964826	0,0097153	0	0	0	0
103631927	LOC103631927	uncharacterized LOC103631927	0	0	0	0	-2,046527	0,0049062	-1,7675086	0,014723	-1,574249	0,0298195
103631979	LOC103631979	uncharacterized LOC103631979	0	0	0	0	1,6054604	0,0105881	1,62096751	0,008603	0	0
103632022	LOC103632022	uncharacterized LOC103632022	-2,7124562	0,007472	0	0	0	0	0	0	0	0
103632037	LOC103632037	uncharacterized LOC103632037	4,31570851	0,006831	0	0	0	0	-3,6451904	0,013098	0	0
103632202	LOC103632202	uncharacterized LOC103632202	0	0	-1,7488638	0,0479126	0	0	0	0	0	0
103632216	LOC103632216	uncharacterized LOC103632216	-2,5795894	0,043564	0	0	0	0	0	0	0	0
103632486	LOC103632486	uncharacterized LOC103632486	0	0	0	0	0	0	2,89627738	0,048783	0	0
103632573	LOC103632573	uncharacterized LOC103632573	0	0	0	0	4,1360194	0,0036721	3,54108209	0,005926	4,3058749	0,0014163
103632575	LOC103632575	uncharacterized LOC103632575	0	0	-1,9601563	0,047737	-2,363157	0,0215769	0	0	0	0
103632729	LOC103632729	uncharacterized LOC103632729	0	0	0	0	0	0	0	0	3,2415186	0,037223
103632806	LOC103632806	uncharacterized LOC103632806	0	0	-1,5039568	0,0083721	-1,768583	0,0021068	0	0	0	0
103632813	LOC103632813	uncharacterized LOC103632813	0	0	0	0	0	0	0	0	1,807285	0,0413155
103633165	LOC103633165	uncharacterized LOC103633165	2,2000288	0,023445	0	0	0	0	-3,8144388	0,000194	0	0
103633395	LOC103633395	uncharacterized LOC103633395	-3,3812613	0,036698	0	0	0	0	3,67041831	0,020238	0	0
103634133	LOC103634133	uncharacterized LOC103634133	0	0	0	0	0	0	3,83459224	0,007537	3,1333139	0,0169912
103634163	LOC103634163	uncharacterized LOC103634163	0	0	0	0	-2,066181	0,00113	-2,3155655	0,000192	0	0
103634362	LOC103634362	uncharacterized LOC103634362	0	0	0	0	5,0079816	0,0069494	6,3925013	0,00057	3,1821271	0,0405077
103634774	LOC103634774	uncharacterized LOC103634774	0	0	0	0	-1,944158	0,0384353	0	0	0	0
103634819	LOC103634819	uncharacterized LOC103634819	-2,6044598	0,005275	-3,0117688	0,0015928	-1,991433	0,0199123	0	0	0	0
103635036	LOC103635036	uncharacterized LOC103635036	0	0	0	0	2,3968726	0,0317039	0	0	0	0
103635054	LOC103635054	uncharacterized LOC103635054	0	0	0	0	3,4427292	0,0432783	3,57630464	0,030214	3,6016583	0,0260728

			Yuc x Ctr		Azo x Ctr		AzoYuc x Ctr		AzoYuc x Yuc		AzoYuc x Azo	
Gene ID	Gene Symbol	Gene Description	Log2(FC)	pvalue	Log2(FC)	pvalue	Log2(FC)	pvalue	Log2(FC)	pvalue	Log2(FC)	pvalue
103635080	LOC103635080	uncharacterized LOC103635080	0	0	0	0	0	0	-2,3373519	0,046541	-2,544301	0,0282851
103635104	LOC103635104	uncharacterized LOC103635104	2,77402668	0,014789	0	0	2,6572013	0,0192934	0	0	0	0
103635293	LOC103635293	uncharacterized LOC103635293	3,51975629	0,041151	0	0	0	0	0	0	0	0
103635799	LOC103635799	uncharacterized LOC103635799	0	0	0	0	0	0	0	0	4,6921221	0,0473411
103635851	LOC103635851	uncharacterized LOC103635851	0	0	0	0	0	0	0	0	2,1253508	0,0187038
103635907	LOC103635907	uncharacterized LOC103635907	0	0	0	0	-1,868048	0,0199444	0	0	0	0
103636109	LOC103636109	uncharacterized LOC103636109	0	0	0	0	0	0	0	0	-4,795074	0,0168566
103636150	LOC103636150	uncharacterized LOC103636150	-3,6460923	0,033223	0	0	0	0	0	0	0	0
103636161	LOC103636161	uncharacterized LOC103636161	0	0	0	0	0	0	0	0	4,9258979	0,0469874
103636287	LOC103636287	uncharacterized LOC103636287	1,96213298	0,020226	0	0	0	0	0	0	0	0
103636932	LOC103636932	uncharacterized LOC103636932	0	0	0	0	0	0	3,31145362	0,032881	3,0286339	0,0415457
103637263	LOC103637263	uncharacterized LOC103637263	-1,7299961	0,002276	0	0	0	0	0	0	0	0
103637645	LOC103637645	uncharacterized LOC103637645	-4,2105922	0,012158	0	0	0	0	5,13729342	0,001558	0	0
103638453	LOC103638453	uncharacterized LOC103638453	0	0	0	0	0	0	-3,3922819	0,034208	0	0
103638694	LOC103638694	uncharacterized LOC103638694	0	0	-4,4665442	0,016106	0	0	0	0	0	0
103638710	LOC103638710	uncharacterized LOC103638710	0	0	0	0	0	0	2,84527583	0,014617	0	0
103638978	LOC103638978	uncharacterized LOC103638978	0	0	0	0	0	0	0	0	-1,59068	0,0398543
103639178	LOC103639178	uncharacterized LOC103639178	0	0	0	0	2,9908945	0,0418693	0	0	0	0
103639197	LOC103639197	uncharacterized LOC103639197	0	0	0	0	0	0	2,11865577	0,00549	0	0
103639235	LOC103639235	uncharacterized LOC103639235	0	0	0	0	-3,394161	0,044662	0	0	0	0
103639247	LOC103639247	uncharacterized LOC103639247	7,562166	0,011149	0	0	0	0	-6,5246348	0,02181	0	0
103639764	LOC103639764	uncharacterized LOC103639764	0	0	0	0	0	0	3,922766	0,034356	3,8731759	0,0353384
103639870	LOC103639870	uncharacterized LOC103639870	-2,5379372	0,001779	-2,6103786	0,0012719	-2,913624	0,000348	0	0	0	0
103639888	LOC103639888	uncharacterized LOC103639888	0	0	0	0	2,5986554	0,0215162	0	0	2,1968008	0,0459025
103639967	LOC103639967	uncharacterized LOC103639967	-5,7995759	0,041997	0	0	0	0	0	0	0	0
103640141	LOC103640141	uncharacterized LOC103640141	0	0	0	0	0	0	0	0	3,5531204	0,0444335
103640263	LOC103640263	uncharacterized LOC103640263	0	0	0	0	3,2690645	0,0363433	0	0	0	0
103640528	LOC103640528	uncharacterized LOC103640528	0	0	0	0	1,8777976	0,0284434	0	0	0	0
103640565	LOC103640565	uncharacterized LOC103640565	0	0	0	0	5,4002411	0,0123685	0	0	5,087124	0,0184524
103640704	LOC103640704	uncharacterized LOC103640704	-2,2324493	0,003577	0	0	-1,813349	0,0149625	0	0	0	0
103640844	LOC103640844	uncharacterized LOC103640844	-4,0408492	0,037256	0	0	0	0	0	0	0	0
103641148	LOC103641148	uncharacterized LOC103641148	0	0	0	0	1,7751772	0,0029788	1,76304646	0,002932	1,6896258	0,0042029
103641300	LOC103641300	uncharacterized LOC103641300	0	0	0	0	0	0	-6,065615	0,030724	0	0
103641521	LOC103641521	uncharacterized LOC103641521	0	0	-4,4457555	0,0494599	0	0	0	0	0	0
103641878	LOC103641878	uncharacterized LOC103641878	0	0	0	0	0	0	3,27214117	0,024032	0	0
103641914	LOC103641914	uncharacterized LOC103641914	-2,2986223	0,027706	0	0	0	0	0	0	0	0
103642028	LOC103642028	uncharacterized LOC103642028	0	0	0	0	0	0	0	0	4,2565766	0,0475553
103642063	LOC103642063	uncharacterized LOC103642063	0	0	-1,7138308	0,0235265	-1,877492	0,0143313	-1,6362415	0,031574	0	0
103642102	LOC103642102	uncharacterized LOC103642102	0	0	-1,7784653	0,0151658	-1,863881	0,0112747	0	0	0	0
103642199	LOC103642199	uncharacterized LOC103642199	0	0	0	0	0	0	3,60944591	0,041955	5,1582631	0,0077996
103642371	LOC103642371	uncharacterized LOC103642371	0	0	0	0	0	0	-1,9768354	0,030742	0	0
103642641	LOC103642641	uncharacterized LOC103642641	0	0	0	0	0	0	-2,0400399	0,02859	0	0

			Yuc x Ctr		Azo x Ctr		AzoYuc x Ctr		AzoYuc x Yuc		AzoYuc x Azo	
Gene ID	Gene Symbol	Gene Description	Log2(FC)	pvalue	Log2(FC)	pvalue	Log2(FC)	pvalue	Log2(FC)	pvalue	Log2(FC)	pvalue
103642963	LOC103642963	uncharacterized LOC103642963	-2,1404342	0,033037	0	0	0	0	0	0	0	0
103643021	LOC103643021	uncharacterized LOC103643021	-1,7678963	0,031216	-1,7070063	0,035442	0	0	0	0	0	0
103643056	LOC103643056	uncharacterized LOC103643056	0	0	0	0	1,6782644	0,0107969	1,6879912	0,00926	1,8654311	0,0040119
103643065	LOC103643065	uncharacterized LOC103643065	0	0	0	0	0	0	1,67980795	0,04332	2,4025119	0,0049833
103643290	LOC103643290	uncharacterized LOC103643290	3,36822671	0,001646	0	0	0	0	-2,8495773	0,005358	0	0
103643416	LOC103643416	uncharacterized LOC103643416	0	0	0	0	-1,825959	0,0355231	0	0	0	0
103643535	LOC103643535	uncharacterized LOC103643535	0	0	0	0	-1,896091	0,0346112	0	0	0	0
103643846	LOC103643846	uncharacterized LOC103643846	-3,2322436	0,036656	0	0	0	0	0	0	0	0
103644017	LOC103644017	uncharacterized LOC103644017	0	0	0	0	-1,598531	0,0161724	0	0	0	0
103644253	LOC103644253	uncharacterized LOC103644253	-5,458779	4,28E-05	-8,7718837	1,051E-07	-7,840436	2,005E-06	0	0	0	0
103644320	LOC103644320	uncharacterized LOC103644320	7,62294481	0,007468	0	0	0	0	-5,34487	0,046806	0	0
103644343	LOC103644343	uncharacterized LOC103644343	-3,961011	0,025058	-3,3058425	0,0473724	-5,540971	0,0041458	0	0	0	0
103644532	LOC103644532	uncharacterized LOC103644532	0	0	0	0	4,4315399	0,0055173	0	0	0	0
103645040	LOC103645040	uncharacterized LOC103645040	0	0	0	0	0	0	0	0	3,1824124	0,0283566
103645216	LOC103645216	uncharacterized LOC103645216	0	0	-2,7319887	0,0349835	-2,636755	0,0399866	0	0	0	0
103645334	LOC103645334	uncharacterized LOC103645334	0	0	0	0	0	0	4,18885184	0,026417	0	0
103645597	LOC103645597	uncharacterized LOC103645597	-5,1278382	0,004983	-3,9032871	0,0166956	-6,345912	0,0005104	0	0	0	0
103645607	LOC103645607	uncharacterized LOC103645607	0	0	0	0	0	0	-1,6868151	0,023795	0	0
103645745	LOC103645745	uncharacterized LOC103645745	-3,8340074	0,034064	0	0	0	0	0	0	0	0
103645891	LOC103645891	uncharacterized LOC103645891	0	0	0	0	0	0	3,947696	0,042434	0	0
103645907	LOC103645907	uncharacterized LOC103645907	0	0	0	0	-3,384372	0,0374685	-3,4515497	0,031039	-3,529511	0,0263096
103646102	LOC103646102	uncharacterized LOC103646102	0	0	0	0	0	0	3,43943491	0,013014	0	0
103646241	LOC103646241	uncharacterized LOC103646241	-3,4180379	0,023397	0	0	0	0	0	0	0	0
103646371	LOC103646371	uncharacterized LOC103646371	0	0	0	0	0	0	3,56505769	0,002927	2,3224755	0,0303713
103646409	LOC103646409	uncharacterized LOC103646409	-4,1106205	0,021708	-3,2549995	0,0491272	-3,969945	0,0217119	0	0	0	0
103646480	LOC103646480	uncharacterized LOC103646480	0	0	0	0	4,1254783	0,0232844	3,21398426	0,049996	0	0
103646825	LOC103646825	uncharacterized LOC103646825	-5,9713028	1,53E-05	-6,3810085	4,593E-06	-5,592078	7,331E-06	0	0	0	0
103647051	LOC103647051	uncharacterized LOC103647051	0	0	0	0	2,5147979	0,0225281	3,1435458	0,004807	2,4703537	0,021352
103647083	LOC103647083	uncharacterized LOC103647083	0	0	-3,637792	0,0244604	0	0	0	0	0	0
103647366	LOC103647366	uncharacterized LOC103647366	0	0	0	0	-4,085041	0,0370623	0	0	0	0
103647383	LOC103647383	uncharacterized LOC103647383	0	0	0	0	0	0	1,89559822	0,021749	0	0
103647449	LOC103647449	uncharacterized LOC103647449	-1,7335224	0,007034	0	0	0	0	1,84810526	0,003203	0	0
103647463	LOC103647463	uncharacterized LOC103647463	-2,8159784	0,04073	0	0	0	0	2,6672095	0,048594	0	0
103647542	LOC103647542	uncharacterized LOC103647542	0	0	-2,2965539	0,0112244	0	0	0	0	0	0
103647707	LOC103647707	uncharacterized LOC103647707	0	0	0	0	2,8443994	0,025681	0	0	0	0
103647724	LOC103647724	uncharacterized LOC103647724	0	0	0	0	0	0	0	0	-1,643889	0,0376393
103647763	LOC103647763	uncharacterized LOC103647763	0	0	0	0	0	0	3,61636046	0,01164	4,5887074	0,0028884
103647937	LOC103647937	uncharacterized LOC103647937	4,21628508	0,008943	3,98606118	0,0135152	3,3187333	0,042889	0	0	0	0
103647941	LOC103647941	uncharacterized LOC103647941	0	0	0	0	0	0	1,78954163	0,044458	0	0
103648014	LOC103648014	uncharacterized LOC103648014	-4,0458684	0,000353	-2,9126908	0,0069076	-2,751817	0,0103177	0	0	0	0
103648023	LOC103648023	uncharacterized LOC103648023	-4,6124905	0,027134	0	0	0	0	0	0	0	0
103648084	LOC103648084	uncharacterized LOC103648084	0	0	0	0	1,6153944	0,0452609	0	0	1,7527944	0,0252852

			Yuc x Ctr		Azo x Ctr		AzoYuc x Ctr		AzoYuc x Yuc		AzoYuc x Azo	
Gene ID	Gene Symbol	Gene Description	Log2(FC)	pvalue	Log2(FC)	pvalue	Log2(FC)	pvalue	Log2(FC)	pvalue	Log2(FC)	pvalue
103648144	LOC103648144	uncharacterized LOC103648144	0	0	4,22346462	0,0179665	3,7788839	0,03538	0	0	0	0
103648231	LOC103648231	uncharacterized LOC103648231	0	0	0	0	3,0690775	0,0045854	3,47371266	0,001324	3,2518841	0,0021009
103648255	LOC103648255	uncharacterized LOC103648255	0	0	0	0	0	0	1,82303968	0,013924	0	0
103648292	LOC103648292	uncharacterized LOC103648292	0	0	-6,4333484	0,0013567	-4,7441	0,0096048	0	0	0	0
103648516	LOC103648516	uncharacterized LOC103648516	0	0	0	0	0	0	0	0	4,4788761	0,0195857
103648694	LOC103648694	uncharacterized LOC103648694	0	0	-2,169471	0,0282657	0	0	0	0	0	0
103648964	LOC103648964	uncharacterized LOC103648964	0	0	0	0	3,3616563	0,0397936	3,09499408	0,041996	3,2846338	0,030479
103649041	LOC103649041	uncharacterized LOC103649041	0	0	4,65297421	0,0067388	0	0	0	0	0	0
103649177	LOC103649177	uncharacterized LOC103649177	0	0	0	0	-2,706633	0,0094197	0	0	0	0
103649203	LOC103649203	uncharacterized LOC103649203	0	0	0	0	3,7418612	0,0445396	0	0	0	0
103649645	LOC103649645	uncharacterized LOC103649645	0	0	0	0	0	0	1,9669298	0,042392	0	0
103650223	LOC103650223	uncharacterized LOC103650223	0	0	0	0	2,8785969	0,0074497	3,82607811	0,000525	4,0553859	0,0002418
103650242	LOC103650242	uncharacterized LOC103650242	0	0	0	0	-3,921793	0,0456522	0	0	0	0
103650294	LOC103650294	uncharacterized LOC103650294	0	0	0	0	0	0	0	0	2,0413068	0,0295449
103650399	LOC103650399	uncharacterized LOC103650399	-3,4259845	0,043128	-4,151307	0,0152091	-4,006555	0,0186481	0	0	0	0
103650412	LOC103650412	uncharacterized LOC103650412	0	0	-3,5532462	0,0295472	0	0	0	0	0	0
103650747	LOC103650747	uncharacterized LOC103650747	0	0	0	0	5,5139802	0,0024949	0	0	3,9376567	0,0174937
103650806	LOC103650806	uncharacterized LOC103650806	0	0	0	0	0	0	0	0	2,0298627	0,0143669
103650972	LOC103650972	uncharacterized LOC103650972	0	0	0	0	-4,626738	0,011471	-4,5794939	0,011638	0	0
103651194	LOC103651194	uncharacterized LOC103651194	0	0	0	0	-3,36236	0,0474485	0	0	0	0
103651445	LOC103651445	uncharacterized LOC103651445	0	0	-1,5334358	0,0342305	0	0	0	0	0	0
103651769	LOC103651769	uncharacterized LOC103651769	0	0	0	0	4,1435676	0,0002869	2,99088482	0,004169	2,7726323	0,007151
103652167	LOC103652167	uncharacterized LOC103652167	0	0	0	0	0	0	0	0	3,0037782	0,0491016
103652297	LOC103652297	uncharacterized LOC103652297	0	0	-4,6357558	0,0129655	0	0	0	0	4,3830653	0,0178091
103652852	LOC103652852	uncharacterized LOC103652852	0	0	-5,1540117	0,0371449	0	0	0	0	0	0
103652865	LOC103652865	uncharacterized LOC103652865	-6,3931987	0,000157	-3,5378047	0,0107378	-5,687684	0,0007703	0	0	0	0
103652964	LOC103652964	uncharacterized LOC103652964	0	0	0	0	0	0	1,67430709	0,017826	1,5426075	0,0285412
103653042	LOC103653042	uncharacterized LOC103653042	-2,3959663	0,006632	-1,8922244	0,0263772	0	0	0	0	0	0
103653151	LOC103653151	uncharacterized LOC103653151	0	0	0	0	2,5811686	0,0036793	2,17949516	0,011457	2,6572006	0,0022494
103653374	LOC103653374	uncharacterized LOC103653374	0	0	0	0	0	0	0	0	3,0770539	0,0102074
103653467	LOC103653467	uncharacterized LOC103653467	0	0	3,78166318	0,0462898	0	0	0	0	0	0
103653535	LOC103653535	uncharacterized LOC103653535	0	0	0	0	0	0	1,66217279	0,02218	0	0
103653616	LOC103653616	uncharacterized LOC103653616	0	0	-2,0627294	0,0389833	-2,307931	0,022768	0	0	0	0
103653665	LOC103653665	uncharacterized LOC103653665	2,56527301	0,009999	2,50404625	0,0116523	3,2273396	0,0009842	0	0	0	0
103653714	LOC103653714	uncharacterized LOC103653714	3,45054579	0,049007	0	0	4,1193113	0,0164737	0	0	0	0
103653832	LOC103653832	uncharacterized LOC103653832	-1,8317091	0,025107	0	0	0	0	0	0	0	0
103653882	LOC103653882	uncharacterized LOC103653882	-1,8828626	0,028164	-2,556919	0,004044	-2,741969	0,0023741	0	0	0	0
103654009	LOC103654009	uncharacterized LOC103654009	0	0	0	0	0	0	0	0	1,81732	0,0434783
103654201	LOC103654201	uncharacterized LOC103654201	0	0	0	0	0	0	-3,5131457	0,005363	0	0
103654253	LOC103654253	uncharacterized LOC103654253	0	0	-3,0127764	0,0007501	-3,139069	0,0005007	-1,9391078	0,034801	0	0
103654384	LOC103654384	uncharacterized LOC103654384	0	0	-1,8438432	0,0289066	0	0	0	0	0	0
103654899	LOC103654899	uncharacterized LOC103654899	0	0	0	0	0	0	0	0	3,299101	0,0356709

Gene ID	Gene Symbol	Gene Description	Yuc x Ctr		Azo x Ctr		AzoYuc x Ctr		AzoYuc x Yuc		AzoYuc x Azo	
			Log2(FC)	pvalue	Log2(FC)	pvalue	Log2(FC)	pvalue	Log2(FC)	pvalue	Log2(FC)	pvalue
103654908	LOC103654908	uncharacterized LOC103654908	-4,3017483	0,022063	-4,5276811	0,0159739	-4,558028	0,0152805	0	0	0	0
103655263	LOC103655263	uncharacterized LOC103655263	-3,0231273	0,000155	-2,6278097	0,0007145	-2,002594	0,0083425	0	0	0	0
103655353	LOC103655353	uncharacterized LOC103655353	0	0	0	0	0	0	1,57732371	0,04075	0	0
103655800	LOC103655800	uncharacterized LOC103655800	0	0	0	0	0	0	2,00083619	0,003203	0	0
103655916	LOC103655916	uncharacterized LOC103655916	0	0	-2,6171483	0,0333417	-2,49893	0,0405631	0	0	0	0
103655972	LOC103655972	uncharacterized LOC103655972	2,57911693	0,021349	0	0	0	0	0	0	0	0
103656015	LOC103656015	uncharacterized LOC103656015	0	0	0	0	0	0	0	0	-3,666822	0,0495565
103656032	LOC103656032	uncharacterized LOC103656032	0	0	0	0	3,7458755	0,0470627	0	0	0	0
107275237	LOC107275237	uncharacterized LOC107275237	0	0	0	0	0	0	0	0	-1,50559	0,0174168
107546776	LOC107546776	uncharacterized LOC107546776	0	0	0	0	3,2948727	0,0002852	0	0	2,1840255	0,0095247
109461487	LOC109461487	uncharacterized LOC109461487	0	0	0	0	0	0	0	0	-1,927291	0,0374161
109623449	LOC109623449	uncharacterized LOC109623449	0	0	1,62265233	0,0481932	0	0	0	0	0	0
109851609	LOC109851609	uncharacterized LOC109851609	0	0	0	0	0	0	4,41069561	0,036418	0	0
109939171	LOC109939171	uncharacterized LOC109939171	0	0	0	0	-1,882757	0,0406559	0	0	-1,953434	0,0294998
109939196	LOC109939196	uncharacterized LOC109939196	5,12588439	0,00026	5,42266207	0,0001015	4,2405592	0,0028065	0	0	0	0
109939475	LOC109939475	uncharacterized LOC109939475	0	0	-2,1489126	0,0012708	-1,674631	0,0102102	0	0	0	0
109939910	LOC109939910	uncharacterized LOC109939910	0	0	-4,5442737	0,0115344	0	0	0	0	0	0
109939937	LOC109939937	uncharacterized LOC109939937	-4,3590265	0,000599	-4,2552606	0,0005327	-4,947143	0,0001809	0	0	0	0
109939951	LOC109939951	uncharacterized LOC109939951	0	0	0	0	0	0	5,58794108	0,005874	4,8520888	0,0167579
109939988	LOC109939988	uncharacterized LOC109939988	0	0	0	0	0	0	0	0	3,2405897	0,02753
109940165	LOC109940165	uncharacterized LOC109940165	-2,2387345	0,040569	0	0	0	0	0	0	0	0
109940180	LOC109940180	uncharacterized LOC109940180	-4,0211591	0,029405	0	0	0	0	0	0	0	0
109940211	LOC109940211	uncharacterized LOC109940211	-4,4445271	0,017397	-4,3381073	0,0167158	0	0	0	0	0	0
109940310	LOC109940310	uncharacterized LOC109940310	0	0	0	0	3,3137425	0,0360329	0	0	0	0
109940311	LOC109940311	uncharacterized LOC109940311	-3,259625	0,02237	0	0	0	0	0	0	0	0
109940494	LOC109940494	uncharacterized LOC109940494	-4,5268468	0,004509	-5,8074024	0,0007959	-4,244252	0,0046979	0	0	0	0
109940843	LOC109940843	uncharacterized LOC109940843	0	0	0	0	0	0	2,26102932	0,038969	0	0
109940881	LOC109940881	uncharacterized LOC109940881	0	0	0	0	0	0	3,14576684	0,042031	0	0
109940894	LOC109940894	uncharacterized LOC109940894	0	0	0	0	0	0	-3,9877052	0,028293	0	0
109940945	LOC109940945	uncharacterized LOC109940945	0	0	-1,585149	0,0120763	-2,014946	0,0018122	-1,8075938	0,004917	0	0
109941468	LOC109941468	uncharacterized LOC109941468	0	0	0	0	5,2171159	0,0116617	5,63985033	0,006399	0	0
109941508	LOC109941508	uncharacterized LOC109941508	4,68962051	0,017679	0	0	0	0	0	0	0	0
109941518	LOC109941518	uncharacterized LOC109941518	-3,9099075	0,01803	0	0	0	0	0	0	0	0
109941537	LOC109941537	uncharacterized LOC109941537	0	0	0	0	0	0	3,35984389	0,021842	0	0
109941578	LOC109941578	uncharacterized LOC109941578	0	0	0	0	-3,600261	0,0400362	0	0	0	0
109941614	LOC109941614	uncharacterized LOC109941614	0	0	0	0	0	0	3,12063873	0,034877	0	0
109941808	LOC109941808	uncharacterized LOC109941808	0	0	4,72695379	0,0070573	0	0	0	0	0	0
109941825	LOC109941825	uncharacterized LOC109941825	-1,5310965	0,007908	0	0	0	0	0	0	0	0
109941899	LOC109941899	uncharacterized LOC109941899	-2,1500631	0,016384	0	0	0	0	2,20843688	0,011864	0	0
109941936	LOC109941936	uncharacterized LOC109941936	-5,8254087	0,002249	-5,0895472	0,0076021	0	0	0	0	0	0
109941998	LOC109941998	uncharacterized LOC109941998	0	0	0	0	0	0	-1,9886933	0,03913	0	0
109942217	LOC109942217	uncharacterized LOC109942217	0	0	0	0	-1,827444	0,0033395	-2,0653474	0,000707	-1,788422	0,0034316

			Yuc x Ctr		Azo x Ctr		AzoYuc x Ctr		AzoYuc x Yuc		AzoYuc x Azo	
Gene ID	Gene Symbol	Gene Description	Log2(FC)	pvalue	Log2(FC)	pvalue	Log2(FC)	pvalue	Log2(FC)	pvalue	Log2(FC)	pvalue
109942229	LOC109942229	uncharacterized LOC109942229	0	0	0	0	0	0	2,83345353	0,043201	0	0
109942265	LOC109942265	uncharacterized LOC109942265	3,24063034	0,044249	0	0	0	0	0	0	0	0
109942320	LOC109942320	uncharacterized LOC109942320	0	0	0	0	-3,394882	0,0289965	0	0	0	0
109942399	LOC109942399	uncharacterized LOC109942399	0	0	0	0	1,6487975	0,0117877	1,54352251	0,017178	0	0
109942740	LOC109942740	uncharacterized LOC109942740	0	0	0	0	0	0	4,63393908	0,020287	4,2882097	0,0281552
109942935	LOC109942935	uncharacterized LOC109942935	0	0	0	0	0	0	0	0	2,9253836	0,04051
109942952	LOC109942952	uncharacterized LOC109942952	-2,5232092	0,027551	-2,9121442	0,0126894	-2,866053	0,0133544	0	0	0	0
109943029	LOC109943029	uncharacterized LOC109943029	0	0	0	0	0	0	1,66081541	0,00764	0	0
109943038	LOC109943038	uncharacterized LOC109943038	0	0	-2,2245167	0,0202496	0	0	0	0	0	0
109943062	LOC109943062	uncharacterized LOC109943062	0	0	0	0	5,4479154	0,0111662	4,11193387	0,048013	5,1347983	0,0167744
109943193	LOC109943193	uncharacterized LOC109943193	0	0	0	0	0	0	0	0	3,3156203	0,011936
109943262	LOC109943262	uncharacterized LOC109943262	0	0	0	0	0	0	0	0	4,0448219	0,0476472
109943514	LOC109943514	uncharacterized LOC109943514	0	0	0	0	0	0	2,23821921	0,039639	0	0
109943652	LOC109943652	uncharacterized LOC109943652	0	0	0	0	-3,999186	0,0341755	-4,0213989	0,030785	-4,034969	0,0293273
109943679	LOC109943679	uncharacterized LOC109943679	0	0	0	0	-1,668096	0,0029282	-1,5179866	0,006711	0	0
109943730	LOC109943730	uncharacterized LOC109943730	0	0	0	0	-4,25505	0,0394407	0	0	0	0
109943740	LOC109943740	uncharacterized LOC109943740	0	0	0	0	3,5223145	0,0163164	0	0	0	0
109943773	LOC109943773	uncharacterized LOC109943773	0	0	-2,1191971	0,0110645	-1,612174	0,0439073	0	0	0	0
109944009	LOC109944009	uncharacterized LOC109944009	0	0	-5,3471807	2,055E-25	-6,274155	3,426E-32	-4,8291576	1,12E-19	0	0
109944010	LOC109944010	uncharacterized LOC109944010	0	0	0	0	3,3944894	0,0203248	3,70655366	0,009766	0	0
109944015	LOC109944015	uncharacterized LOC109944015	0	0	0	0	0	0	0	0	-4,098701	0,0476301
109944048	LOC109944048	uncharacterized LOC109944048	0	0	0	0	0	0	2,00866771	0,03721	0	0
109944084	LOC109944084	uncharacterized LOC109944084	0	0	0	0	0	0	0	0	4,265196	0,0494569
109944278	LOC109944278	uncharacterized LOC109944278	-2,012774	0,026653	0	0	0	0	0	0	0	0
109944416	LOC109944416	uncharacterized LOC109944416	0	0	0	0	-1,962922	0,0336413	-1,8325833	0,044785	0	0
109944790	LOC109944790	uncharacterized LOC109944790	0	0	-3,2447976	0,0031757	-2,675357	0,0098029	0	0	0	0
109944807	LOC109944807	uncharacterized LOC109944807	3,21295742	0,033286	4,42310615	0,0022167	4,4204799	0,0022109	0	0	0	0
109944859	LOC109944859	uncharacterized LOC109944859	0	0	0	0	0	0	5,20555131	0,047062	0	0
109944884	LOC109944884	uncharacterized LOC109944884	0	0	0	0	0	0	4,84663504	0,026089	5,0725646	0,0198803
109945043	LOC109945043	uncharacterized LOC109945043	0	0	-3,2439028	0,025829	0	0	0	0	0	0
109945301	LOC109945301	uncharacterized LOC109945301	-4,730336	0,006497	0	0	-3,15291	0,0327127	0	0	0	0
109945549	LOC109945549	uncharacterized LOC109945549	0	0	0	0	0	0	0	0	3,0735527	0,0244053
109945745	LOC109945745	uncharacterized LOC109945745	0	0	0	0	1,5367287	0,0437533	1,67933897	0,024518	0	0
109945802	LOC109945802	uncharacterized LOC109945802	-2,8296734	0,001818	-3,5353878	0,0001718	-3,428152	0,000228	0	0	0	0
109945979	LOC109945979	uncharacterized LOC109945979	0	0	0	0	4,4555896	0,0304813	0	0	0	0
109945989	LOC109945989	uncharacterized LOC109945989	0	0	0	0	0	0	0	0	4,0058059	0,015502
109946011	LOC109946011	uncharacterized LOC109946011	0	0	0	0	1,8831542	0,003855	0	0	0	0
109946015	LOC109946015	uncharacterized LOC109946015	0	0	0	0	0	0	0	0	2,7882975	0,0413637
109946021	LOC109946021	uncharacterized LOC109946021	0	0	0	0	0	0	-3,445713	0,012769	0	0
109946028	LOC109946028	uncharacterized LOC109946028	0	0	1,70916034	0,0332449	0	0	0	0	0	0
110354864	LOC110354864	uncharacterized LOC110354864	0	0	0	0	1,8075532	0,0348607	2,45598492	0,004824	2,2662215	0,0074738
111347722	LOC111347722	uncharacterized LOC111347722	0	0	0	0	0	0	2,22225792	0,018558	0	0

			Yuc x Ctr		Azo x Ctr		AzoYuc x Ctr		AzoYuc x Yuc		AzoYuc x Azo	
Gene ID	Gene Symbol	Gene Description	Log2(FC)	pvalue	Log2(FC)	pvalue	Log2(FC)	pvalue	Log2(FC)	pvalue	Log2(FC)	pvalue
111589286	LOC111589286	uncharacterized LOC111589286	0	0	0	0	0	0	3,54144582	0,009863	0	0
111589300	LOC111589300	uncharacterized LOC111589300	0	0	-4,0000655	0,0184701	-4,030412	0,0175998	0	0	0	0
111589342	LOC111589342	uncharacterized LOC111589342	-2,014159	0,010723	0	0	0	0	0	0	0	0
111589344	LOC111589344	uncharacterized LOC111589344	0	0	0	0	4,9882214	0,0189343	5,41095559	0,010906	0	0
111589557	LOC111589557	uncharacterized LOC111589557	0	0	0	0	3,4247195	0,008629	3,22970782	0,009257	0	0
111589728	LOC111589728	uncharacterized LOC111589728	0	0	0	0	1,53297	0,0075083	0	0	0	0
111589793	LOC111589793	uncharacterized LOC111589793	0	0	0	0	1,9568078	0,0220995	2,10697186	0,01332	1,9823283	0,0193548
111590133	LOC111590133	uncharacterized LOC111590133	0	0	0	0	0	0	3,89509966	0,025668	0	0
111590268	LOC111590268	uncharacterized LOC111590268	0	0	0	0	0	0	0	0	1,6784448	0,0202812
111590293	LOC111590293	uncharacterized LOC111590293	-4,6171341	1,74E-05	-4,8623876	6,307E-06	-4,344493	2,068E-05	0	0	0	0
111590552	LOC111590552	uncharacterized LOC111590552	0	0	0	0	0	0	2,46775966	0,048582	0	0
111590837	LOC111590837	uncharacterized LOC111590837	0	0	0	0	2,2849078	0,0023295	2,31189807	0,001728	1,7826504	0,0134623
111591126	LOC111591126	uncharacterized LOC111591126	0	0	0	0	0	0	4,07907627	0,017361	0	0
111591151	LOC111591151	uncharacterized LOC111591151	0	0	0	0	0	0	2,1462755	0,021022	0	0
541632	gpm91	uncharacterized LOC541632	0	0	0	0	0	0	0	0	1,9006744	0,026061
541875	TIDP2897	uncharacterized LOC541875	3,95360115	0,009751	3,56445841	0,0204205	4,1793693	0,0057341	0	0	0	0
542109	TIDP3321	uncharacterized LOC542109	0	0	0	0	4,6108438	9,988E-05	3,60654835	0,000771	3,7718585	0,0004273
778433	LOC778433	uncharacterized LOC778433	0	0	0	0	1,6072923	0,0351019	0	0	0	0
103639891	LOC103639891	uncharacterized protein	0	0	0	0	-1,706848	0,013249	0	0	0	0
103641455	LOC103641455	uncharacterized protein	0	0	0	0	1,5790487	0,0190851	0	0	0	0
103653829	LOC103653829	uncharacterized protein At1g65710	0	0	0	0	0	0	3,07078002	0,009381	3,2684104	0,0056919
103650845	LOC103650845	uncharacterized protein At4g15970	-2,9821828	0,039621	-3,6682735	0,0136454	-4,372389	0,0051177	0	0	0	0
103636147	LOC103636147	uncharacterized protein At4g18257	0	0	0	0	0	0	0	0	5,3841908	0,0450817
103639027	LOC103639027	uncharacterized protein C630.12	0	0	0	0	0	0	2,00800136	0,047625	0	0
103637556	LOC103637556	uncharacterized protein DDB_G0271670	0	0	0	0	2,8750221	0,0107349	0	0	2,3247516	0,0260386
100192831	LOC100192831	Uncharacterized protein family (UPF0114)	0	0	-2,1792153	0,0371554	0	0	0	0	0	0
109945706	LOC109945706	uncharacterized protein LOC109945706	-1,6337658	0,005618	0	0	0	0	2,1657402	0,000138	0	0
732735	gpm309	uncharacterized protein LOC732735	1,75228425	0,027003	0	0	0	0	0	0	0	0
103643652	LOC103643652	uncharacterized protein PFB0765w	4,13158428	0,003096	0	0	0	0	0	0	0	0
109939916	LOC109939916	uncharacterized vacuolar membrane protein YML018C	-4,1591204	0,019896	-3,5647931	0,0334513	0	0	0	0	0	0
103651003	LOC103651003	UPF0503 protein At3g09070, chloroplastic	0	0	2,42019207	0,0485446	0	0	0	0	0	0
103629941	LOC103629941	urea-proton symporter DUR3	0	0	0	0	2,1808372	0,0125621	0	0	0	0
100193891	LOC100193891	ustilago maydis induced8	-1,7592856	0,007824	0	0	0	0	0	0	0	0
100282817	LOC100282817	vacuolar ATP synthase 16 kDa proteolipid subunit	0	0	0	0	0	0	1,62886155	0,018377	0	0

			Yuc x Ctr		Azo x Ctr		AzoYuc x Ctr		AzoYuc x Yuc		AzoYuc x Azo	
Gene ID	Gene Symbol	Gene Description	Log2(FC)	pvalue	Log2(FC)	pvalue	Log2(FC)	pvalue	Log2(FC)	pvalue	Log2(FC)	pvalue
100276400	LOC100276400	Vacuolar ATPase assembly integral membrane protein VMA21-like domain	0	0	0	0	4,8743413	0,0013841	4,71958244	0,001096	4,970065	0,0006063
100383083	LOC100383083	Vacuolar iron transporter homolog 1	0	0	0	0	1,9665937	0,0104027	2,51545649	0,001147	1,8145958	0,0143903
100286140	pco079297	Vacuolar processing enzyme, beta-isozyme	1,96290091	0,037396	0	0	1,92868	0,0396004	0	0	0	0
103654298	LOC103654298	vacuolar protein sorting-associated protein 32 homolog 2-like	0	0	0	0	0	0	0	0	1,5117655	0,0384518
100304345	LOC100304345	Vacuolar protein sorting-associated protein 41-like protein	0	0	0	0	1,7282735	0,0112276	1,55378218	0,01859	0	0
100192923	LOC100192923	vacuolar protein sorting-associated protein 54, chloroplastic	0	0	0	0	0	0	1,5251767	0,003141	0	0
100272263	LOC100272263	vacuolar protein-sorting-associated protein 37 homolog 1	0	0	0	0	3,7301718	0,0493549	0	0	0	0
100192667	LOC100192667	Vacuolar-processing enzyme gamma-isozyme	0	0	0	0	4,4143265	9,02E-06	3,76296766	3,88E-05	3,104083	0,000419
100383090	LOC100383090	Vacuolar-sorting receptor 1	0	0	0	0	0	0	0	0	4,2743322	0,0470281
100383531	LOC100383531	Vacuolar-sorting receptor 1	0	0	1,68456049	0,0291413	0	0	0	0	0	0
100194398	pco116016	Valine--tRNA ligase chloroplastic/mitochondrial 2	-1,6548436	0,024943	0	0	0	0	0	0	0	0
103632992	LOC103632992	vegetative cell wall protein gp1	0	0	0	0	0	0	-3,8711351	0,005328	0	0
103639976	LOC103639976	vegetative cell wall protein gp1-like	-4,1824479	0,004632	-5,8900883	0,0005138	-5,920435	0,0004806	0	0	0	0
103640021	LOC103640021	vegetative cell wall protein gp1-like	0	0	0	0	0	0	0	0	-3,624607	0,0404009
103644774	LOC103644774	vegetative cell wall protein gp1-like	-1,876505	0,039977	0	0	0	0	0	0	0	0
103645391	LOC103645391	vegetative cell wall protein gp1-like	0	0	0	0	0	0	1,72573473	0,028357	0	0
109940438	LOC109940438	vegetative cell wall protein gp1-like	0	0	0	0	0	0	0	0	1,6140372	0,0318553
109941716	LOC109941716	vegetative cell wall protein gp1-like	0	0	-2,9251084	0,0496458	0	0	0	0	0	0
109941771	LOC109941771	vegetative cell wall protein gp1-like	0	0	0	0	0	0	2,82747972	0,03304	0	0
109941905	LOC109941905	vegetative cell wall protein gp1-like	0	0	0	0	1,9975192	0,0179138	0	0	0	0
109945974	LOC109945974	vegetative cell wall protein gp1-like	0	0	2,01360926	0,0366179	0	0	0	0	0	0
100284565	umc2754	Vegetative storage protein 2	-1,7072744	0,001136	0	0	0	0	0	0	0	0
100285462	LOC100285462	versicolorin reductase	0	0	0	0	1,5026858	0,0235463	0	0	0	0
103634953	LOC103634953	very-long-chain 3-oxoacyl-CoA reductase-like protein At1g24470	0	0	0	0	4,9192738	0,0239963	0	0	0	0
103633692	LOC103633692	very-long-chain enoyl-CoA reductase	4,91614494	0,000806	3,63062791	0,01562	3,6436035	0,0150978	0	0	0	0
100283494	LOC100283494	vesicle-associated membrane protein 714	0	0	0	0	1,8378727	0,0063241	0	0	0	0
100273789	LOC100273789	Villin-2	0	0	0	0	2,2621664	0,000941	2,1305223	0,001682	1,6742308	0,012954
103639123	LOC103639123	villin-2	0	0	0	0	0	0	3,39196574	0,04069	0	0
100280056	LOC100280056	VIN3-like protein 1	0	0	0	0	1,81191	0,0425617	0	0	0	0
103652991	LOC103652991	VIN3-like protein 2	0	0	0	0	-1,667583	0,0014613	-1,8791668	0,000276	0	0

			Yuc x Ctr		Azo x Ctr		AzoYuc x Ctr		AzoYuc x Yuc		AzoYuc x Azo	
Gene ID	Gene Symbol	Gene Description	Log2(FC)	pvalue	Log2(FC)	pvalue	Log2(FC)	pvalue	Log2(FC)	pvalue	Log2(FC)	pvalue
100381876	LOC100381876	violaxanthin de-epoxidase3	0	0	-2,4669799	0,0146627	-3,427785	0,0013647	0	0	0	0
542622	vdac1a	voltage-dependent anion channel protein1a	0	0	0	0	-1,665766	0,008374	0	0	0	0
100280493	LOC100280493	von Willebrand factor type A domain containing protein	0	0	0	0	0	0	0	0	1,5724721	0,0330647
100281794	LOC100281794	VQ motif family protein	0	0	0	0	0	0	0	0	2,8489039	0,041213
100274416	pco086632	V-type proton ATPase subunit C	0	0	0	0	0	0	1,93309618	0,007812	1,7960732	0,0122851
100281682	LOC100281682	WAK1 - OsWAK receptor-like cytoplasmic kinase (OsWAK-RLCK)	0	0	0	0	1,8777578	0,0215436	2,01866347	0,012739	2,406537	0,0031515
100281403	LOC100281403	WAK111 - OsWAK receptor-like protein kinase	0	0	1,51804696	0,0469103	0	0	0	0	0	0
100280667	LOC100280667	WAK80 - OsWAK receptor-like protein kinase	0	0	0	0	2,3056859	0,0033614	2,05503987	0,007526	3,3865528	2,503E-05
103648521	LOC103648521	wall-associated receptor kinase 1	-4,1245132	0,03598	-5,3122403	0,0069116	0	0	0	0	0	0
103653092	LOC103653092	wall-associated receptor kinase 1	0	0	0	0	0	0	-2,1283655	0,03367	0	0
103641702	LOC103641702	wall-associated receptor kinase 2	-5,2319284	0,006848	-5,4578613	0,0047884	0	0	0	0	0	0
100193004	LOC100193004	Wall-associated receptor kinase 3	0	0	0	0	2,1448878	0,0166935	1,82880297	0,033366	1,8705266	0,028444
103645132	LOC103645132	wall-associated receptor kinase 3	6,44340922	0,010662	0	0	0	0	-5,8316842	0,015305	0	0
103641905	LOC103641905	wall-associated receptor kinase 5	0	0	-5,3763971	0,0159528	-4,44495	0,0463198	0	0	0	0
100280685	pco142103	WAT1-related protein	0	0	0	0	1,5520162	0,0471855	1,91926264	0,013988	0	0
100272362	si605018e07b	WAT1-related protein	0	0	1,81071014	0,0260806	0	0	0	0	-1,581822	0,0459035
103627609	LOC103627609	WAT1-related protein At5g45370	0	0	3,61146272	0,029643	0	0	0	0	0	0
103639610	LOC103639610	WD repeat-containing protein 6	0	0	0	0	2,7716701	0,0095694	0	0	2,6028094	0,007976
109939830	LOC109939830	WEB family protein At1g75720	0	0	0	0	3,1438749	0,0332034	3,09039567	0,028333	0	0
103652813	LOC103652813	WIN1	-4,3218769	1,52E-06	-5,1174309	9,48E-08	-2,350707	0,002849	1,97116866	0,031861	2,7667204	0,0046458
100280811	LOC100280811	wound induced protein	0	0	0	0	0	0	-3,9310838	0,044205	0	0
542729	wip1	wound induced protein 1	0	0	0	0	-2,121813	0,0369249	0	0	0	0
103650495	LOC103650495	WPP domain-associated protein	0	0	0	0	2,1494842	0,029922	0	0	0	0
100279277	cl37545_1	WPP domain-interacting protein 2	0	0	1,64054708	0,033027	0	0	0	0	-1,901273	0,0120998
100216676	LOC100216676	WREBP-2 protein	0	0	0	0	2,2622953	0,0365044	0	0	0	0
100286379	LOC100286379	WRKY DNA binding domain containing protein	0	0	0	0	2,8579149	0,0074481	0	0	2,6375578	0,0082038
100285086	LOC100285086	WRKY transcription factor 4	-1,9415206	0,028357	0	0	0	0	0	0	0	0
103649526	LOC103649526	WRKY transcription factor 6	-2,130321	0,045416	0	0	0	0	0	0	0	0
100285621	LOC100285621	WRKY39v2 - superfamily of TFs having WRKY and zinc finger domains	0	0	0	0	-5,083396	0,0090899	0	0	0	0
100280956	LOC100280956	WW domain-binding protein 11	0	0	3,39109246	0,0245354	0	0	0	0	0	0
100284097	LOC100284097	xyloglucan endotransglucosylase/hydrolase protein 1	0	0	0	0	-4,205004	0,0193257	-4,3976158	0,012773	0	0

			Yuc x Ctr		Azo x Ctr		AzoYuc x Ctr		AzoYuc x Yuc		AzoYuc x Azo	
Gene ID	Gene Symbol	Gene Description	Log2(FC)	pvalue	Log2(FC)	pvalue	Log2(FC)	pvalue	Log2(FC)	pvalue	Log2(FC)	pvalue
103641948	LOC103641948	xyloglucan galactosyltransferase XLT2	3,4189367	0,045971	3,81921637	0,0235684	0	0	0	0	0	0
100286101	LOC100286101	xylosyltransferase 1	0	0	-1,5386504	0,0401886	0	0	1,77031018	0,015061	2,4199552	0,001039
100281681	LOC100281681	xylosyltransferase oxt	0	0	-6,0502275	0,0015141	0	0	0	0	0	0
100136882	y1	yellow endosperm 1	0	0	0	0	0	0	0	0	2,5007939	0,033954
100285703	LOC100285703	yfnA	0	0	0	0	1,7246562	0,0219267	0	0	0	0
103639823	LOC103639823	YLP motif-containing protein 1-like	-3,4509221	0,035361	0	0	0	0	0	0	0	0
103634612	LOC103634612	Yos1-like protein	0	0	0	0	2,0901984	0,0485385	0	0	2,7589308	0,0093573
100276706	pco129709	Yos1-like protein	0	0	3,23079621	0,0407773	3,2182415	0,0414045	0	0	0	0
542531	ypt1	ypt homolog 1	0	0	0	0	0	0	1,54404233	0,049951	1,719789	0,0288513
100127513	ZCN2	ZCN2 protein	3,68175952	0,017131	0	0	0	0	-3,8867729	0,008597	0	0
100192567	pco122535	zinc finger (C2H2 type) family protein	0	0	0	0	1,9054847	0,0223921	0	0	1,9613123	0,015488
100501523	LOC100501523	zinc finger (C3HC4-type RING finger) family protein	0	0	0	0	2,2713037	0,0178609	2,51527634	0,008455	3,013997	0,001741
103627653	LOC103627653	Zinc finger BED domain-containing protein DAYSLEEPER	0	0	0	0	-2,375615	0,0401158	0	0	0	0
103649890	LOC103649890	zinc finger BED domain-containing protein RICESLEEPER 2	0	0	0	0	0	0	0	0	2,4344651	0,0442859
109941203	LOC109941203	zinc finger BED domain-containing protein RICESLEEPER 2-like	0	0	0	0	0	0	0	0	4,0046597	0,0260899
109943709	LOC109943709	zinc finger BED domain-containing protein RICESLEEPER 2-like	0	0	0	0	0	0	0	0	1,8584312	0,0263899
103651410	LOC103651410	zinc finger CCCH domain-containing protein 13	0	0	0	0	2,1595517	0,0037524	0	0	0	0
100275303	LOC100275303	zinc finger CCCH domain-containing protein 15 homolog	-2,2988999	0,003744	0	0	0	0	0	0	0	0
103634911	LOC103634911	zinc finger CCCH domain-containing protein 2	0	0	2,23440113	0,0297216	0	0	0	0	0	0
103641439	LOC103641439	zinc finger CCCH domain-containing protein 24	0	0	0	0	2,6366859	0,0102297	1,9816011	0,037681	1,8555799	0,0482429
103634705	LOC103634705	zinc finger CCCH domain-containing protein 3	0	0	0	0	-4,287358	0,0214895	-3,8142244	0,041172	0	0
100273243	LOC100273243	Zinc finger CCCH domain-containing protein 33	0	0	0	0	0	0	1,5607631	0,032053	1,5699175	0,0305154
103653706	LOC103653706	zinc finger CCCH domain-containing protein 53-like	0	0	0	0	-3,869509	0,024161	0	0	0	0
103650585	LOC103650585	zinc finger CCCH domain-containing protein 67	0	0	0	0	2,5328165	0,0215522	4,33575115	0,000251	2,9775549	0,0066222
100284552	LOC100284552	zinc finger CCCH type domain-containing protein ZFN-like 1	1,68651393	0,044715	0	0	0	0	0	0	0	0

			Yuc x Ctr		Azo x Ctr		AzoYuc x Ctr		AzoYuc x Yuc		AzoYuc x Azo	
Gene ID	Gene Symbol	Gene Description	Log2(FC)	pvalue	Log2(FC)	pvalue	Log2(FC)	pvalue	Log2(FC)	pvalue	Log2(FC)	pvalue
100286049	LOC100286049	zinc finger C-x8-C-x5-C-x3-H type family protein	0	0	-2,3205728	0,0098937	-2,25795	0,011891	0	0	0	0
103646416	LOC103646416	zinc finger matrin-type protein 2-like	0	0	0	0	-4,530865	0,0458357	0	0	0	0
103653765	LOC103653765	zinc finger MYM-type protein 1-like	0	0	0	0	5,4164428	0,0037252	3,54822546	0,03632	0	0
103634372	LOC103634372	zinc finger protein 36	0	0	0	0	4,3860858	0,028125	0	0	4,0304154	0,0320782
109943107	LOC109943107	zinc finger protein CONSTANS	1,81809472	0,011872	0	0	0	0	-2,0441886	0,003865	0	0
103647917	LOC103647917	zinc finger protein CONSTANS-LIKE 15-like	0	0	0	0	5,1460161	0,0047303	5,5687518	0,002236	5,7946823	0,001468
103646270	LOC103646270	zinc finger protein CONSTANS-LIKE 3	3,4925122	0,005374	0	0	0	0	-2,5587082	0,03098	0	0
100216849	LOC100216849	zinc finger, C3HC4 type family protein	0	0	0	0	0	0	0	0	1,583348	0,0273936
100281133	LOC100281133	zinc finger, C3HC4 type family protein	3,94539953	0,010436	0	0	0	0	0	0	0	0
100192675	LOC100192675	zinc knuckle (CCHC-type) family protein	0	0	0	0	0	0	2,00555321	0,046246	2,0514257	0,0395122
100284121	LOC100284121	zinc transporter 4	0	0	0	0	2,5612029	0,0020531	2,22585407	0,006583	2,076719	0,010889
103625827	LOC103625827	zinc transporter ZTP29	0	0	0	0	0	0	-2,1787647	0,031839	0	0
100273932	LOC100273932	Zinc-finger domain of monoamine-oxidase A repressor R1	0	0	0	0	2,7099827	0,0481497	3,71157526	0,011206	2,6708261	0,0395531
103652837	LOC103652837	zinc-finger homeodomain protein 4	0	0	0	0	0	0	2,14631114	0,024653	2,0371069	0,0263885
100282128	sca1	*Zmaba2* (aka sca1 - short chain alcohol dehydrogenase 1)	1,86956273	0,00148	0	0	0	0	-1,28032	0,022586	0	0
103642400	LOC103642400	*Zmarf1 (Arf-transcription factor 1)*	0	0	0	0	1,5497897	0,0040418	0	0	0	0
100383226	ARF4	*Zmarf4 (Arf-transcription factor 4)*	2,85175926	0,047165	0	0	0	0	0	0	0	0
100857063	LOC100857063	*Zmarf7 (Arf-transcription factor 7)*	0	0	0	0	0	0	0	0	1,503674	0,0008362
103654892	ARF16	*Zmarf16 (Arf-transcription factor 16)*	-1,8752477	0,022374	0	0	0	0	0	0	0	0
100502480	LOC100502480	*Zmarf18 (Arf-transcription factor 18)*	0	0	0	0	0	0	0	0	1,5095444	0,0119078
100280136	LOC100280136	*Zmarf19 (Arf-transcription factor 19)*	-1,5769473	0,043389	0	0	0	0	0	0	0	0
103629639	LOC103629639	*Zmarf22 (Arf-transcription factor 22)*	0	0	0	0	1,7496819	0,0222355	0	0	0	0
103630727	LOC103630727	*Zmarf24 (Arf-transcription factor 24)*	0	0	-1,7782055	0,006104	-1,527487	0,016904	0	0	0	0
100273501	LOC100273501	*Zmarf25 (Arf-transcription factor 25)*	0	0	0	0	0	0	-1,6302519	0,001535	0	0
103627702	LOC103627702	*Zmdel1c* (member of E2F transcription factor family - DEL type)	0	0	0	0	2,9154732	0,0454705	2,76913372	0,049957	3,1922419	0,0254075
100193303	cl2682_1	*Zmgh3.10*	0	0	0	0	2,2741547	0,0050714	2,05531804	0,008753	1,5945156	0,0372075
100284457	LOC100284457	*Zmiaa10/rum1* - Aux/IAA-transcription factor 10/rum1 (rootless with undetectable meristems1)	0	0	0	0	0	0	2,00974033	0,029499	2,3064966	0,0130437
100194253	AUX22	*Zmiaa27/bif1* - Aux/IAA-transcription factor 27/bif1	0	0	3,14519261	0,0046839	0	0	0	0	-2,228641	0,0177913

			Yuc x Ctr		Azo x Ctr		AzoYuc x Ctr		AzoYuc x Yuc		AzoYuc x Azo	
Gene ID	Gene Symbol	Gene Description	Log2(FC)	pvalue	Log2(FC)	pvalue	Log2(FC)	pvalue	Log2(FC)	pvalue	Log2(FC)	pvalue
100274580	AUX18	*Zmiaa7* - Aux/IAA-transcription factor 7	0	0	0	0	1,932135	0,0438487	0	0	0	0
100193847	LOC100193847	*Zmick4* -Inhibitor of cyclin-dependent kinase	0	0	0	0	0	0	3,15901329	0,024966	2,7688997	0,0362389
109946070	LOC109946070	*Zmmkk5* (mitogen-activated protein kinase kinase 5)	3,51103099	0,002083	0	0	0	0	-2,2790099	0,017106	0	0
541618	LOC541618	*Zmmpk5* (mitogen-activated protein kinase 5)	1,95482855	0,007492	0	0	0	0	-2,3713411	0,001083	0	0
103654258	LOC103654258	*Zmpin1c* (PIN-formed protein 1c)	0	0	0	0	1,801325	0,0337059	1,56229126	0,049793	0	0
100285745	cl464_-1	*Zmpin1d* (PIN-formed protein 1d)	0	0	0	0	0	0	3,15943589	0,031069	0	0
100192073	PP2C14	*Zmpp2c14* (protein phosphatase 2C 14)	0	0	2,11087176	0,0259002	0	0	0	0	-2,138141	0,0173425
100282657	LOC100282657	*Zmpp2c3* (protein phosphatase 2C 3)	0	0	1,62209061	0,0367907	0	0	0	0	-1,734429	0,0184994
100381549	PP2C4	*Zmpp2c4* (protein phosphatase 2C 4)	0	0	2,33038937	0,0074928	0	0	0	0	-1,711966	0,0362707
103634514	LOC103634514	*Zmpyl7*	2,19026959	0,004209	0	0	2,3862512	0,0017719	0	0	3,3389902	1,63E-05
103653849	LOC103653849	*Zmsaur41* -auxin-responsive SAUR family member	0	0	4,8360325	0,0070572	0	0	0	0	0	0
100277080	LOC100277080	*Zmtlc17* (TRAM/LAG/CRN8 17)	4,64095739	0,00045	0	0	0	0	-3,1368981	0,004368	0	0
100274425	LOC100274425	*Zmtlc9* (TRAM/LAG/CRN8 9)	-17,480033	5,21E-05	0	0	0	0	20,0287248	2,43E-06	0	0

	Yuc x Ctr	Azo x Ctr	AzoYuc x Ctr	AzoYuc x Yuc	AzoYuc x Azo
Dow regulated loci	463	429	501	271	219
up regulated loci	272	221	716	698	684
Total DEG	735	650	1217	969	903
