# Supplementary material for: Effects on gene expression during maize-Azospirillum interaction in the presence of a plant-specific inhibitor of indole-3-acetic acid production
Source: Genet Mol Biol. 2023 Sep 18;46(3 Suppl 1):e20230100. doi: 10.1590/1678-4685-GMB-2023-0100 (PMC10510588; doi:10.1590/1678-4685-GMB-2023-0100)
Supplement: Table S3 - [file 1415-4757-GMB-46-3-s1-e20230100-s5.pdf]

Genetics and Molecular Biology

https://doi.org/10.1590/1678-4685-GMB-2023-0100

Supplementary Material to "Effects on gene expression during maize-*Azospirillum* interaction in the presence of a plant-specific inhibitor of indole-3-acetic acid production"

Table S3 - Maize uncharacterized differentially expressed genes (UDEGs) in all experimental conditions. Genes that presented |Log2(FC) ≥ 1.5| and p-value ≤ 0.05 were considered as differentially expressed. Gene descriptions were obtained using the MaizeMine databank at https://maizemine.rnet.missouri.edu/maizemine/begin.do. Ctr = control plantlets; Yuc = plantlets that received 50 µM of yucasin; Azo = plantlets inoculated with *A. brasilense* FP2; AzoYuc = plantlets that received 50 µM of yucasin and were inoculated with *A. brasilense* FP2.

			Yuc x Ctr		Azo x Ctr		AzoYuc x Ctr		AzoYuc x Yuc		AzoYuc x Azo	
**Gene ID**	**Gene Symbol**	**Gene Description**	**Log2(FC)**	**pvalue**	**Log2(FC)**	**pvalue**	**Log2(FC)**	**pvalue**	**Log2(FC)**	**pvalue**	**Log2(FC)**	**pvalue**
541632	gpm91	uncharacterized LOC541632	0	0	0	0	0	0	0	0	1.9006744	0.02606097
541875	TIDP2897	uncharacterized LOC541875	3.95360115	0.009751	3.5644584	0.02042	4.1793693	0.005734	0	0	0	0
542109	TIDP3321	uncharacterized LOC542109	0	0	0	0	4.6108438	9.99E-05	3.606548	0.0007709	3.7718585	0.00042726
778433	LOC778433	uncharacterized LOC778433	0	0	0	0	1.6072923	0.035102	0	0	0	0
100037785	gpm255	uncharacterized LOC100037785	0	0	0	0	0	0	0	0	-4.052054	0.02718214
100101546	TIDP3215	uncharacterized LOC100101546	0	0	0	0	0	0	-1.884059	0.0403483	0	0
100125658	umc1760	uncharacterized LOC100125658	2.7192843	0.006738	0	0	0	0	-1.928599	0.0432361	0	0
100170237	pco064225	uncharacterized LOC100170237	2.50341273	0.041543	3.023325	0.01266	0	0	0	0	0	0

			Yuc x Ctr		Azo x Ctr		AzoYuc x Ctr		AzoYuc x Yuc		AzoYuc x Azo	
**Gene ID**	**Gene Symbol**	**Gene Description**	**Log2(FC)**	**pvalue**	**Log2(FC)**	**pvalue**	**Log2(FC)**	**pvalue**	**Log2(FC)**	**pvalue**	**Log2(FC)**	**pvalue**
100191163	LOC100191163	uncharacterized LOC100191163	0	0	0	0	3.468039	0.000417	3.268828	0.0006406	3.4270449	0.00033919
100191176	LOC100191176	uncharacterized LOC100191176	3.68398079	0.02731	3.3673094	0.04432	3.5673472	0.031966	0	0	0	0
100191184	AI665898	uncharacterized LOC100191184	0	0	0	0	0	0	-1.823152	0.0466924	-2.715447	0.00237946
100191196	umc1979	uncharacterized LOC100191196	0	0	0	0	4.3420505	0.039992	0	0	0	0
100191198	si606063g10	uncharacterized LOC100191198	0	0	0	0	0	0	1.504348	0.0284566	1.6168028	0.01796155
100191203	pco137008b	uncharacterized LOC100191203	2.49947534	0.00709	0	0	0	0	-1.961502	0.0266081	0	0
100191221	LOC100191221	uncharacterized LOC100191221	0	0	0	0	1.8069459	0.049415	3.107972	0.0020941	0	0
100191249	LOC100191249	uncharacterized LOC100191249	0	0	0	0	0	0	0	0	-4.211053	0.0110796
100191422	LOC100191422	uncharacterized LOC100191422	0	0	3.6846433	0.00486	0	0	0	0	-2.693634	0.02161048
100191433	LOC100191433	uncharacterized LOC100191433	0	0	0	0	0	0	0	0	3.300904	0.04604435
100191451	pco120288	uncharacterized LOC100191451	0	0	0	0	0	0	3.921783	0.0412315	0	0
100191575	LOC100191575	uncharacterized LOC100191575	0	0	0	0	-1.7718707	0.045938	0	0	0	0
100191599	LOC100191599	uncharacterized LOC100191599	0	0	0	0	1.6322447	0.027166	0	0	0	0

			Yuc x Ctr		Azo x Ctr		AzoYuc x Ctr		AzoYuc x Yuc		AzoYuc x Azo	
**Gene ID**	**Gene Symbol**	**Gene Description**	**Log2(FC)**	**pvalue**	**Log2(FC)**	**pvalue**	**Log2(FC)**	**pvalue**	**Log2(FC)**	**pvalue**	**Log2(FC)**	**pvalue**
100191709	LOC100191709	uncharacterized LOC100191709	0	0	3.5106759	0.04022	0	0	0	0	0	0
100191723	LOC100191723	uncharacterized LOC100191723	0	0	0	0	0	0	0	0	-1.912097	0.04629469
100191836	pco081433(10)	uncharacterized LOC100191836	0	0	0	0	-3.0045342	0.013262	0	0	-3.052576	0.0110337
100191866	LOC100191866	uncharacterized LOC100191866	0	0	-1.693127	0.04567	0	0	0	0	1.8503812	0.02431229
100191932	cl14781_1	uncharacterized LOC100191932	-1.5122202	0.009985	0	0	-1.5972522	0.005831	0	0	0	0
100192117	IDP2565	uncharacterized LOC100192117	0	0	-1.65903	0.0205	0	0	0	0	0	0
100192499	LOC100192499	uncharacterized LOC100192499	-3.2362563	0.002311	-3.982225	0.00023	-4.4510926	5.05E-05	0	0	0	0
100192503	IDP753	uncharacterized LOC100192503	0	0	0	0	3.8204315	0.001864	2.927562	0.0107752	3.6192405	0.00199086
100192543	LOC100192543	uncharacterized LOC100192543	0	0	0	0	2.5635094	0.004587	1.880107	0.0254162	2.1055639	0.01241218
100192551	LOC100192551	uncharacterized LOC100192551	-1.5807326	0.005116	0	0	0	0	0	0	0	0
100192676	LOC100192676	uncharacterized LOC100192676	0	0	-1.559434	0.04682	-1.7480141	0.026886	0	0	0	0
100192679	TIDP3001	uncharacterized LOC100192679	0	0	0	0	-1.8941803	0.000902	0	0	0	0
100192738	LOC100192738	uncharacterized LOC100192738	0	0	-1.794605	0.04634	0	0	0	0	0	0

			Yuc x Ctr		Azo x Ctr		AzoYuc x Ctr		AzoYuc x Yuc		AzoYuc x Azo	
**Gene ID**	**Gene Symbol**	**Gene Description**	**Log2(FC)**	**pvalue**	**Log2(FC)**	**pvalue**	**Log2(FC)**	**pvalue**	**Log2(FC)**	**pvalue**	**Log2(FC)**	**pvalue**
100192786	pco111209	uncharacterized LOC100192786	0	0	0	0	1.9175734	0.04209	0	0	0	0
100192807	LOC100192807	uncharacterized LOC100192807	0	0	3.9297476	0.00868	4.0269142	0.007053	5.411439	0.0002938	0	0
100192831	LOC100192831	Uncharacterized protein family (UPF0114)	0	0	-2.179215	0.03716	0	0	0	0	0	0
100192834	LOC100192834	uncharacterized LOC100192834	0	0	0	0	2.3423419	0.035727	0	0	0	0
100192870	LOC100192870	uncharacterized LOC100192870	0	0	0	0	0	0	0	0	-4.21134	0.02246001
100193013	LOC100193013	uncharacterized LOC100193013	2.71787179	0.003629	0	0	0	0	-2.589733	0.0040429	0	0
100193067	LOC100193067	uncharacterized LOC100193067	0	0	0	0	3.9666277	0.027884	0	0	0	0
100193119	pco130460	uncharacterized LOC100193119	1.81096506	0.013745	0	0	0	0	-1.883272	0.0095037	0	0
100193206	LOC100193206	uncharacterized LOC100193206	0	0	0	0	3.3856872	0.049267	0	0	0	0
100193252	LOC100193252	uncharacterized LOC100193252	0	0	0	0	0	0	0	0	5.0217186	0.00941764
100193350	LOC100193350	uncharacterized LOC100193350	3.36090621	0.034486	0	0	0	0	0	0	0	0
100193369	LOC100193369	uncharacterized LOC100193369	-3.2626575	0.003778	-2.4468	0.02059	0	0	0	0	0	0
100193385	LOC100193385	uncharacterized LOC100193385	0	0	0	0	0	0	0	0	1.9384452	0.04057256

			Yuc x Ctr		Azo x Ctr		AzoYuc x Ctr		AzoYuc x Yuc		AzoYuc x Azo	
**Gene ID**	**Gene Symbol**	**Gene Description**	**Log2(FC)**	**pvalue**	**Log2(FC)**	**pvalue**	**Log2(FC)**	**pvalue**	**Log2(FC)**	**pvalue**	**Log2(FC)**	**pvalue**
100193490	LOC100193490	uncharacterized LOC100193490	0	0	0	0	0	0	-3.466452	0.0017626	-3.479098	0.00161879
100193544	LOC100193544	uncharacterized LOC100193544	-3.2841419	0.000386	-2.644129	0.00301	-3.5234986	0.000138	0	0	0	0
100193572	LOC100193572	uncharacterized LOC100193572	0	0	0	0	0	0	-2.899678	0.0317786	0	0
100193630	LOC100193630	uncharacterized LOC100193630	0	0	0	0	4.5471526	0.029983	0	0	0	0
100193631	pco148690(146)	uncharacterized LOC100193631	0	0	0	0	0	0	0	0	1.6607767	0.02295329
100193643	LOC100193643	uncharacterized LOC100193643	2.21026145	0.019306	0	0	0	0	-2.478156	0.0080093	0	0
100193686	LOC100193686	uncharacterized LOC100193686	0	0	0	0	1.8819576	0.015207	0	0	1.6336442	0.02515668
100193708	LOC100193708	uncharacterized LOC100193708	0	0	-2.707981	0.0438	0	0	0	0	0	0
100193792	LOC100193792	uncharacterized LOC100193792	0	0	0	0	-2.0135201	0.048073	0	0	0	0
100193832	LOC100193832	uncharacterized LOC100193832	0	0	0	0	3.1259235	0.011079	4.404868	0.0006755	4.4084008	0.00055528
100193845	LOC100193845	uncharacterized LOC100193845	-1.5442768	0.036804	0	0	0	0	0	0	0	0
100193903	LOC100193903	uncharacterized LOC100193903	0	0	0	0	-2.3090423	0.025534	0	0	0	0
100193962	LOC100193962	uncharacterized LOC100193962	2.3355842	0.01388	0	0	0	0	0	0	0	0

			Yuc x Ctr		Azo x Ctr		AzoYuc x Ctr		AzoYuc x Yuc		AzoYuc x Azo	
**Gene ID**	**Gene Symbol**	**Gene Description**	**Log2(FC)**	**pvalue**	**Log2(FC)**	**pvalue**	**Log2(FC)**	**pvalue**	**Log2(FC)**	**pvalue**	**Log2(FC)**	**pvalue**
100193967	LOC100193967	uncharacterized LOC100193967	1.89503893	0.027866	0	0	0	0	0	0	0	0
100194035	LOC100194035	uncharacterized LOC100194035	0	0	0	0	0	0	0	0	-1.775621	0.0276304
100194075	IDP2402	uncharacterized LOC100194075	0	0	0	0	3.0544123	0.008265	3.008679	0.0079274	3.4774241	0.0024095
100194144	LOC100194144	uncharacterized LOC100194144	0	0	0	0	0	0	-4.104826	0.0303815	0	0
100194194	LOC100194194	uncharacterized LOC100194194	0	0	0	0	1.8715262	0.01198	1.529684	0.0351427	1.6092074	0.02626008
100194233	LOC100194233	uncharacterized LOC100194233	0	0	0	0	0	0	0	0	-3.260815	0.04278462
100194263	LOC100194263	uncharacterized LOC100194263	0	0	0	0	0	0	0	0	3.9474362	0.04032545
100194267	LOC100194267	uncharacterized LOC100194267	-2.1561859	0.049756	-2.405214	0.02897	-2.6432949	0.017351	0	0	0	0
100194317	LOC100194317	uncharacterized LOC100194317	-4.4161215	0.007306	-5.626793	0.00145	-3.3371883	0.022075	0	0	0	0
100194322	LOC100194322	uncharacterized LOC100194322	0	0	0	0	1.6938499	0.048844	0	0	0	0
100194342	pco083336	uncharacterized LOC100194342	0	0	0	0	0	0	-2.210018	0.0482786	0	0
100216602	LOC100216602	uncharacterized LOC100216602	0	0	0	0	1.567423	0.026788	0	0	0	0
100216613	LOC100216613	uncharacterized LOC100216613	3.45350896	0.023267	0	0	0	0	0	0	0	0

			Yuc x Ctr		Azo x Ctr		AzoYuc x Ctr		AzoYuc x Yuc		AzoYuc x Azo	
**Gene ID**	**Gene Symbol**	**Gene Description**	**Log2(FC)**	**pvalue**	**Log2(FC)**	**pvalue**	**Log2(FC)**	**pvalue**	**Log2(FC)**	**pvalue**	**Log2(FC)**	**pvalue**
100216658	LOC100216658	Uncharacterized conserved protein UCP009193	0	0	0	0	0	0	1.954297	0.0226673	0	0
100216691	cl5021_1	uncharacterized LOC100216691	0	0	0	0	1.7776274	0.001721	0	0	0	0
100216693	pco137385	uncharacterized LOC100216693	0	0	0	0	0	0	-1.512945	0.0363798	0	0
100216724	cl426_1a	uncharacterized LOC100216724	0	0	0	0	-1.9993611	0.037091	-2.017959	0.0325668	0	0
100216752	cl56262_1	uncharacterized LOC100216752	2.69903241	0.014882	0	0	0	0	0	0	0	0
100216762	LOC100216762	uncharacterized LOC100216762	0	0	0	0	-1.7703138	0.046081	0	0	0	0
100216854	LOC100216854	uncharacterized LOC100216854	-4.1593705	0.01751	0	0	0	0	0	0	0	0
100216879	LOC100216879	uncharacterized LOC100216879	0	0	0	0	0	0	3.161587	0.0188387	0	0
100216964	LOC100216964	uncharacterized LOC100216964	0	0	0	0	3.3140136	0.008405	2.430282	0.0313642	2.1685821	0.04936376
100216966	LOC100216966	uncharacterized LOC100216966	0	0	0	0	-1.8851315	0.031874	0	0	0	0
100217030	pco131458	uncharacterized LOC100217030	-2.1296474	0.048565	-2.533312	0.02067	0	0	0	0	0	0
100217059	LOC100217059	uncharacterized LOC100217059	0	0	0	0	-1.7328925	0.026267	0	0	0	0
100217087	LOC100217087	uncharacterized LOC100217087	0	0	0	0	4.4342441	0.003356	2.953998	0.0189557	2.7403196	0.02626847

			Yuc x Ctr		Azo x Ctr		AzoYuc x Ctr		AzoYuc x Yuc		AzoYuc x Azo	
**Gene ID**	**Gene Symbol**	**Gene Description**	**Log2(FC)**	**pvalue**	**Log2(FC)**	**pvalue**	**Log2(FC)**	**pvalue**	**Log2(FC)**	**pvalue**	**Log2(FC)**	**pvalue**
100217101	LOC100217101	uncharacterized LOC100217101	0	0	-2.166667	0.02867	0	0	0	0	0	0
100217106	LOC100217106	uncharacterized LOC100217106	0	0	0	0	2.3739915	0.037315	2.777258	0.0147552	0	0
100217114	TIDP2761	uncharacterized LOC100217114	0	0	0	0	-1.5176224	0.037379	0	0	0	0
100217130	LOC100217130	uncharacterized LOC100217130	0	0	0	0	0	0	-2.296395	0.0094787	-2.823149	0.00116514
100217178	LOC100217178	uncharacterized LOC100217178	-2.7567802	0.021217	-2.434562	0.03641	-3.6801377	0.003426	0	0	0	0
100217254	LOC100217254	uncharacterized LOC100217254	0	0	0	0	1.6100673	0.046312	0	0	0	0
100217290	LOC100217290	uncharacterized LOC100217290	0	0	0	0	0	0	0	0	4.3539808	0.02408099
100217299	LOC100217299	uncharacterized LOC100217299	0	0	2.884877	0.01918	0	0	0	0	-2.960909	0.0138803
100217309	LOC100217309	uncharacterized LOC100217309	4.06483679	0.007512	3.5646034	0.02003	3.8358883	0.011595	0	0	0	0
100272290	LOC100272290	uncharacterized LOC100272290	0	0	2.9768916	0.02028	2.7895219	0.030043	0	0	0	0
100272298	LOC100272298	uncharacterized LOC100272298	0	0	0	0	3.4856066	0.014312	6.103425	0.0004143	3.0540284	0.02311825
100272343	uaz277(201)	uncharacterized LOC100272343	0	0	0	0	2.7392193	0.045607	0	0	0	0
100272353	gpm443	uncharacterized LOC100272353	0	0	0	0	4.6070165	0.000617	4.145331	0.0006978	3.1577008	0.00532842

			Yuc x Ctr		Azo x Ctr		AzoYuc x Ctr		AzoYuc x Yuc		AzoYuc x Azo	
**Gene ID**	**Gene Symbol**	**Gene Description**	**Log2(FC)**	**pvalue**	**Log2(FC)**	**pvalue**	**Log2(FC)**	**pvalue**	**Log2(FC)**	**pvalue**	**Log2(FC)**	**pvalue**
100272427	LOC100272427	uncharacterized LOC100272427	0	0	0	0	-1.5757889	0.019945	0	0	0	0
100272433	LOC100272433	uncharacterized LOC100272433	0	0	0	0	0	0	0	0	4.1988521	0.02441304
100272453	pco133450	uncharacterized LOC100272453	4.13294714	0.028448	0	0	0	0	0	0	0	0
100272543	LOC100272543	uncharacterized LOC100272543	0	0	-3.716826	0.04356	-3.7471725	0.041871	0	0	0	0
100272571	LOC100272571	uncharacterized LOC100272571	-3.5027714	0.005922	-3.185839	0.00949	0	0	0	0	0	0
100272634	LOC100272634	uncharacterized LOC100272634	0	0	0	0	0	0	0	0	3.2981719	0.0344641
100272671	LOC100272671	uncharacterized LOC100272671	0	0	0	0	1.5718433	0.035812	0	0	0	0
100272733	LOC100272733	uncharacterized LOC100272733	0	0	0	0	0	0	0	0	3.8877232	0.03179363
100272755	CL11475_1	uncharacterized LOC100272755	0	0	0	0	2.0733205	0.018778	1.801895	0.0340411	0	0
100273040	LOC100273040	uncharacterized LOC100273040	0	0	0	0	3.3854612	0.048689	0	0	0	0
100273080	LOC100273080	uncharacterized LOC100273080	-2.5280016	0.013665	-2.813406	0.00623	-2.1694881	0.031123	0	0	0	0
100273158	LOC100273158	uncharacterized LOC100273158	-1.809192	0.021069	-1.585049	0.04176	-1.975159	0.011644	0	0	0	0
100273339	LOC100273339	uncharacterized LOC100273339	0	0	1.599086	0.0344	0	0	0	0	-1.552559	0.03575157

			Yuc x Ctr		Azo x Ctr		AzoYuc x Ctr		AzoYuc x Yuc		AzoYuc x Azo	
**Gene ID**	**Gene Symbol**	**Gene Description**	**Log2(FC)**	**pvalue**	**Log2(FC)**	**pvalue**	**Log2(FC)**	**pvalue**	**Log2(FC)**	**pvalue**	**Log2(FC)**	**pvalue**
100273371	LOC100273371	uncharacterized LOC100273371	0	0	2.9256142	0.02161	0	0	0	0	-3.833346	0.0034186
100273476	pco087970b	uncharacterized LOC100273476	1.60205166	0.022392	0	0	0	0	0	0	0	0
100273501	LOC100273501	uncharacterized LOC100273501	0	0	0	0	0	0	-1.630252	0.0015351	0	0
100273577	TIDP3460	uncharacterized LOC100273577	0	0	3.078084	0.04895	0	0	0	0	0	0
100273700	pco073762	uncharacterized LOC100273700	0	0	0	0	0	0	0	0	3.4442462	0.02246655
100273721	LOC100273721	uncharacterized LOC100273721	-2.0566228	0.034778	0	0	0	0	0	0	0	0
100273729	cl7341_1a	uncharacterized LOC100273729	0	0	0	0	-2.0841944	0.022798	-2.272996	0.0114252	0	0
100273730	LOC100273730	uncharacterized LOC100273730	-2.2098681	0.007489	-2.439612	0.00318	-2.8253965	0.000694	0	0	0	0
100273734	LOC100273734	uncharacterized LOC100273734	0	0	0	0	0	0	1.908113	0.0406582	0	0
100273811	LOC100273811	uncharacterized LOC100273811	0	0	0	0	0	0	2.490364	0.046899	2.4909702	0.04539177
100273889	LOC100273889	uncharacterized LOC100273889	0	0	0	0	2.1754539	0.005325	1.506181	0.0444687	1.8989959	0.01175586
100273916	LOC100273916	uncharacterized LOC100273916	0	0	0	0	3.2065506	0.025842	0	0	0	0
100274052	pco079415(520)	uncharacterized LOC100274052	0	0	0	0	0	0	0	0	3.8752543	0.03081857

			Yuc x Ctr		Azo x Ctr		AzoYuc x Ctr		AzoYuc x Yuc		AzoYuc x Azo	
**Gene ID**	**Gene Symbol**	**Gene Description**	**Log2(FC)**	**pvalue**	**Log2(FC)**	**pvalue**	**Log2(FC)**	**pvalue**	**Log2(FC)**	**pvalue**	**Log2(FC)**	**pvalue**
100274066	LOC100274066	uncharacterized LOC100274066	-2.5286973	0.024807	-3.299193	0.00521	-4.1003746	0.001531	0	0	0	0
100274126	LOC100274126	uncharacterized LOC100274126	0	0	0	0	0	0	1.597054	0.0285065	0	0
100274143	LOC100274143	uncharacterized LOC100274143	0	0	0	0	-2.8408813	0.033876	-3.117103	0.0170252	-2.906416	0.0260864
100274175	LOC100274175	uncharacterized LOC100274175	0	0	0	0	3.3120495	0.035049	3.194213	0.0302309	0	0
100274247	LOC100274247	uncharacterized LOC100274247	0	0	0	0	0	0	0	0	4.5165776	0.01134117
100274327	pco077290a	uncharacterized LOC100274327	0	0	0	0	0	0	2.600152	0.0363882	0	0
100274346	LOC100274346	uncharacterized LOC100274346	0	0	0	0	-1.5836538	0.032394	0	0	0	0
100274388	pco128421	uncharacterized LOC100274388	1.51307248	0.010004	0	0	0	0	0	0	0	0
100274417	LOC100274417	uncharacterized LOC100274417	0	0	0	0	3.7606412	0.020953	0	0	0	0
100274428	LOC100274428	uncharacterized LOC100274428	0	0	0	0	1.5971232	0.031001	0	0	0	0
100274481	LOC100274481	uncharacterized LOC100274481	0	0	0	0	1.8548853	0.038747	2.704151	0.0032991	0	0
100274493	cl4945_1	uncharacterized LOC100274493	0	0	1.9799276	0.0288	0	0	0	0	0	0
100274547	LOC100274547	uncharacterized LOC100274547	0	0	0	0	0	0	0	0	3.213486	0.03273595

			Yuc x Ctr		Azo x Ctr		AzoYuc x Ctr		AzoYuc x Yuc		AzoYuc x Azo	
**Gene ID**	**Gene Symbol**	**Gene Description**	**Log2(FC)**	**pvalue**	**Log2(FC)**	**pvalue**	**Log2(FC)**	**pvalue**	**Log2(FC)**	**pvalue**	**Log2(FC)**	**pvalue**
100274555	LOC100274555	uncharacterized LOC100274555	0	0	-3.869411	0.03341	0	0	0	0	3.7798918	0.0339059
100274655	LOC100274655	uncharacterized LOC100274655	0	0	0	0	0	0	0	0	4.7697462	0.03503288
100274722	LOC100274722	uncharacterized LOC100274722	0	0	-1.753035	0.04566	0	0	0	0	0	0
100274819	LOC100274819	uncharacterized LOC100274819	0	0	0	0	2.248102	0.039223	4.550557	0.0005417	2.7752012	0.01049194
100274850	IDP2453	uncharacterized LOC100274850	0	0	0	0	-3.0039414	0.000312	-3.263637	8.664E-05	-2.664582	0.00137072
100274866	LOC100274866	uncharacterized LOC100274866	1.65231536	0.011085	0	0	1.6629872	0.009986	0	0	0	0
100274896	LOC100274896	uncharacterized LOC100274896	0	0	0	0	0	0	1.959213	0.0128956	1.7405333	0.02548238
100274912	LOC100274912	uncharacterized LOC100274912	0	0	0	0	0	0	5.064573	0.017726	4.3287219	0.04268709
100274944	LOC100274944	uncharacterized LOC100274944	0	0	0	0	4.2950513	0.025375	4.717786	0.0140618	0	0
100274990	LOC100274990	uncharacterized LOC100274990	0	0	0	0	-1.5180652	0.009625	0	0	0	0
100274993	LOC100274993	uncharacterized LOC100274993	-4.047178	0.028792	0	0	0	0	0	0	0	0
100275001	LOC100275001	uncharacterized LOC100275001	0	0	0	0	0	0	5.526434	0.0456629	0	0
100275057	LOC100275057	uncharacterized LOC100275057	-2.9114086	0.002326	-2.512473	0.0054	-2.0837476	0.017169	0	0	0	0

			Yuc x Ctr		Azo x Ctr		AzoYuc x Ctr		AzoYuc x Yuc		AzoYuc x Azo	
**Gene ID**	**Gene Symbol**	**Gene Description**	**Log2(FC)**	**pvalue**	**Log2(FC)**	**pvalue**	**Log2(FC)**	**pvalue**	**Log2(FC)**	**pvalue**	**Log2(FC)**	**pvalue**
100275125	LOC100275125	uncharacterized LOC100275125	0	0	0	0	1.8136456	0.048973	0	0	0	0
100275129	LOC100275129	uncharacterized LOC100275129	0	0	-3.511109	0.00824	-2.8541747	0.025372	0	0	0	0
100275204	TIDP3241	uncharacterized LOC100275204	-1.6114537	0.037318	-1.797108	0.01964	0	0	0	0	0	0
100275297	LOC100275297	uncharacterized LOC100275297	-1.6683333	0.029106	0	0	0	0	0	0	0	0
100275309	pco110563	uncharacterized LOC100275309	0	0	0	0	0	0	0	0	-5.058115	0.0033463
100275318	LOC100275318	uncharacterized LOC100275318	0	0	2.3045914	0.02709	0	0	0	0	0	0
100275374	LOC100275374	uncharacterized LOC100275374	0	0	0	0	4.2752647	0.035232	4.697999	0.0206739	0	0
100275449	LOC100275449	uncharacterized LOC100275449	-3.5412097	4.33E-05	-2.966526	0.00052	-3.1695714	0.000216	0	0	0	0
100275463	cl5155_1	uncharacterized LOC100275463	0	0	0	0	2.6059817	0.001354	2.021044	0.0075565	1.9427769	0.00922464
100275495	LOC100275495	uncharacterized LOC100275495	-5.5765119	0.0013	-4.84065	0.00524	0	0	3.940339	0.0254286	0	0
100275503	LOC100275503	uncharacterized LOC100275503	0	0	0	0	2.6447994	0.027518	0	0	0	0
100275527	LOC100275527	uncharacterized LOC100275527	3.45375998	0.004183	0	0	0	0	-4.992062	0.0001116	0	0
100275576	LOC100275576	uncharacterized LOC100275576	0	0	0	0	4.0209283	0.026638	0	0	0	0

			Yuc x Ctr		Azo x Ctr		AzoYuc x Ctr		AzoYuc x Yuc		AzoYuc x Azo	
**Gene ID**	**Gene Symbol**	**Gene Description**	**Log2(FC)**	**pvalue**	**Log2(FC)**	**pvalue**	**Log2(FC)**	**pvalue**	**Log2(FC)**	**pvalue**	**Log2(FC)**	**pvalue**
100275588	LOC100275588	uncharacterized LOC100275588	0	0	2.9561921	0.00263	0	0	0	0	-3.579267	0.00026518
100275671	LOC100275671	uncharacterized LOC100275671	0	0	-3.584823	0.01218	0	0	0	0	0	0
100275705	LOC100275705	uncharacterized LOC100275705	0	0	0	0	0	0	3.839435	0.0031224	3.2522487	0.00779486
100275748	LOC100275748	uncharacterized LOC100275748	0	0	0	0	2.8273106	0.047957	0	0	0	0
100275772	LOC100275772	uncharacterized LOC100275772	0	0	0	0	0	0	-4.054103	0.0495173	0	0
100275797	LOC100275797	uncharacterized LOC100275797	-5.1922343	0.021392	0	0	0	0	0	0	0	0
100275804	LOC100275804	uncharacterized LOC100275804	-2.3340838	0.013385	0	0	0	0	0	0	0	0
100275868	LOC100275868	uncharacterized LOC100275868	0	0	2.3579079	0.00387	0	0	0	0	-2.368108	0.00172086
100275894	LOC100275894	uncharacterized LOC100275894	-3.6607237	0.006376	0	0	-3.817477	0.004265	0	0	0	0
100275960	IDP2353	uncharacterized LOC100275960	0	0	-2.694464	0.02757	0	0	0	0	0	0
100276011	cl16961_1(9)	uncharacterized LOC100276011	0	0	-1.965994	0.01924	0	0	0	0	0	0
100276016	LOC100276016	uncharacterized LOC100276016	0	0	0	0	4.029539	0.021269	0	0	0	0
100276169	LOC100276169	uncharacterized LOC100276169	0	0	0	0	0	0	1.952995	0.031435	2.005661	0.02652271

			Yuc x Ctr		Azo x Ctr		AzoYuc x Ctr		AzoYuc x Yuc		AzoYuc x Azo	
**Gene ID**	**Gene Symbol**	**Gene Description**	**Log2(FC)**	**pvalue**	**Log2(FC)**	**pvalue**	**Log2(FC)**	**pvalue**	**Log2(FC)**	**pvalue**	**Log2(FC)**	**pvalue**
100276236	umc2366	uncharacterized LOC100276236	0	0	0	0	0	0	-2.350588	0.0372681	0	0
100276246	pco101498	uncharacterized LOC100276246	0	0	0	0	-1.6548509	0.036157	0	0	0	0
100276267	IDP1437	uncharacterized LOC100276267	0	0	0	0	0	0	0	0	-2.710649	0.03198982
100276273	IDP1474	uncharacterized LOC100276273	0	0	0	0	2.7446123	0.010501	0	0	0	0
100276291	cl5598_1(581)	uncharacterized LOC100276291	0	0	0	0	3.3679023	0.012615	0	0	3.8126702	0.00418896
100276318	LOC100276318	uncharacterized LOC100276318	0	0	0	0	3.9622078	0.007153	4.321695	0.0032345	5.0302543	0.00094318
100276376	TIDP3268	uncharacterized LOC100276376	3.21493747	0.035089	0	0	0	0	0	0	0	0
100276388	LOC100276388	uncharacterized LOC100276388	3.4795556	0.032155	3.3531031	0.03843	5.1902758	0.000912	0	0	0	0
100276395	LOC100276395	uncharacterized LOC100276395	0	0	0	0	1.785715	0.046537	0	0	0	0
100276401	LOC100276401	uncharacterized LOC100276401	0	0	0	0	4.7082834	0.005495	2.939779	0.0389519	3.0396709	0.03210725
100276431	LOC100276431	uncharacterized LOC100276431	0	0	-1.524287	0.01623	0	0	0	0	0	0
100276434	LOC100276434	uncharacterized LOC100276434	0	0	0	0	0	0	1.599547	0.0264767	0	0
100276521	pco076094	uncharacterized LOC100276521	0	0	3.974491	0.01199	4.340231	0.005683	0	0	0	0

			Yuc x Ctr		Azo x Ctr		AzoYuc x Ctr		AzoYuc x Yuc		AzoYuc x Azo	
**Gene ID**	**Gene Symbol**	**Gene Description**	**Log2(FC)**	**pvalue**	**Log2(FC)**	**pvalue**	**Log2(FC)**	**pvalue**	**Log2(FC)**	**pvalue**	**Log2(FC)**	**pvalue**
100276526	LOC100276526	uncharacterized LOC100276526	0	0	-1.767678	0.01453	0	0	0	0	0	0
100276553	LOC100276553	uncharacterized LOC100276553	-1.6978776	0.013079	0	0	-1.5170453	0.024951	0	0	0	0
100276570	LOC100276570	uncharacterized LOC100276570	0	0	0	0	0	0	0	0	3.7585163	0.00762183
100276581	LOC100276581	uncharacterized LOC100276581	-1.756316	0.032483	0	0	-1.8002922	0.02638	0	0	0	0
100276587	LOC100276587	uncharacterized LOC100276587	0	0	0	0	2.7317398	0.001642	2.393588	0.0044598	2.9311499	0.00058064
100276802	pco097773	uncharacterized LOC100276802	0	0	0	0	0	0	0	0	-2.3338	0.02875072
100276845	LOC100276845	uncharacterized LOC100276845	0	0	0	0	-1.8833136	0.038287	0	0	0	0
100276855	umc1238	uncharacterized LOC100276855	0	0	0	0	0	0	0	0	-2.165457	0.03689917
100276899	LOC100276899	uncharacterized LOC100276899	0	0	0	0	0	0	0	0	3.0860253	0.04714647
100276922	LOC100276922	uncharacterized LOC100276922	-3.8721789	0.029871	0	0	0	0	0	0	0	0
100276955	LOC100276955	uncharacterized LOC100276955	0	0	1.8004113	0.02632	0	0	0	0	0	0
100276958	LOC100276958	uncharacterized LOC100276958	0	0	0	0	0	0	2.254538	0.0430023	0	0
100276977	LOC100276977	uncharacterized LOC100276977	0	0	0	0	2.1846288	0.046633	2.643916	0.0163894	2.4069027	0.02407916

			Yuc x Ctr		Azo x Ctr		AzoYuc x Ctr		AzoYuc x Yuc		AzoYuc x Azo	
**Gene ID**	**Gene Symbol**	**Gene Description**	**Log2(FC)**	**pvalue**	**Log2(FC)**	**pvalue**	**Log2(FC)**	**pvalue**	**Log2(FC)**	**pvalue**	**Log2(FC)**	**pvalue**
100276989	LOC100276989	uncharacterized LOC100276989	2.64933311	0.049485	0	0	0	0	0	0	0	0
100277035	pco133003	uncharacterized LOC100277035	0	0	0	0	1.951998	0.018886	2.334247	0.0049412	1.9602258	0.01622684
100277055	LOC100277055	uncharacterized LOC100277055	2.04194019	0.01706	0	0	0	0	-2.531351	0.0030083	0	0
100277126	LOC100277126	uncharacterized LOC100277126	-2.3388491	0.035949	0	0	0	0	0	0	0	0
100277172	LOC100277172	uncharacterized LOC100277172	-2.6594035	0.00905	-2.686566	0.00745	-2.0184142	0.035696	0	0	0	0
100277363	LOC100277363	uncharacterized LOC100277363	0	0	-3.805845	0.02025	0	0	0	0	0	0
100277380	LOC100277380	uncharacterized LOC100277380	0	0	0	0	3.7200508	0.026967	0	0	0	0
100277402	cl7681_1a	uncharacterized LOC100277402	0	0	0	0	0	0	0	0	-2.913372	0.04777346
100277500	umc2034	uncharacterized LOC100277500	0	0	0	0	0	0	2.736111	0.0389079	0	0
100277507	LOC100277507	uncharacterized LOC100277507	0	0	0	0	2.89239	0.003353	2.842264	0.0028156	1.8863896	0.03597868
100277551	LOC100277551	uncharacterized LOC100277551	4.40179006	0.003526	0	0	3.3647044	0.027361	0	0	0	0
100277562	pco136884	uncharacterized LOC100277562	0	0	2.099163	0.00081	0	0	0	0	-1.504086	0.00851262
100277564	LOC100277564	uncharacterized LOC100277564	0	0	0	0	-2.2310306	0.037196	0	0	0	0

			Yuc x Ctr		Azo x Ctr		AzoYuc x Ctr		AzoYuc x Yuc		AzoYuc x Azo	
**Gene ID**	**Gene Symbol**	**Gene Description**	**Log2(FC)**	**pvalue**	**Log2(FC)**	**pvalue**	**Log2(FC)**	**pvalue**	**Log2(FC)**	**pvalue**	**Log2(FC)**	**pvalue**
100277588	umc2512	uncharacterized LOC100277588	-2.2595235	0.012454	0	0	0	0	0	0	0	0
100277664	LOC100277664	uncharacterized LOC100277664	-3.3863018	0.001027	-3.995061	0.00014	-3.2240307	0.001554	0	0	0	0
100277683	LOC100277683	uncharacterized LOC100277683	0	0	0	0	2.7710684	0.011001	2.757849	0.0098366	2.2673668	0.02991505
100277689	LOC100277689	uncharacterized LOC100277689	0	0	0	0	0	0	-2.068221	0.044883	-2.239543	0.0276695
100277718	LOC100277718	uncharacterized LOC100277718	0	0	0	0	0	0	4.097712	0.0059624	0	0
100277730	LOC100277730	uncharacterized LOC100277730	0	0	0	0	0	0	1.829556	0.0295184	0	0
100277755	LOC100277755	uncharacterized LOC100277755	0	0	0	0	3.0292386	0.045099	0	0	0	0
100277777	LOC100277777	uncharacterized LOC100277777	0	0	0	0	3.2521263	0.045724	0	0	0	0
100277794	LOC100277794	uncharacterized LOC100277794	-4.0836844	0.049002	0	0	0	0	0	0	0	0
100277797	LOC100277797	uncharacterized LOC100277797	-5.1312441	0.009501	-4.147312	0.02907	-5.3571055	0.006704	0	0	0	0
100277822	LOC100277822	uncharacterized LOC100277822	0	0	0	0	-2.1819749	0.033996	0	0	0	0
100277858	LOC100277858	uncharacterized LOC100277858	0	0	0	0	3.0562797	0.011294	0	0	2.9370717	0.00816667
100277880	LOC100277880	uncharacterized LOC100277880	0	0	0	0	0	0	2.727271	0.0100755	0	0

			Yuc x Ctr		Azo x Ctr		AzoYuc x Ctr		AzoYuc x Yuc		AzoYuc x Azo	
**Gene ID**	**Gene Symbol**	**Gene Description**	**Log2(FC)**	**pvalue**	**Log2(FC)**	**pvalue**	**Log2(FC)**	**pvalue**	**Log2(FC)**	**pvalue**	**Log2(FC)**	**pvalue**
100277944	LOC100277944	uncharacterized LOC100277944	0	0	0	0	-1.5323284	0.043945	0	0	0	0
100277950	cl6482_1b	uncharacterized LOC100277950	2.24877614	0.02959	0	0	0	0	0	0	0	0
100278035	LOC100278035	uncharacterized LOC100278035	0	0	3.8634278	0.03628	0	0	0	0	0	0
100278056	LOC100278056	uncharacterized LOC100278056	0	0	0	0	2.3439208	0.015321	2.622069	0.0061736	2.5590745	0.00658906
100278071	LOC100278071	uncharacterized LOC100278071	0	0	-3.114495	0.03761	0	0	0	0	0	0
100278125	LOC100278125	uncharacterized LOC100278125	0	0	-1.693427	0.02033	0	0	0	0	0	0
100278194	LOC100278194	uncharacterized LOC100278194	0	0	0	0	0	0	0	0	3.2560193	0.01904606
100278201	LOC100278201	uncharacterized LOC100278201	0	0	2.8114594	0.02343	0	0	0	0	0	0
100278255	IDP353	uncharacterized LOC100278255	0	0	0	0	0	0	0	0	1.833375	0.0171706
100278279	LOC100278279	uncharacterized LOC100278279	0	0	0	0	0	0	0	0	2.0782328	0.03851539
100278302	LOC100278302	uncharacterized LOC100278302	0	0	0	0	2.7247532	0.027778	0	0	0	0
100278316	si946092e03	uncharacterized LOC100278316	1.55774951	0.020889	0	0	0	0	-1.947572	0.0032428	0	0
100278318	IDP667	uncharacterized LOC100278318	0	0	0	0	-2.6615268	0.019884	0	0	0	0

			Yuc x Ctr		Azo x Ctr		AzoYuc x Ctr		AzoYuc x Yuc		AzoYuc x Azo	
**Gene ID**	**Gene Symbol**	**Gene Description**	**Log2(FC)**	**pvalue**	**Log2(FC)**	**pvalue**	**Log2(FC)**	**pvalue**	**Log2(FC)**	**pvalue**	**Log2(FC)**	**pvalue**
100278326	LOC100278326	uncharacterized LOC100278326	3.49423461	0.021679	3.1015046	0.04235	0	0	0	0	0	0
100278328	LOC100278328	uncharacterized LOC100278328	0	0	0	0	0	0	2.446312	0.0191854	2.4704948	0.01658108
100278395	LOC100278395	uncharacterized LOC100278395	0	0	0	0	-1.8111634	0.020709	0	0	0	0
100278460	LOC100278460	uncharacterized LOC100278460	0	0	0	0	0	0	2.602849	0.0087814	2.7204177	0.00609619
100278461	LOC100278461	uncharacterized LOC100278461	0	0	0	0	0	0	1.521925	0.0005361	0	0
100278465	LOC100278465	uncharacterized LOC100278465	-2.7699553	0.011682	0	0	0	0	0	0	0	0
100278522	LOC100278522	uncharacterized LOC100278522	0	0	-2.673047	0.01009	0	0	0	0	0	0
100278542	LOC100278542	uncharacterized LOC100278542	0	0	0	0	0	0	-2.475205	0.0337046	0	0
100278555	LOC100278555	uncharacterized LOC100278555	0	0	2.5394636	0.03639	0	0	0	0	0	0
100278616	LOC100278616	uncharacterized LOC100278616	0	0	0	0	2.084328	0.049049	0	0	0	0
100278628	LOC100278628	uncharacterized LOC100278628	0	0	0	0	0	0	2.052782	0.0339461	0	0
100278657	LOC100278657	uncharacterized LOC100278657	0	0	-3.227878	0.02944	0	0	0	0	0	0
100278713	LOC100278713	uncharacterized LOC100278713	0	0	0	0	3.8258994	0.002981	4.578957	0.0005048	4.0359623	0.00137684

			Yuc x Ctr		Azo x Ctr		AzoYuc x Ctr		AzoYuc x Yuc		AzoYuc x Azo	
**Gene ID**	**Gene Symbol**	**Gene Description**	**Log2(FC)**	**pvalue**	**Log2(FC)**	**pvalue**	**Log2(FC)**	**pvalue**	**Log2(FC)**	**pvalue**	**Log2(FC)**	**pvalue**
100278787	LOC100278787	uncharacterized LOC100278787	0	0	0	0	0	0	-1.912037	0.0226877	0	0
100278838	LOC100278838	uncharacterized LOC100278838	-3.9358592	0.012361	0	0	0	0	3.47151	0.0266551	0	0
100278859	LOC100278859	uncharacterized LOC100278859	0	0	0	0	2.2068463	0.033578	1.962097	0.0413341	0	0
100278862	LOC100278862	uncharacterized LOC100278862	0	0	2.1449991	0.03618	0	0	0	0	0	0
100278869	LOC100278869	uncharacterized LOC100278869	0	0	0	0	-2.2340532	0.028963	0	0	0	0
100278901	LOC100278901	uncharacterized LOC100278901	0	0	0	0	0	0	2.207422	0.0334615	2.3732328	0.0219217
100279003	LOC100279003	uncharacterized LOC100279003	0	0	0	0	1.8004412	0.043511	2.238501	0.0122079	0	0
100279006	LOC100279006	uncharacterized LOC100279006	0	0	0	0	2.1506081	0.036039	0	0	0	0
100279155	LOC100279155	uncharacterized LOC100279155	0	0	0	0	0	0	1.515587	0.0264055	0	0
100279197	LOC100279197	uncharacterized LOC100279197	0	0	-3.052088	0.0261	0	0	0	0	0	0
100279214	LOC100279214	uncharacterized LOC100279214	-4.8239552	0.021584	0	0	0	0	0	0	0	0
100279239	cl21263_1	uncharacterized LOC100279239	0	0	0	0	2.1831084	0.002397	2.389308	0.0007801	2.0747863	0.00286285
100279249	LOC100279249	uncharacterized LOC100279249	0	0	0	0	2.1356459	0.000726	0	0	0	0

			Yuc x Ctr		Azo x Ctr		AzoYuc x Ctr		AzoYuc x Yuc		AzoYuc x Azo	
**Gene ID**	**Gene Symbol**	**Gene Description**	**Log2(FC)**	**pvalue**	**Log2(FC)**	**pvalue**	**Log2(FC)**	**pvalue**	**Log2(FC)**	**pvalue**	**Log2(FC)**	**pvalue**
100279259	LOC100279259	uncharacterized LOC100279259	0	0	0	0	2.4698588	0.047512	0	0	0	0
100279299	LOC100279299	uncharacterized LOC100279299	0	0	0	0	1.888341	0.03478	0	0	0	0
100279324	LOC100279324	uncharacterized LOC100279324	0	0	0	0	4.4675361	0.003956	0	0	0	0
100279364	LOC100279364	uncharacterized LOC100279364	-3.9815156	0.00471	-4.868617	0.00065	-3.9136703	0.005276	0	0	0	0
100279366	pco123556	uncharacterized LOC100279366	0	0	-2.299451	0.00072	-2.3760207	0.000475	0	0	0	0
100279369	LOC100279369	uncharacterized LOC100279369	0	0	0	0	2.3365608	0.017874	0	0	0	0
100279390	LOC100279390	uncharacterized LOC100279390	3.10345212	0.013793	3.286201	0.00866	3.1164763	0.012955	0	0	0	0
100279423	umc1322	uncharacterized LOC100279423	0	0	0	0	4.1300887	0.033465	0	0	0	0
100279455	pco076369b	uncharacterized LOC100279455	0	0	0	0	1.7554151	0.029521	0	0	0	0
100279471	LOC100279471	uncharacterized LOC100279471	0	0	0	0	-3.2091976	0.028531	0	0	0	0
100279525	LOC100279525	uncharacterized LOC100279525	0	0	0	0	2.1766223	0.021597	3.195879	0.0010501	2.5031051	0.00760522
100279655	IDP1984	uncharacterized LOC100279655	0	0	0	0	1.9633076	0.00071	0	0	0	0
100279714	umc2615	uncharacterized LOC100279714	1.8850073	0.021457	0	0	2.4225988	0.002576	0	0	0	0

			Yuc x Ctr		Azo x Ctr		AzoYuc x Ctr		AzoYuc x Yuc		AzoYuc x Azo	
**Gene ID**	**Gene Symbol**	**Gene Description**	**Log2(FC)**	**pvalue**	**Log2(FC)**	**pvalue**	**Log2(FC)**	**pvalue**	**Log2(FC)**	**pvalue**	**Log2(FC)**	**pvalue**
100279717	cl715_-2a	uncharacterized LOC100279717	0	0	0	0	2.0666716	0.012212	1.769244	0.028044	2.0585931	0.01080401
100279753	LOC100279753	uncharacterized LOC100279753	0	0	0	0	0	0	3.182001	0.0022101	3.7430098	0.00041388
100279761	LOC100279761	uncharacterized LOC100279761	-3.135828	0.009944	-4.031075	0.00219	-3.1566275	0.007948	0	0	0	0
100279862	LOC100279862	uncharacterized LOC100279862	0	0	0	0	2.4891405	0.014411	0	0	0	0
100279863	LOC100279863	uncharacterized LOC100279863	0	0	3.2743829	0.01141	0	0	0	0	-3.265468	0.00848982
100279897	LOC100279897	uncharacterized LOC100279897	0	0	0	0	0	0	2.226469	0.0043414	0	0
100279908	pco101952	uncharacterized LOC100279908	0	0	0	0	2.247108	0.000305	2.254277	0.000245	2.4546598	6.5455E-05
100279915	LOC100279915	uncharacterized LOC100279915	-1.8521075	0.000872	0	0	0	0	0	0	0	0
100279917	LOC100279917	uncharacterized LOC100279917	0	0	0	0	0	0	-1.653133	0.0133976	0	0
100279942	cl40794_1	uncharacterized LOC100279942	0	0	0	0	0	0	2.333115	0.0222308	2.6236561	0.0104873
100279985	LOC100279985	uncharacterized LOC100279985	-1.569972	0.022403	0	0	0	0	0	0	0	0
100279991	LOC100279991	uncharacterized LOC100279991	0	0	0	0	0	0	0	0	-4.220807	0.04909685
100280005	LOC100280005	uncharacterized LOC100280005	0	0	0	0	2.4721817	0.042553	0	0	0	0

			Yuc x Ctr		Azo x Ctr		AzoYuc x Ctr		AzoYuc x Yuc		AzoYuc x Azo	
**Gene ID**	**Gene Symbol**	**Gene Description**	**Log2(FC)**	**pvalue**	**Log2(FC)**	**pvalue**	**Log2(FC)**	**pvalue**	**Log2(FC)**	**pvalue**	**Log2(FC)**	**pvalue**
100280049	LOC100280049	uncharacterized LOC100280049	0	0	0	0	4.0843365	0.01724	0	0	0	0
100280100	LOC100280100	uncharacterized LOC100280100	0	0	0	0	2.5402771	0.002228	2.659078	0.001109	1.6911426	0.02950655
100280106	LOC100280106	uncharacterized LOC100280106	0	0	0	0	3.0968129	0.014385	2.871568	0.0167988	2.8842012	0.01501704
100280146	LOC100280146	uncharacterized LOC100280146	0	0	0	0	-3.774919	0.027814	-4.545411	0.00725	0	0
100280165	LOC100280165	uncharacterized LOC100280165	0	0	0	0	3.7629828	0.01186	0	0	0	0
100280286	LOC100280286	uncharacterized LOC100280286	-4.4489256	9.46E-06	-4.119237	3E-05	-3.4717646	0.000352	0	0	0	0
100280323	LOC100280323	uncharacterized LOC100280323	0	0	0	0	1.843845	0.007138	2.047268	0.0027278	1.9118791	0.00494903
100280354	LOC100280354	uncharacterized LOC100280354	-1.72767	0.028315	-2.010917	0.01108	0	0	0	0	0	0
100280368	LOC100280368	uncharacterized LOC100280368	0	0	0	0	0	0	3.420515	0.007446	3.4995748	0.0056476
100280415	LOC100280415	uncharacterized LOC100280415	0	0	0	0	2.2527055	0.021991	2.426233	0.0122714	0	0
100280438	LOC100280438	uncharacterized LOC100280438	1.52311554	0.032855	0	0	0	0	0	0	0	0
100280497	gpm667	uncharacterized LOC100280497	0	0	0	0	0	0	2.458133	0.015894	2.4820175	0.01396043
100280586	si605047e03	uncharacterized LOC100280586	-2.1835159	0.003717	-2.490465	0.001	-2.062606	0.005333	0	0	0	0

			Yuc x Ctr		Azo x Ctr		AzoYuc x Ctr		AzoYuc x Yuc		AzoYuc x Azo	
**Gene ID**	**Gene Symbol**	**Gene Description**	**Log2(FC)**	**pvalue**	**Log2(FC)**	**pvalue**	**Log2(FC)**	**pvalue**	**Log2(FC)**	**pvalue**	**Log2(FC)**	**pvalue**
100280589	pco099218(710)	uncharacterized LOC100280589	-1.636848	0.035694	0	0	-1.5179499	0.049698	0	0	0	0
100280710	TIDP3391	uncharacterized LOC100280710	0	0	0	0	-2.3389535	0.002119	0	0	0	0
100280770	pco066751	uncharacterized LOC100280770	1.78568213	0.033248	0	0	0	0	0	0	0	0
100280824	uaz235(px)	uncharacterized LOC100280824	0	0	3.226964	0.00656	0	0	0	0	-4.861949	0.00015583
100280958	IDP641	uncharacterized LOC100280958	0	0	0	0	-1.6013123	0.035411	0	0	0	0
100281002	pco108588	uncharacterized LOC100281002	0	0	-1.879238	0.02334	0	0	0	0	0	0
100281016	si603006c09	uncharacterized LOC100281016	0	0	2.6224452	0.01355	0	0	0	0	-2.123374	0.02010719
100281034	pco102615	uncharacterized LOC100281034	0	0	-1.550104	0.02404	0	0	0	0	0	0
100281131	gpm680	uncharacterized LOC100281131	-2.195424	0.045234	0	0	0	0	0	0	0	0
100281166	si687013d08	uncharacterized LOC100281166	2.41548105	0.000651	0	0	0	0	0	0	0	0
100281219	pco141803	uncharacterized LOC100281219	0	0	0	0	0	0	1.749642	0.0117001	0	0
100281298	si687036a06	uncharacterized LOC100281298	4.05614602	0.003189	0	0	0	0	-2.433243	0.0443778	0	0
100281394	si707002b03b	uncharacterized LOC100281394	0	0	0	0	3.3198498	0.008459	2.926901	0.0136555	0	0

			Yuc x Ctr		Azo x Ctr		AzoYuc x Ctr		AzoYuc x Yuc		AzoYuc x Azo	
**Gene ID**	**Gene Symbol**	**Gene Description**	**Log2(FC)**	**pvalue**	**Log2(FC)**	**pvalue**	**Log2(FC)**	**pvalue**	**Log2(FC)**	**pvalue**	**Log2(FC)**	**pvalue**
100281422	cl29544_1a	uncharacterized LOC100281422	0	0	0	0	2.07988	0.024041	0	0	2.0291687	0.02607733
100281458	pco141348	uncharacterized LOC100281458	0	0	0	0	0	0	0	0	-2.222642	0.02639941
100281468	magi100654	uncharacterized LOC100281468	0	0	1.5314657	0.04005	0	0	0	0	0	0
100281503	umc1155	uncharacterized LOC100281503	0	0	0	0	1.8999004	0.003869	0	0	0	0
100281507	pco138672	uncharacterized LOC100281507	2.64999218	0.013922	0	0	0	0	0	0	0	0
100281537	si605069e01	uncharacterized LOC100281537	0	0	0	0	2.4243538	0.024705	2.35806	0.024493	2.2535698	0.02916338
100281767	pco104637	uncharacterized LOC100281767	0	0	0	0	0	0	0	0	-1.968679	0.02005684
100281771	cl18044_1	uncharacterized LOC100281771	-2.6021897	0.024377	0	0	0	0	0	0	0	0
100282018	umc2196	uncharacterized LOC100282018	0	0	0	0	0	0	-2.198676	0.0154698	0	0
100282310	umc1314	uncharacterized LOC100282310	0	0	-1.509667	0.02005	0	0	0	0	0	0
100282455	pco106809	uncharacterized LOC100282455	0	0	-2.198095	0.00261	-1.8662101	0.009111	0	0	0	0
100282684	pco120183	uncharacterized LOC100282684	0	0	0	0	1.822801	0.009688	0	0	0	0
100282946	gpm447	uncharacterized LOC100282946	0	0	0	0	-1.7064577	0.003556	0	0	0	0

			Yuc x Ctr		Azo x Ctr		AzoYuc x Ctr		AzoYuc x Yuc		AzoYuc x Azo	
**Gene ID**	**Gene Symbol**	**Gene Description**	**Log2(FC)**	**pvalue**	**Log2(FC)**	**pvalue**	**Log2(FC)**	**pvalue**	**Log2(FC)**	**pvalue**	**Log2(FC)**	**pvalue**
100283086	pco084622	uncharacterized LOC100283086	0	0	0	0	2.1339196	0.026712	0	0	0	0
100283169	umc2388	uncharacterized LOC100283169	0	0	0	0	1.9058368	0.000259	0	0	0	0
100283198	pco133953b	uncharacterized LOC100283198	0	0	-1.597518	0.01523	0	0	0	0	0	0
100283272	pco138129	uncharacterized LOC100283272	0	0	0	0	0	0	1.748452	0.008961	0	0
100283315	pco087009	uncharacterized LOC100283315	0	0	-1.94959	0.03424	-2.697026	0.005543	0	0	0	0
100283321	cl421_1	uncharacterized LOC100283321	2.68434888	0.005654	0	0	0	0	-2.755013	0.0031817	0	0
100283417	pco127462b	uncharacterized LOC100283417	1.90634645	0.002087	0	0	0	0	0	0	0	0
100283481	cl15601_2	uncharacterized LOC100283481	0	0	0	0	3.474748	0.02419	3.865545	0.0114967	0	0
100283761	pco066552	uncharacterized LOC100283761	-1.6609796	0.04283	0	0	-1.6250302	0.044402	0	0	0	0
100284077	pco109288	uncharacterized LOC100284077	4.82599207	0.006114	0	0	0	0	-2.89492	0.0422918	0	0
100284102	gpm917	uncharacterized LOC100284102	0	0	0	0	0	0	2.816157	0.0171244	0	0
100284215	pco101548(16)	uncharacterized LOC100284215	0	0	0	0	1.9192384	0.023764	1.808864	0.0279607	1.8592094	0.02288189
100284334	IDP1611	uncharacterized LOC100284334	0	0	0	0	-1.8390633	0.002439	0	0	0	0

			Yuc x Ctr		Azo x Ctr		AzoYuc x Ctr		AzoYuc x Yuc		AzoYuc x Azo	
**Gene ID**	**Gene Symbol**	**Gene Description**	**Log2(FC)**	**pvalue**	**Log2(FC)**	**pvalue**	**Log2(FC)**	**pvalue**	**Log2(FC)**	**pvalue**	**Log2(FC)**	**pvalue**
100284351	umc2542	uncharacterized LOC100284351	0	0	0	0	2.079388	0.000362	0	0	1.8541455	0.00088311
100284363	si707024f02	uncharacterized LOC100284363	0	0	0	0	1.5763669	0.046822	0	0	0	0
100284589	cl362_2b	uncharacterized LOC100284589	1.6742817	0.035565	0	0	0	0	0	0	0	0
100284700	pco142662	uncharacterized LOC100284700	0	0	-1.836335	0.01056	0	0	1.618614	0.0206173	2.3361661	0.00099997
100284708	umc2624	uncharacterized LOC100284708	-1.8764007	0.007895	0	0	0	0	0	0	0	0
100284832	si614088f03a	uncharacterized LOC100284832	-1.7325686	0.029551	-1.73495	0.02827	-2.3751802	0.003332	0	0	0	0
100284871	pco093706	uncharacterized LOC100284871	1.85979548	0.024184	0	0	0	0	-1.546513	0.0367862	0	0
100284970	cl1774_-2(594)	uncharacterized LOC100284970	-5.0121175	0.005699	-3.330316	0.03239	0	0	0	0	0	0
100285006	umc2775	uncharacterized LOC100285006	2.03743855	0.025547	0	0	3.0936893	0.000538	0	0	1.5524175	0.0386727
100285339	cl4013_1	uncharacterized LOC100285339	0	0	-1.780489	0.00278	0	0	0	0	0	0
100285342	umc2759	uncharacterized LOC100285342	0	0	0	0	-2.4401527	0.011833	0	0	0	0
100285440	cl574_1	uncharacterized LOC100285440	-4.4648392	0.006192	0	0	0	0	4.834755	0.0024302	0	0
100285558	pco148653	uncharacterized LOC100285558	-1.9485546	0.009374	-1.933956	0.00894	-1.881124	0.010558	0	0	0	0

			Yuc x Ctr		Azo x Ctr		AzoYuc x Ctr		AzoYuc x Yuc		AzoYuc x Azo	
**Gene ID**	**Gene Symbol**	**Gene Description**	**Log2(FC)**	**pvalue**	**Log2(FC)**	**pvalue**	**Log2(FC)**	**pvalue**	**Log2(FC)**	**pvalue**	**Log2(FC)**	**pvalue**
100285816	LOC100285816	uncharacterized LOC100285816	0	0	0	0	3.0672134	0.029051	0	0	0	0
100285908	cl7206_1	uncharacterized LOC100285908	-1.8585573	0.021238	0	0	0	0	0	0	0	0
100285911	pco148010	uncharacterized LOC100285911	0	0	0	0	0	0	2.910441	0.0379497	2.7348375	0.04681943
100285948	cl31549_1	uncharacterized LOC100285948	0	0	0	0	0	0	0	0	2.7203512	0.04097448
100286147	si605092h09	uncharacterized LOC100286147	6.04545034	0.004364	0	0	0	0	-5.411068	0.0060484	0	0
100286373	cl27617_1b	uncharacterized LOC100286373	0	0	-2.136759	0.00476	-1.6282219	0.023682	0	0	0	0
100303788	LOC100303788	uncharacterized LOC100303788	0	0	-2.227822	0.03358	0	0	0	0	0	0
100303858	LOC100303858	uncharacterized LOC100303858	3.26191991	0.046851	0	0	0	0	0	0	0	0
100304181	LOC100304181	uncharacterized LOC100304181	0	0	0	0	2.0411403	0.039094	0	0	0	0
100304309	LOC100304309	uncharacterized LOC100304309	0	0	0	0	1.7863466	0.039427	0	0	0	0
100304328	LOC100304328	uncharacterized LOC100304328	2.03174037	0.03517	0	0	0	0	0	0	0	0
100304388	LOC100304388	uncharacterized LOC100304388	0	0	0	0	3.9213976	0.00263	4.195508	0.0010941	3.6610011	0.00251662
100381417	LOC100381417	uncharacterized LOC100381417	2.13559011	0.004441	0	0	0	0	-1.83978	0.0116545	0	0

			Yuc x Ctr		Azo x Ctr		AzoYuc x Ctr		AzoYuc x Yuc		AzoYuc x Azo	
**Gene ID**	**Gene Symbol**	**Gene Description**	**Log2(FC)**	**pvalue**	**Log2(FC)**	**pvalue**	**Log2(FC)**	**pvalue**	**Log2(FC)**	**pvalue**	**Log2(FC)**	**pvalue**
100381420	LOC100381420	uncharacterized LOC100381420	0	0	0	0	1.8592934	0.045429	1.902798	0.0346983	0	0
100381430	LOC100381430	uncharacterized LOC100381430	0	0	0	0	0	0	4.309354	0.0246097	0	0
100381474	LOC100381474	uncharacterized LOC100381474	0	0	0	0	3.0989998	0.003633	2.868967	0.005187	2.9610085	0.00374059
100381493	LOC100381493	uncharacterized LOC100381493	-2.0304927	0.010633	0	0	0	0	1.559423	0.0485716	0	0
100381535	LOC100381535	uncharacterized LOC100381535	0	0	1.5548614	0.00186	0	0	0	0	0	0
100381550	LOC100381550	uncharacterized LOC100381550	-2.2776491	4.13E-05	-2.369287	2E-05	-2.3113622	3.04E-05	0	0	0	0
100381562	LOC100381562	uncharacterized LOC100381562	-1.6797112	0.020507	0	0	0	0	0	0	0	0
100381570	LOC100381570	uncharacterized LOC100381570	-1.641366	0.001577	0	0	0	0	0	0	0	0
100381574	LOC100381574	uncharacterized LOC100381574	0	0	0	0	2.6103827	0.022926	0	0	0	0
100381723	LOC100381723	uncharacterized LOC100381723	0	0	0	0	0	0	2.349386	0.0139432	0	0
100381760	pco065117	uncharacterized LOC100381760	-1.7334226	0.015735	-1.703183	0.01745	-1.7278601	0.015842	0	0	0	0
100381855	LOC100381855	uncharacterized LOC100381855	-3.4701058	0.032257	0	0	0	0	0	0	0	0
100381900	LOC100381900	uncharacterized LOC100381900	0	0	0	0	6.3215116	0.004039	5.846208	0.0058114	4.0489193	0.03565179

			Yuc x Ctr		Azo x Ctr		AzoYuc x Ctr		AzoYuc x Yuc		AzoYuc x Azo	
**Gene ID**	**Gene Symbol**	**Gene Description**	**Log2(FC)**	**pvalue**	**Log2(FC)**	**pvalue**	**Log2(FC)**	**pvalue**	**Log2(FC)**	**pvalue**	**Log2(FC)**	**pvalue**
100381927	LOC100381927	uncharacterized LOC100381927	-3.2678582	0.029546	0	0	0	0	0	0	0	0
100381936	LOC100381936	uncharacterized LOC100381936	0	0	2.1550417	0.001	0	0	0	0	-1.780835	0.00527283
100382105	LOC100382105	uncharacterized LOC100382105	0	0	0	0	3.1680696	0.002003	3.351993	0.000934	3.4623089	0.00059561
100382148	LOC100382148	uncharacterized LOC100382148	0	0	0	0	2.4040697	0.009472	0	0	2.2022966	0.0120173
100382289	LOC100382289	uncharacterized LOC100382289	0	0	0	0	0	0	1.692985	0.0008831	0	0
100382339	LOC100382339	uncharacterized LOC100382339	-2.4037984	0.012211	0	0	0	0	1.932993	0.04228	0	0
100382354	LOC100382354	uncharacterized LOC100382354	0	0	0	0	0	0	3.773645	0.012311	2.9728643	0.03456442
100382367	TIDP2768	uncharacterized LOC100382367	0	0	3.2268313	0.00822	0	0	0	0	-3.018104	0.00904944
100382396	LOC100382396	uncharacterized LOC100382396	0	0	-3.95648	0.04368	0	0	0	0	0	0
100382441	LOC100382441	uncharacterized LOC100382441	0	0	0	0	0	0	-2.840222	0.0315178	0	0
100382444	LOC100382444	uncharacterized LOC100382444	0	0	0	0	0	0	0	0	2.0265528	0.01648165
100382497	LOC100382497	uncharacterized LOC100382497	-2.0494018	0.030554	-2.855621	0.00363	-2.3318195	0.014	0	0	0	0
100382515	LOC100382515	uncharacterized LOC100382515	0	0	0	0	4.9829374	0.009279	3.361418	0.0428607	4.1328256	0.01656627

			Yuc x Ctr		Azo x Ctr		AzoYuc x Ctr		AzoYuc x Yuc		AzoYuc x Azo	
**Gene ID**	**Gene Symbol**	**Gene Description**	**Log2(FC)**	**pvalue**	**Log2(FC)**	**pvalue**	**Log2(FC)**	**pvalue**	**Log2(FC)**	**pvalue**	**Log2(FC)**	**pvalue**
100382629	LOC100382629	uncharacterized LOC100382629	0	0	-1.899676	0.04516	-2.4138452	0.011392	0	0	0	0
100382694	LOC100382694	uncharacterized LOC100382694	0	0	3.5655633	0.02351	0	0	0	0	-2.95644	0.03038569
100382748	LOC100382748	uncharacterized LOC100382748	0	0	2.0125119	0.02496	2.0379526	0.022919	0	0	0	0
100382837	LOC100382837	uncharacterized LOC100382837	0	0	0	0	2.5536243	0.033412	2.553577	0.0279328	2.355677	0.0375352
100382848	LOC100382848	uncharacterized LOC100382848	0	0	3.9691451	0.00018	0	0	0	0	-3.49262	0.00059656
100382849	LOC100382849	uncharacterized LOC100382849	0	0	4.4716894	0.0194	0	0	0	0	0	0
100382925	si946015a02(578)	uncharacterized LOC100382925	0	0	0	0	2.054934	0.010141	2.599962	0.0011776	2.976281	0.0002169
100382927	LOC100382927	uncharacterized LOC100382927	0	0	0	0	0	0	1.605036	0.0277156	0	0
100382929	LOC100382929	uncharacterized LOC100382929	0	0	-2.81678	0.04869	0	0	0	0	0	0
100382956	LOC100382956	uncharacterized LOC100382956	0	0	0	0	0	0	3.997631	0.0040701	0	0
100383117	LOC100383117	uncharacterized LOC100383117	0	0	2.4146359	0.01776	2.102489	0.04024	0	0	0	0
100383128	LOC100383128	uncharacterized LOC100383128	0	0	0	0	1.9428979	0.008385	1.64149	0.017469	1.5732294	0.02023354
100383285	LOC100383285	uncharacterized LOC100383285	0	0	0	0	0	0	2.103682	0.0131869	0	0

			Yuc x Ctr		Azo x Ctr		AzoYuc x Ctr		AzoYuc x Yuc		AzoYuc x Azo	
**Gene ID**	**Gene Symbol**	**Gene Description**	**Log2(FC)**	**pvalue**	**Log2(FC)**	**pvalue**	**Log2(FC)**	**pvalue**	**Log2(FC)**	**pvalue**	**Log2(FC)**	**pvalue**
100383323	LOC100383323	uncharacterized LOC100383323	0	0	-2.751064	0.00847	0	0	0	0	2.7671069	0.00708341
100383331	LOC100383331	uncharacterized LOC100383331	0	0	0	0	2.8640129	0.046356	2.725424	0.0499174	0	0
100383493	LOC100383493	uncharacterized LOC100383493	1.87621016	0.002434	0	0	0	0	-1.542912	0.0109053	0	0
100383495	LOC100383495	uncharacterized LOC100383495	-1.9966857	0.021652	-2.452167	0.00504	-3.3749796	0.00017	0	0	0	0
100383497	LOC100383497	uncharacterized LOC100383497	2.72476415	0.0066	0	0	0	0	-1.992879	0.0271678	0	0
100383501	TIDP3746	uncharacterized LOC100383501	0	0	0	0	1.9905047	0.044035	0	0	1.9473149	0.033465
100383508	LOC100383508	uncharacterized LOC100383508	0	0	0	0	5.5116168	0.000948	3.48217	0.0084654	3.5733036	0.00645564
100383513	LOC100383513	uncharacterized LOC100383513	0	0	0	0	0	0	-1.723702	0.0041794	0	0
100383606	LOC100383606	uncharacterized LOC100383606	0	0	0	0	2.6308219	0.012856	0	0	0	0
100383614	LOC100383614	uncharacterized LOC100383614	0	0	0	0	0	0	1.547897	0.0407416	0	0
100383655	LOC100383655	uncharacterized LOC100383655	0	0	0	0	-2.2846177	0.045893	0	0	0	0
100383661	LOC100383661	uncharacterized LOC100383661	0	0	0	0	0	0	2.132099	0.0363451	2.0010212	0.04528774
100383715	LOC100383715	uncharacterized LOC100383715	0	0	0	0	-3.3538701	0.048945	0	0	0	0

			Yuc x Ctr		Azo x Ctr		AzoYuc x Ctr		AzoYuc x Yuc		AzoYuc x Azo	
**Gene ID**	**Gene Symbol**	**Gene Description**	**Log2(FC)**	**pvalue**	**Log2(FC)**	**pvalue**	**Log2(FC)**	**pvalue**	**Log2(FC)**	**pvalue**	**Log2(FC)**	**pvalue**
100383726	LOC100383726	uncharacterized LOC100383726	0	0	0	0	0	0	4.441209	0.0223057	4.6671389	0.0163339
100383771	LOC100383771	uncharacterized LOC100383771	0	0	-2.149342	0.02508	-2.8185974	0.005016	0	0	0	0
100383810	LOC100383810	uncharacterized LOC100383810	0	0	0	0	3.9958118	0.041721	0	0	0	0
100383860	LOC100383860	uncharacterized LOC100383860	-1.5753899	0.043578	0	0	-1.5278355	0.046919	0	0	0	0
100383887	LOC100383887	uncharacterized LOC100383887	0	0	0	0	-1.7599148	0.018578	0	0	0	0
100383941	LOC100383941	uncharacterized LOC100383941	-4.5729042	0.008439	0	0	0	0	4.277855	0.0129916	0	0
100384029	LOC100384029	uncharacterized LOC100384029	0	0	0	0	0	0	3.935416	0.0490677	0	0
100384037	LOC100384037	uncharacterized LOC100384037	0	0	0	0	0	0	2.17678	0.0071298	1.7971558	0.02472344
100384052	LOC100384052	uncharacterized LOC100384052	0	0	0	0	0	0	-1.855241	0.0085707	0	0
100384061	LOC100384061	uncharacterized LOC100384061	-4.7790753	0.011675	-5.798518	0.00273	-6.6736095	0.000855	0	0	0	0
100384180	LOC100384180	uncharacterized LOC100384180	0	0	0	0	0	0	4.418424	0.0308711	0	0
100384269	LOC100384269	uncharacterized LOC100384269	0	0	0	0	0	0	1.667912	0.0118914	1.724205	0.00910795
100384296	umc2190	uncharacterized LOC100384296	-2.7213779	0.043675	0	0	0	0	0	0	0	0

			Yuc x Ctr		Azo x Ctr		AzoYuc x Ctr		AzoYuc x Yuc		AzoYuc x Azo	
**Gene ID**	**Gene Symbol**	**Gene Description**	**Log2(FC)**	**pvalue**	**Log2(FC)**	**pvalue**	**Log2(FC)**	**pvalue**	**Log2(FC)**	**pvalue**	**Log2(FC)**	**pvalue**
100384354	LOC100384354	uncharacterized LOC100384354	0	0	0	0	0	0	0	0	3.9654267	0.01459107
100384436	LOC100384436	uncharacterized LOC100384436	-2.3394191	0.000572	-1.940056	0.00361	-1.5413751	0.019464	0	0	0	0
100384533	LOC100384533	uncharacterized LOC100384533	0	0	0	0	3.7017382	0.002271	3.527684	0.0023975	3.9339292	0.00082794
100384790	LOC100384790	uncharacterized LOC100384790	-2.6551571	0.004104	0	0	0	0	0	0	0	0
100384837	LOC100384837	uncharacterized LOC100384837	0	0	0	0	0	0	2.09215	0.0016817	0	0
100384854	LOC100384854	uncharacterized LOC100384854	0	0	0	0	0	0	3.07894	0.049492	0	0
100500963	LOC100500963	uncharacterized LOC100500963	0	0	0	0	-2.0041488	0.045403	0	0	0	0
100501018	LOC100501018	uncharacterized LOC100501018	3.20558051	0.023657	0	0	0	0	-2.54434	0.0292162	0	0
100501100	LOC100501100	uncharacterized LOC100501100	0	0	0	0	0	0	0	0	2.8421251	0.00936329
100501222	LOC100501222	uncharacterized LOC100501222	0	0	0	0	0	0	2.231193	0.0335384	0	0
100501255	LOC100501255	uncharacterized LOC100501255	0	0	0	0	0	0	0	0	2.7011054	0.02945504
100501315	LOC100501315	uncharacterized LOC100501315	-2.2024784	0.003465	-2.576977	0.00065	-2.4663062	0.001065	0	0	0	0
100501385	LOC100501385	uncharacterized LOC100501385	0	0	1.8714221	0.03474	0	0	0	0	0	0

			Yuc x Ctr		Azo x Ctr		AzoYuc x Ctr		AzoYuc x Yuc		AzoYuc x Azo	
**Gene ID**	**Gene Symbol**	**Gene Description**	**Log2(FC)**	**pvalue**	**Log2(FC)**	**pvalue**	**Log2(FC)**	**pvalue**	**Log2(FC)**	**pvalue**	**Log2(FC)**	**pvalue**
100501426	LOC100501426	uncharacterized LOC100501426	-2.2124459	0.008543	-2.537092	0.00267	-2.4093541	0.004121	0	0	0	0
100501437	LOC100501437	uncharacterized LOC100501437	4.84974693	0.001365	3.3444143	0.0321	3.7595088	0.014625	0	0	0	0
100501464	LOC100501464	uncharacterized LOC100501464	0	0	0	0	0	0	0	0	1.83494	0.03714522
100501650	LOC100501650	uncharacterized LOC100501650	0	0	0	0	-3.5538869	0.016625	0	0	0	0
100501673	LOC100501673	uncharacterized LOC100501673	0	0	0	0	0	0	0	0	3.4311799	0.01280127
100501712	LOC100501712	uncharacterized LOC100501712	0	0	0	0	2.3370358	0.007639	1.933195	0.0221918	0	0
100501753	LOC100501753	uncharacterized LOC100501753	0	0	0	0	1.9740268	0.009697	0	0	0	0
100501789	LOC100501789	uncharacterized LOC100501789	-1.8537725	0.006651	0	0	0	0	0	0	0	0
100501821	LOC100501821	uncharacterized LOC100501821	0	0	0	0	0	0	-1.554874	0.0192768	0	0
100501921	LOC100501921	uncharacterized LOC100501921	0	0	0	0	3.8776976	0.011793	0	0	0	0
100501994	LOC100501994	uncharacterized LOC100501994	-3.2247412	0.00068	0	0	-1.7833183	0.021927	0	0	0	0
100502152	LOC100502152	uncharacterized LOC100502152	1.51115305	0.01947	0	0	0	0	0	0	0	0
100502153	LOC100502153	uncharacterized LOC100502153	3.54767514	0.044717	0	0	0	0	0	0	0	0

			Yuc x Ctr		Azo x Ctr		AzoYuc x Ctr		AzoYuc x Yuc		AzoYuc x Azo	
**Gene ID**	**Gene Symbol**	**Gene Description**	**Log2(FC)**	**pvalue**	**Log2(FC)**	**pvalue**	**Log2(FC)**	**pvalue**	**Log2(FC)**	**pvalue**	**Log2(FC)**	**pvalue**
100502159	LOC100502159	uncharacterized LOC100502159	0	0	0	0	1.5596231	0.041018	0	0	1.9689936	0.00924988
100502162	LOC100502162	uncharacterized LOC100502162	0	0	0	0	0	0	3.710266	0.0283214	0	0
100502267	LOC100502267	uncharacterized LOC100502267	-2.9090924	0.031365	0	0	0	0	3.017377	0.0224794	0	0
100502313	LOC100502313	uncharacterized LOC100502313	0	0	0	0	2.1894569	0.002909	0	0	2.2112055	0.00183726
100502421	LOC100502421	uncharacterized LOC100502421	0	0	0	0	0	0	0	0	-2.240741	0.03021937
100502453	LOC100502453	uncharacterized LOC100502453	3.63802724	0.030234	0	0	0	0	0	0	0	0
100502455	LOC100502455	uncharacterized LOC100502455	0	0	0	0	0	0	-3.525473	0.0268603	0	0
100502500	LOC100502500	uncharacterized LOC100502500	0	0	0	0	-3.5027381	0.042946	0	0	0	0
100856921	LOC100856921	uncharacterized LOC100856921	0	0	0	0	3.1596638	0.036299	0	0	0	0
103625797	LOC103625797	uncharacterized LOC103625797	0	0	0	0	0	0	0	0	3.9319702	0.04219393
103625887	LOC103625887	uncharacterized LOC103625887	0	0	0	0	0	0	1.599239	0.0291378	0	0
103626186	LOC103626186	uncharacterized LOC103626186	0	0	2.6301899	0.03594	0	0	0	0	-4.020825	0.00260493
103626188	LOC103626188	uncharacterized LOC103626188	0	0	0	0	0	0	-3.585896	0.0432005	0	0

			Yuc x Ctr		Azo x Ctr		AzoYuc x Ctr		AzoYuc x Yuc		AzoYuc x Azo	
**Gene ID**	**Gene Symbol**	**Gene Description**	**Log2(FC)**	**pvalue**	**Log2(FC)**	**pvalue**	**Log2(FC)**	**pvalue**	**Log2(FC)**	**pvalue**	**Log2(FC)**	**pvalue**
103626226	LOC103626226	uncharacterized LOC103626226	0	0	0	0	1.8645859	0.038139	0	0	0	0
103626267	LOC103626267	uncharacterized LOC103626267	0	0	0	0	3.6718989	0.020877	0	0	0	0
103626314	LOC103626314	uncharacterized LOC103626314	0	0	0	0	4.1342948	0.006402	2.616693	0.0314501	0	0
103626342	LOC103626342	uncharacterized LOC103626342	0	0	-2.520352	0.03498	0	0	0	0	0	0
103626490	LOC103626490	uncharacterized LOC103626490	2.07451983	0.007357	0	0	0	0	-2.197587	0.0037796	0	0
103626595	LOC103626595	uncharacterized LOC103626595	0	0	0	0	2.7272749	0.016004	2.353348	0.0289378	0	0
103626607	LOC103626607	uncharacterized LOC103626607	-4.6644863	8.46E-05	-4.949446	3.4E-05	-3.4362757	0.001311	0	0	0	0
103626673	LOC103626673	uncharacterized LOC103626673	-4.9612486	0.010739	0	0	0	0	0	0	0	0
103626751	LOC103626751	uncharacterized LOC103626751	0	0	0	0	3.9280012	0.040315	5.31252	0.0055498	0	0
103626979	LOC103626979	uncharacterized LOC103626979	-3.5764854	0.018558	-3.177569	0.02253	0	0	0	0	0	0
103627150	LOC103627150	uncharacterized LOC103627150	0	0	0	0	0	0	5.155639	0.0197655	0	0
103627207	LOC103627207	uncharacterized LOC103627207	4.59150342	0.02028	0	0	0	0	0	0	0	0
103627564	LOC103627564	uncharacterized LOC103627564	-1.8676748	0.033834	0	0	0	0	0	0	0	0

			Yuc x Ctr		Azo x Ctr		AzoYuc x Ctr		AzoYuc x Yuc		AzoYuc x Azo	
**Gene ID**	**Gene Symbol**	**Gene Description**	**Log2(FC)**	**pvalue**	**Log2(FC)**	**pvalue**	**Log2(FC)**	**pvalue**	**Log2(FC)**	**pvalue**	**Log2(FC)**	**pvalue**
103627782	LOC103627782	uncharacterized LOC103627782	0	0	0	0	3.2832027	0.023709	0	0	0	0
103628086	LOC103628086	uncharacterized LOC103628086	0	0	-2.524582	0.02779	0	0	0	0	0	0
103628099	LOC103628099	uncharacterized LOC103628099	0	0	0	0	-3.5199908	0.003929	-2.483732	0.0459021	-2.615639	0.03347773
103628528	LOC103628528	uncharacterized LOC103628528	0	0	0	0	2.0395687	0.022384	2.568019	0.0044625	0	0
103628985	LOC103628985	uncharacterized LOC103628985	0	0	0	0	2.3979842	0.029768	0	0	0	0
103629117	LOC103629117	uncharacterized LOC103629117	0	0	0	0	1.643385	0.04161	1.797682	0.0238037	0	0
103629455	LOC103629455	uncharacterized LOC103629455	0	0	0	0	0	0	4.427505	0.008502	0	0
103629667	LOC103629667	uncharacterized LOC103629667	0	0	0	0	2.9627893	0.000749	2.700427	0.001635	2.6483745	0.00187567
103629968	LOC103629968	uncharacterized LOC103629968	-4.4906845	0.012889	0	0	-3.7851698	0.03607	0	0	0	0
103630054	LOC103630054	uncharacterized LOC103630054	-4.1156736	0.003471	-3.531242	0.0066	-3.7615724	0.004423	0	0	0	0
103630114	LOC103630114	uncharacterized LOC103630114	0	0	0	0	2.1732191	0.006739	2.219047	0.0053648	2.4839473	0.00185494
103630157	LOC103630157	uncharacterized LOC103630157	0	0	-3.907289	0.00446	-2.5310597	0.026402	-2.433541	0.0312561	0	0
103630187	LOC103630187	uncharacterized LOC103630187	0	0	-2.496249	0.04777	0	0	0	0	0	0

			Yuc x Ctr		Azo x Ctr		AzoYuc x Ctr		AzoYuc x Yuc		AzoYuc x Azo	
**Gene ID**	**Gene Symbol**	**Gene Description**	**Log2(FC)**	**pvalue**	**Log2(FC)**	**pvalue**	**Log2(FC)**	**pvalue**	**Log2(FC)**	**pvalue**	**Log2(FC)**	**pvalue**
103630270	LOC103630270	uncharacterized LOC103630270	0	0	0	0	1.6438503	0.036671	0	0	0	0
103630401	LOC103630401	uncharacterized LOC103630401	0	0	0	0	0	0	-2.704637	0.0306157	0	0
103630456	LOC103630456	uncharacterized LOC103630456	0	0	0	0	-1.6723607	0.009627	-1.681919	0.0080181	-1.697296	0.00702376
103630492	LOC103630492	uncharacterized LOC103630492	0	0	0	0	0	0	0	0	3.0167637	0.04519075
103630642	LOC103630642	uncharacterized LOC103630642	-3.6693108	0.01093	-3.304449	0.02042	0	0	0	0	0	0
103630787	LOC103630787	uncharacterized LOC103630787	0	0	0	0	0	0	0	0	2.4912257	0.04323153
103631148	LOC103631148	uncharacterized LOC103631148	0	0	-1.54827	0.03935	-1.9648256	0.009715	0	0	0	0
103631927	LOC103631927	uncharacterized LOC103631927	0	0	0	0	-2.0465272	0.004906	-1.767509	0.0147235	-1.574249	0.02981946
103631979	LOC103631979	uncharacterized LOC103631979	0	0	0	0	1.6054604	0.010588	1.620968	0.0086027	0	0
103632022	LOC103632022	uncharacterized LOC103632022	-2.7124562	0.007472	0	0	0	0	0	0	0	0
103632037	LOC103632037	uncharacterized LOC103632037	4.31570851	0.006831	0	0	0	0	-3.64519	0.0130976	0	0
103632202	LOC103632202	uncharacterized LOC103632202	0	0	-1.748864	0.04791	0	0	0	0	0	0
103632216	LOC103632216	uncharacterized LOC103632216	-2.5795894	0.043564	0	0	0	0	0	0	0	0

			Yuc x Ctr		Azo x Ctr		AzoYuc x Ctr		AzoYuc x Yuc		AzoYuc x Azo	
**Gene ID**	**Gene Symbol**	**Gene Description**	**Log2(FC)**	**pvalue**	**Log2(FC)**	**pvalue**	**Log2(FC)**	**pvalue**	**Log2(FC)**	**pvalue**	**Log2(FC)**	**pvalue**
103632486	LOC103632486	uncharacterized LOC103632486	0	0	0	0	0	0	2.896277	0.0487835	0	0
103632573	LOC103632573	uncharacterized LOC103632573	0	0	0	0	4.1360194	0.003672	3.541082	0.0059263	4.3058749	0.00141635
103632575	LOC103632575	uncharacterized LOC103632575	0	0	-1.960156	0.04774	-2.3631569	0.021577	0	0	0	0
103632729	LOC103632729	uncharacterized LOC103632729	0	0	0	0	0	0	0	0	3.2415186	0.03722303
103632806	LOC103632806	uncharacterized LOC103632806	0	0	-1.503957	0.00837	-1.7685831	0.002107	0	0	0	0
103632813	LOC103632813	uncharacterized LOC103632813	0	0	0	0	0	0	0	0	1.807285	0.04131552
103633165	LOC103633165	uncharacterized LOC103633165	2.2000288	0.023445	0	0	0	0	-3.814439	0.0001944	0	0
103633290	LOC103633290	uncharacterized GPI-anchored protein At4g28100	-2.3673827	0.017256	0	0	0	0	0	0	0	0
103633395	LOC103633395	uncharacterized LOC103633395	-3.3812613	0.036698	0	0	0	0	3.670418	0.0202385	0	0
103634133	LOC103634133	uncharacterized LOC103634133	0	0	0	0	0	0	3.834592	0.0075374	3.1333139	0.01699118
103634163	LOC103634163	uncharacterized LOC103634163	0	0	0	0	-2.0661807	0.00113	-2.315566	0.000192	0	0
103634362	LOC103634362	uncharacterized LOC103634362	0	0	0	0	5.0079816	0.006949	6.392501	0.00057	3.1821271	0.04050774
103634774	LOC103634774	uncharacterized LOC103634774	0	0	0	0	-1.9441583	0.038435	0	0	0	0

			Yuc x Ctr		Azo x Ctr		AzoYuc x Ctr		AzoYuc x Yuc		AzoYuc x Azo	
**Gene ID**	**Gene Symbol**	**Gene Description**	**Log2(FC)**	**pvalue**	**Log2(FC)**	**pvalue**	**Log2(FC)**	**pvalue**	**Log2(FC)**	**pvalue**	**Log2(FC)**	**pvalue**
103634819	LOC103634819	uncharacterized LOC103634819	-2.6044598	0.005275	-3.011769	0.00159	-1.9914332	0.019912	0	0	0	0
103635036	LOC103635036	uncharacterized LOC103635036	0	0	0	0	2.3968726	0.031704	0	0	0	0
103635054	LOC103635054	uncharacterized LOC103635054	0	0	0	0	3.4427292	0.043278	3.576305	0.0302142	3.6016583	0.02607275
103635080	LOC103635080	uncharacterized LOC103635080	0	0	0	0	0	0	-2.337352	0.046541	-2.544301	0.02828507
103635104	LOC103635104	uncharacterized LOC103635104	2.77402668	0.014789	0	0	2.6572013	0.019293	0	0	0	0
103635293	LOC103635293	uncharacterized LOC103635293	3.51975629	0.041151	0	0	0	0	0	0	0	0
103635799	LOC103635799	uncharacterized LOC103635799	0	0	0	0	0	0	0	0	4.6921221	0.04734114
103635851	LOC103635851	uncharacterized LOC103635851	0	0	0	0	0	0	0	0	2.1253508	0.01870384
103635907	LOC103635907	uncharacterized LOC103635907	0	0	0	0	-1.8680477	0.019944	0	0	0	0
103636109	LOC103636109	uncharacterized LOC103636109	0	0	0	0	0	0	0	0	-4.795074	0.01685662
103636147	LOC103636147	uncharacterized protein At4g18257	0	0	0	0	0	0	0	0	5.3841908	0.04508165
103636150	LOC103636150	uncharacterized LOC103636150	-3.6460923	0.033223	0	0	0	0	0	0	0	0
103636161	LOC103636161	uncharacterized LOC103636161	0	0	0	0	0	0	0	0	4.9258979	0.04698741

			Yuc x Ctr		Azo x Ctr		AzoYuc x Ctr		AzoYuc x Yuc		AzoYuc x Azo	
**Gene ID**	**Gene Symbol**	**Gene Description**	**Log2(FC)**	**pvalue**	**Log2(FC)**	**pvalue**	**Log2(FC)**	**pvalue**	**Log2(FC)**	**pvalue**	**Log2(FC)**	**pvalue**
103636287	LOC103636287	uncharacterized LOC103636287	1.96213298	0.020226	0	0	0	0	0	0	0	0
103636932	LOC103636932	uncharacterized LOC103636932	0	0	0	0	0	0	3.311454	0.0328813	3.0286339	0.04154572
103637263	LOC103637263	uncharacterized LOC103637263	-1.7299961	0.002276	0	0	0	0	0	0	0	0
103637556	LOC103637556	uncharacterized protein DDB_G0271670	0	0	0	0	2.8750221	0.010735	0	0	2.3247516	0.02603857
103637645	LOC103637645	uncharacterized LOC103637645	-4.2105922	0.012158	0	0	0	0	5.137293	0.0015578	0	0
103638453	LOC103638453	uncharacterized LOC103638453	0	0	0	0	0	0	-3.392282	0.0342084	0	0
103638694	LOC103638694	uncharacterized LOC103638694	0	0	-4.466544	0.01611	0	0	0	0	0	0
103638710	LOC103638710	uncharacterized LOC103638710	0	0	0	0	0	0	2.845276	0.0146168	0	0
103638978	LOC103638978	uncharacterized LOC103638978	0	0	0	0	0	0	0	0	-1.59068	0.03985428
103639027	LOC103639027	uncharacterized protein C630.12	0	0	0	0	0	0	2.008001	0.0476248	0	0
103639178	LOC103639178	uncharacterized LOC103639178	0	0	0	0	2.9908945	0.041869	0	0	0	0
103639197	LOC103639197	uncharacterized LOC103639197	0	0	0	0	0	0	2.118656	0.0054905	0	0
103639235	LOC103639235	uncharacterized LOC103639235	0	0	0	0	-3.3941606	0.044662	0	0	0	0

			Yuc x Ctr		Azo x Ctr		AzoYuc x Ctr		AzoYuc x Yuc		AzoYuc x Azo	
**Gene ID**	**Gene Symbol**	**Gene Description**	**Log2(FC)**	**pvalue**	**Log2(FC)**	**pvalue**	**Log2(FC)**	**pvalue**	**Log2(FC)**	**pvalue**	**Log2(FC)**	**pvalue**
103639247	LOC103639247	uncharacterized LOC103639247	7.562166	0.011149	0	0	0	0	-6.524635	0.0218098	0	0
103639764	LOC103639764	uncharacterized LOC103639764	0	0	0	0	0	0	3.922766	0.0343556	3.8731759	0.03533837
103639870	LOC103639870	uncharacterized LOC103639870	-2.5379372	0.001779	-2.610379	0.00127	-2.9136239	0.000348	0	0	0	0
103639888	LOC103639888	uncharacterized LOC103639888	0	0	0	0	2.5986554	0.021516	0	0	2.1968008	0.04590248
103639891	LOC103639891	uncharacterized protein	0	0	0	0	-1.7068484	0.013249	0	0	0	0
103639967	LOC103639967	uncharacterized LOC103639967	-5.7995759	0.041997	0	0	0	0	0	0	0	0
103640141	LOC103640141	uncharacterized LOC103640141	0	0	0	0	0	0	0	0	3.5531204	0.04443354
103640263	LOC103640263	uncharacterized LOC103640263	0	0	0	0	3.2690645	0.036343	0	0	0	0
103640528	LOC103640528	uncharacterized LOC103640528	0	0	0	0	1.8777976	0.028443	0	0	0	0
103640565	LOC103640565	uncharacterized LOC103640565	0	0	0	0	5.4002411	0.012369	0	0	5.087124	0.01845239
103640704	LOC103640704	uncharacterized LOC103640704	-2.2324493	0.003577	0	0	-1.8133495	0.014962	0	0	0	0
103640844	LOC103640844	uncharacterized LOC103640844	-4.0408492	0.037256	0	0	0	0	0	0	0	0
103641148	LOC103641148	uncharacterized LOC103641148	0	0	0	0	1.7751772	0.002979	1.763046	0.0029324	1.6896258	0.00420286

			Yuc x Ctr		Azo x Ctr		AzoYuc x Ctr		AzoYuc x Yuc		AzoYuc x Azo	
**Gene ID**	**Gene Symbol**	**Gene Description**	**Log2(FC)**	**pvalue**	**Log2(FC)**	**pvalue**	**Log2(FC)**	**pvalue**	**Log2(FC)**	**pvalue**	**Log2(FC)**	**pvalue**
103641300	LOC103641300	uncharacterized LOC103641300	0	0	0	0	0	0	-6.065615	0.0307236	0	0
103641455	LOC103641455	uncharacterized protein	0	0	0	0	1.5790487	0.019085	0	0	0	0
103641521	LOC103641521	uncharacterized LOC103641521	0	0	-4.445756	0.04946	0	0	0	0	0	0
103641878	LOC103641878	uncharacterized LOC103641878	0	0	0	0	0	0	3.272141	0.0240315	0	0
103641914	LOC103641914	uncharacterized LOC103641914	-2.2986223	0.027706	0	0	0	0	0	0	0	0
103642028	LOC103642028	uncharacterized LOC103642028	0	0	0	0	0	0	0	0	4.2565766	0.04755527
103642063	LOC103642063	uncharacterized LOC103642063	0	0	-1.713831	0.02353	-1.8774919	0.014331	-1.636241	0.0315737	0	0
103642102	LOC103642102	uncharacterized LOC103642102	0	0	-1.778465	0.01517	-1.8638812	0.011275	0	0	0	0
103642199	LOC103642199	uncharacterized LOC103642199	0	0	0	0	0	0	3.609446	0.0419553	5.1582631	0.00779958
103642371	LOC103642371	uncharacterized LOC103642371	0	0	0	0	0	0	-1.976835	0.0307416	0	0
103642641	LOC103642641	uncharacterized LOC103642641	0	0	0	0	0	0	-2.04004	0.0285899	0	0
103642963	LOC103642963	uncharacterized LOC103642963	-2.1404342	0.033037	0	0	0	0	0	0	0	0
103643021	LOC103643021	uncharacterized LOC103643021	-1.7678963	0.031216	-1.707006	0.03544	0	0	0	0	0	0

			Yuc x Ctr		Azo x Ctr		AzoYuc x Ctr		AzoYuc x Yuc		AzoYuc x Azo	
**Gene ID**	**Gene Symbol**	**Gene Description**	**Log2(FC)**	**pvalue**	**Log2(FC)**	**pvalue**	**Log2(FC)**	**pvalue**	**Log2(FC)**	**pvalue**	**Log2(FC)**	**pvalue**
103643056	LOC103643056	uncharacterized LOC103643056	0	0	0	0	1.6782644	0.010797	1.687991	0.0092602	1.8654311	0.00401195
103643065	LOC103643065	uncharacterized LOC103643065	0	0	0	0	0	0	1.679808	0.0433197	2.4025119	0.0049833
103643290	LOC103643290	uncharacterized LOC103643290	3.36822671	0.001646	0	0	0	0	-2.849577	0.0053576	0	0
103643416	LOC103643416	uncharacterized LOC103643416	0	0	0	0	-1.8259594	0.035523	0	0	0	0
103643535	LOC103643535	uncharacterized LOC103643535	0	0	0	0	-1.8960914	0.034611	0	0	0	0
103643652	LOC103643652	uncharacterized protein PFB0765w	4.13158428	0.003096	0	0	0	0	0	0	0	0
103643846	LOC103643846	uncharacterized LOC103643846	-3.2322436	0.036656	0	0	0	0	0	0	0	0
103644017	LOC103644017	uncharacterized LOC103644017	0	0	0	0	-1.5985307	0.016172	0	0	0	0
103644253	LOC103644253	uncharacterized LOC103644253	-5.458779	4.28E-05	-8.771884	1.1E-07	-7.8404359	2E-06	0	0	0	0
103644320	LOC103644320	uncharacterized LOC103644320	7.62294481	0.007468	0	0	0	0	-5.34487	0.0468062	0	0
103644343	LOC103644343	uncharacterized LOC103644343	-3.961011	0.025058	-3.305842	0.04737	-5.5409708	0.004146	0	0	0	0
103644532	LOC103644532	uncharacterized LOC103644532	0	0	0	0	4.4315399	0.005517	0	0	0	0
103645040	LOC103645040	uncharacterized LOC103645040	0	0	0	0	0	0	0	0	3.1824124	0.02835665

			Yuc x Ctr		Azo x Ctr		AzoYuc x Ctr		AzoYuc x Yuc		AzoYuc x Azo	
**Gene ID**	**Gene Symbol**	**Gene Description**	**Log2(FC)**	**pvalue**	**Log2(FC)**	**pvalue**	**Log2(FC)**	**pvalue**	**Log2(FC)**	**pvalue**	**Log2(FC)**	**pvalue**
103645216	LOC103645216	uncharacterized LOC103645216	0	0	-2.731989	0.03498	-2.6367554	0.039987	0	0	0	0
103645334	LOC103645334	uncharacterized LOC103645334	0	0	0	0	0	0	4.188852	0.0264172	0	0
103645597	LOC103645597	uncharacterized LOC103645597	-5.1278382	0.004983	-3.903287	0.0167	-6.3459125	0.00051	0	0	0	0
103645607	LOC103645607	uncharacterized LOC103645607	0	0	0	0	0	0	-1.686815	0.0237948	0	0
103645745	LOC103645745	uncharacterized LOC103645745	-3.8340074	0.034064	0	0	0	0	0	0	0	0
103645891	LOC103645891	uncharacterized LOC103645891	0	0	0	0	0	0	3.947696	0.0424335	0	0
103645907	LOC103645907	uncharacterized LOC103645907	0	0	0	0	-3.384372	0.037469	-3.45155	0.0310388	-3.529511	0.02630957
103646102	LOC103646102	uncharacterized LOC103646102	0	0	0	0	0	0	3.439435	0.0130143	0	0
103646241	LOC103646241	uncharacterized LOC103646241	-3.4180379	0.023397	0	0	0	0	0	0	0	0
103646371	LOC103646371	uncharacterized LOC103646371	0	0	0	0	0	0	3.565058	0.002927	2.3224755	0.03037126
103646409	LOC103646409	uncharacterized LOC103646409	-4.1106205	0.021708	-3.255	0.04913	-3.9699455	0.021712	0	0	0	0
103646480	LOC103646480	uncharacterized LOC103646480	0	0	0	0	4.1254783	0.023284	3.213984	0.049996	0	0
103646825	LOC103646825	uncharacterized LOC103646825	-5.9713028	1.53E-05	-6.381008	4.6E-06	-5.5920777	7.33E-06	0	0	0	0

			Yuc x Ctr		Azo x Ctr		AzoYuc x Ctr		AzoYuc x Yuc		AzoYuc x Azo	
**Gene ID**	**Gene Symbol**	**Gene Description**	**Log2(FC)**	**pvalue**	**Log2(FC)**	**pvalue**	**Log2(FC)**	**pvalue**	**Log2(FC)**	**pvalue**	**Log2(FC)**	**pvalue**
103647051	LOC103647051	uncharacterized LOC103647051	0	0	0	0	2.5147979	0.022528	3.143546	0.0048066	2.4703537	0.02135195
103647083	LOC103647083	uncharacterized LOC103647083	0	0	-3.637792	0.02446	0	0	0	0	0	0
103647366	LOC103647366	uncharacterized LOC103647366	0	0	0	0	-4.0850412	0.037062	0	0	0	0
103647383	LOC103647383	uncharacterized LOC103647383	0	0	0	0	0	0	1.895598	0.0217486	0	0
103647449	LOC103647449	uncharacterized LOC103647449	-1.7335224	0.007034	0	0	0	0	1.848105	0.0032032	0	0
103647463	LOC103647463	uncharacterized LOC103647463	-2.8159784	0.04073	0	0	0	0	2.667209	0.0485943	0	0
103647542	LOC103647542	uncharacterized LOC103647542	0	0	-2.296554	0.01122	0	0	0	0	0	0
103647707	LOC103647707	uncharacterized LOC103647707	0	0	0	0	2.8443994	0.025681	0	0	0	0
103647724	LOC103647724	uncharacterized LOC103647724	0	0	0	0	0	0	0	0	-1.643889	0.03763929
103647763	LOC103647763	uncharacterized LOC103647763	0	0	0	0	0	0	3.61636	0.0116397	4.5887074	0.00288842
103647937	LOC103647937	uncharacterized LOC103647937	4.21628508	0.008943	3.9860612	0.01352	3.3187333	0.042889	0	0	0	0
103647941	LOC103647941	uncharacterized LOC103647941	0	0	0	0	0	0	1.789542	0.0444584	0	0
103648014	LOC103648014	uncharacterized LOC103648014	-4.0458684	0.000353	-2.912691	0.00691	-2.7518171	0.010318	0	0	0	0

			Yuc x Ctr		Azo x Ctr		AzoYuc x Ctr		AzoYuc x Yuc		AzoYuc x Azo	
**Gene ID**	**Gene Symbol**	**Gene Description**	**Log2(FC)**	**pvalue**	**Log2(FC)**	**pvalue**	**Log2(FC)**	**pvalue**	**Log2(FC)**	**pvalue**	**Log2(FC)**	**pvalue**
103648023	LOC103648023	uncharacterized LOC103648023	-4.6124905	0.027134	0	0	0	0	0	0	0	0
103648084	LOC103648084	uncharacterized LOC103648084	0	0	0	0	1.6153944	0.045261	0	0	1.7527944	0.02528521
103648144	LOC103648144	uncharacterized LOC103648144	0	0	4.2234646	0.01797	3.7788839	0.03538	0	0	0	0
103648231	LOC103648231	uncharacterized LOC103648231	0	0	0	0	3.0690775	0.004585	3.473713	0.0013236	3.2518841	0.00210086
103648255	LOC103648255	uncharacterized LOC103648255	0	0	0	0	0	0	1.82304	0.0139243	0	0
103648292	LOC103648292	uncharacterized LOC103648292	0	0	-6.433348	0.00136	-4.7440999	0.009605	0	0	0	0
103648516	LOC103648516	uncharacterized LOC103648516	0	0	0	0	0	0	0	0	4.4788761	0.01958568
103648694	LOC103648694	uncharacterized LOC103648694	0	0	-2.169471	0.02827	0	0	0	0	0	0
103648964	LOC103648964	uncharacterized LOC103648964	0	0	0	0	3.3616563	0.039794	3.094994	0.0419963	3.2846338	0.03047899
103649041	LOC103649041	uncharacterized LOC103649041	0	0	4.6529742	0.00674	0	0	0	0	0	0
103649177	LOC103649177	uncharacterized LOC103649177	0	0	0	0	-2.7066333	0.00942	0	0	0	0
103649203	LOC103649203	uncharacterized LOC103649203	0	0	0	0	3.7418612	0.04454	0	0	0	0
103649645	LOC103649645	uncharacterized LOC103649645	0	0	0	0	0	0	1.96693	0.0423918	0	0

			Yuc x Ctr		Azo x Ctr		AzoYuc x Ctr		AzoYuc x Yuc		AzoYuc x Azo	
**Gene ID**	**Gene Symbol**	**Gene Description**	**Log2(FC)**	**pvalue**	**Log2(FC)**	**pvalue**	**Log2(FC)**	**pvalue**	**Log2(FC)**	**pvalue**	**Log2(FC)**	**pvalue**
103650223	LOC103650223	uncharacterized LOC103650223	0	0	0	0	2.8785969	0.00745	3.826078	0.0005247	4.0553859	0.00024184
103650242	LOC103650242	uncharacterized LOC103650242	0	0	0	0	-3.921793	0.045652	0	0	0	0
103650294	LOC103650294	uncharacterized LOC103650294	0	0	0	0	0	0	0	0	2.0413068	0.02954487
103650399	LOC103650399	uncharacterized LOC103650399	-3.4259845	0.043128	-4.151307	0.01521	-4.0065551	0.018648	0	0	0	0
103650412	LOC103650412	uncharacterized LOC103650412	0	0	-3.553246	0.02955	0	0	0	0	0	0
103650747	LOC103650747	uncharacterized LOC103650747	0	0	0	0	5.5139802	0.002495	0	0	3.9376567	0.01749372
103650806	LOC103650806	uncharacterized LOC103650806	0	0	0	0	0	0	0	0	2.0298627	0.0143669
103650845	LOC103650845	uncharacterized protein At4g15970	-2.9821828	0.039621	-3.668274	0.01365	-4.3723892	0.005118	0	0	0	0
103650972	LOC103650972	uncharacterized LOC103650972	0	0	0	0	-4.6267379	0.011471	-4.579494	0.011638	0	0
103651194	LOC103651194	uncharacterized LOC103651194	0	0	0	0	-3.3623605	0.047448	0	0	0	0
103651445	LOC103651445	uncharacterized LOC103651445	0	0	-1.533436	0.03423	0	0	0	0	0	0
103651769	LOC103651769	uncharacterized LOC103651769	0	0	0	0	4.1435676	0.000287	2.990885	0.0041688	2.7726323	0.00715099
103652167	LOC103652167	uncharacterized LOC103652167	0	0	0	0	0	0	0	0	3.0037782	0.04910159

			Yuc x Ctr		Azo x Ctr		AzoYuc x Ctr		AzoYuc x Yuc		AzoYuc x Azo	
**Gene ID**	**Gene Symbol**	**Gene Description**	**Log2(FC)**	**pvalue**	**Log2(FC)**	**pvalue**	**Log2(FC)**	**pvalue**	**Log2(FC)**	**pvalue**	**Log2(FC)**	**pvalue**
103652297	LOC103652297	uncharacterized LOC103652297	0	0	-4.635756	0.01297	0	0	0	0	4.3830653	0.0178091
103652852	LOC103652852	uncharacterized LOC103652852	0	0	-5.154012	0.03714	0	0	0	0	0	0
103652865	LOC103652865	uncharacterized LOC103652865	-6.3931987	0.000157	-3.537805	0.01074	-5.6876839	0.00077	0	0	0	0
103652964	LOC103652964	uncharacterized LOC103652964	0	0	0	0	0	0	1.674307	0.017826	1.5426075	0.02854123
103653042	LOC103653042	uncharacterized LOC103653042	-2.3959663	0.006632	-1.892224	0.02638	0	0	0	0	0	0
103653151	LOC103653151	uncharacterized LOC103653151	0	0	0	0	2.5811686	0.003679	2.179495	0.0114567	2.6572006	0.00224944
103653374	LOC103653374	uncharacterized LOC103653374	0	0	0	0	0	0	0	0	3.0770539	0.01020744
103653467	LOC103653467	uncharacterized LOC103653467	0	0	3.7816632	0.04629	0	0	0	0	0	0
103653535	LOC103653535	uncharacterized LOC103653535	0	0	0	0	0	0	1.662173	0.0221799	0	0
103653616	LOC103653616	uncharacterized LOC103653616	0	0	-2.062729	0.03898	-2.3079309	0.022768	0	0	0	0
103653665	LOC103653665	uncharacterized LOC103653665	2.56527301	0.009999	2.5040462	0.01165	3.2273396	0.000984	0	0	0	0
103653714	LOC103653714	uncharacterized LOC103653714	3.45054579	0.049007	0	0	4.1193113	0.016474	0	0	0	0
103653829	LOC103653829	uncharacterized protein At1g65710	0	0	0	0	0	0	3.07078	0.0093808	3.2684104	0.0056919

			Yuc x Ctr		Azo x Ctr		AzoYuc x Ctr		AzoYuc x Yuc		AzoYuc x Azo	
**Gene ID**	**Gene Symbol**	**Gene Description**	**Log2(FC)**	**pvalue**	**Log2(FC)**	**pvalue**	**Log2(FC)**	**pvalue**	**Log2(FC)**	**pvalue**	**Log2(FC)**	**pvalue**
103653832	LOC103653832	uncharacterized LOC103653832	-1.8317091	0.025107	0	0	0	0	0	0	0	0
103653882	LOC103653882	uncharacterized LOC103653882	-1.8828626	0.028164	-2.556919	0.00404	-2.7419693	0.002374	0	0	0	0
103654009	LOC103654009	uncharacterized LOC103654009	0	0	0	0	0	0	0	0	1.81732	0.04347832
103654201	LOC103654201	uncharacterized LOC103654201	0	0	0	0	0	0	-3.513146	0.0053627	0	0
103654253	LOC103654253	uncharacterized LOC103654253	0	0	-3.012776	0.00075	-3.1390692	0.000501	-1.939108	0.0348011	0	0
103654384	LOC103654384	uncharacterized LOC103654384	0	0	-1.843843	0.02891	0	0	0	0	0	0
103654899	LOC103654899	uncharacterized LOC103654899	0	0	0	0	0	0	0	0	3.299101	0.03567094
103654908	LOC103654908	uncharacterized LOC103654908	-4.3017483	0.022063	-4.527681	0.01597	-4.5580281	0.015281	0	0	0	0
103655263	LOC103655263	uncharacterized LOC103655263	-3.0231273	0.000155	-2.62781	0.00071	-2.0025938	0.008342	0	0	0	0
103655353	LOC103655353	uncharacterized LOC103655353	0	0	0	0	0	0	1.577324	0.0407502	0	0
103655800	LOC103655800	uncharacterized LOC103655800	0	0	0	0	0	0	2.000836	0.0032033	0	0
103655972	LOC103655972	uncharacterized LOC103655972	2.57911693	0.021349	0	0	0	0	0	0	0	0
103656015	LOC103656015	uncharacterized LOC103656015	0	0	0	0	0	0	0	0	-3.666822	0.04955653

			Yuc x Ctr		Azo x Ctr		AzoYuc x Ctr		AzoYuc x Yuc		AzoYuc x Azo	
**Gene ID**	**Gene Symbol**	**Gene Description**	**Log2(FC)**	**pvalue**	**Log2(FC)**	**pvalue**	**Log2(FC)**	**pvalue**	**Log2(FC)**	**pvalue**	**Log2(FC)**	**pvalue**
103656032	LOC103656032	uncharacterized LOC103656032	0	0	0	0	3.7458755	0.047063	0	0	0	0
107275237	LOC107275237	uncharacterized LOC107275237	0	0	0	0	0	0	0	0	-1.50559	0.01741678
107546776	LOC107546776	uncharacterized LOC107546776	0	0	0	0	3.2948727	0.000285	0	0	2.1840255	0.00952473
109461487	LOC109461487	uncharacterized LOC109461487	0	0	0	0	0	0	0	0	-1.927291	0.03741609
109623449	LOC109623449	uncharacterized LOC109623449	0	0	1.6226523	0.04819	0	0	0	0	0	0
109851609	LOC109851609	uncharacterized LOC109851609	0	0	0	0	0	0	4.410696	0.0364176	0	0
109939171	LOC109939171	uncharacterized LOC109939171	0	0	0	0	-1.8827568	0.040656	0	0	-1.953434	0.02949984
109939196	LOC109939196	uncharacterized LOC109939196	5.12588439	0.00026	5.4226621	0.0001	4.2405592	0.002806	0	0	0	0
109939475	LOC109939475	uncharacterized LOC109939475	0	0	-2.148913	0.00127	-1.6746305	0.01021	0	0	0	0
109939910	LOC109939910	uncharacterized LOC109939910	0	0	-4.544274	0.01153	0	0	0	0	0	0
109939916	LOC109939916	uncharacterized vacuolar membrane protein YML018C	-4.1591204	0.019896	-3.564793	0.03345	0	0	0	0	0	0
109939937	LOC109939937	uncharacterized LOC109939937	-4.3590265	0.000599	-4.255261	0.00053	-4.9471431	0.000181	0	0	0	0
109939951	LOC109939951	uncharacterized LOC109939951	0	0	0	0	0	0	5.587941	0.005874	4.8520888	0.01675794

			Yuc x Ctr		Azo x Ctr		AzoYuc x Ctr		AzoYuc x Yuc		AzoYuc x Azo	
**Gene ID**	**Gene Symbol**	**Gene Description**	**Log2(FC)**	**pvalue**	**Log2(FC)**	**pvalue**	**Log2(FC)**	**pvalue**	**Log2(FC)**	**pvalue**	**Log2(FC)**	**pvalue**
109939988	LOC109939988	uncharacterized LOC109939988	0	0	0	0	0	0	0	0	3.2405897	0.02753002
109940165	LOC109940165	uncharacterized LOC109940165	-2.2387345	0.040569	0	0	0	0	0	0	0	0
109940180	LOC109940180	uncharacterized LOC109940180	-4.0211591	0.029405	0	0	0	0	0	0	0	0
109940211	LOC109940211	uncharacterized LOC109940211	-4.4445271	0.017397	-4.338107	0.01672	0	0	0	0	0	0
109940310	LOC109940310	uncharacterized LOC109940310	0	0	0	0	3.3137425	0.036033	0	0	0	0
109940311	LOC109940311	uncharacterized LOC109940311	-3.259625	0.02237	0	0	0	0	0	0	0	0
109940494	LOC109940494	uncharacterized LOC109940494	-4.5268468	0.004509	-5.807402	0.0008	-4.2442517	0.004698	0	0	0	0
109940843	LOC109940843	uncharacterized LOC109940843	0	0	0	0	0	0	2.261029	0.0389687	0	0
109940881	LOC109940881	uncharacterized LOC109940881	0	0	0	0	0	0	3.145767	0.0420307	0	0
109940894	LOC109940894	uncharacterized LOC109940894	0	0	0	0	0	0	-3.987705	0.0282925	0	0
109940945	LOC109940945	uncharacterized LOC109940945	0	0	-1.585149	0.01208	-2.0149465	0.001812	-1.807594	0.0049172	0	0
109941468	LOC109941468	uncharacterized LOC109941468	0	0	0	0	5.2171159	0.011662	5.63985	0.0063993	0	0
109941508	LOC109941508	uncharacterized LOC109941508	4.68962051	0.017679	0	0	0	0	0	0	0	0

			Yuc x Ctr		Azo x Ctr		AzoYuc x Ctr		AzoYuc x Yuc		AzoYuc x Azo	
**Gene ID**	**Gene Symbol**	**Gene Description**	**Log2(FC)**	**pvalue**	**Log2(FC)**	**pvalue**	**Log2(FC)**	**pvalue**	**Log2(FC)**	**pvalue**	**Log2(FC)**	**pvalue**
109941518	LOC109941518	uncharacterized LOC109941518	-3.9099075	0.01803	0	0	0	0	0	0	0	0
109941537	LOC109941537	uncharacterized LOC109941537	0	0	0	0	0	0	3.359844	0.021842	0	0
109941578	LOC109941578	uncharacterized LOC109941578	0	0	0	0	-3.6002608	0.040036	0	0	0	0
109941614	LOC109941614	uncharacterized LOC109941614	0	0	0	0	0	0	3.120639	0.0348767	0	0
109941808	LOC109941808	uncharacterized LOC109941808	0	0	4.7269538	0.00706	0	0	0	0	0	0
109941825	LOC109941825	uncharacterized LOC109941825	-1.5310965	0.007908	0	0	0	0	0	0	0	0
109941899	LOC109941899	uncharacterized LOC109941899	-2.1500631	0.016384	0	0	0	0	2.208437	0.0118638	0	0
109941936	LOC109941936	uncharacterized LOC109941936	-5.8254087	0.002249	-5.089547	0.0076	0	0	0	0	0	0
109941998	LOC109941998	uncharacterized LOC109941998	0	0	0	0	0	0	-1.988693	0.0391302	0	0
109942217	LOC109942217	uncharacterized LOC109942217	0	0	0	0	-1.8274437	0.00334	-2.065347	0.0007074	-1.788422	0.00343157
109942229	LOC109942229	uncharacterized LOC109942229	0	0	0	0	0	0	2.833454	0.0432009	0	0
109942265	LOC109942265	uncharacterized LOC109942265	3.24063034	0.044249	0	0	0	0	0	0	0	0
109942320	LOC109942320	uncharacterized LOC109942320	0	0	0	0	-3.3948822	0.028997	0	0	0	0

			Yuc x Ctr		Azo x Ctr		AzoYuc x Ctr		AzoYuc x Yuc		AzoYuc x Azo	
**Gene ID**	**Gene Symbol**	**Gene Description**	**Log2(FC)**	**pvalue**	**Log2(FC)**	**pvalue**	**Log2(FC)**	**pvalue**	**Log2(FC)**	**pvalue**	**Log2(FC)**	**pvalue**
109942399	LOC109942399	uncharacterized LOC109942399	0	0	0	0	1.6487975	0.011788	1.543523	0.0171784	0	0
109942740	LOC109942740	uncharacterized LOC109942740	0	0	0	0	0	0	4.633939	0.0202874	4.2882097	0.02815518
109942935	LOC109942935	uncharacterized LOC109942935	0	0	0	0	0	0	0	0	2.9253836	0.04051003
109942952	LOC109942952	uncharacterized LOC109942952	-2.5232092	0.027551	-2.912144	0.01269	-2.8660526	0.013354	0	0	0	0
109943029	LOC109943029	uncharacterized LOC109943029	0	0	0	0	0	0	1.660815	0.0076404	0	0
109943038	LOC109943038	uncharacterized LOC109943038	0	0	-2.224517	0.02025	0	0	0	0	0	0
109943062	LOC109943062	uncharacterized LOC109943062	0	0	0	0	5.4479154	0.011166	4.111934	0.0480131	5.1347983	0.01677444
109943193	LOC109943193	uncharacterized LOC109943193	0	0	0	0	0	0	0	0	3.3156203	0.01193598
109943262	LOC109943262	uncharacterized LOC109943262	0	0	0	0	0	0	0	0	4.0448219	0.04764724
109943514	LOC109943514	uncharacterized LOC109943514	0	0	0	0	0	0	2.238219	0.0396385	0	0
109943652	LOC109943652	uncharacterized LOC109943652	0	0	0	0	-3.9991856	0.034175	-4.021399	0.0307853	-4.034969	0.02932727
109943679	LOC109943679	uncharacterized LOC109943679	0	0	0	0	-1.6680956	0.002928	-1.517987	0.0067105	0	0
109943730	LOC109943730	uncharacterized LOC109943730	0	0	0	0	-4.25505	0.039441	0	0	0	0

			Yuc x Ctr		Azo x Ctr		AzoYuc x Ctr		AzoYuc x Yuc		AzoYuc x Azo	
**Gene ID**	**Gene Symbol**	**Gene Description**	**Log2(FC)**	**pvalue**	**Log2(FC)**	**pvalue**	**Log2(FC)**	**pvalue**	**Log2(FC)**	**pvalue**	**Log2(FC)**	**pvalue**
109943740	LOC109943740	uncharacterized LOC109943740	0	0	0	0	3.5223145	0.016316	0	0	0	0
109943773	LOC109943773	uncharacterized LOC109943773	0	0	-2.119197	0.01106	-1.6121744	0.043907	0	0	0	0
109944009	LOC109944009	uncharacterized LOC109944009	0	0	-5.347181	2.1E-25	-6.2741548	3.43E-32	-4.829158	1.125E-19	0	0
109944010	LOC109944010	uncharacterized LOC109944010	0	0	0	0	3.3944894	0.020325	3.706554	0.0097662	0	0
109944015	LOC109944015	uncharacterized LOC109944015	0	0	0	0	0	0	0	0	-4.098701	0.04763006
109944048	LOC109944048	uncharacterized LOC109944048	0	0	0	0	0	0	2.008668	0.03721	0	0
109944084	LOC109944084	uncharacterized LOC109944084	0	0	0	0	0	0	0	0	4.265196	0.04945694
109944278	LOC109944278	uncharacterized LOC109944278	-2.012774	0.026653	0	0	0	0	0	0	0	0
109944416	LOC109944416	uncharacterized LOC109944416	0	0	0	0	-1.962922	0.033641	-1.832583	0.0447848	0	0
109944790	LOC109944790	uncharacterized LOC109944790	0	0	-3.244798	0.00318	-2.6753568	0.009803	0	0	0	0
109944807	LOC109944807	uncharacterized LOC109944807	3.21295742	0.033286	4.4231061	0.00222	4.4204799	0.002211	0	0	0	0
109944859	LOC109944859	uncharacterized LOC109944859	0	0	0	0	0	0	5.205551	0.0470621	0	0
109944884	LOC109944884	uncharacterized LOC109944884	0	0	0	0	0	0	4.846635	0.026089	5.0725646	0.01988031

			Yuc x Ctr		Azo x Ctr		AzoYuc x Ctr		AzoYuc x Yuc		AzoYuc x Azo	
**Gene ID**	**Gene Symbol**	**Gene Description**	**Log2(FC)**	**pvalue**	**Log2(FC)**	**pvalue**	**Log2(FC)**	**pvalue**	**Log2(FC)**	**pvalue**	**Log2(FC)**	**pvalue**
109945043	LOC109945043	uncharacterized LOC109945043	0	0	-3.243903	0.02583	0	0	0	0	0	0
109945301	LOC109945301	uncharacterized LOC109945301	-4.730336	0.006497	0	0	-3.15291	0.032713	0	0	0	0
109945549	LOC109945549	uncharacterized LOC109945549	0	0	0	0	0	0	0	0	3.0735527	0.02440533
109945745	LOC109945745	uncharacterized LOC109945745	0	0	0	0	1.5367287	0.043753	1.679339	0.0245183	0	0
109945802	LOC109945802	uncharacterized LOC109945802	-2.8296734	0.001818	-3.535388	0.00017	-3.4281519	0.000228	0	0	0	0
109945979	LOC109945979	uncharacterized LOC109945979	0	0	0	0	4.4555896	0.030481	0	0	0	0
109945989	LOC109945989	uncharacterized LOC109945989	0	0	0	0	0	0	0	0	4.0058059	0.01550202
109946011	LOC109946011	uncharacterized LOC109946011	0	0	0	0	1.8831542	0.003855	0	0	0	0
109946015	LOC109946015	uncharacterized LOC109946015	0	0	0	0	0	0	0	0	2.7882975	0.04136367
109946021	LOC109946021	uncharacterized LOC109946021	0	0	0	0	0	0	-3.445713	0.0127687	0	0
109946028	LOC109946028	uncharacterized LOC109946028	0	0	1.7091603	0.03324	0	0	0	0	0	0
110354864	LOC110354864	uncharacterized LOC110354864	0	0	0	0	1.8075532	0.034861	2.455985	0.004824	2.2662215	0.00747383
111347722	LOC111347722	uncharacterized LOC111347722	0	0	0	0	0	0	2.222258	0.0185581	0	0

			Yuc x Ctr		Azo x Ctr		AzoYuc x Ctr		AzoYuc x Yuc		AzoYuc x Azo	
**Gene ID**	**Gene Symbol**	**Gene Description**	**Log2(FC)**	**pvalue**	**Log2(FC)**	**pvalue**	**Log2(FC)**	**pvalue**	**Log2(FC)**	**pvalue**	**Log2(FC)**	**pvalue**
111589286	LOC111589286	uncharacterized LOC111589286	0	0	0	0	0	0	3.541446	0.0098631	0	0
111589300	LOC111589300	uncharacterized LOC111589300	0	0	-4.000066	0.01847	-4.0304125	0.0176	0	0	0	0
111589342	LOC111589342	uncharacterized LOC111589342	-2.014159	0.010723	0	0	0	0	0	0	0	0
111589344	LOC111589344	uncharacterized LOC111589344	0	0	0	0	4.9882214	0.018934	5.410956	0.0109056	0	0
111589557	LOC111589557	uncharacterized LOC111589557	0	0	0	0	3.4247195	0.008629	3.229708	0.0092567	0	0
111589728	LOC111589728	uncharacterized LOC111589728	0	0	0	0	1.53297	0.007508	0	0	0	0
111589793	LOC111589793	uncharacterized LOC111589793	0	0	0	0	1.9568078	0.022099	2.106972	0.0133199	1.9823283	0.01935484
111590133	LOC111590133	uncharacterized LOC111590133	0	0	0	0	0	0	3.8951	0.0256678	0	0
111590268	LOC111590268	uncharacterized LOC111590268	0	0	0	0	0	0	0	0	1.6784448	0.02028119
111590293	LOC111590293	uncharacterized LOC111590293	-4.6171341	1.74E-05	-4.862388	6.3E-06	-4.3444928	2.07E-05	0	0	0	0
111590552	LOC111590552	uncharacterized LOC111590552	0	0	0	0	0	0	2.46776	0.0485819	0	0
111590837	LOC111590837	uncharacterized LOC111590837	0	0	0	0	2.2849078	0.002329	2.311898	0.0017277	1.7826504	0.01346231
111591126	LOC111591126	uncharacterized LOC111591126	0	0	0	0	0	0	4.079076	0.0173614	0	0

			Yuc x Ctr		Azo x Ctr		AzoYuc x Ctr		AzoYuc x Yuc		AzoYuc x Azo	
**Gene ID**	**Gene Symbol**	**Gene Description**	**Log2(FC)**	**pvalue**	**Log2(FC)**	**pvalue**	**Log2(FC)**	**pvalue**	**Log2(FC)**	**pvalue**	**Log2(FC)**	**pvalue**
111591151	LOC111591151	uncharacterized LOC111591151	0	0	0	0	0	0	2.146276	0.0210223	0	0

	Yuc x Ctr	Azo x Ctr	AzoYuc x Ctr	AzoYuc x Yuc	AzoYuc x Azo
Down-regulated loci	135	134	147	76	52
Up-regulated loci	70	51	200	199	166
Total	205	185	347	275	218
Loci with LG2(FC) > 3	95	83	143	106	98
