# Supplementary material for: Effects on gene expression during maize-Azospirillum interaction in the presence of a plant-specific inhibitor of indole-3-acetic acid production
Source: Genet Mol Biol. 2023 Sep 18;46(3 Suppl 1):e20230100. doi: 10.1590/1678-4685-GMB-2023-0100 (PMC10510588; doi:10.1590/1678-4685-GMB-2023-0100)
Supplement: Table S4 - [file 1415-4757-GMB-46-3-s1-e20230100-s6.pdf]

Genetics and Molecular Biology

https://doi.org/10.1590/1678-4685-GMB-2023-0100

Supplementary Material to "Effects on gene expression during maize-*Azospirillum* interaction in the presence of a plant-specific inhibitor of indole-3-acetic acid production"

Table S4 - Maize differentially expressed genes that changed their pattern of expression from being down-regulated to up-regulated, and vice versa, when comparing two experimental conditions (Yuc x Ctr and AzoYuc x Yuc). Ctr = control plantlets; Yuc = plantlets that received 50 µM of yucasin; AzoYuc = plantlets that received 50 µM of yucasin and were inoculated with *A. brasilense* FP2.

			**Yuc x Ctr**	**AzoYuc x Yuc**
**Gene ID**	**Gene Symbol**	**Gene Description**	**Log2FoldChange**	**Log2FoldChange**
100274425	LOC100274425	Zmt*lc9* (TRAM/LAG/CRN8 9)	-17.48003338	20.0287248
100274519	LOC100274519	Cation/calcium exchanger 2	-6.769018936	3.744145032
100275495	LOC100275495	uncharacterized LOC100275495	-5.576511893	3.940338859
100280034	LOC100280034	putative cytochrome P450 superfamily protein	-5.570676273	2.888995861
100272879	LOC100272879	4-hydroxy-tetrahydrodipicolinate reductase 2 chloroplastic	-5.099107437	4.477736093
100383941	LOC100383941	er1 (erecta-like1)	-4.572904204	4.277854522
103642860	LOC103642860	flavanone 3-dioxygenase 2	-4.509657891	3.865355085
100285440	cl574_1	thic1 (hydroxymethylpyrimidine phosphate synthase1)	-4.464839214	4.834755493
103652813	LOC103652813	Win1	-4.321876905	1.97116866
100281450	LOC100281450	Bzip45 (bZIP-transcription factor 45)	-4.282338585	3.804437956
103647759	LOC103647759	L-type lectin-domain containing receptor kinase IX.1	-4.250283916	4.310202232
103637645	LOC103637645	ncRNA - uncharacterized LOC103637645	-4.21059218	5.137293422
103631128	LOC103631128	anthocyanidin 3-O-glucoside 6"-O-acyltransferase	-4.111989201	4.209651414
103632317	LOC103632317	ABC transporter B family member 28	-4.056802254	3.437832854

			Yuc x Ctr	AzoYuc x Yuc
**Gene ID**	**Gene Symbol**	**Gene Description**	**Log2FoldChange**	**Log2FoldChange**
103650615	LOC103650615	protein SUPPRESSOR OF GENE SILENCING 3 homolog	-4.007262958	5.494623332
100278838	LOC100278838	uncharacterized LOC100278838	-3.935859193	3.471509913
103630646	LOC103630646	uncharacterized LOC103630646	-3.834852286	3.533941968
109945065	LOC109945065	atherin-like	-3.813478066	4.574858702
109943608	LOC109943608	protein ILITYHIA	-3.741831645	3.484233486
103647445	LOC103647445	glutamate receptor 2.9	-3.71844012	3.893901986
100194033	LOC100194033	adenosine/AMP deaminase family protein	-3.622452195	4.868198679
103625826	LOC103625826	hypothetical protein	-3.526542267	3.269865307
100283804	LOC100283804	erwinia induced protein 1	-3.515181213	3.284399877
103647706	LOC103647706	G-type lectin S-receptor-like serine/threonine-protein kinase	-3.38804516	3.396528917
103633395	LOC103633395	uncharacterized LOC103633395	-3.381261263	3.670418312
100275369	TIDP3280	hypothetical protein	-3.299703791	3.288934564
109940248	LOC109940248	methyl-CpG-binding domain protein 2-like	-3.264709836	3.110573689
100284981	LOC100284981	uncharacterized LOC100284981	-3.160913416	4.768535977
100501616	LOC100501616	putative subtilase family protein	-2.99125157	2.930192294
100502267	LOC100502267	uncharacterized LOC100502267	-2.909092396	3.017376578
103647463	LOC103647463	ncRNA - uncharacterized LOC103647463	-2.815978371	2.667209498
100382339	LOC100382339	uncharacterized LOC100382339	-2.403798393	1.932993299
100217046	pco130711	uncharacterized LOC100217046	-2.212145214	2.514231063
109941899	LOC109941899	uncharacterized LOC109941899	-2.150063061	2.208436876
103647201	PARP1	poly (ADP-ribose) polymerase 1	-2.04805656	1.771278376
100381493	LOC100381493	ncRNA - uncharacterized LOC100381493	-2.03049272	1.559422798
109941581	LOC109941581	protein trichome berefringence-like 7	-1.991626282	2.291587592
100283644	IDP582	Flavonol synthase-like protein (NCBI) / GRMZM5G807276 (MaizeGDB)	-1.91046458	1.951633332
100382657	LOC100382657	Cinnamoyl-CoA reductase 1	-1.878923214	1.883677043
103648254	LOC103648254	lipid transfer protein	-1.844099681	1.826157289

			Yuc x Ctr	AzoYuc x Yuc
**Gene ID**	**Gene Symbol**	**Gene Description**	**Log2FoldChange**	**Log2FoldChange**
109945814	LOC109945814	translation initiation factor IF-2-like	-1.839424845	1.757408577
100283177	LOC100283177	hexose carrier protein HEX6	-1.823345462	1.913040354
100283764	LOC100283764	HVA22-like protein a	-1.786743629	2.179824809
103637736	LOC103637736	*rth6* (roothairless6)	-1.736659862	2.27833168
103647449	LOC103647449	ncRNA - uncharacterized LOC103647449	-1.733522378	1.848105258
100501569	LOC100501569	putative SPOC domain / Transcription elongation factor S-II protein	-1.709219073	1.868977388
103655625	LOC103655625	myosin-1-like	-1.688581832	1.873971975
109945706	LOC109945706	uncharacterized protein LOC109945706	-1.633765832	2.165740201
100278316	si946092e03	uncharacterized LOC100278316	1.557749514	-1.947572377
103647175	LOC103647175	snrk1a2 (SNF1-related kinase alpha1-like2) - GRMZM2G180704	1.571788814	-1.870917828
109942878	LOC109942878	50S ribosomal protein L2, chloroplastic	1.57254957	-3.205083012
100284115	LOC100284115	60S ribosomal protein L19-3	1.574161713	-1.912276895
100279000	LOC100279000	uncharacterized LOC100279000	1.629869323	-2.183624072
541628	pco084558	Coatomer subunit zeta-2	1.638332934	-1.645875795
100275554	cl40750_1	Phosphatidylserine decarboxylase proenzyme 3	1.67609769	-1.60545172
100382538	IDP1623	uncharacterized LOC100382538	1.731274107	-1.941016813
100216858	LOC100216858	putative vesicle-associated membrane protein family protein	1.73510139	-1.640046415
100273597	LOC100273597	cyp23 (cytochrome P-450 23)	1.739676366	-2.301779802
100501230	LOC100501230	putative leucine-rich repeat protein kinase family protein	1.750900906	-1.807466863
103638709	LOC103638709	116 kDa U5 small nuclear ribonucleoprotein component-like	1.769472095	-1.966045996
100193119	pco130460	uncharacterized LOC100193119	1.810965063	-1.883271914
109943107	LOC109943107	*Zmbbx34* (b-box 34)	1.818094716	-2.044188622
100284871	pco093706	uncharacterized LOC100284871	1.859795481	-1.546513221
100282128	sca1	*Zmaba2* (aka sca1 - short chain alcohol dehydrogenase 1)	1.869562727	-1.28031897
100383493	LOC100383493	uncharacterized LOC100383493	1.876210156	-1.542912322
541618	LOC541618	*Zmmpk5* (mitogen-activated protein kinase 5)	1.954828547	-2.371341104

			Yuc x Ctr	AzoYuc x Yuc
**Gene ID**	**Gene Symbol**	**Gene Description**	**Log2FoldChange**	**Log2FoldChange**
100382319	LOC100382319	bsdtf7 - BSD-transcription factor 7 (GRMZM2G111672)	2.007984542	-1.507402186
100277055	LOC100277055	uncharacterized LOC100277055	2.041940188	-2.53135101
542560	pco103506(726)	*Zmomt1* (oxo-glutarate/malate transporter1)	2.043864734	-2.036407645
103626490	LOC103626490	uncharacterized LOC103626490	2.074519828	-2.19758744
103636370	LOC103636370	protein MEN-8	2.079180991	-1.837693532
100216810	LOC100216810	P-loop containing nucleoside triphosphate hydrolase superfamily protein	2.083415793	-2.256633064
100381417	LOC100381417	dcl101 - dicer-like 101 (GRMZM2G040762) - envolvido na formação correta das anteras (Field 2016)	2.135590107	-1.839780076
100191806	pco069906	Proteasome subunit beta type-6	2.142134996	-1.911329116
103633165	LOC103633165	uncharacterized LOC103633165	2.200028795	-3.814438821
103625888	LOC103625888	40S ribosomal protein S27	2.207277241	-2.476887546
100193643	LOC100193643	uncharacterized LOC100193643	2.210261449	-2.478156016
103636340	LOC103636340	atg6b (autophagy6b)	2.21608073	-1.79853763
100273641	LOC100273641	translocon-associated protein alpha subunit	2.217726906	-1.950275169
100279889	gpm923	hypothetical protein	2.28623316	-2.504204901
111589818	LOC111589818	small nucleolar RNA U54	2.309775779	-1.738048265
103649936	LOC103649936	small G protein signaling modulator 1	2.368542136	-2.682352356
100284922	LOC100284922	RING-H2 finger protein ATL2B	2.420280097	-2.251764377
100191203	pco137008b	uncharacterized LOC100191203	2.49947534	-1.96150234
103646181	LOC103646181	DDB1- and CUL4-associated factor homolog 1	2.547750446	-2.051546175
103636567	LOC103636567	chaperone protein ClpB2, chloroplastic-like	2.585350927	-2.562051204
732835	HK1b2	histidine kinase	2.605591282	-2.232286009
100283321	cl421_1	uncharacterized LOC100283321	2.684348881	-2.755013315
100285433	LOC100285433	AER	2.692665871	-1.94649229
100193013	LOC100193013	uncharacterized LOC100193013	2.717871793	-2.589732624
100125658	umc1760	uncharacterized LOC100125658	2.7192843	-1.928599253

			Yuc x Ctr	AzoYuc x Yuc
**Gene ID**	**Gene Symbol**	**Gene Description**	**Log2FoldChange**	**Log2FoldChange**
100383497	LOC100383497	uncharacterized LOC100383497	2.724764145	-1.992879082
100384847	LOC100384847	Putative RING zinc finger domain superfamily protein	2.790838311	-3.016814452
100274135	LOC100274135	putative protein kinase superfamily protein	2.890230221	-2.638968878
100277571	LOC100277571	hypothetical protein	2.897993284	-3.288019003
100281425	LOC100281425	uncharacterized LOC100281425	2.912185182	-2.884318949
103633393	LOC103633393	pentatricopeptide repeat-containing protein, mitochondrial-like	2.947103466	-2.687199114
100272542	LOC100272542	Eukaryotic aspartyl protease family protein	2.954750965	-2.739674488
100283869	LOC100283869	uncharacterized LOC100283869	2.9919231	-2.265759851
100240693	Atg12	atg12 (autophagy12)	3.032824681	-3.289937502
103640631	LOC103640631	2-aminoethanethiol dioxygenase-like	3.156842235	-2.859041768
100284060	LOC100284060	mitochondrial carrier protein CGI-69	3.184216094	-3.254509864
100277761	LOC100277761	Remorin family protein	3.192961491	-2.274396153
100501018	LOC100501018	uncharacterized LOC100501018	3.205580513	-2.544339917
103643290	LOC103643290	uncharacterized LOC103643290	3.36822671	-2.849577273
100275527	LOC100275527	uncharacterized LOC100275527	3.45375998	-4.992061985
103646270	LOC103646270	*Zmbbx5* (B-box 5)	3.492512201	-2.558708195
109946070	LOC109946070	*Zmmkk5* (mitogen-activated protein kinase kinase 5)	3.511030994	-2.279009933
103651542	LOC103651542	bnlg1754	3.595997777	-2.269616502
100282349	LOC100282349	LSM7-like	3.68112702	-3.311927459
100127513	ZCN2	pebp2 (phosphatidylethanolamine-binding protein2)	3.681759523	-3.886772855
100217281	LOC100217281	Protein tesmin/TSO1-like CXC 4	3.778820344	-2.683512939
100281965	LOC100281965	uncharacterized LOC100281965	3.812324652	-4.0551332
100281298	si687036a06	uncharacterized LOC100281298	4.056146021	-2.433242827
100285688	LOC100285688	serine esterase family protein	4.197414877	-3.892549178
100283336	LOC100283336	CF9	4.283851274	-2.774805486
103635232	LOC103635232	rboh5 - respiratory burst oxidase 5 (GRMZM2G323731)	4.287186138	-5.589409125

			Yuc x Ctr	AzoYuc x Yuc
**Gene ID**	**Gene Symbol**	**Gene Description**	**Log2FoldChange**	**Log2FoldChange**
103632037	LOC103632037	uncharacterized LOC103632037	4.315708514	-3.64519037
111589804	LOC111589804	small nucleolar RNA Z105	4.435663484	-3.73229491
103649534	LOC103649534	nlp6 (NLP-transcription factor 6)	4.503522645	-2.370606102
103631736	LOC103631736	expansin-A15	4.580592805	-3.879190452
103653295	LOC103653295	Remorin family protein	4.633354556	-3.209847199
100277080	LOC100277080	*Zmtlc17* (TRAM/LAG/CRN8 17)	4.640957394	-3.136898104
100383923	LOC100383923	methyl binding domain123	4.766912244	-5.338287831
100285784	TIDP2770	uncharacterized LOC100285784	4.79387768	-3.831117978
100284077	pco109288	uncharacterized LOC100284077	4.825992067	-2.894919718
100286147	si605092h09	uncharacterized LOC100286147	6.045450336	-5.411068381
103645132	LOC103645132	wall-associated receptor kinase 3	6.443409219	-5.831684223
103639247	LOC103639247	uncharacterized LOC103639247	7.562166002	-6.524634766
103644320	LOC103644320	uncharacterized LOC103644320	7.62294481	-5.344869977
		Up-regulated loci	81	48
		Down-regulated loci	48	81
		Total	129	
		Uncharacterized	42	
