# Supplementary material for: Effects on gene expression during maize-Azospirillum interaction in the presence of a plant-specific inhibitor of indole-3-acetic acid production
Source: Genet Mol Biol. 2023 Sep 18;46(3 Suppl 1):e20230100. doi: 10.1590/1678-4685-GMB-2023-0100 (PMC10510588; doi:10.1590/1678-4685-GMB-2023-0100)
Supplement: Table S5 - [file 1415-4757-GMB-46-3-s1-e20230100-s7.pdf]

Genetics and Molecular Biology

https://doi.org/10.1590/1678-4685-GMB-2023-0100

Supplementary Material to "Effects on gene expression during maize-*Azospirillum* interaction in the presence of a plant-specific inhibitor of indole-3-acetic acid production"

Table S5 - *Azospirillum brasilense* differential Expressed Genes (DEGs) in the experimental condition evaluated. Genes that presented |Log2(FC) ≥ 1.5| and p-value ≤ 0.05 were considered DEGs. Azo = plantlets inoculated with *A. brasilense* FP2; AzoYuc = plantlets that received 50 µM of yucasin and were inoculated with *A. brasilense* FP2.

Experimental Condition	GeneID	Locus tag	Description	log2(FC)	pvalue
AzoYuc x Azo	56448279	OH82_RS04865	oxIT - oxalate/formate MFS antiporter	6.6862167	0.000443133
	56451403	OH82_RS20730	4-(cytidine 5'-diphospho)-2-C-methyl-D-erythritol kinase	5.4187731	0.005594081
	56450418	OH82_RS15740	PrkA family serine protein kinase	4.4623745	0.025934774
